# Bioinspired Synthesis of Alstoscholarinoids A and B

**DOI:** 10.1021/jacsau.5c00102

**Published:** 2025-03-03

**Authors:** Nicolas Kratena, Maximilian Kaiser, Kirill Naumov, Martin Waxmann, Peter Gaertner

**Affiliations:** Institute of Applied Synthetic Chemistry, TU Wien, Getreidemarkt 9, A-1060 Vienna, Austria

**Keywords:** Biomimetic synthesis, Triterpenoids, Hock rearrangement, Transannular aldol, Singlet oxygen

## Abstract

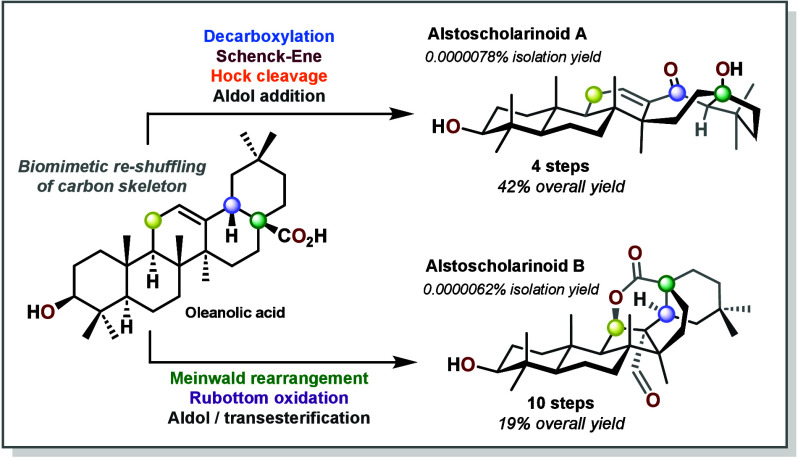

Biomimetic synthesis can be an attractive approach to access complex natural products by addressing challenging structural features through cascade reactions, which are inferred through tangible biosynthetic hypotheses. In some instances, the originally proposed structure or biosynthetic path might be revised through synthesis. In this communication we report a short and efficient bioinspired synthesis of Alstoscholarinoids A and B, rearranged triterpenes from the *Alstonia scholaris* tree. Salient features of the synthesis include a transannular aldol addition as well as a cascade consisting of a Schenck–Ene reaction, Hock rearrangement, and aldol addition. This culminated in a revision of the likely biosynthetic origin of Alstoscholarinoid A and a thorough exploration of the previously proposed intermediates.

Triterpenoids continue to be an important and interesting class of natural products due to their very diverse biological activities as well as interesting structural features.^1−4^ Since some common triterpenes are cheaply available in bulk quantities, semisynthetic approaches from biogenetically related compounds are an attractive option to approach the more complex members of this family.^5−8^ The rearranged *abeo* triterpenoids Alstoscholarinoids A (**1**) and B (**2**, see Scheme 1A) from the evergreen tree *Alstonia scholaris* attracted our attention due to their novel carbon frameworks, potent antihyperuricimic properties *in vivo*,^9^ and the extremely low isolation yield of ca. 0.00001% from dried leaves. The biosynthetic origin of both products (Scheme 1B) was proposed in the isolation report^10^ and traced these compounds back to oleanolic acid (**3**). Luo and co-workers suggested an intramolecular aldol/lactonization cascade to arrive at **2** and a transannular aldol addition to form the 7,5-ring system in **1**. For this to occur, the decarboxylated intermediate **4** would have to be oxidatively cleaved in a selective fashion to give diketone **5** en route to **1**. Likewise, the 12,13-alkene in oleanolic acid (**3**) would need to be isomerized to the corresponding 11,12-alkene **6** and cleaved oxidatively to reveal the dialdehyde **7**. During our own efforts in this area the groups of Shi,^11^ Wu,^12^ and Xie/Yan^13^ already disclosed semisyntheses of **2**, employing very similar bioinspired strategies by accessing derivatives of dialdehyde **7**, as in our own approach which is included in this manuscript. However, none of these reports mentioned any approaches toward **1**, which prompted us to disclose our findings concerning both Alstoscholarinoids in this communication. We planned to access desired aldol precursor **5** by first removing the C-28 carboxylic acid moiety in oleanolic acid to obtain diene **4** and then addressing the tetrasubstituted double bond with a variety of oxidation protocols to arrive at diketone **5**. From here a selective enolization at C-19 and addition toward the C-17 carbonyl would give rise to the desired product **1**. To date, only few examples of such selective, transannular aldol additions across macrocycles^14−18^ have been reported, posing a formidable challenge to be faced in this project. For the preparation of **2**, olefin isomerization processes toward **7** and different rearrangement reactions to realize the desired ring contraction were evaluated.

**Scheme 1:**
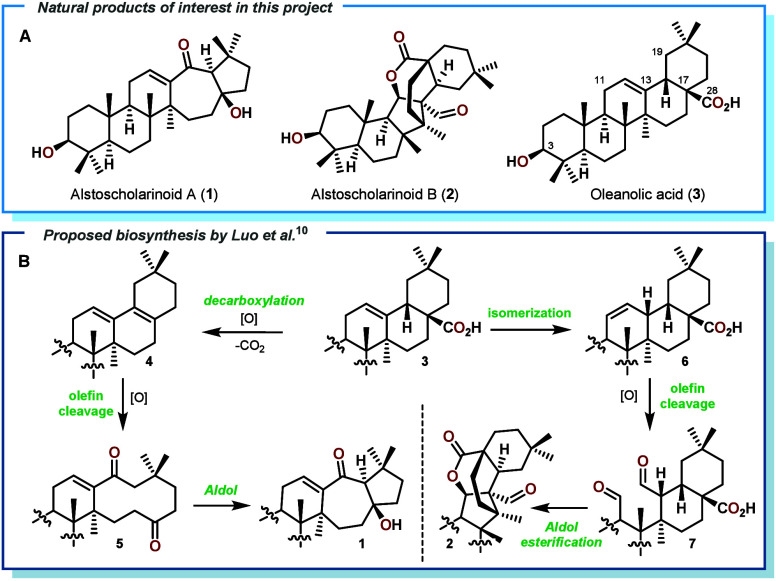
A: Structures of Relevant Natural Products and B: Proposed Biosynthesis of Compounds **1** and **2** According to the Isolation Report

Our initial efforts were focused on obtaining the designated precursor toward **1**, 10-membered diketone **5**. Starting from acetylated oleanolic acid **8** (Scheme 2), the C-17 carboxylic acid could be removed in high yield via an oxidative decarboxylation using Pb(OAc)_4_ (LTA) with Cu(OAc)_2_ and pyridine as additives.^19^ A mixture of regioisomeric olefins **9a**:**9b**:**9c** in the ratio 61:23:10 was obtained, favoring the desired tetrasubstituted compound **9a**. Alternatively, to avoid the toxic Pb(OAc)_4_, Ritter’s photoredox protocol^20^ could be employed to exclusively give the two isomers **9b** and **9c** in a 64:36 ratio. Gratifyingly, this was without consequence, as the mixture of isomers could simply be equilibrated to the thermodynamically stable natural product Aegiceradienol (**4**) by exposure to concentrated HCl at 50 °C. With this key intermediate in hand, the selective oxidation of the tetrasubstituted olefin was investigated. This challenging transformation could neither be achieved directly by ozonolysis nor cleavage with OsO_4_ and NaIO_4_. Selective epoxidation of the desired olefin was possible with *m*-CPBA (see SI) which instilled hope that other oxidation protocols could also be selective toward this olefin. After thorough investigation, only stoichiometric OsO_4_ was capable of affecting the desired dihydroxylation, as the formed osmate esters were recalcitrant to hydrolysis, ruling out catalytic approaches. The diol **10** was subsequently cleaved in excellent yield by exposure to Pb(OAc)_4_ or NaIO_4_ to deliver diketone **5**.^21^

**Scheme 2:**
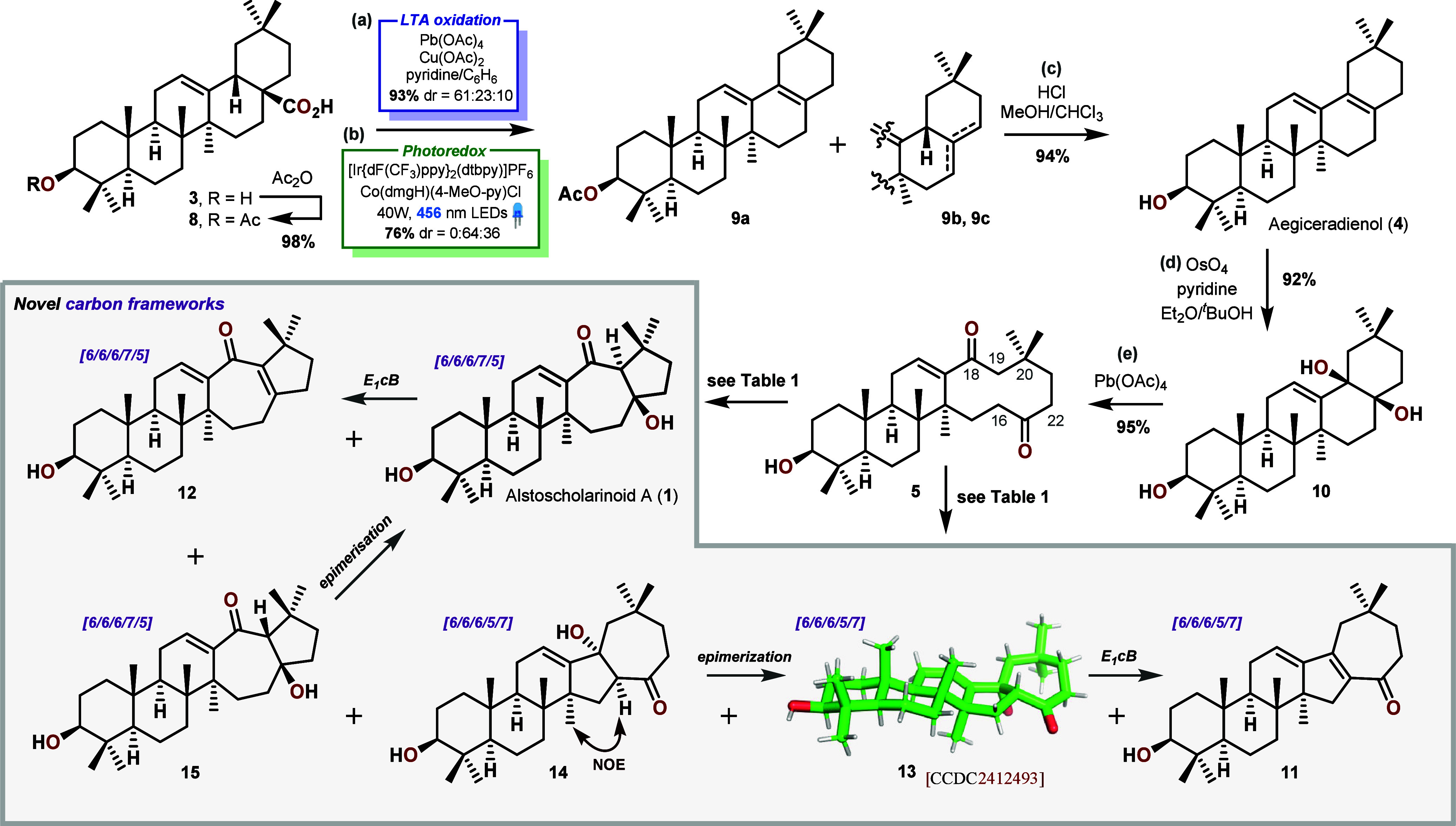
Transannular Diketone Approach toward Alstoscholarinoid A (**1**) (a) Pb(OAc)_4_ (1.2 equiv), Cu(OAc)_2_ (0.25 equiv), C_6_H_6_:Py = 10:1, 75 °C; (b) [Ir] (1 mol %), [Co] (5 mol %), Cs_2_CO_3_ (1.0 equiv), DME:H_2_O = 20:1, 30 °C, 456 nm LEDs; (c) 37% aq. HCl (50.0 equiv), MeOH:CHCl_3_ = 1:1, 50 °C; (d) 2.5% OsO_4_ in ^*t*^BuOH (1.0 equiv), Et_2_O:Py = 10:1, rt; (e) Pb(OAc)_4_ (1.1 equiv), C_6_H_6_, rt.

The bioinspired transannular aldol addition toward the target was attempted with a wide range of conditions (see Table 1 and SI), resulting in six identifiable products **1** and **11**–**15**. At first, standard aldol conditions using NaOH were used, which resulted in the formation of two eliminated dienones **11** and **12** as well as aldol addition product **13**. After careful separation of the minute amounts of **12** we could deduce its structural similarity to that of the desired natural product. A new, epimeric product **14** was formed when the reaction was performed at low temperatures or with amidine bases (entries 4 and 6). In line with initial expectations,^14−18^ the sterically hindered C-19–CH_2_ was seemingly only deprotonated in trace quantities, whereas enolate formation at C-16 resulted in a kinetically favored (5-membered) cyclization toward the C-18 ketone (**11**, **13**, **14**). The Thorpe–Ingold effect of the C-20 dimethyl likely further stabilizes a specific macrocyclic conformer, bringing these two carbons in proximity, thereby enabling the C-16 → C-18 cyclization rather than the putative C-22 → C-18 cyclization, which was not observed at all. The compounds **13**, **14**, and **11** possess a novel 6/6/6/5/7 carbon skeleton and were carefully characterized, including single-crystal X-ray diffraction of **13** (monotoluene solvate, CSD 2412493, see SI for details), and their reactivity was explored further to obtain information regarding putative retro-aldol reactions of these adducts. Unfortunately, product **14**, when treated with base, slowly epimerized to **13**, which eliminates to give **11** as the thermodynamic and kinetic sink. Enamine catalysis, of which l-proline^16^ was most efficient, delivered **13** in varying selectivities. When the reaction was in turn performed with an excess of amidine base like DBN or DBU at elevated temperatures in a nonpolar solvent (DCE or toluene), small amounts of the desired product **1** were formed initially, together with another isomer, identified as its C-19 epimer (**15**). Gratifyingly, prolonged heating of this mixture transformed **15** to **1** through epimerization. Exploiting this minor pathway under the same conditions, we were able to isolate Alstoscholarinoid A (**1**) in just a 9% yield from diketone **5**. While these results can be considered somewhat successful, the fact that the isomeric systems **13**–**15** were not found in nature suggested that **5** might not be the operational intermediate in the biosynthesis after all.

**Table 1:** Conditions for the Transannular Aldol Addition of Diketone **5** (Ratio Determined by ^1^H NMR)

#	Conditions (>3.0 equiv)	**11**	**12**	**13**	**14**	**15**	**1**
1	NaOH, EtOH, 25 °C, 5 min	59	3	38			
2	NaOH, EtOH, 25 °C, 1 day	97	1	2			
3	TFA, CD_2_Cl_2_, 25 °C, 1 day	99					
4	KOH, EtOH, –30 °C, 5 h	1		58	40		
5	l-Proline, DMSO, 50 °C, 1 day	3		96	1		
6	DBU, PhMe, 60 °C, 10 min			50	42	4	3
7	DBU, DCE, 65 °C, 5 min			58	31	6	5
8	DBN, PhMe, 60 °C, 5 min			71	20	5	4
9	DBN, DCE, 60 °C, 60 min			73	16		**10**

As the compounds were isolated exclusively from the leaves of *Alstonia scholaris*(22) we suspected the involvement of a photochemical step in the biosynthesis, perhaps analogous the biosynthesis of the antimalarial drug Artemisinin.^23,24^ In our revised biosynthesis, diene **4** serves as the precursor, which can engage singlet oxygen in a Schenck–Ene reaction,^25−27^ delivering the tertiary hydroperoxide **16** initially (see Scheme 3). The formation of the initial per-epoxy species on the β-face is consistent with other oxidation reactions on this system. The observed regioselectivity toward C-19 is suspected to arise in the presence of an axial H atom on the β-face at this position with only equatorial hydrogens being available at C-16 and C-22. Acidic activation of this hydroperoxide could trigger Hock cleavage^28−33^ of the C–C bond between carbons C-17 and C-18 to give hemiacetal **17**. Upon opening of the acetal, the correct enolate is formed, and the C-17 ketone is protonated, finally establishing the correct selectivity for the aldol addition. To test this hypothesis, a solution of diene **4** and TFA under oxygen was irradiated in the presence of different photosensitizers. To our delight, the desired natural product **1** was isolated in a one-pot fashion after workup and purification in 49% yield, without any of the other aldol addition products (**13**–**15**) present. The analytical data collected matched the isolation report for **1** (see SI). This type of Ene/Hock/Aldol cascade has been reported^34−36^ for cholesterol, the formerly mentioned related transformation of Artemisinin,^23,24^ but remains quite rare overall. To our knowledge, this is the first example of such a cascade culminating in the aldol reaction of two ketones.^37,38^ The mechanism proposed above is supported by isolation of the relevant intermediates hydroperoxide **16** and acetal **17**. The former serves as a point for further oxidative diversification of 28-nor-oleanes. For example, when the peroxide functionality in **16** was reduced with PPh_3_, the corresponding alcohol **18** is obtained (**18** could also be obtained by epoxidation and epoxide opening of **4**, see SI). Exposure of intermediates **4**, **16**, or **18** for prolonged reaction times with singlet oxygen led to [4 + 2] cycloaddition of the diene system to give a peroxy-bridged hydroperoxide (not depicted) which could be subsequently reduced with thiourea to **19** in 48% overall yield from **4**. The tertiary alcohol present in **18** could also be exploited to affect directed epoxidation under vanadium catalysis. The resulting epoxide **20** was readily eliminated to give **21** as another novel product and concluded the exploration of the decarboxylated oleanolic acid derivatives.

**Scheme 3:**
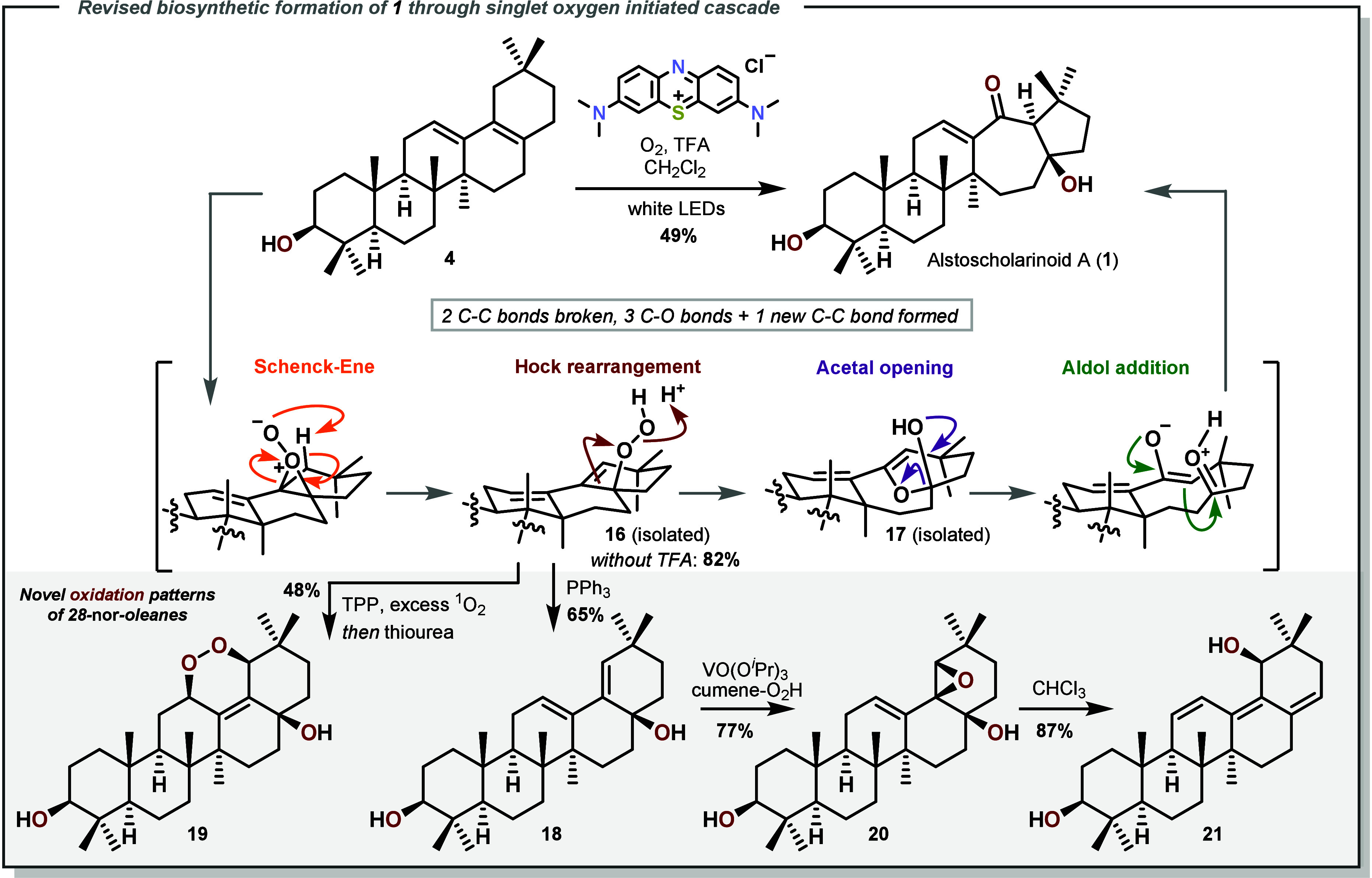
Revised Proposal for the Biosynthetic Formation of **1** through a Schenck–Ene/Hock/Aldol Cascade and Divergent Access to Novel Oleanes via Intermediate **16**

As indicated, we also developed a route toward the biosynthetic precursor to Alstoscholarinoid B (**2**), dialdehyde **7**. This was at first approached as an alkene isomerization problem of **3** to **6**. The C-11 α-alcohol (not shown) of protected oleanolic acid **22** was obtained by selective allylic oxidation.^39,40^ Regrettably, Mitsunobu conditions that could affect the required isomerization^41−43^ did not proceed, as large nucleophiles approaching the C-ring are likely blocked by adjacent axial methyl groups. It was then decided that accessing an 11,12-diol which could be cleaved to the desired dialdehyde would be a more forgiving strategy. The TBS-protected compound **22** (see Scheme 4) was thus carried to its C-12 ketone **23** by way of epoxidation and Meinwald rearrangement. The C-11/C-12 silyl enol ether was formed in quantitative yield and oxidized with *m-*CPBA to deliver the TMS-protected α-ketol **24**. Deprotection of the TMS silyl ether was followed by reduction of the ketone with NaBH_4_, giving *trans*-diol **25** in high yield. The C-3 protecting group was then removed under acidic conditions. Cleavage of the *trans*-diol was possible in our hands using Pb(OAc)_4_ or PhI(OAc)_2_ to give dialdehyde **7** in 74% yield over two steps.^44^ While our own experiments into the aldol cascade were ongoing, other reports on this target were published, which included optimization for this reaction. Thus, optimized conditions^11^ using DBU/toluene at 170 °C were applied to **7** and delivered the desired natural product. With this, a successful synthesis of Alstoscholarinoid B (**2**) was also achieved. The data collected again matched data provided in the isolation report and previous syntheses.^10−13^ In addition to this synthetic route, other approaches to trigger ring contraction were investigated, at first through an epoxyketone rearrangement^45−48^ (**28** to **29**, see SI for details). In analogy to the Hock cleavage observed during the synthesis of **1**, we were intrigued by the related Criegee rearrangement of secondary hydroperoxides, as explored by Kishi and Goodman, which could give rise to the desired C–C bond cleavage.^49,50^ The α-hydroperoxide **26** generated from photocatalytic^51^ allylic oxidation, however, did not undergo the desired rearrangement. Instead, a myriad of products was formed under different conditions, most prominently the epoxide **27** exhibiting a rearranged taraxerane skeleton.^52^

**Scheme 4:**
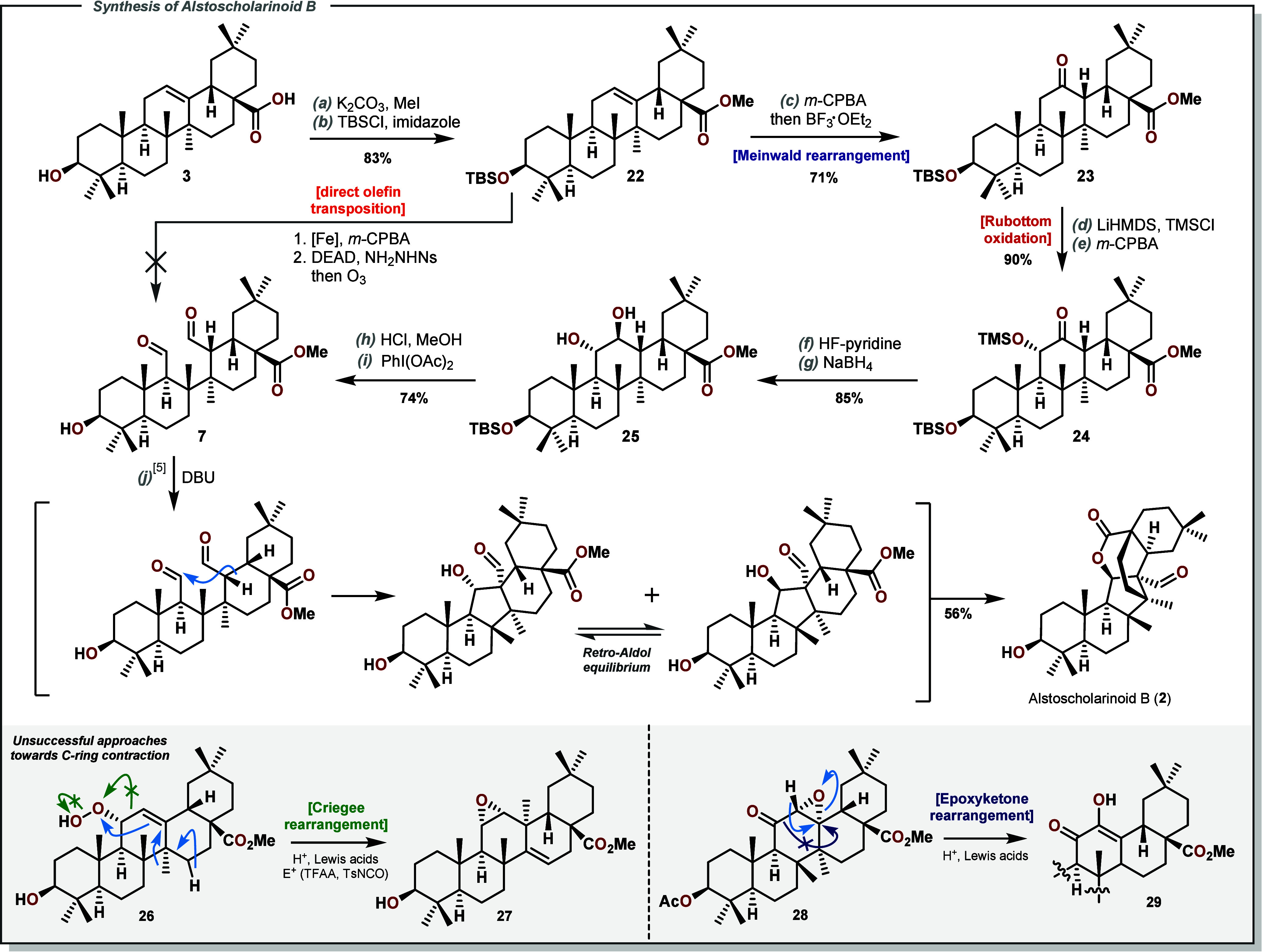
Synthesis of Dialdehyde **7** and Biomimetic Aldol/Retro-Aldol/Transesterification Cascade toward Alstoscholarinoid B (**2**) (a) CH_3_I (1.5 equiv), K_2_CO_3_ (1.1 equiv), acetone; (b) TBSCl (2.2 equiv), imidazole (3 equiv), DMF, 50 °C; (c) *m*-CPBA (2.1 equiv), CHCl_3_, then BF_3_·OEt_2_ (1.1 equiv), toluene, −30 °C; (d) LiHMDS (6.5 equiv), TMSCl (4 equiv), THF, −75 °C; (e) *m*-CPBA (1.5 equiv), CH_2_Cl_2_, 0 °C; (f) HF-Py (17.2 equiv), THF, rt; (g) NaBH_4_ (5.2 equiv), MeOH:THF = 1:1, 0 °C; (h) AcCl (50 equiv), MeOH:Et_2_O = 4:1; (i) PhI(OAc)_2_ (1.2 equiv), CHCl_3_, 0 °C to rt; (j) DBU:toluene = 2:1, 170 °C.

To summarize, several approaches for the biomimetic synthesis of Alstoscholarinoids A (**1**) and B (**2**) were evaluated and resulted in the efficient preparation of both compounds. For Alstoscholarinoid A (**1**), the transannular aldol addition was extensively investigated, resulting in modest quantities (7% over 6 steps) of the desired natural product. Furthermore, the most probable biosynthetic formation of **1** could be revised to a different precursor, Aegiceradienol (**4**), engaging in a regioselective Schenck–Ene/Hock/Aldol cascade with singlet oxygen. This process could be reproduced to great effect to deliver the product directly from **4**, resulting in a 42% overall yield of **1** in just 4 steps from oleanolic acid (**3**). Additionally, the sister natural product Alstoscholarinoid B (**2**) could be prepared in 10 steps and 19% overall yield with a bioinspired aldol/esterification cascade as the key step.

## Methods

### Procedure for the Ene/Hock/Aldol Cascade

In a 50 mL round-bottom flask was charged Aegiceradienol (**4**, 0.1 g, 0.24 mmol, 1.0 equiv), dissolved in CH_2_Cl_2_ (15 mL) and chilled to −15 °C. Tetraphenylporphyrin (1.5 mg, 2.4 μmol, 1 mol %) was introduced, followed by bubbling O_2_ through the solution. The flask was then irradiated with 30 W white LEDs, and trifluoroacetic acid (27 μL, 0.36 mmol, 1.5 equiv) was introduced after 5 min. The reaction was run under these conditions for 45 min before evaporating to dryness to provide the crude product. This residue was purified via column chromatography (7 g SiO_2_) using pentane:EtOAc = 5:1 as eluent to give 48 mg (45%) of Alstoscholarinoid (**1**). Additionally, 5 mg (5%) of acetal **17** wase isolated.

## Supporting Information Available

General information, synthetic routes, full optimization data for the aldol addition of **5**, detailed methods and spectroscopic data for compounds **1**–**2** and **4**–**29**, NMR comparison of synthetic and natural Alstoscholarinoid A and B, and NMR spectra (PDF)
X-ray analysis file for **13** (CIF)

## Supplementary Material

au5c00102_si_001.pdf

au5c00102_si_002.cif
