# Supplementary material for: Bioinspired Synthesis of Alstoscholarinoids A and B
Source: JACS Au. 2025 Mar 3;5(3):1076–82. doi: 10.1021/jacsau.5c00102 (PMC11938012; doi:10.1021/jacsau.5c00102)
Supplement: Supplementary file 1 — au5c00102_si_001.pdf [file au5c00102_si_001.pdf]

Bioinspired synthesis of Alstoscholarinoids A and B

Nicolas Kratena*[1], Maximilian Kaiser[1], Kirill Naumov[1], Martin Waxmann[1], Peter Gaertner[1]

[1]Institute of Applied Synthetic Chemistry, TU Wien, Getreidemarkt 9, A-1060, Vienna, Austria

Correspondence to: nicolas.kratena@tuwien.ac.at

Supplementary information

Table of Contents

General Experimental Techniques S5

Chemicals and solvents S5

Glassware and reaction conditions S5

Analytical techniques S5

Chromatography S5

X-ray crystallography S5

High-resolution mass spectrometry S6

Procedures S7

Synthesis of Aegiceradienol **4** S7

Scheme of synthetic route towards **4** S7

3-Acetyloleanolic acid, (4aS,6aS,6bR,8aR,10S,12aR,12bR,14bS)-10-acetoxy-2,2,6a,6b,9,9,12a-heptamethyl-1,3,4,5,6,6a,6b,7,8,8a,9,10,11,12,12a,12b,13,14b-octadecahydropicene-4a(2H)-carboxylic acid (**8**) S8

(3S,4aR,6aR,6bS,14aR,14bR)-4,4,6a,6b,11,11,14b-heptamethyl-1,2,3,4,4a,5,6,6a,6b,7,8,9,10,11,12,14,14a,14b-octadecahydropicen-3-yl acetate (**9a**, **9b**, **9c**) .. S9

Aegiceradienol, (3S,4aR,6aR,6bS,14aR,14bR)-4,4,6a,6b,11,11,14b-heptamethyl-1,2,3,4,4a,5,6,6a,6b,7,8,9,10,11,12,14,14a,14b-octadecahydropicen-3-ol (**4**) S11

Chemical oxidation of the Aegiceradienol S12

Scheme of oxidations of diene **4** S12

(4aS,6aS,6bR,8aR,10S,12aR,12bR,14bR)-2,2,6a,6b,9,9,12a-heptamethyl-1,2,3,4,5,6,6a,6b,7,8,8a,9,10,11,12,12a,12b,13-octadecahydro-4a,14b-epoxypicen-10-ol (**SI-2**) S13

(3S,4aR,6aR,6bS,8aS,14aR,14bR)-4,4,6a,6b,11,11,14b-heptamethyl-2,3,4,4a,5,6,6a,6b,7,8,9,10,11,14,14a,14b-hexadecahydropicene-3,8a(1H)-diol (**18**) S14

(2S,4aR,4bR,6bS,7aS,10aR,12aS,12bR,14aR)-1,1,4a,8,8,12a,12b-heptamethyl-1,2,3,4,4a,4b,5,7a,8,9,10,11,12,12a,12b,13,14,14a-octadecahydro-10aH-piceno[1,14b-b]oxirene-2,10a-diol (**20**) S15

(1S,6aS,6bR,8aR,10S,12aS,12bR)-2,2,6a,6b,9,9,12a-heptamethyl-1,2,3,5,6,6a,6b,7,8,8a,9,10,11,12,12a,12b-hexadecahydropicene-1,10-diol (**21**) S16

(3S,4aR,6aR,6bS,8aS,12aR,14aR,14bR)-4,4,6a,6b,11,11,14b-heptamethyl-1,2,3,4,4a,5,6,6a,6b,7,8,9,10,11,12,14,14a,14b-octadecahydropicene-3,8a,12a-triol (**4**) S17

(2S,4aR,4bR,14aS,14bR,16aR)-2-hydroxy-1,1,4a,9,9,14a,14b-heptamethyl-1,2,3,4,4a,4b,5,8,9,10,11,13,14,14a,14b,15,16,16a-octadecahydrocyclodeca[a]phenanthrene-7,12-dione (**5**) S18

Transannular Aldol reaction of 10-membered diketone **5**........ S19

Transannular aldol reaction scheme: S19

Alstoscholarinoid A, (2aR,4S,6aR,6bR,9aS,12aS,14aS,14bR)-4,12a-dihydroxy-3,3,6a,10,10,14a,14b-heptamethyl-1,2a,3,4,5,6,6a,6b,7,9a,10,11,12,12a,13,14,14a,14b-octadecahydroazuleno[6,5-a]phenanthren-9(2H)-one (**1**) S20

(2aR,4S,6aR,6bR,14aS,14bR)-4-hydroxy-3,3,6a,10,10,14a,14b-heptamethyl-1,2a,3,4,5,6,6a,6b,7,10,11,12,13,14,14a,14b-hexadecahydroazuleno[6,5-a]phenanthren-9(2H)-one (**12**) S24

(2aR,4S,6aR,6bR,8bR,13aS,14aS,14bR)-4,8b-dihydroxy-3,3,6a,10,10,14a,14b-heptamethyl-2,2a,3,4,5,6,6a,6b,7,8b,9,10,11,12,13a,14,14a,14b-octadecahydroazuleno[2,1-a]phenanthren-13(1H)-one (**14**) S25

(2aR,4S,6aR,6bR,8bR,13aR,14aS,14bR)-4,8b-dihydroxy-3,3,6a,10,10,14a,14b-heptamethyl-2,2a,3,4,5,6,6a,6b,7,8b,9,10,11,12,13a,14,14a,14b-octadecahydroazuleno[2,1-a]phenanthren-13(1H)-one (**13**) S26

(2aR,4S,6aR,6bR,14aS,14bR)-4-hydroxy-3,3,6a,10,10,14a,14b-heptamethyl-2,2a,3,4,5,6,6a,6b,7,9,10,11,12,14,14a,14b-hexadecahydroazuleno[2,1-a]phenanthren-13(1H)-one (**11**) S27

(3aR,6aS,6bR,8aR,10S,12aR,12bR,14bS)-10,14b-dihydroxy-2,2,6a,6b,9,9,12a-heptamethyl-2,3,3a,5,6,6a,6b,7,8,8a,9,10,11,12,12a,12b,13,14b-octadecahydroazuleno[5,4-a]phenanthren-4(1H)-one (**15**) S28

Photochemical reactions of diene **4**........ S29

Oxidation of **4** to hydroperoxide and divergent synthesis of oxidised oleanes: S29

(3S,4aR,6aR,6bS,8aS,14aR,14bR)-8a-hydroperoxy-4,4,6a,6b,11,11,14b-heptamethyl-1,2,3,4,4a,5,6,6a,6b,7,8,8a,9,10,11,14,14a,14b-octadecahydropicen-3-ol (**16**)........ S30

(2S,4aR,4bR,14aS,14bR,16aR)-1,1,4a,9,9,14a,14b-heptamethyl-1,3,4,4a,4b,5,9,10,11,13,14,14a,14b,15,16,16a-hexadecahydro-7,12-epoxycyclodeca[a]phenanthrene-2,12(2H)-diol (**17**) S31

Alstoscholarinoid A, (2aR,4S,6aR,6bR,9aS,12aS,14aS,14bR)-4,12a-dihydroxy-3,3,6a,10,10,14a,14b-heptamethyl-1,2a,3,4,5,6,6a,6b,7,9a,10,11,12,12a,13,14,14a,14b-octadecahydroazuleno[6,5-a]phenanthren-9(2H)-one (**1**) S32

(3aS,5aR,6aR,6bR,9S,10aR,12aR,12bS,14aR)-3,3,6b,10,10,12a,12b-heptamethyl-2,3,3a,5a,6,6a,6b,7,8,9,10,10a,11,12,12a,12b,13,14-octadecahydropiceno[1,14-cde][1,2]dioxine-9,14a(1H)-diol (**19**) S33

(6aR,6bR,9S,10aR,12aR,12bS)-3,3,6b,10,10,12a,12b-heptamethyl-1,2,3,6,6a,6b,7,8,9,10,10a,11,12,12a,12b,13-hexadecahydropiceno[1,14-cde][1,2]dioxin-9-ol (**SI-3**) S34

Unsuccessful routes towards Alstoscholarinoid B S35

Unsuccessful epoxyketone, Criegee rearrangement and Mitsunobu approaches S35

Methyl (4aS,6aS,6bR,8aR,10S,12aR,12bR,14bS)-10-acetoxy-2,2,6a,6b,9,9,12a-heptamethyl-1,3,4,5,6,6a,6b,7,8,8a,9,10,11,12,12a,12b,13,14b-octadecahydropicene-4a(2H)-carboxylate (**SI-4**) S36

Methyl (4aS,6aS,6bR,8aR,10S,12aS,12bR,13R,14bS)-10-acetoxy-13-hydroxy-2,2,6a,6b,9,9,12a-heptamethyl-1,3,4,5,6,6a,6b,7,8,8a,9,10,11,12,12a,12b,13,14b-octadecahydropicene-4a(2H)-carboxylate (**SI-5**) S37

Methyl (4aR,4bR,5aR,6aR,6bS,9S,10aR,12aR,12bS,14aS)-9-acetoxy-3,3,6b,10,10,12a,12b-heptamethyl-6-oxooctadecahydro-1H-piceno[12b,13-b]oxirene-14a(5aH)-carboxylate (**28**) ... S38

Methyl (4aS,6aS,6bR,8aR,10S,12aS,12bR,14bS)-10-acetoxy-14-hydroxy-2,2,6a,6b,9,9,12a-heptamethyl-13-oxo-1,3,4,5,6,6a,6b,7,8,8a,9,10,11,12,12a,12b,13,14b-octadecahydropicene-4a(2H)-carboxylate (**29**) S39

Methyl (4aS,6aS,6bR,8aR,10S,12aR,12bR,14bS)-10-hydroxy-2,2,6a,6b,9,9,12a-heptamethyl-1,3,4,5,6,6a,6b,7,8,8a,9,10,11,12,12a,12b,13,14b-octadecahydropicene-4a(2H)-carboxylate (**SI-6**) S40

Methyl (4aS,6aS,6bR,8aR,10S,12aS,12bR,13R,14bS)-13-hydroperoxy-10-hydroxy-2,2,6a,6b,9,9,12a-heptamethyl-1,3,4,5,6,6a,6b,7,8,8a,9,10,11,12,12a,12b,13,14b-octadecahydropicene-4a(2H)-carboxylate (**26**) S41

Methyl (4aS,6bR,8aR,10S,12aS,12bR,12cS,13aR,13bS,13cS)-10-hydroxy-2,2,6b,9,9,12a,13b-heptamethyl-1,3,4,5,6b,7,8,8a,9,10,11,12,12a,12b,12c,13a,13b,13c-octadecahydropiceno[13,14-b]oxirene-4a(2H)-carboxylate (**27**) S42

Synthesis of Alstoscholarinoid B S43

Methyl (4aS,6aS,6bR,8aR,10S,12aR,12bR,14bS)-10-((*tert*-butyldimethylsilyl)oxy)-*2,2,6a,6b,9,9,12a-heptamethyl-1,3,4,5,6,6a,6b,7,8,8a,9,10,11,12,12a,12b,13,14b-*octadecahydropicene-4a(2H)-carboxylate (**22**) S44

Methyl (4aS,6aR,6bR,8aR,10S,12aR,12bR,14aR,14bS)-10-((*tert*-butyldimethylsilyl)oxy)-2,2,6a,6b,9,9,12a-heptamethyl-14-oxoicosahydropicene-4a(2H)-carboxylate (**23**) S45

Methyl (4aS,6aR,6bR,8aR,10S,12aS,12bR,13S,14aR,14bS)-10-((*tert*-butyldimethylsilyl)oxy)-2,2,6a,6b,9,9,12a-heptamethyl-14-oxo-13-((trimethylsilyl)oxy)icosahydropicene-4a(2H)-carboxylate (**24**) S46

Methyl (4aS,6aR,6bR,8aR,10S,12aS,12bR,13S,14aR,14bS)-10-((*tert*-butyldimethylsilyl)oxy)-13-hydroxy-2,2,6a,6b,9,9,12a-heptamethyl-14-oxoicosahydropicene-4a(2H)-carboxylate (**SI-8**) .. S47

Methyl (4aS,6aR,6bR,8aR,10S,12aS,12bR,13S,14S,14aR,14bS)-10-((*tert*-butyldimethylsilyl)oxy)-13,14-dihydroxy-2,2,6a,6b,9,9,12a-heptamethylicosahydropicene-4a(2H)-carboxylate (**25**) ... S48

Methyl (1R,1'R,2R,2'R,4aS,4'aR,6'S,8aS,8'aS)-6'-((tert-butyldimethylsilyl)oxy)-1,1'-diformyl-2,2',5',5',7,7,8'a-heptamethyloctadecahydro-[2,2'-binaphthalene]-4a(2H)-carboxylate (**SI-9**) . S49

Methyl (4aS,6aR,6bR,8aR,10S,12aS,12bR,13S,14S,14aR,14bS)-10,13,14-trihydroxy-2,2,6a,6b,9,9,12a-heptamethylicosahydropicene-4a(2H)-carboxylate (**SI-10**) S50

Methyl (1R,1'R,2R,2'R,4aS,4'aR,6'S,8aS,8'aS)-1,1'-diformyl-6'-hydroxy-2,2',5',5',7,7,8'a-heptamethyloctadecahydro-[2,2'-binaphthalene]-4a(2H)-carboxylate (**7**) S51

Alstoscholarinoid B, (4aS,6aS,6bR,8aR,10S,12aS,12bR,13R,13aR,13bS)-10-hydroxy-2,2,6a,6b,9,9,12a-heptamethyl-15-oxooctadecahydro-13,4a-(epoxymethano)dibenzo[a,i]fluorene-13a(2H)-carbaldehyde (**2**).. S52

Spectra S56

References S90

General Experimental Techniques

Chemicals and solvents

Unless stated otherwise, all chemicals were purchased from commercial suppliers (Sigma-Aldrich, BLDPharm, TCI, abcr, Acros, Fisher, VWR, FluoroChem, Angene) and used without further purification. Some dry solvents (toluene, CH_2Cl_2, THF, Et_2O) were obtained from a PureSolv SPS system by Innovative Technologies. Dry MeCN, MeOH, pyridine, MTBE, dioxane and all other dry solvents were obtained from Acros Organics over molecular sieves and used without further purification. All other solvents used were HPLC grade or p.a. unless stated otherwise.

Glassware and reaction conditions

Reactions were carried out in round bottom flasks, oven-dried Schlenk flasks or microwave vials under an inert atmosphere (Argon) unless stated otherwise.

Analytical techniques

^{1}H, and ^{13}C NMR spectra were recorded on a Bruker AVIII 400 Spectrometer (^{1}H: 400 MHz and ^{13}C: 101 MHz) or a Bruker Avance III 600 (^{1}H: 600 MHz and ^{13}C: 151 MHz) in CD_2Cl_2, $CDCl_3$, DMSO-d_6, C_6D_6 or CD_3OD and referenced to residual solvent peaks. Chemical shifts δ are quoted in parts per million (ppm) to the nearest 0.01 for ^{1}H and 0.1 for ^{13}C, coupling constants *J* are quoted in Hz to the nearest 0.1 and splitting are recorded as singlet (s), doublet (d), triplet (t), quartet (q), pentet (p), hexet (h), heptet (hept), and multiplet (m). Assignments were based upon COSY, HSQC and HMBC experiments. Where unambiguous assignments could not be made the candidate positions are indicated by solidus "/". Due to very small quantities of product in some late-stage experiments (<1 mg) residual grease or solvent was observed in some of the NMR spectra. Any grease or residual solvent impurity will be indicated in the spectrum.

Chromatography

Analytical thin layer chromatography was performed on pre-coated silica gel aluminium sheets from Merck (TLC Silica Gel 60 F_{254}). Spots were visualized either by the quenching of UV fluorescence or by staining with phosphomolybdic acid/cerium sulfate, potassium permanganate or acidic *p*-anisaldehyde or vanillin solutions. Preparative column chromatography was carried out using Geduran Silica Gel 60 (40 – 63 µm) from Merck or LiChroprep® RP-18 (25 – 40 µm), which will be indicated as "fine silica". In cases where mixtures of solvents were used, the ratios refer to the component volumes. In cases where gradients where used, the start and the end ratio are stated.

X-ray crystallography

X-ray diffraction intensities of **13** were collected at T = 100 K in a dry stream of nitrogen on a STOE STADIVARI diffractometer system equipped with a Dectris Eiger CdTe hybrid photon counting

detector using Cu-*K*α radiation (λ = 1.54186 Å). Data were reduced with X-Area.[1] An absorption correction was applied with the multi-scan approach implemented in LANA. The structure was solved by the dual-space approach implemented in SHELXT[2]and refined against F^2 with SHELXL.[3] Hydrogen atoms connected to C were placed at calculated positions and refined as riding on their parent atom. Hydroxyl hydrogen atoms were located from difference Fourier maps and the O—H distance restrained to 0.870(2) Å.

High-resolution mass spectrometry

The HR-MS analysis was carried out from methanol or acetonitrile or water or a mixture of these solvents (concentration: 10 µM) by using an Agilent G7167B multi sampler, an Agilent G7120A binary pump with degasser, an Agilent G7116B oven and Agilent 6545 Q-TOF mass spectrometer equipped with a dual AJS ion score.

[1] X-Area 1.31.194.0, LANA 2.8.4 (STOE & Cie GmbH, Darmstadt, Germany, 2024).
[2] G. M. Sheldrick, *Acta Crystallogr.* 2015**,** A**71**, 3–8.
[3] G. M. Sheldrick, *Acta Crystallogr.* 2015**,** C**71**, 3–8.

Procedures

Synthesis of Aegiceradienol **4**

Scheme of synthetic route towards **4**

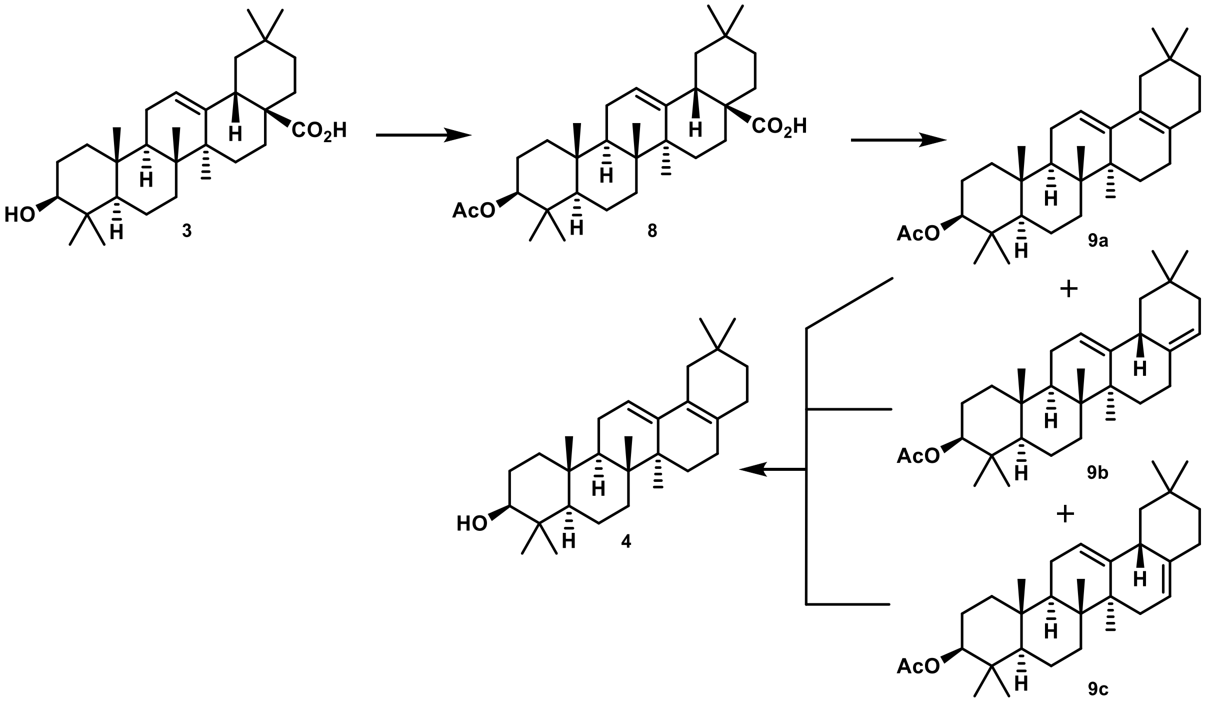

Scheme S1: Decarboxylation of oleanolic acid towards Aegiceradienol **4**

Decarboxylation was higher yielding when C-3 protected material **3** was used. A mixture of isomers **9a**-**c** consisting of mainly (71%) **9a** was converted in just 10 hours, in contrast a mixture consisting of **9c** and **9b** requires 48 hours to fully equilibrate the olefins to give pure **4**.

3-Acetyloleanolic acid, (4aS,6aS,6bR,8aR,10S,12aR,12bR,14bS)-10-acetoxy-2,2,6a,6b,9,9,12a-heptamethyl-1,3,4,5,6,6a,6b,7,8,8a,9,10,11,12,12a,12b,13,14b-octadecahydropicene-4a(2H)-carboxylic acid (8)

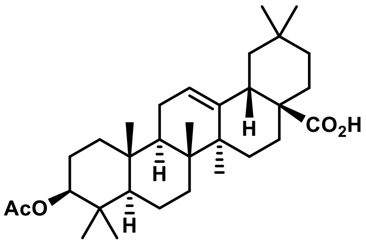

Chemical Formula: $C_{32}H_{50}O_4$
Exact Mass: 498,3709

In a 100-mL round-bottom flask was charged oleanolic acid (**3**) (95%, 4.00 g, 8.32 mmol, 1 eq.) in dry pyridine (50 mL) and treated with a catalytic amount of 4-dimethylaminopyridine (DMAP, ca. 5 mg) and acetic anhydride (2.28 mL, 2.47 g, 24.2 mmol, 2.9 eq.). The mixture was stirred at room temperature for 24 hours then evaporated to dryness. The crude residue was taken up with 1M HCl (50 mL) and CH_2Cl_2 (100 mL) and phases separated. The organic phase was washed once more with 1M HCl (10 mL), dried over $MgSO_4$ and evaporated. Treatment with heptane and subsequent evaporation of the solvent gave 4.10 g (98%) of the title compound **8** as an off-white solid.

Analytical data in accordance with literature.[1][2]

m.p.: 259 – 260 °C, Lit. 286 – 289 °C[1], 260 – 262 °C[2]

Optical rotation: $[\alpha]_D^{20}$= +68.1 (c 0.33, $CHCl_3$), Lit.: +69.4 (c 0.3, $CHCl_3$)[1]

^{1}H NMR (400 MHz, $CDCl_3$) δ = 11.61 (br s, 1H), 5.27 (t, *J* = 3.6, 1H), 4.51 – 4.44 (m, 1H), 2.82 (dd, *J* = 13.9, 4.6, 1H), 2.04 (s, 3H), 1.97 (td, *J* = 13.4, 4.0, 1H), 1.92 – 1.84 (m, 2H), 1.82 – 1.68 (m, 2H), 1.68 – 1.52 (m, 8H), 1.41 (m, 2H), 1.37 – 1.29 (m, 2H), 1.29 – 1.20 (m, 2H), 1.21 – 1.15 (m, 1H), 1.12 (s, 3H), 1.07 (dt, *J* = 13.3, 3.1, 1H), 0.94 (s, 3H), 0.92 (s, 3H), 0.90 (s, 3H), 0.86 (s, 3H), 0.85 (s, 3H), 0.83 (s, 1H), 0.74 (s, 3H).

^{13}C NMR (151 MHz, $CDCl_3$) δ = 184.1, 171.2, 143.7, 122.7, 81.1, 55.4, 47.7, 46.7, 46.0, 41.7, 41.1, 39.4, 38.2, 37.8, 37.1, 33.9, 33.2, 32.7, 32.6, 30.8, 28.2, 27.8, 26.0, 23.7, 23.7, 23.5, 23.0, 21.5, 18.3, 17.3, 16.8, 15.5.

HR-MS (ESI): Calculated for $C_{32}H_{51}O_4$ $[M+H]^+$ 499.3782, found: 499.3780.

(3S,4aR,6aR,6bS,14aR,14bR)-4,4,6a,6b,11,11,14b-heptamethyl-1,2,3,4,4a,5,6,6a,6b,7,8,9,10,11,12,14,14a,14b-octadecahydropicen-3-yl acetate (**9a**, **9b**, **9c**)

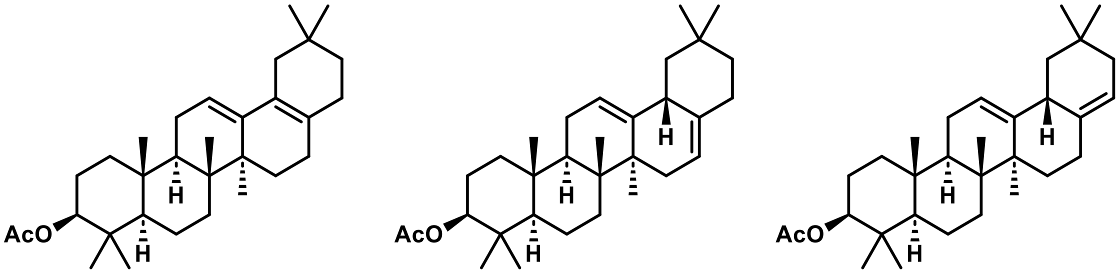

9a **9b** **9c**

Chemical Formula: $C_{31}H_{48}O_2$
Exact Mass: 452,3654

Procedure A:

In a 250-mL round-bottom flask was charged acetylated oleanolic acid **8** (1.95 g, 3.9 mmol, 1 eq.) in benzene (60 mL) and pyridine was added (6 mL), followed by $Cu(OAc)_2$ (177 mg, 0.98 mmol, 0.25 eq.) and the mixture was warmed to 75 °C in an oil bath (under argon). To the hot mixture $Pb(OAc)_4$ (2.08 g, 4.69 mmol, 1.2 eq.) was added in portions over 1 hour. After 1.5 hours the reaction was complete on TLC, cooled to room temperature and quenched by addition of sat. NH_4Cl solution. The reaction mixture was then extracted with Et_2O (3x) and washed with water. The crude material was purified by column chromatography (31 g SiO_2) using pentane/EtOAc = 15/1 to give a mixture (61:29:10, 1.64 g, 93%) of **9a**:**9b**:**9c** as a white solid. A previous run yielded a mixture with dr 74:21:5.

Analytical data in accordance with literature.[3]

Selected spectroscopic peaks for **9a**:

^{1}H NMR (600 MHz, $CDCl_3$) δ = 5.50 (dd, *J* = 5.0, 3.1, 1H), 4.54 – 4.44 (m, 1H), 2.04 (s, 3H), 2.01 – 1.94 (m, 2H), 1.90 – 1.85 (m, 1H), 1.76 – 1.53 (m, 6H), 1.50 (dt, *J* = 8.4, 6.6, 2H), 1.41 – 1.28 (m, 3H), 0.98 (s, 3H), 0.96 (s, 3H), 0.89 (s, 3H), 0.86 (s, 3H), 0.86 (s, 3H), 0.86 (s, 3H), 0.86 (s, 3H).

^{13}C NMR (151 MHz, $CDCl_3$) δ = 171.2, 140.4, 129.2, 125.4, 116.6, 81.0, 55.7, 47.4, 41.2, 39.7, 38.7, 38.6, 37.9, 37.0, 35.4, 33.9, 29.6, 29.4, 29.0, 28.5, 28.4, 28.2, 27.4, 23.7, 23.6, 21.5, 21.13, 18.39, 17.0, 16.9, 16.2.

HR-MS (ESI): Calculated for $C_{31}H_{49}O_2$ $[M+H]^+$ 453.3727, found: 453.3727.

Procedure B:

In a microwave vial was charged acetylated oleanolic acid **8** (150 mg, 0.3 mmol, 1 eq.), $(Ir[dF(CF_3)ppy]_2(dtbpy))PF_6$ (1 mol%, 3.4 mg, 3.0 μmol), $[Co(dmgH)_2(4\text{-MeO-py})]Cl$ (5 mol%, 6.5 mg, 15 μmol)[4] and cesium carbonate (98 mg, 0.3 mmol, 1 eq.). The atmosphere was exchanged for argon and the solid put under vacuum and argon (3x). DME (3 mL) and water (0.17 mL) were then added, and the mixture was stirred at 1000 rpm under irradiation of 40W blue LEDs (456 nm Kessil lamp). The mixture was stirred for 18 hours before being transferred and evaporated to dryness. The crude

material was purified by column chromatography (12 g SiO_2) using pentane/EtOAc = 10/1 to give a mixture (65:35, 104 mg, 76%) of **9b**:**9c** as a white solid. Additionally, γ-lactone **SI-1** (21 mg, 14%) could be isolated as a side-product.[5]

Selected spectroscopic peaks for **9b**:

^{1}H NMR (600 MHz, $CDCl_3$) δ = 5.37 (m, 1H), 4.53 – 4.47 (m, 1H), 2.70 (d, *J* = 12.8, 1H), 2.32 – 2.12 (m, 2H), 2.05 (s, 1H), 2.05 (s, 3H), 1.97 – 1.83 (m, 1H), 1.07 (s, 3H), 1.01 (s, 3H), 0.98 (s, 3H), 0.96 (s, 3H), 0.91 (s, 3H), 0.88 (s, 3H), 0.87 (s, 3H),

^{13}C NMR (151 MHz, $CDCl_3$) δ = 171.2, 143.3, 137.1, 121.2, 117.2, 81.0, 55.6, 48.1, 42.9, 39.9, 38.7, 38.5, 37.9, 37.1, 33.9, 33.0, 31.8, 31.5, 30.5, 28.3, 28.2, 24.4, 24.0, 23.8, 23.4, 23.3, 21.5, 18.3, 17.6, 17.0, 16.0.

Selected spectroscopic peaks for **9c**:

^{1}H NMR (600 MHz, $CDCl_3$) δ = 5.37 (m, 1H), 5.35 – 5.33 (m, 1H), 4.53 – 4.47 (m, 1H), 2.83 (m, 1H), 2.07 (dd, *J* = 4.5, 2.3, 1H), 2.05 (s, 3H), 1.81 – 1.75 (m, 1H), 0.93 (s, 3H), 0.88 (s, 3H), 0.87 (s, 3H), 0.86 (s, 3H), 0.86 (s, 3H).

^{13}C NMR (151 MHz, $CDCl_3$) δ = 171.2, 145.2, 135.7, 118.95, 117.8, 81.0, 55.7, 51.7, 47.5, 44.4, 43.1, 39.4, 39.0, 38.4, 37.9, 37.13, 37.11, 33.6, 32.1, 29.9, 27.1, 25.5, 23.8, 18.4, 16.9, 16.7, 16.0.

HR-MS (ESI): Calculated for $C_{31}H_{49}O_2$ $[M+H]^+$ 453.3727, found: 453.3730.

γ-lactone **SI-1**: Data in accordance with literature.[5]

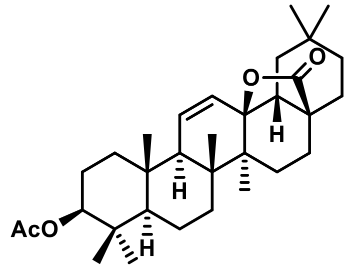

Chemical Formula: $C_{32}H_{48}O_4$
Exact Mass: 496,3553

^{1}H NMR (400 MHz, $CDCl_3$) δ = 6.02 (dd, *J* = 10.3, 1.7, 1H), 5.41 (dd, *J* = 10.3, 3.2, 1H), 4.59 – 4.37 (m, 1H), 2.19 – 2.07 (m, 2H), 2.05 (s, 3H), 1.91 (t, *J* = 2.5, 1H), 1.86 (d, *J* = 3.5, 1H), 1.87 – 1.77 (m, 2H), 1.78 – 1.50 (m, 6H), 1.43 – 1.15 (m, 7H), 1.05 (s, 2x 3H), 1.03 – 0.99 (m, 1H), 0.97 (s, 3H), 0.93 (s, 3H), 0.87 (s, 3H), 0.86 (s, 3H), 0.85 (s, 3H), 0.81 (d, *J* = 3.3, 1H).

^{13}C NMR (101 MHz, $CDCl_3$) δ = 180.1, 171.1, 135.8, 127.2, 89.9, 80.7, 55.0, 53.2, 50.7, 44.1, 41.7, 41.5, 38.0, 38.0, 37.4, 36.4, 34.5, 33.4, 31.6, 31.2, 27.8, 27.3, 25.5, 23.7, 23.4, 21.4, 19.1, 18.4, 18.1, 17.6, 16.2.

HR-MS (ESI): Calculated for $C_{32}H_{48}O_4$ $[M+H]^+$ 497.3626, found: 497.3632.

Aegiceradienol, (3S,4aR,6aR,6bS,14aR,14bR)-4,4,6a,6b,11,11,14b-heptamethyl-1,2,3,4,4a,5,6,6a,6b,7,8,9,10,11,12,14,14a,14b-octadecahydropicen-3-ol (**4**)

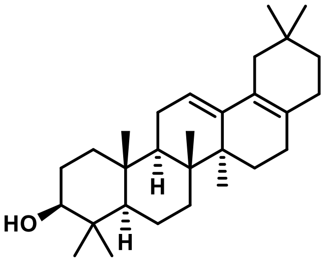

Chemical Formula: $C_{29}H_{46}O$
Exact Mass: 410,3549

In a 50-mL round-bottom flask was charged a mixture of olefins **9a**:**9b**:**9c** (from Procedure A or B, see above, 1.65 g, 3.64 mmol, 1.0 eq.) and dissolved in methanol/chloroform (150 mL, 1:1). Concentrated HCl (37%, 18.0 g, 182 mmol, 50 eq.) was introduced via pipette and the mixture was warmed to 50 °C in an oil-bath. After 18-48 hours (depending on the composition of the original mixture) at that temperature the reaction was diluted with water (10 mL) and extracted with dichloromethane (3x 50 mL). Organic phases washed with Na_2CO_3 solution and dried over $MgSO_4$. The crude product was crystallized from diisopropylether to give 1.40 g (94%) of Aegiceradienol **4** as a white solid.
Analytical data in accordance with literature.[6][7]

m.p.: 185 °C, Lit.: 191 – 192 °C[6] and 185 – 188 °C[7]

Optical rotation: $[\alpha]_D^{20}$= +78.5 (c 0.5, $CHCl_3$), Lit.:[6] +74.3 (c 0.33, C_6H_6)

^{1}H NMR (600 MHz, $CDCl_3$) δ = 5.52 (dd, *J* = 5.1, 3.1, 1H), 3.23 (dd, *J* = 11.3, 5.0, 1H), 2.15 – 1.93 (m, 5H), 1.89 (dd, *J* = 17.5, 5.6, 1H), 1.76 – 1.67 (m, 2H), 1.66 – 1.47 (m, 8H), 1.45 – 1.27 (m, 5H), 1.25 (m, 1H), 1.03 (m, 1H), 1.02 – 0.99 (m, 1H), 1.00 (s, 3H), 0.97 (s, 3H), 0.90 (s, 3H), 0.87 (s, 6H), 0.79 (s, 3H), 0.77 (dd, *J* = 11.8, 1.9, 1H).

^{13}C NMR (151 MHz, $CDCl_3$) δ = 140.4, 129.3, 125.4, 116.7, 79.2, 55.6, 47.5, 41.3, 39.7, 38.95, 38.92, 38.7, 37.2, 35.4, 34.0, 29.6, 29.4, 29.1, 28.5, 28.4, 28.2, 27.44, 27.40, 23.6, 21.2, 18.5, 17.0, 16.1, 15.8.

HR-MS (ESI): Exact mass calculated for $C_{29}H_{47}O$ $[M+H]^+$ 411.3622, found: 411.3613.

Chemical oxidation of the Aegiceradienol

Scheme of oxidations of diene **4**

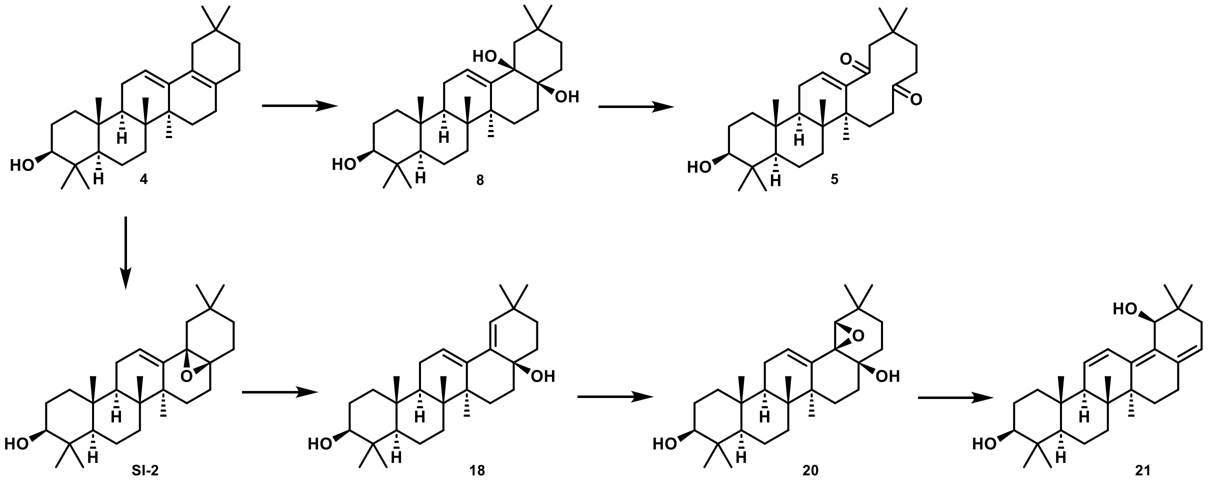

Scheme S2: Selective oxidation reactions of fully substituted double bond in **4**

Oxidative cleavage of epoxide **SI-2** (H_5IO_6, $NaIO_4$) was not possible in this system to circumvent use of stoichiometric OsO_4 to reach diketone **5**. Epoxyalcohol **20** could not be hydrolysed or attacked by nucleophiles in a selective manner, decomposition was marked by elimination reactions, forming extended π-systems across rings C, D and E with 2-3 double bonds, similar to **21**.

(4aS,6aS,6bR,8aR,10S,12aR,12bR,14bR)-2,2,6a,6b,9,9,12a-heptamethyl-1,2,3,4,5,6,6a,6b,7,8,8a,9,10,11,12,12a,12b,13-octadecahydro-4a,14b-epoxypicen-10-ol (**SI-2**)

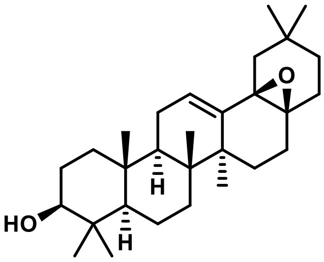

Chemical Formula: $C_{29}H_{46}O_2$
Exact Mass: 426,3498

In a 100-mL round bottom flask was charged diene **4** (280 mg, 0.68 mmol, 1.0 eq.) in CH_2Cl_2 (30 mL) and chilled to 0 °C in an ice-bath. Next, *m*-CPBA (77%, 156 mg, 0.7 mmol, 1.02 eq.) was introduced in portions and the mixture stirred for 45 minutes. The reaction was then quenched by the addition of saturated aqueous $Na_2S_2O_3$ and $NaHCO_3$ solutions and extracted with CH_2Cl_2. The crude product after evaporation of the solvent was purified by column chromatography (20 g SiO_2) using pentane/EtOAc = 10/1 as eluent to give 192 mg (66%) pure epoxide **SI-2** as a light yellow solid.

m.p.: 191 – 192 °C

Optical rotation: $[\alpha]_D^{20}$= +36.6 (c 0.41, CH_2Cl_2)

^{1}H NMR (600 MHz, C_6D_6) δ = 5.87 (dd, *J* = 4.7, 3.0, 1H), 3.00 (dd, *J* = 10.9, 5.0, 1H), 2.08 (d, *J* = 14.6, 1H), 1.93 – 1.77 (m, 6H), 1.73 – 1.64 (m, 2H), 1.59 (td, *J* = 12.4, 5.2, 1H), 1.55 – 1.37 (m, 6H), 1.33 (td, *J* = 12.5, 3.7, 1H), 1.27 (ddd, *J* = 12.9, 11.5, 3.1, 1H), 1.01 (s, 3H), 1.00 – 0.98 (m, 1H), 0.97 (s, 3H), 0.95 – 0.94 (m, 1H), 0.93 (s, 3H), 0.87 (s, 3H), 0.82 (s, 3H), 0.80 (s, 2x 3H), 0.79 – 0.76 (m, 1H), 0.65 (dd, *J* = 11.7, 1.9, 1H).

^{13}C NMR (151 MHz, C_6D_6) δ = 142.1, 125.5, 78.5, 60.7, 59.7, 56.0, 48.0, 43.1, 41.3, 39.1, 39.0, 38.8, 37.3, 34.4, 32.2, 31.7, 29.0, 28.4, 28.0, 27.8, 27.7, 27.0, 23.7, 23.1, 22.4, 18.7, 17.3, 16.1, 16.0.

HR-MS (ESI): Calculated for $C_{29}H_{47}O_2$ $[M+H]^+$ 427.3571, found: 427.3578.

(3S,4aR,6aR,6bS,8aS,14aR,14bR)-4,4,6a,6b,11,11,14b-heptamethyl-2,3,4,4a,5,6,6a,6b,7,8,9,10,11,14,14a,14b-hexadecahydropicene-3,8a(1H)-diol (**18**)

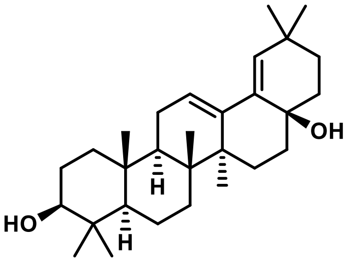

Chemical Formula: $C_{29}H_{46}O_2$
Exact Mass: 426,3498

Procedure A (from **SI-2**): In a 10-mL round bottom flask was charged epoxide **SI-2** (50 mg, 0.11 mmol, 1.0 eq.) in non-stabilised chloroform (4 mL) and stirred over 24 hours. Conversion was checked by ^{1}H-NMR and upon complete reaction the mixture was directly purified by column chromatography (4 g SiO_2) using pentane/EtOAc = 10/1 to give 42 mg (84%) of the dienol **18** as a white solid.

Procedure B (from **16**): In a 10-mL round bottom flask was charged hydroperoxide **16** (40 mg, 0.14 mmol, 1.0 eq.) and dissolved in dry THF (4 mL). At room temperature triphenylphosphine (72 mg, 0.27 mmol, 2.0 eq.) was introduced and the mixture stirred for 3 hours. The reaction mixture was concentrated and subsequently purified by column chromatography (5 g SiO_2) using pentane/EtOAc = 5/1 as eluent to give 25 mg (65%) of dienol **18**.

m.p.: 202 – 203 °C

Optical rotation: $[\alpha]_D^{20}$= +197.2 (c 0.25, CH_2Cl_2)

^{1}H NMR (400 MHz, CD_2Cl_2) δ = 5.57 (dd, *J* = 5.1, 2.7, 1H), 5.40 (d, *J* = 1.6, 1H), 3.18 (dd, *J* = 9.2, 7.0, 1H), 2.06 (td, *J* = 13.6, 4.5, 1H), 1.96 (dd, *J* = 11.9, 2.7, 1H), 1.85 (dt, *J* = 18.5, 5.2, 1H), 1.74 – 1.64 (m, 3H), 1.63 – 1.50 (m, 8H), 1.46 – 1.34 (m, 3H), 1.19 – 1.12 (m, 2H), 1.03 – 0.98 (m, 1H), 0.99 (s, 6H), 0.97 (s, 12H), 0.77 (s, 3H), 0.75 (d, *J* = 1.9, 1H).

^{13}C NMR (151 MHz, CD_2Cl_2) δ = 142.4, 139.5, 136.0, 122.6, 79.2, 68.9, 55.9, 48.1, 44.1, 39.41, 39.35, 39.2, 37.5, 35.9, 35.7, 34.7, 33.3, 33.0, 31.2, 28.4, 27.9, 27.2, 26.2, 24.1, 22.4, 18.8, 17.9, 16.3, 16.0.

HR-MS (ESI): Calculated for $C_{29}H_{47}O_2$ $[M+H]^+$ 427.3571, found: 427.3561.

(2S,4aR,4bR,6bS,7aS,10aR,12aS,12bR,14aR)-1,1,4a,8,8,12a,12b-heptamethyl-1,2,3,4,4a,4b,5,7a,8,9,10,11,12,12a,12b,13,14,14a-octadecahydro-10[a]H-piceno[1,14b-b]oxirene-2,10a-diol (20)

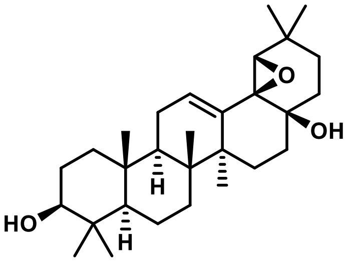

Chemical Formula: $C_{29}H_{46}O_3$
Exact Mass: 442,3447

In a Schlenk flask was charged dienol **18** (34 mg, 0.08 mmol, 1.0 eq.) and 4Å molecular sieves (ca. 100 mg). The mixture was suspended with dichloromethane (3 mL) before a stock solution of vanadium isopropoxide (12 mg in 4 mL dry CH_2Cl_2 → 1.6mL, 0.02 mmol, 0.25 eq.) was introduced at 0 °C. To the chilled mixture was added cumene hydroperoxide (45 μL, 0.24 mmol, 3.0 eq.) and it was stirred for 2.5 hours. After this time the reaction was quenched by addition of saturated $Na_2S_2O_3$ and $NaHCO_3$ solutions. Extraction with CH_2Cl_2 (3x) gave the crude product, which was purified (3 g SiO_2) using pentane/EtOAc = 10/1 to 5/1 to give 27 mg (77%) of the epoxyalcohol **20** as a colourless oil.

Optical rotation: $[\alpha]_D^{20}$= +48.2 (c 0.5, CH_2Cl_2)
^{1}H NMR (600 MHz, CD_2Cl_2) δ = 5.57 (dd, *J* = 5.2, 2.5, 1H), 3.31 – 3.10 (m, 1H), 2.44 (s, 1H), 2.09 (td, *J* = 13.0, 4.1, 1H), 1.99 – 1.93 (m, 1H), 1.90 (ddd, *J* = 18.1, 11.7, 2.6, 1H), 1.83 (dt, *J* = 18.1, 5.3, 1H), 1.67 (dt, *J* = 13.3, 3.7, 1H), 1.57 – 1.51 (m, 5H), 1.44 – 1.26 (m, 8H), 1.19 – 1.14 (m, 1H), 1.08 (s, 3H), 1.05 (s, 3H), 1.02 (s, 3H), 0.98 (s, 2x 3H), 0.96 (s, 3H), 0.77 (s, 3H), 0.77 – 0.74 (m, 1H).
^{13}C NMR (151 MHz, CD_2Cl_2) δ = 139.4, 119.3, 79.2, 76.8, 71.6, 64.8, 55.9, 48.2, 44.4, 39.6, 39.4, 39.3, 37.5, 34.48, 34.45, 33.6, 32.2, 30.0, 28.6, 28.5, 27.9, 26.0, 25.2, 23.2 (2C), 18.8, 17.8, 16.3, 16.0.
HR-MS (ESI): Calculated for $C_{29}H_{47}O_3$ $[M+H]^+$ 443.3520, found: 443.3526.

(1S,6aS,6bR,8aR,10S,12aS,12bR)-2,2,6a,6b,9,9,12a-heptamethyl-1,2,3,5,6,6a,6b,7,8,8a,9,10,11,12,12a,12b-hexadecahydropicene-1,10-diol (**21**)

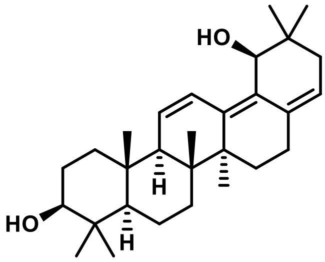

Chemical Formula: $C_{29}H_{44}O_2$
Exact Mass: 424,3341

In a round-bottom flask was charged epoxyalcohol **20** (12 mg, 27 μmol, 1.0 eq.) and dissolved in non-stabilised chloroform (1 mL). After stirring at room temperature for 3 hours the solvent was evaporated and the crude product purified via column chromatography (0.8 g SiO_2) using pentane/EtOAc = 5/1 to give 10 mg (87%) of triene **21** as a white foam.

^{1}H NMR (600 MHz, $CDCl_3$) δ = 6.67 (dd, *J* = 10.5, 3.2 Hz, 1H), 5.81 (dd, *J* = 10.5, 2.2 Hz, 1H), 5.45 (dd, *J* = 5.7, 3.3 Hz, 1H), 4.22 (s, 1H), 3.25 (dd, *J* = 11.5, 4.8 Hz, 1H), 2.54 – 2.39 (m, 1H), 2.34 – 2.26 (m, 2H), 2.03 (s, 1H), 1.92 (dt, *J* = 13.0, 3.6 Hz, 1H), 1.75 – 1.59 (m, 4H), 1.58 – 1.50 (m, 2H), 1.45 – 1.33 (m, 3H), 1.26 (dtd, *J* = 7.1, 4.9, 2.2 Hz, 2H), 1.21 (s, 1H), 1.07 (s, 3H), 1.00 (s, 3H), 0.98 (s, 3H), 0.93 (s, 3H), 0.84 – 0.80 (m, 1H), 0.78 (s, 6H), 0.70 (s, 3H).

^{13}C NMR (151 MHz, $CDCl_3$) δ = 140.3, 131.8, 130.8, 130.2, 124.4, 122.4, 79.1, 72.3, 54.9, 54.2, 42.2, 41.3, 39.0, 38.2, 36.9, 34.2, 32.7, 28.0, 27.2, 27.0, 26.7, 26.6, 24.6, 24.0, 19.1, 18.32, 18.26, 16.7, 15.2.

HR-MS (ESI): Calculated for $C_{29}H_{44}O_2Na$ $[M+Na]^+$ 447.3234, found: 447.3209.

(3S,4aR,6aR,6bS,8aS,12aR,14aR,14bR)-4,4,6a,6b,11,11,14b-heptamethyl-1,2,3,4,4a,5,6,6a,6b,7,8,9,10,11,12,14,14a,14b-octadecahydropicene-3,8a,12a-triol (**4**)

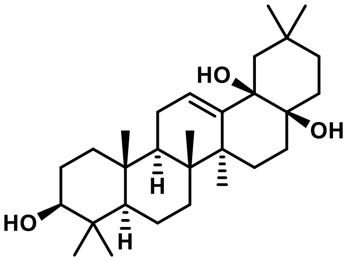

Chemical Formula: $C_{29}H_{48}O_3$
Exact Mass: 444,3603

In a 100-mL round-bottom flask was charged diene **4** (240 mg, 0.58 mmol, 1.0 eq.) and dissolved in a mixture of Et_2O (8 mL) and pyridine (0.8 mL). At room temperature a stock solution of OsO_4 (2.5% in *tert*-BuOH, 7.82 mL, 149 mg, 0.58 mmol, 1.0 eq.) was added and the solution turned dark brown over the next 15 minutes. After stirring for 3 hours a freshly prepared aqueous solution of sodium metabisulfite ($Na_2S_2O_5$) was introduced and stirring continued for 1 hour. Extraction with CH_2Cl_2 (3x 30 mL) and drying over Na_2SO_4 provided the crude product which was purified by column chromatography (12 g SiO_2) using pentane/EtOAc = 4/1 to give 12 mg (5%) of recovered starting material **4**, 58 mg (22%) of pure diol **10** and 320 mg of the corresponding osmate ester. The osmate ester was dissolved in THF/methanol = 1/1 and treated once again with sodium metabisulfite solution; upon extraction as before additional 181 mg (70%) of diol **10** were recovered as an off-white solid.

m.p.: 235 °C

Optical rotation: $[\alpha]_D^{20}$= +93.1 (c 1.25, CH_2Cl_2)

^{1}H NMR (600 MHz, $CDCl_3$) δ = 5.90 (t, *J* = 3.8, 1H), 3.22 (dd, *J* = 11.4, 4.5, 1H), 2.13 – 2.08 (m, 2H), 2.04 (ddt, *J* = 17.3, 14.1, 3.6, 1H), 1.97 – 1.92 (m, 2H), 1.69 (d, *J* = 15.1, 1H), 1.64 – 1.54 (m, 5H), 1.47 – 1.33 (m, 7H), 1.29 (dt, *J* = 12.8, 3.3, 1H), 1.13 (s, 3H), 1.12 (s, 3H), 0.99 (s, 3H), 0.97 (m, 2H) 0.91 (s, 3H), 0.88 (s, 3H), 0.87 (s, 3H), 0.79 (s, 3H), 0.77 – 0.73 (m, 1H).

^{13}C NMR (151 MHz, $CDCl_3$) δ = 149.8, 122.5, 79.2, 76.0, 73.3, 55.3, 51.8, 47.2, 42.9, 39.9, 38.9, 38.3, 37.2, 36.6, 34.2, 34.0, 32.8, 30.9, 29.0, 28.3, 27.7, 27.3, 25.3, 24.7, 23.7, 18.6, 17.4, 15.7, 15.2.

HR-MS (ESI): Calculated for $C_{29}H_{48}O_3Na$ $[M+Na]^+$ 467.3495, found: 467.3497.

(2S,4aR,4bR,14aS,14bR,16aR)-2-hydroxy-1,1,4a,9,9,14a,14b-heptamethyl-1,2,3,4,4a,4b,5,8,9,10,11,13,14,14a,14b,15,16,16a-octadecahydrocyclodeca[a]phenanthrene-7,12-dione (**5**)

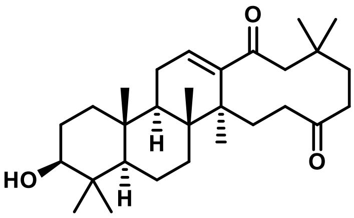

Chemical Formula: $C_{29}H_{46}O_3$
Exact Mass: 442,3447

In a 50-mL round-bottom flask diol **10** (120 mg, 0.27 mmol, 1 eq.) was dissolved in benzene (15 mL). Lead tetraacetate (131 mg, 0.30 mmol, 1.1 eq.) was added and the mixture stirred for 10 minutes at room temperature. The reaction mixture was then quenched with sat. $NaHCO_3$ solution and extracted with EtOAc (2x). The combined organic layers were dried over Na_2SO_4, filtered, and evaporated. The crude product was purified by column chromatography (10 g SiO_2) using pentane/EtOAc = 5/1 to give 112 mg (95%) of diketone **5** as a colourless solid.

m.p.: 224 – 225 °C

Optical rotation: $[\alpha]_D^{20}$= +4.4 (c 1.0, CH_2Cl_2)

^{1}H NMR (600 MHz, $CDCl_3$) δ = 5.88 (t, *J* = 3.9, 1H), 3.22 (dd, *J* = 11.4, 4.8 Hz, 1H), 2.71 – 2.58 (m, 1H), 2.54 (ddd, *J* = 17.7, 7.2, 4.9, 1H), 2.41 – 2.31 (m, 4H), 2.15 (ddd, *J* = 17.8, 8.5, 4.4, 1H), 2.03 – 1.99 (m, 1H), 1.97 (dd, *J* = 8.6, 3.8, 2H), 1.84 (ddd, *J* = 15.1, 8.5, 4.9, 1H), 1.69 – 1.64 (m, 1H), 1.64 – 1.47 (m, 7H), 1.40 – 1.35 (m, 1H), 1.26 (s, 3H), 1.21 (s, 3H), 0.99 (s, 3H), 0.98 (m, 1H), 0.95 (s, 3H), 0.95 (s, 3H), 0.89 (s, 3H), 0.79 (s, 3H), 0.73 (dd, *J* = 11.8, 1.9, 1H).

^{13}C NMR (151 MHz, $CDCl_3$) δ = 215.8, 208.2, 147.5, 129.1, 78.9, 55.3, 50.6, 47.6, 47.5, 40.3, 40.2, 38.90, 38.88, 37.1, 35.1, 33.6, 33.4, 30.3, 29.9, 29.6, 28.2, 27.5, 27.3, 23.6, 22.8, 18.3, 17.7, 16.2, 15.8.

HR-MS (ESI): Calculated for $C_{29}H_{46}O_3Na$ $[M+Na]^+$ 465.3339, found: 465.3345.

The reaction could also be carried out using $NaIO_4$ (THF, H_2O, rt) to give the diketone **5** in a slightly diminished yield of 80%.

Transannular Aldol reaction of 10-membered diketone **5**

Transannular aldol reaction scheme:

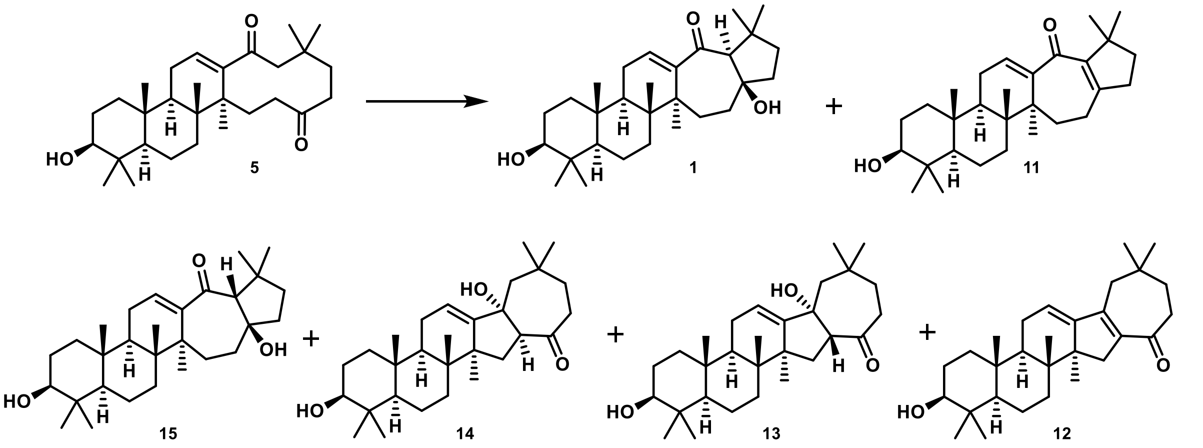

Scheme S3: Transannular aldol reactions towards products **1**, **11**-**15**

Entry	Reaction conditions	1	11	12	13	14	15
1	Pyrrolidine, AcOH, $CDCl_3$, rt, 18 h			68	20	11	
2	NaOH, EtOH, rt, 15 min		3	57	40		
3	NaOH, EtOH, rt, 20 h			98	2		
4	TFA, CD_2Cl_2, rt, 18 h			100			
5	KHMDS, THF, -78 °C		1	2	78	19	
6	TBD, C_6D_6, rt, 10 min				73	26	
7	DIPEA, toluene, 140 °C, 2.5 h			30	70		
8	Piperidine, toluene, 140 °C			6	83	11	
9	DABCO, toluene, 140 °C (partial conv.)				99		
10	KOH, EtOH, -30 °C, 40 min				60	40	
11	LiOH, 0 °C, 5 min			3	56	41	
12	NaOH, EtOH, 50 °C, 2 min		3	58	39		
13	NaOH, EtOH, 50 °C, 20 min		4	78	19		
14	DBU, CD_2Cl_2, 5 h	1			45	54	
15	DBU, toluene, 100 °C, 30 min	4		23	50	22	
16	DBU, toluene, 100 °C, 6 h			95			
17	DBU, toluene, 60 °C, 10 min	3			50	42	4
18	DBU, DCE, 65 °C, 5 min	5			58	31	6
19	DBN, toluene, 60 °C, 5 min	4			71	20	5
20	DBN, DCE, 60 °C, 60 min	10			73	16	
21	$TiCl_4$, $^{n}Bu_3N$, CH_2Cl_2			99			

Alstoscholarinoid A, (2aR,4S,6aR,6bR,9aS,12aS,14aS,14bR)-4,12a-dihydroxy-3,3,6a,10,10,14a,14b-heptamethyl-1,2a,3,4,5,6,6a,6b,7,9a,10,11,12,12a,13,14,14a,14b-octadecahydroazuleno[6,5-a]phenanthren-9(2H)-one (1)

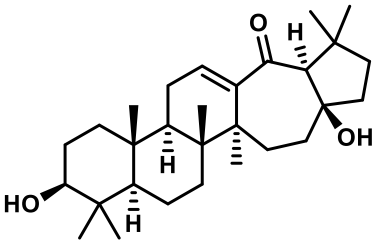

Chemical Formula: $C_{29}H_{46}O_3$
Exact Mass: 442,3447

In a microwave vial was charged diketone **5** (20 mg, 45 mmol, 1.0 eq.), dissolved in dry dichloroethane (1.5 mL) and heated to 60 °C. In a separate flask DBN (0.27 mL, 2.3 mmol, 50 eq.) was pre-heated and then added to the mixture in one portion. After 60 minutes the reaction mixture was poured onto ice/NH_4Cl solution and extracted twice with dichloromethane (20 mL). The pooled organic extracts were dried over $MgSO_4$ and evaporated. The crude product was purified by column chromatography (3 g fine silica) using hexanes/Et_2O = 2/1 to give 2 mg (90% purity, 9%) of Alstoscholarinoid A (**1**) as a white solid.

m.p.: 241 -242 °C.

Optical rotation: $[\alpha]_D^{20}$= +20.7 (c 0.4, MeOH); Lit[8]: +20 (c 0.11, MeOH).

^{1}H NMR (600 MHz, $CDCl_3$) δ = 6.60 (t, *J* = 4.0, 1H), 3.23 (dd, *J* = 11.4, 4.6, 1H), 2.87 (s, 1H), 2.15 (ddd, *J* = 20.2, 6.1, 4.5, 1H), 2.05 – 1.85 (m, 5H), 1.83 – 1.74 (m, 3H), 1.73 – 1.53 (m, 7H), 1.53 – 1.45 (m, 1H), 1.43 (s, 3H), 1.42 – 1.35 (m, 2H), 1.33 (s, 3H), 1.01 (s, 3H), 1.00 (s, 3H), 0.98 (m, 1H), 0.93 (s, 3H), 0.82 (s, 3H), 0.79 (s, 3H), 0.76 (d, *J* = 11.6, 1H).

^{13}C NMR (151 MHz, $CDCl_3$) δ = 202.9, 147.3, 135.8, 81.9, 79.0, 68.0, 55.3, 47.1, 43.9, 41.9, 41.8, 41.6, 41.1, 39.0, 38.9, 38.8, 37.3, 33.3, 29.6, 28.2, 27.3, 26.6, 24.3, 24.0, 22.5, 18.7, 16.3, 15.8, 15.7.

HR-MS (ESI): Calculated for $C_{29}H_{46}O_3Na$ $[M+Na]^+$ 465.3339, found: 465.3344.

NMR comparison with original isolation report [5]

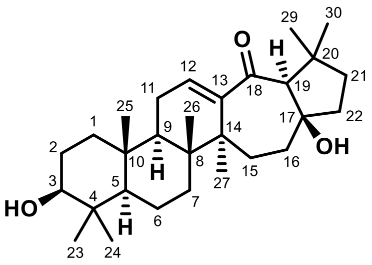

	Natural **1**[5]	Synthetic **1** - this work	$\Delta\delta_H$
Atom	δ_H [ppm (Hz)]	δ_H [ppm (Hz)]	[ppm]
**1**	1.67, 0.97	1.67, 0.98	+0.01
**2**	1.64	1.63	-0.01
**3**	3.24 (11.0, 4.8)	3.23 (11.4, 4.6)	-0.01
**5**	0.76 (11.6)	0.76 (11.6)	0
**6**	1.58, 1.42	1.59, 1.42	+0.01
**7**	1.60, 1.45	1.61, 1.45	+0.01
**9**	1.58	1.58	0
**11**	2.16, 2.01	2.15, 1.99	-0.02
**12**	6.61	6.60 (4.0)	-0.01
**15**	1.79, 1.48	1.78, 1.48	-0.01
**16**	1.93, 1.89	1.94, 1.90	+0.01
**19**	2.88	2.87	-0.01
**21**	1.58, 1.39	1.58, 1.39	0
**22**	1.96, 1.93	1.98, 1.92	+0.02
**23**	1.007	1.00	0
**24**	0.79	0.79	0
**25**	0.94	0.93	-0.01
**26**	0.83	0.82	-0.01
**27**	1.33	1.33	0
**29**	1.44	1.43	-0.01
**30**	1.015	1.01	0

NMR comparison with original isolation report by Luo et al. [5]

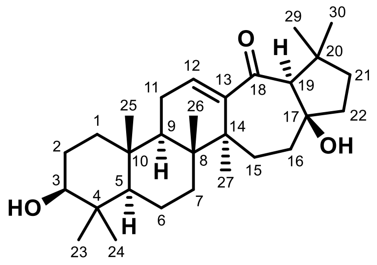

Atom	Natural **1**[5] δ_C [ppm]	Synthetic **1** - this work δ_C [ppm]	$\Delta\delta_C$ [ppm]
**1**	38.7	38.9	+0.02
**2**	27.2	27.3	+0.01
**3**	78.9	79.0	+0.01
**4**	38.7	38.8	+0.01
**5**	55.2	55.3	+0.01
**6**	18.5	18.7	+0.02
**7**	33.2	33.3	+0.01
**8**	41.0	41.1	+0.01
**9**	47.0	47.1	+0.01
**10**	37.2	37.3	+0.01
**11**	23.9	24.0	+0.01
**12**	135.7	135.8	+0.01
**13**	147.2	147.3	+0.01
**14**	41.7	41.8	+0.01
**15**	26.5	26.6	+0.01
**16**	38.8	39.0	+0.02
**17**	81.8	81.9	+0.01
**18**	202.8	202.9	+0.01
**19**	67.9	68.0	+0.01
**20**	43.7	43.9	+0.02
**21**	41.5	41.6	+0.01
**22**	41.8	41.9	+0.01
**23**	28.1	28.2	+0.01
**24**	15.6	15.7	+0.01
**25**	15.7	15.8	+0.01
**26**	16.2	16.3	+0.01
**27**	22.3	22.5	+0.02
**29**	24.2	24.3	+0.01
**30**	29.5	29.6	+0.01

^{1}H-NMR from natural source reproduced from [8] Copyright © 2021 American Chemical Society.

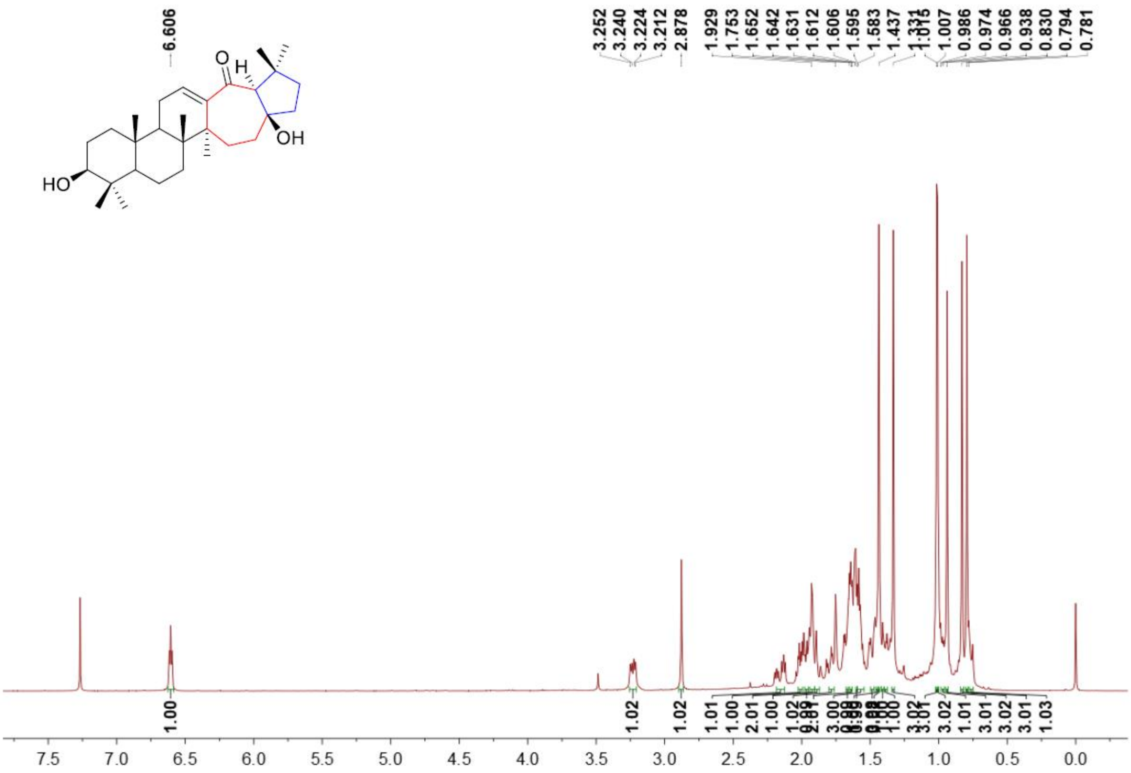

^{1}H-NMR of synthetic material:

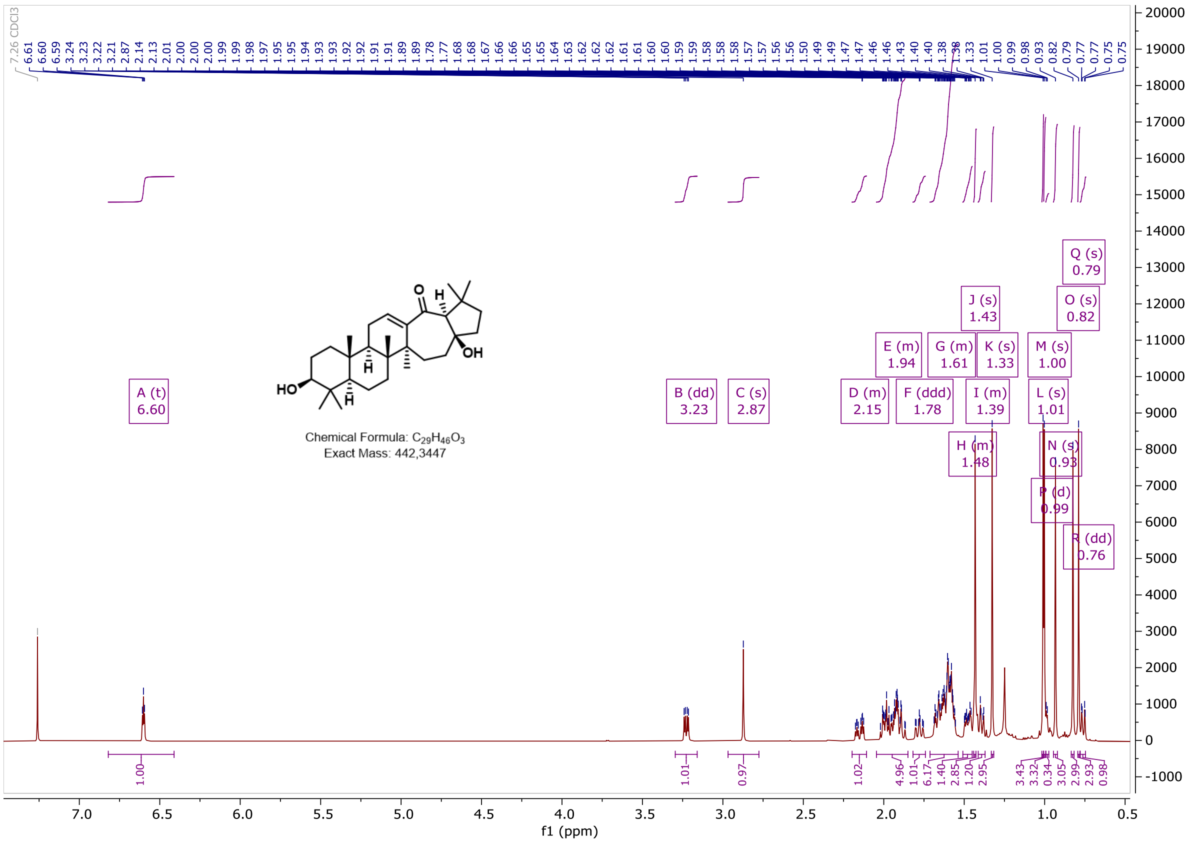

(2aR,4S,6aR,6bR,14aS,14bR)-4-hydroxy-3,3,6a,10,10,14a,14b-heptamethyl-1,2a,3,4,5,6,6a,6b,7,10,11,12,13,14,14a,14b-hexadecahydroazuleno[6,5-a]phenanthren-9(2H)-one (**12**)

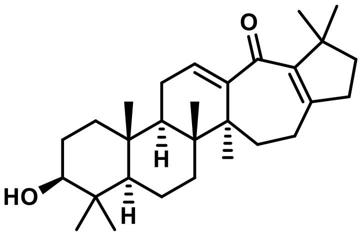

Chemical Formula: $C_{29}H_{44}O_2$
Exact Mass: 424,3341

From **1**: Alstoscholarinoid A (5 mg, 11 µmol, 1.0 eq.) was charged in a 10-mL round-bottom flask and dissolved in dichloromethane (1 mL). TFA (5 µL, 60 µmol 5.0 eq.) was added and the reaction mixture stirred at room temperature for 10 hours. After evaporation to dryness the residue was purified by column chromatography using pentane/EtOAc = 4/1 to give 4 mg (80%) of dienone **12** as a white solid.

From **5**: In a round-bottom flask was charged diketone **5** (16 mg, 36 µmol, 1.0 eq.) and dissolved in ethanol (3 mL). To this, a 1M solution of sodium hydroxide (0.18 mL, 0.18 mmol, 5.0 eq.) was added and the mixture allowed to stir for exactly 30 minutes. After this time the reaction was quenched by addition of excess aqueous NH_4Cl solution and extraction with EtOAc (3x). The crude product was purified using column chromatography (4 g fine SiO_2) using pentane/EtOAc = 5/1 as eluent to give a fraction containing the title compound which was purified once again using preparative TLC with the same eluent to give 0.5 mg (3%) of **12** as colourless oil.

m.p.: 223 – 225 °C
Optical rotation: $[\alpha]_D^{20}$= +28.6 (c 0.4, CH_2Cl_2)
^{1}H NMR (600 MHz, $CDCl_3$) δ = 6.62 (t, *J* = 3.9 Hz, 1H), 3.23 (dd, *J* = 11.4, 4.6 Hz, 1H), 2.51 – 2.39 (m, 1H), 2.39 – 2.28 (m, 2H), 2.24 (ddd, *J* = 17.3, 8.2, 2.7 Hz, 1H), 2.17 (dt, 1H), 2.07 – 1.96 (m, 1H), 1.71 – 1.54 (m, 10H), 1.46 – 1.39 (m, 2H), 1.28 (s, 3H), 1.16 (s, 3H), 1.09 (s, 3H), 1.01 (m, 1H), 1.00 (s, 3H), 0.94 (s, 3H), 0.83 (s, 3H), 0.79 (s, 3H), 0.78 – 0.75 (m, 1H).
^{13}C NMR (151 MHz, $CDCl_3$) δ = 194.3, 154.3, 148.5, 145.5, 134.7, 79.0, 55.5, 49.5, 47.5, 41.9, 40.34, 40.26, 38.9, 38.7, 37.3, 36.2, 33.4, 30.3, 29.4, 28.2, 27.4, 27.0, 26.7, 24.1, 19.6, 18.6, 16.0, 15.9, 15.7.
HR-MS (ESI): Calculated for $C_{29}H_{45}O_2$ $[M+H]^+$ 425.3414, found: 425.3423.

(2aR,4S,6aR,6bR,8bR,13aS,14aS,14bR)-4,8b-dihydroxy-3,3,6a,10,10,14a,14b-heptamethyl-2,2a,3,4,5,6,6a,6b,7,8b,9,10,11,12,13a,14,14a,14b-octadecahydroazuleno[2,1-a]phenanthren-13(1H)-one (**14**)

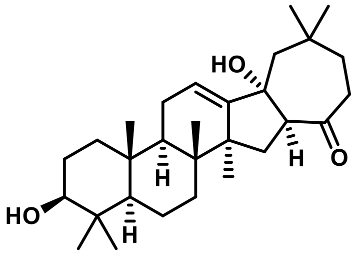

Chemical Formula: $C_{29}H_{46}O_3$
Exact Mass: 442,3447

In an NMR tube was charged diketone **5** (3 mg, 7 µmol, 1.0 eq.) and dissolved in CD_3CN (0.5 mL). DBN (2.5 µL, 20 µmol, 3.0 eq.) was added via syringe and thoroughly mixed. Proton NMR spectra were then recorded until full conversion was obtained (20 min) and the mixture was subsequently quenched with saturated NH_4Cl solution and extracted with EtOAc. After evaporation of the solvent, the crude product was purified via column chromatography (1 g SiO_2) using 1:1 pentane:Et_2O to give 1.9 mg (63%) of **14** as a white solid.

m.p.: 203 °C

Optical rotation: $[\alpha]_D^{20}$= +35.8 (c 0.3, $CHCl_3$)

^{1}H NMR (600 MHz, CD_2Cl_2) δ = 5.60 (t, *J* = 3.7, 1H), 3.42 – 3.35 (m, 1H), 3.28 – 3.13 (m, 1H), 2.63 (ddd, *J* = 15.2, 6.6, 3.6, 1H), 2.46 (ddd, *J* = 15.2, 12.1, 3.3, 1H), 2.37 (ddd, *J* = 14.1, 12.1, 3.7, 1H), 2.28 (ddd, *J* = 13.4, 9.8, 1.2, 1H), 2.05 – 1.95 (m, 1H), 1.93 – 1.83 (m, 1H), 1.79 (d, *J* = 15.5, 1H), 1.70 – 1.61 (m, 3H), 1.61 – 1.52 (m, 9H), 1.49 – 1.42 (m, 1H), 1.41 – 1.37 (m, 2H), 1.37 – 1.31 (m, 2H), 1.28 (s, 3H), 1.23 (s, 3H), 0.98 (s, 3H), 0.88 (s, 3H), 0.84 – 0.79 (m, 1H), 0.78 (s, 3H), 0.78 (s, 2x 3H).

^{13}C NMR (151 MHz, CD_2Cl_2) δ = 212.1, 153.9, 118.6, 81.8, 79.1, 62.1, 56.1, 48.9, 48.2, 47.6, 40.6, 39.17, 39.12, 38.6, 37.5, 34.79, 34.76, 33.8, 31.75, 31.73, 31.6, 28.3, 27.7, 25.6, 23.6, 18.6, 17.2, 15.87, 15.79.

HR-MS (ESI): Calculated for $C_{29}H_{46}O_3Na$ $[M+Na]^+$ 465.3339, found: 465.3334.

NOESY correlations:

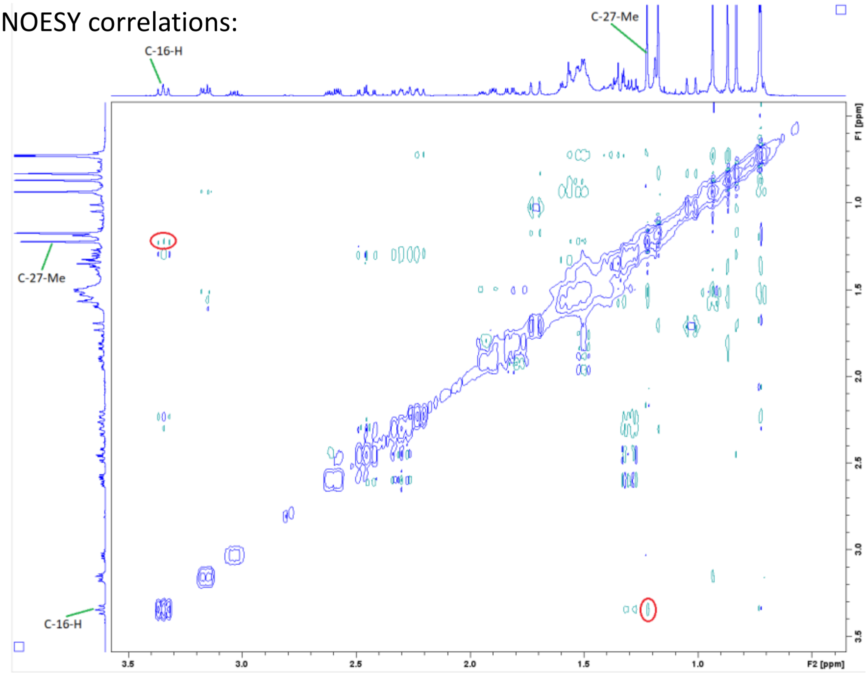

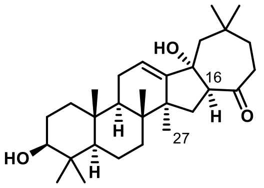

Chemical Formula: $C_{29}H_{46}O_3$
Exact Mass: 442,3447

(2aR,4S,6aR,6bR,8bR,13aR,14aS,14bR)-4,8b-dihydroxy-3,3,6a,10,10,14a,14b-heptamethyl-2,2a,3,4,5,6,6a,6b,7,8b,9,10,11,12,13a,14,14a,14b-octadecahydroazuleno[2,1-a]phenanthren-13(1H)-one (**13**)

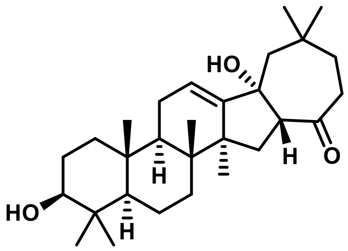

Chemical Formula: $C_{29}H_{46}O_3$
Exact Mass: 442,3447

In an NMR tube was charged diketone **5** (5 mg, 11 µmol, 1.0 eq.) and dissolved in DMSO-d6 (0.6 mL). Then, L-proline (0.5 mg, 5 µmol, 0.4 eq.) was added and the solution thoroughly mixed to dissolve. The mixture was warmed to 60 °C for 24h and subsequently quenched with saturated NH_4Cl solution and extracted with EtOAc. After evaporation of the solvent, the crude product was crystallized from heptane to give 4.5 mg (90%) of the product **13** as a white solid. A single crystal for X-ray diffraction analysis was grown from a mixture of dichloromethane and toluene/acetonitrile by slow evaporation.

m.p.: 235 °C

Optical rotation: $[\alpha]_D^{20}$= -27.0 (c 0.2, CH_2Cl_2)

^{1}H NMR (600 MHz, CD_2Cl_2) δ = 5.56 (dd, *J* = 4.5, 3.0, 1H), 3.23 – 3.10 (m, 1H), 2.67 – 2.56 (m, 1H), 2.45 – 2.33 (m, 1H), 2.10 – 1.96 (m, 3H), 1.95 – 1.82 (m, 2H), 1.77 (dd, *J* = 14.4, 9.1, 1H), 1.69 – 1.35 (m, 9H), 1.32 – 1.28 (m, 1H), 1.27 (s, 3H), 1.10 (s, 3H), 1.02 (s, 3H), 0.97 (s, 3H), 0.81 (dd, *J* = 11.9, 2.1, 1H), 0.77 (s, 3H), 0.65 (s, 3H).

^{13}C NMR (151 MHz, CD_2Cl_2) δ = 211.0, 153.2, 117.9, 81.6, 79.0, 60.1, 56.1, 51.2, 48.0, 47.3, 39.8, 39.2, 38.7, 38.4, 37.6, 35.9, 35.8, 33.7, 33.6, 32.7, 28.2, 27.8, 27.6, 25.6, 23.4, 18.7, 16.6, 15.7, 15.6.

HR-MS (ESI): Calculated for $C_{29}H_{46}O_3Na$ $[M+Na]^+$ 465.3339, found: 465.3346.

X-ray: **13** was crystallized as the mono-toluene solvate (CSD 2412493):

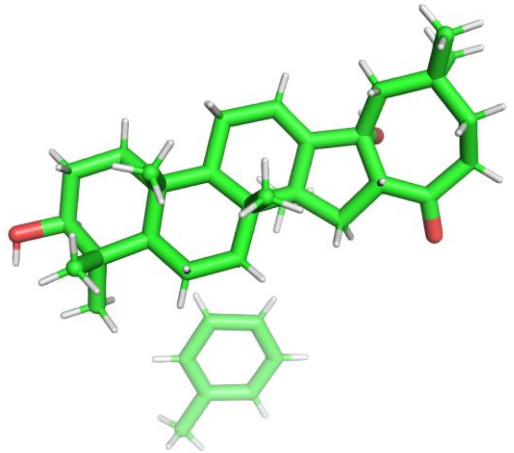

(2aR,4S,6aR,6bR,14aS,14bR)-4-hydroxy-3,3,6a,10,10,14a,14b-heptamethyl-2,2a,3,4,5,6,6a,6b,7,9,10,11,12,14,14a,14b-hexadecahydroazuleno[2,1-a]phenanthren-13(1H)-one (**11**)

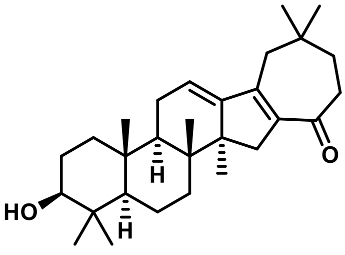

In an NMR tube was charged diketone **5** (5 mg, 11 µmol, 1.0 eq.) in $CDCl_3$ (0.5 mL) and treated with trifluoroacetic acid (2.5 µL, 34 µmol, 3.0 eq.) at room temperature for 24 hours. The reaction mixture was then evaporated to dryness and purified by column chromatography (1 g SiO_2) using pentane/EtOAc = 5/1 to give 4.2 mg (84%) of dienone **11** as a colourless solid.

m.p.: 217 °C

Optical rotation: $[\alpha]_D^{20}$= +9.7 (c 0.3, CH_2Cl_2)

^{1}H NMR (600 MHz, $CDCl_3$) δ = 5.88 (t, *J* = 4.0, 1H), 3.24 (dd, *J* = 11.4, 4.4, 1H), 2.70 (d, *J* = 16.7, 1H), 2.63 – 2.43 (m, 2H), 2.38 – 2.16 (m, 3H), 2.09 (d, *J* = 16.7, 1H), 2.07 – 1.98 (m, 1H), 1.81 – 1.52 (m, 8H), 1.51 – 1.34 (m, 2H), 1.06 (s, 3H), 1.04 (m, 1H), 1.01 (s, 3H), 0.99 (s, 3H), 0.98 (s, 3H), 0.92 (s, 3H), 0.84 – 0.81 (m, 1H), 0.80 (s, 3H), 0.69 (s, 3H).

^{13}C NMR (151 MHz, $CDCl_3$) δ = 203.4, 154.2, 151.6, 139.9, 121.8, 79.0, 56.0, 48.6, 48.3, 39.6, 39.0, 38.7, 38.6, 37.44, 37.42, 37.36, 35.7, 34.3, 33.1, 30.2, 30.1, 28.2, 27.3, 25.3, 24.4, 18.3, 17.6, 15.7, 15.5.

HR-MS (ESI): Calculated for $C_{29}H_{45}O_2$ $[M+H]^+$ 425.3414, found: 425.3421.

(3aR,6aS,6bR,8aR,10S,12aR,12bR,14bS)-10,14b-dihydroxy-2,2,6a,6b,9,9,12a-heptamethyl-2,3,3a,5,6,6a,6b,7,8,8a,9,10,11,12,12a,12b,13,14b-octadecahydroazuleno[5,4-a]phenanthren-4(1H)-one (**15**)

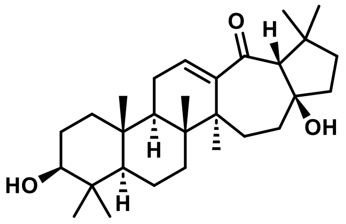

Chemical Formula: $C_{29}H_{44}O_2$
Exact Mass: 424,3341

In a microwave vial was charged diketone **5** (22 mg, 49 mmol, 1.0 eq.), dissolved in dry toluene (1.2 mL) and heated to 60 °C. In a separate flask DBU (50 eq.) was pre-heated and then added to the mixture in one portion. After 200 seconds the reaction mixture was poured onto ice/NH_4Cl solution and extracted twice with dichloromethane (10 mL). The pooled organic extracts were dried over $MgSO_4$ and evaporated. The crude product was purified by column chromatography (2.9 g fine silica) using hexanes/Et_2O = 2/1 to give a fraction of 4 mg, containing 22% (by ^{1}H-NMR) of compound **15**, together with aldol adduct **13**. This fraction was further purified by column chromatography over 15-25 µm silica gel (1 g) using pentane/CH_2Cl_2/acetone = 10/10/1 to give 0.7 mg (3%) of aldol product **15** as a colourless oil.

^{1}H NMR (600 MHz, $CDCl_3$) δ = 5.81 (dd, *J* = 4.7, 3.1, 1H), 3.22 (dd, *J* = 11.5, 4.7, 1H), 2.90 (s, 1H), 2.04 – 1.95 (m, 2H), 1.95 – 1.88 (m, 2H), 1.88 – 1.81 (m, 2H), 1.80 – 1.72 (m, 2H), 1.69 – 1.58 (m, 3H), 1.51 (m, 4H), 1.48 – 1.40 (m, 1H), 1.39 (s, 3H), 1.34 – 1.28 (m, 1H), 1.11 (s, 3H), 1.08 (s, 3H), 0.99 (s, 2x 3H), 0.99 – 0.97 (m, 1H), 0.95 (s, 3H), 0.79 (s, 3H), 0.75 (d, *J* = 11.1, 1H).
^{13}C NMR (151 MHz, $CDCl_3$) δ = 207.7, 150.6, 126.1, 82.7, 78.9, 68.4, 55.3, 48.0, 43.3, 42.0, 41.5, 40.9, 39.9, 38.9, 38.8, 37.9, 37.2, 33.5, 32.2, 28.9, 28.2, 27.3, 27.2, 24.4, 22.8, 18.5, 17.8, 16.1, 15.7.
HR-MS (ESI): Calculated for $C_{29}H_{46}O_3Na$ $[M+Na]^+$ 465.3339, found: 465.3337.

Photochemical reactions of diene **4**

Oxidation of **4** to hydroperoxide and divergent synthesis of oxidised oleanes:

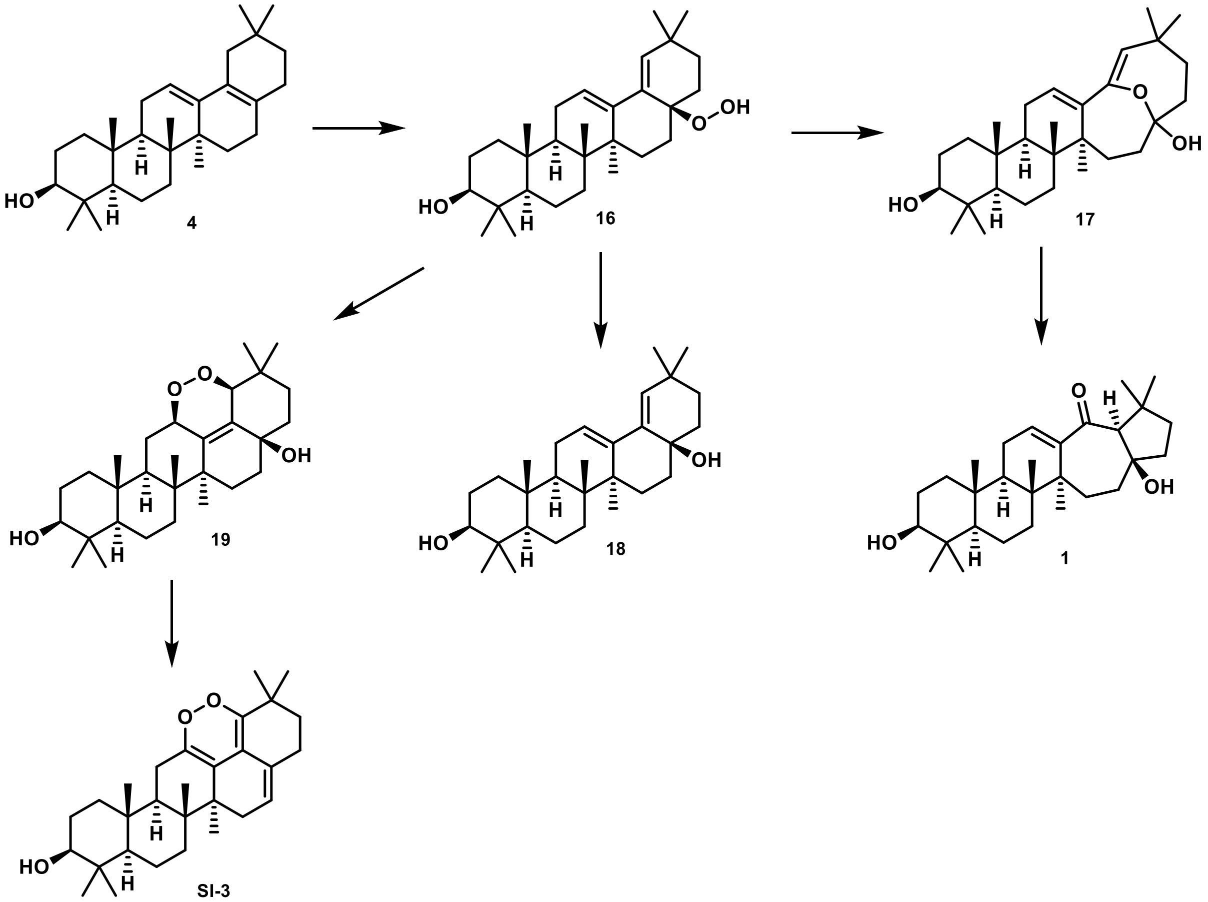

Scheme S4: Photochemical reactions towards Alstoscholarinoid A and exploration of intermediates

The first photocatalytic oxidation of alkene **4** generally forms **16** selectively, although in the presence of TPP as the photocatalyst significant conversion of this compound to doubly oxidized **19** (and its hydroperoxide) takes place as well. Under neutral conditions all of **4** is converted to **16** (using methylene blue) or **19** hydroperoxide (using TPP) after prolonged exposure to light and oxygen. Under acidic conditions (TFA or methanesulfonic acid) conversion of **16** to **17** and furthermore **1** is rapid and is typically complete within one hour. Peculiarly, compound **19** could be further transformed to cyclic endoperoxydiene **SI-3** by means of aqueous Fe(II). Reaction towards Alstoscholarinoid A was feasible using both methylene blue or TPP as photocatalyst (but not Rose Bengal) giving the product in yields of 40-50%.

(3S,4aR,6aR,6bS,8aS,14aR,14bR)-8a-hydroperoxy-4,4,6a,6b,11,11,14b-heptamethyl-1,2,3,4,4a,5,6,6a,6b,7,8,8a,9,10,11,14,14a,14b-octadecahydropicen-3-ol (**16**)

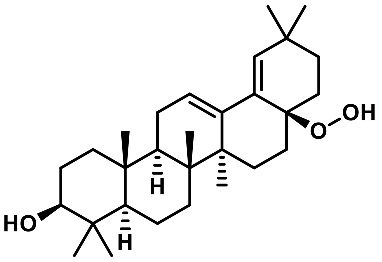

Chemical Formula: $C_{29}H_{46}O_3$
Exact Mass: 442,3447

In a 50-mL round bottom flask was charged diene **4** (102 mg, 0.25 mmol, 1.0 eq.) in dichloromethane/methanol = 20/1 (10 mL). Methylene blue (3.2 mg, 0.01 mmol, 4 mol%) was introduced and pure oxygen was bubbled slowly through the solution under 100W white LED irradiation. After 1.5 hours the reaction was concentrated and purified directly by column chromatography (8 g SiO_2) using pentane/EtOAc = 10/1 to 5/1 to give 6 mg of recovered diene **4** as well as 90 mg (82%, 87% brsm) of hydroperoxide **16** as white solids.

m.p.: 180 – 181 °C

Optical rotation: $[\alpha]_D^{20}$= +209.1 (c 0.68, CH_2Cl_2)

^{1}H NMR (600 MHz, $CDCl_3$) δ = 7.25 (s, 1H), 5.64 (d, *J* = 1.4 Hz, 1H), 5.52 (dd, *J* = 5.0, 2.7 Hz, 1H), 3.21 (dd, *J* = 11.2, 5.0 Hz, 1H), 2.15 (ddt, *J* = 14.4, 4.1, 3.0 Hz, 2H), 2.06 – 1.93 (m, 2H), 1.88 – 1.78 (m, 1H), 1.69 – 1.30 (m, 12H), 1.13 (dt, *J* = 13.0, 3.4 Hz, 1H), 1.00 (s, 3H), 0.99 (s, 9H), 0.98 (s, 3H), 0.96 (s, 3H), 0.79 (s, 3H), 0.73 (dd, *J* = 11.7, 1.8 Hz, 1H).

^{13}C NMR (151 MHz, $CDCl_3$) δ = 142.4, 140.0, 134.7, 121.1, 81.0, 79.1, 55.6, 47.7, 43.6, 38.98, 38.91 (2C), 37.1, 34.4, 33.1, 32.5, 30.5, 30.0, 28.8, 28.28, 27.47, 27.38, 25.7, 23.6, 22.6, 18.4, 17.6, 16.1, 15.8.

HR-MS (ESI): Calculated for $C_{29}H_{47}O_3$ $[M+H]^+$ 443.3520, found: 443.3523.

(2S,4aR,4bR,14aS,14bR,16aR)-1,1,4a,9,9,14a,14b-heptamethyl-1,3,4,4a,4b,5,9,10,11,13,14,14a,14b,15,16,16a-hexadecahydro-7,12-epoxycyclodeca[a]phenanthrene-2,12(2H)-diol (**17**)

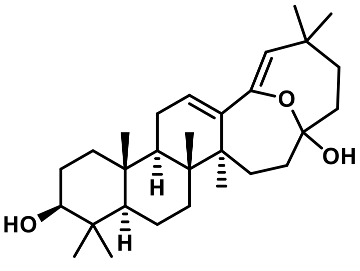

Chemical Formula: $C_{29}H_{46}O_3$
Exact Mass: 442,3447

In a 50-mL round-bottom flask was charged diene **4** (0.1 g, 0.24 mmol, 1.0 eq.) and dissolved in CH_2Cl_2 (15 mL) and chilled to -15 °C. Tetraphenylporphyrin (1.5 mg, 2.4 µmol, 1 mol%) was introduced, followed by O_2 bubbling through the solution. The flask was irradiated (30W white LEDs) and trifluoroacetic acid (27 µL, 0.36 mmol, 1.5 eq.) was introduced after 5 minutes. The reaction was run under these conditions for 45 minutes before evaporating to dryness to provide the crude product. This residue was purified via column chromatography (7 g SiO_2) using pentane/EtOAc = 5/1 as eluent to give 5 mg (5%) of **17** as a colourless oil. Additionally, 48 mg (45%) of Alstoscholarinoid A (**1**) were isolated from this run as a colorless solid.

^{1}H NMR (600 MHz, $CDCl_3$) δ = 5.45 (t, *J* = 3.6 Hz, 1H), 4.97 (s, 1H), 3.22 (dd, *J* = 11.4, 4.6 Hz, 1H), 2.71 (ddd, *J* = 14.2, 11.7, 4.4 Hz, 1H), 2.43 (dd, *J* = 16.2, 11.5 Hz, 1H), 2.23 (ddd, *J* = 14.3, 5.3, 3.7 Hz, 1H), 2.13 – 2.00 (m, 2H), 1.90 (dd, *J* = 8.7, 3.7 Hz, 2H), 1.75 – 1.61 (m, 3H), 1.59 – 1.50 (m, 6H), 1.45 – 1.39 (m, 2H), 1.29 (s, 3H), 1.24 – 1.20 (m, 1H), 1.04 (s, 3H), 1.02 (s, 3H), 1.00 (s, 3H), 0.99 – 0.97 (m, 1H), 0.95 (s, 3H), 0.94 (s, 3H), 0.79 (s, 3H), 0.78 – 0.73 (m, 1H).

^{13}C NMR (151 MHz, $CDCl_3$) δ = 150.3, 140.9, 128.0, 124.6, 111.7, 78.9, 55.3, 47.8, 43.5, 38.77, 38.75, 38.65, 37.2, 36.0, 33.93, 33.86, 33.4, 32.1, 29.4, 28.1, 27.2, 25.7, 25.4, 22.6, 22.0, 18.4, 16.7, 15.7, 15.6.

HR-MS (ESI): Calculated for $C_{29}H_{46}O_3Na$ $[M+Na]^+$ 465.3339, found: 465.3344.

Alstoscholarinoid A, (2aR,4S,6aR,6bR,9aS,12aS,14aS,14bR)-4,12a-dihydroxy-3,3,6a,10,10,14a,14b-heptamethyl-1,2a,3,4,5,6,6a,6b,7,9a,10,11,12,12a,13,14,14a,14b-octadecahydroazuleno[6,5-a]phenanthren-9(2H)-one (1)

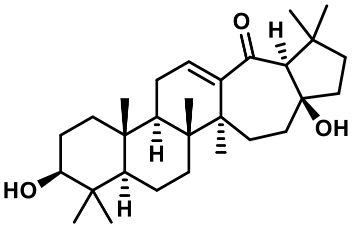

Chemical Formula: $C_{29}H_{46}O_3$
Exact Mass: 442,3447

In a 50-mL round-bottom flask was charged diene **4** (100 mg, 0.24 mmol, 1.0 eq.) and dissolved in CH_2Cl_2/MeCN = 20/1 (10 mL). Methylene blue (1.5 mg, 5 µmol, 2 mol%) was introduced, followed by trifluoroacetic acid (27 µL, 0.36 mmol, 1.5 eq.) and oxygen was bubbled through the solution. The flask was irradiated (30W white LEDs) for 45 minutes in the presence of oxygen at 15 °C. The reaction was then concentrated and the crude residue purified via column chromatography (7 g SiO_2) using pentane/EtOAc = 5/1 as eluent to give 53 mg (49%) of Alstoscholarinoid A (**1**) as a white solid.

m.p.: 241 - 242 °C.

Optical rotation: $[\alpha]_D^{20}$= +20.7 (c 0.4, MeOH); Lit[8]: +20 (c 0.11, MeOH).

^{1}H NMR (600 MHz, $CDCl_3$) δ = 6.60 (t, *J* = 4.0, 1H), 3.23 (dd, *J* = 11.4, 4.6, 1H), 2.87 (s, 1H), 2.15 (ddd, *J* = 20.2, 6.1, 4.5, 1H), 2.05 – 1.85 (m, 5H), 1.83 – 1.74 (m, 3H), 1.73 – 1.53 (m, 7H), 1.53 – 1.45 (m, 1H), 1.43 (s, 3H), 1.42 – 1.35 (m, 2H), 1.33 (s, 3H), 1.01 (s, 3H), 1.00 (s, 3H), 0.98 (m, 1H), 0.93 (s, 3H), 0.82 (s, 3H), 0.79 (s, 3H), 0.76 (d, *J* = 11.6, 1H).

^{13}C NMR (151 MHz, $CDCl_3$) δ = 202.9, 147.3, 135.8, 81.9, 79.0, 68.0, 55.3, 47.1, 43.9, 41.9, 41.8, 41.6, 41.1, 39.0, 38.9, 38.8, 37.3, 33.3, 29.6, 28.2, 27.3, 26.6, 24.3, 24.0, 22.5, 18.7, 16.3, 15.8, 15.7.

HR-MS (ESI): Calculated for $C_{29}H_{46}O_3Na$ $[M+Na]^+$ 465.3339, found: 465.3344.

(3aS,5aR,6aR,6bR,9S,10aR,12aR,12bS,14aR)-3,3,6b,10,10,12a,12b-heptamethyl-2,3,3a,5a,6,6a,6b,7,8,9,10,10a,11,12,12a,12b,13,14-octadecahydropiceno[1,14-cde][1,2]dioxine-9,14a(1H)-diol (**19**)

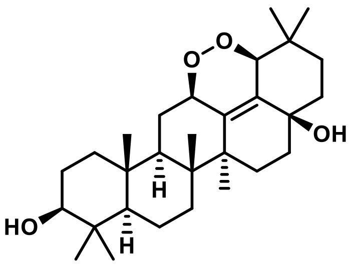

Chemical Formula: $C_{29}H_{46}O_4$
Exact Mass: 458,3396

In a 25-mL round-bottom flask was charged diene **4** (70 mg, 0.17 mmol, 1.0 eq.) and tetraphenylporphyrin (TPP, 2.1 mg, 3 μmol, 2 mol%). The solids were dissolved in 10 mL of dichloromethane and stirred under irradiation (18 W, white LEDs). Oxygen was bubbled through the solution for 2 hours, until TLC indicated complete consumption of diene **4** and hydroperoxide **16**. The reaction mixture was concentrated to 1/5 and redissolved in methanol (10 mL). Thiourea (51 mg, 0.68 mmol, 4.0 eq.) was then added and the mixture stirred for 1.5 hours at room temperature. Celite (0.5 g) was introduced and the solvent removed. The celite was applied to the top of a column (5 g SiO_2) using pentane/EtOAc = 5/1 as eluent system, followed by trituration with 0.7 mL of benzene to give 38 mg (48%) of **19** as a white solid.

m.p.: 188 – 190 °C

Optical rotation: $[\alpha]_D^{20}$= +59.8 (c 0.9, CH_2Cl_2)

^{1}H NMR (600 MHz, CD_2Cl_2) δ = 4.87 (ddd, *J* = 12.9, 8.6, 1.5 Hz, 1H), 4.29 (d, *J* = 1.5 Hz, 1H), 3.24 – 3.10 (m, 1H), 2.08 (td, *J* = 14.7, 4.7 Hz, 1H), 1.86 (ddd, *J* = 15.1, 4.6, 2.6 Hz, 1H), 1.76 (dddd, *J* = 8.4, 5.2 Hz, 2H), 1.74 – 1.67 (m, 2H), 1.66 – 1.53 (m, 7H), 1.53 – 1.44 (m, 1H), 1.40 – 1.20 (m, 6H), 1.08 (s, 3H), 1.00 (s, 6H), 0.99 (s, 3H), 0.96 (s, 3H), 0.93 (m, 1H), 0.91 (s, 3H), 0.80 (dd, *J* = 12.4, 2.5 Hz, 1H), 0.77 (s, 3H).

^{13}C NMR (151 MHz, CD_2Cl_2) δ = 139.0, 128.1, 85.7, 79.2, 75.9, 68.8, 56.2, 49.3, 42.4, 39.5, 39.3, 39.2, 39.0, 38.1, 36.9, 36.5, 35.31, 35.29, 28.5, 28.2, 27.6, 24.0, 21.3, 21.1, 21.0, 20.3, 18.8, 16.9, 15.5.

HR-MS (ESI): Calculated for $C_{29}H_{49}O_4$ $[M+H]^+$ 461.3626, found: 461.3629.

(6aR,6bR,9S,10aR,12aR,12bS)-3,3,6b,10,10,12a,12b-heptamethyl-1,2,3,6,6a,6b,7,8,9,10,10a,11,12,12a,12b,13-hexadecahydropiceno[1,14-cde][1,2]dioxin-9-ol (SI-3)

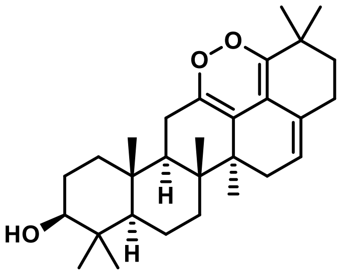

Chemical Formula: $C_{29}H_{42}O_3$
Exact Mass: 438,3134

In a 10-mL round bottom flask was charged starting material **19** (20 mg, 43 μmol, 1.0 eq.) in 6 mL of a mixture of methanol, tetrahydrofuran and water (1/1/1). At room temperature a solution of $FeSO_4$ heptahydrate (75 mg, 0.26 mmol, 6.0 eq.) was introduced and the mixture stirred at room temperature for 1 hour. The mixture was extracted with dichloromethane (3x 10 mL) and dried over $MgSO_4$. The crude product was purified by column chromatography (2 g SiO_2) using pentane/EtOAc = 5/1 to give 14 mg (73%) of peroxy compound **SI-3** as a white solid.

m.p.: 198 – 200 °C.

Optical rotation: $[\alpha]_D^{20}$= +67.0 (c 0.6, MeOH)

^{1}H NMR (600 MHz, CD_2Cl_2) δ = 5.16 (ddd, *J* = 5.4, 2.3, 1.1 Hz, 1H). 3.32 – 3.11 (m, 1H), 2.57 (dd, *J* = 16.2, 5.4 Hz, 1H), 2.51 (ddtt, *J* = 13.1, 11.6, 3.9, 1.7 Hz, 1H), 2.46 – 2.39 (m, 1H), 2.34 (dd, *J* = 16.2, 11.2 Hz, 1H), 2.28 (dt, *J* = 15.1, 4.5 Hz, 1H), 1.89 (dd, *J* = 11.1, 5.4 Hz, 1H), 1.84 (ddd, *J* = 16.6, 5.3, 1.6 Hz, 1H), 1.77 – 1.66 (m, 4H), 1.66 – 1.56 (m, 4H), 1.47 (ddd, *J* = 13.5, 5.3, 3.0 Hz, 1H), 1.24 (s, 3H), 1.23 (s, 3H), 1.13 (s, 3H), 1.11 – 1.07 (m, 1H), 1.04 (s, 3H), 1.00 (s, 3H), 0.87 – 0.84 (m, 1H), 0.83 (s, 3H), 0.79 (s, 3H).

^{13}C NMR (151 MHz, CD_2Cl_2) δ = 154.9, 145.9, 129.0, 121.4, 116.5, 115.1, 79.0, 56.3, 51.7, 42.6, 40.4, 39.8, 39.4, 38.7, 37.9, 34.9, 34.0, 33.5, 28.6, 27.9, 27.7, 27.1, 25.5, 24.2, 21.9, 18.7, 17.9, 16.8, 16.0.

HR-MS (ESI): Calculated for $C_{29}H_{42}O_3Na$ $[M+Na]^+$ 461.3026, found: 461.3032.

Unsuccessful routes towards Alstoscholarinoid B

Unsuccessful epoxyketone, Criegee rearrangement and Mitsunobu approaches

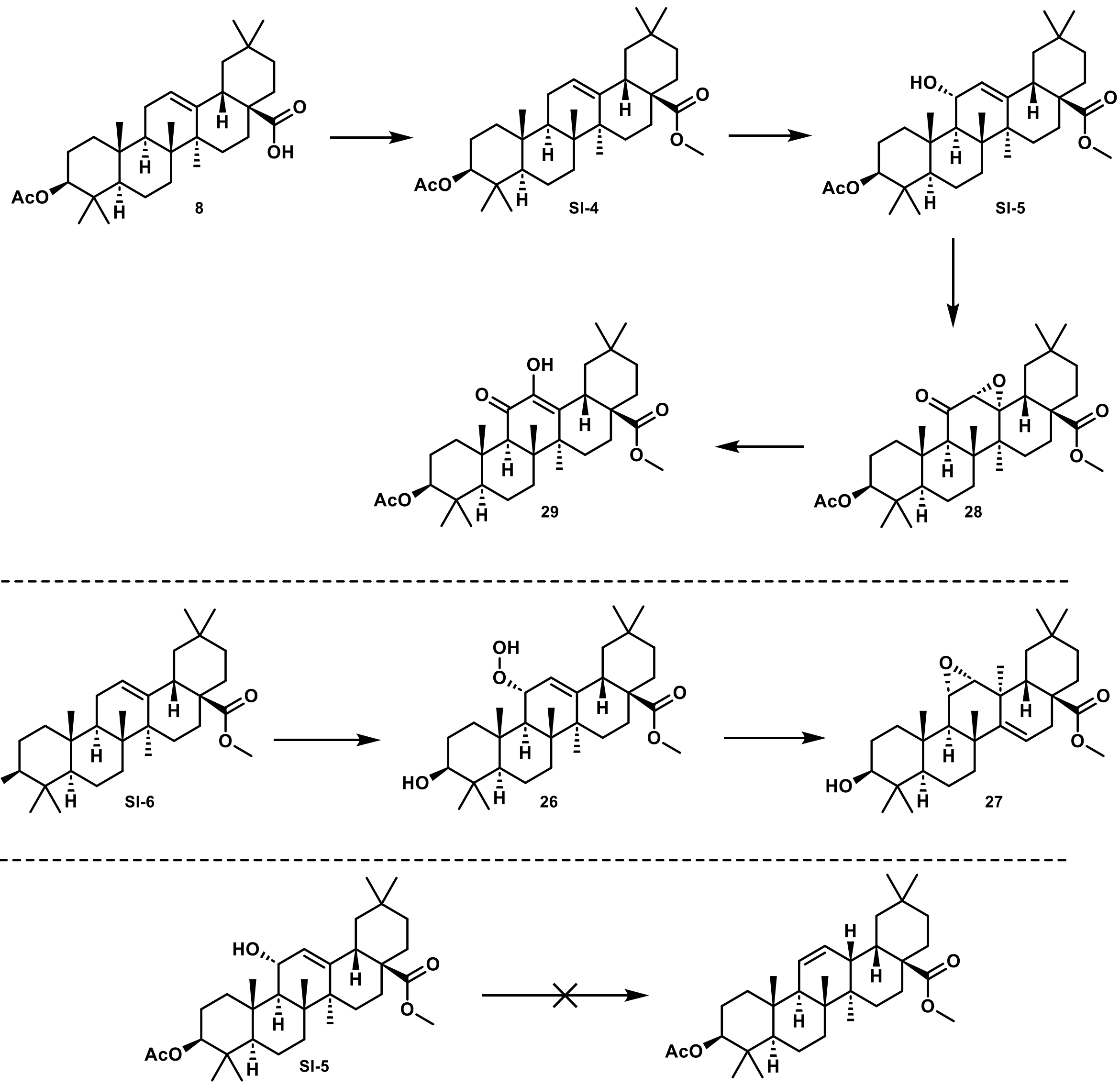

Scheme S5: Failed approaches towards Alstoscholarinoid B: a) ring contraction via epoxyketone rearrangement; b) Criegee rearrangement of secondary hydroperoxide; c) direct olefin transposition via Mitsunobu reaction

Epoxyketone **28** was accessed stereoselective allylic oxidation followed by epoxidation and DMP oxidation of the secondary alcohol. A range of acidic and photolytic conditions (CSA, $Bi(OTf)_3$, $MeSO_3H$, $TiCl_4$, *p*-TSA, 254 nm light) were tested on **28** revealing the H-migration to form **29** as the dominant pathway. Methyl oleanolate **SI-6** was used to access a-hydroperoxide **26**, which under the tested reaction conditions (Ac_2O, TFAA, TFA, TsNCO, $Sc(OTf)_3$, CSA, $Sn(OTf)_2$) delivered many different products with epoxide **27** being the most prominent. Mitsunobu replacement of **SI-5** was also evaluated (NH_2NHNs, DEAD/DIAD in NMM) and abandoned due to lack of reactivity.

Methyl (4aS,6aS,6bR,8aR,10S,12aR,12bR,14bS)-10-acetoxy-2,2,6a,6b,9,9,12a-heptamethyl-1,3,4,5,6,6a,6b,7,8,8a,9,10,11,12,12a,12b,13,14b-octadecahydropicene-4a(2H)-carboxylate (SI-4)

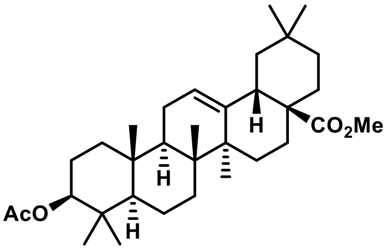

Chemical Formula: $C_{33}H_{52}O_4$
Exact Mass: 512,3866

In a 100-mL round-bottom flask was charged acetyl oleanolic acid **8** (1.2 g, 2.4 mmol, 1.0 eq.) and dissolved in acetone (40 mL). Potassium carbonate (1.33 g, 9.62 mmol, 4.0 eq.) was introduced followed by methyl iodide (0.3 mL, 4.8 mmol, 2.0 eq.) and the mixture was heated to 35 °C for 24 hours. The reaction was then concentrated to ¼ volume and taken up with 1M aq. HCl and dichloromethane and extracted. Pooled dichloromethane extracts were washed with saturated aq. $NaHCO_3$ solution and dried over $MgSO_4$. The crude product was purified by flash chromatography using pentane/EtOAc = 10/1 as eluent to give 1.21 g (98%) of methyl oleanolate **SI-4** as a white solid.

Analytical data in accordance with literature.[9]

m.p.: 215 – 217 °C, Lit.: 218 – 220 °C[9]

Optical rotation: $[\alpha]_D^{20}$= +69.2 (c 0.5, $CHCl_3$), Lit.:[9] +71.6 (c 1.0, $CHCl_3$)

^{1}H NMR (600 MHz, $CDCl_3$) δ = ^{1}H NMR (600 MHz, $CDCl_3$) δ 5.27 (t, *J* = 3.7 Hz, 1H), 4.70 – 4.37 (m, 1H), 3.62 (s, 3H), 2.85 (dd, *J* = 14.0, 4.6 Hz, 1H), 2.04 (s, 3H), 1.96 (td, *J* = 13.7, 4.1 Hz, 1H), 1.88 (hd, *J* = 11.1, 3.7 Hz, 2H), 1.68 (td, *J* = 13.9, 4.4 Hz, 1H), 1.64 – 1.57 (m, 7H), 1.58 – 1.49 (m, 3H), 1.44 (td, *J* = 12.5, 3.9 Hz, 1H), 1.42 – 1.35 (m, 1H), 1.32 (dd, *J* = 13.9, 4.1 Hz, 1H), 1.28 (dt, *J* = 12.3, 3.0 Hz, 1H), 1.17 (dddd, *J* = 16.4, 6.8, 4.4, 2.5 Hz, 1H), 1.12 (s, 3H), 1.08 – 1.00 (m, 2H), 0.92 (s, 3H), 0.92 (s, 3H), 0.89 (s, 3H), 0.86 (s, 3H), 0.85 (s, 3H), 0.84 – 0.81 (m, 1H), 0.72 (s, 3H).

^{13}C NMR (151 MHz, $CDCl_3$) δ = 178.4, 171.2, 143.9, 122.4, 81.1, 55.4, 51.7, 47.7, 46.9, 45.9, 41.8, 41.4, 39.4, 38.2, 37.8, 37.1, 34.0, 33.2, 32.7, 32.5, 30.8, 28.2, 27.8, 26.0, 23.8, 23.7, 23.5, 23.2, 21.5, 18.3, 17.0, 16.8, 15.5.

Methyl (4aS,6aS,6bR,8aR,10S,12aS,12bR,13R,14bS)-10-acetoxy-13-hydroxy-2,2,6a,6b,9,9,12a-heptamethyl-1,3,4,5,6,6a,6b,7,8,8a,9,10,11,12,12a,12b,13,14b-octadecahydropicene-4a(2H)-carboxylate (SI-5)

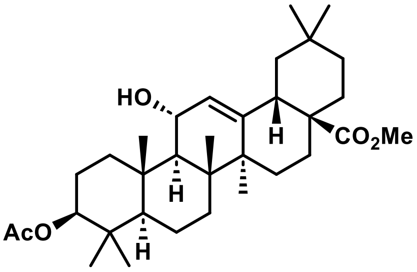

Chemical Formula: $C_{33}H_{52}O_5$
Exact Mass: 528,381

In a 25-mL schlenk-flask was charged methyl acetyl oleanolate **SI-4** (145 mg, 0.28 mmol, 1.0 eq.) in dry dichloromethane (10 mL). The (pentafluorophenyl)tetraporphyriniron(III) catalyst (9 mg, 8 µmol, 3 mol%) was added and the mixture chilled to -78 °C. At this temperature *m*-CPBA (79 mg, 0.35 mmol, 1.25 eq.) was added in 2 portions and the mixture stirred for 4.5 hours. The mixture was then poured onto saturated aq. $NaHCO_3$ and $Na_2S_2O_3$ solution (25 mL each) and extracted with copious amounts of dichloromethane. The crude product was purified by column chromatography to yield 145 mg (97%) of allyl alcohol **SI-5** as light green solid.

Analytical data in accordance with literature.[10]

m.p.: 199 – 201 °C, Lit.: 202 – 205 °C[10]

Optical rotation: $[\alpha]_D^{20}$= +37.0 (c 0.5, CH_2Cl_2), Lit.:[10] +39.92 (c 1.0, $CHCl_3$)

^{1}H NMR (600 MHz, $CDCl_3$) δ = 5.33 (d, *J* = 4.0 Hz, 1H), 4.53 – 4.37 (m, 1H), 4.17 (dd, *J* = 8.0, 4.1 Hz, 1H), 3.61 (s, 3H), 2.86 (dd, *J* = 14.0, 4.5 Hz, 1H), 2.03 (s, 3H), 2.02 – 1.88 (m, 2H), 1.73 – 1.60 (m, 5H), 1.60 – 1.47 (m, 5H), 1.45 – 1.28 (m, 5H), 1.26 – 1.22 (m, 1H), 1.20 (s, 3H), 1.17 (d, *J* = 4.4 Hz, 1H), 1.09 – 1.03 (m, 1H), 1.02 (s, 3H), 0.92 (s, 3H), 0.89 (s, 3H), 0.86 (m, 1H), 0.85 (s, 3H), 0.85 (s, 3H), 0.72 (s, 3H).

^{13}C NMR (151 MHz, $CDCl_3$) δ = 178.2, 171.1, 148.3, 125.7, 80.8, 67.3, 56.6, 55.4, 51.8, 46.4, 45.6, 43.1, 41.9, 40.6, 39.9, 38.1, 38.0, 33.8, 33.1, 33.1, 32.3, 30.8, 28.2, 27.8, 26.4, 23.7, 23.7, 22.9, 21.5, 18.39, 18.38, 16.9, 16.7.

Methyl (4aR,4bR,5aR,6aR,6bS,9S,10aR,12aR,12bS,14aS)-9-acetoxy-3,3,6b,10,10,12a,12b-heptamethyl-6-oxooctadecahydro-1H-piceno[12b,13-b]oxirene-14a(5aH)-carboxylate (28)

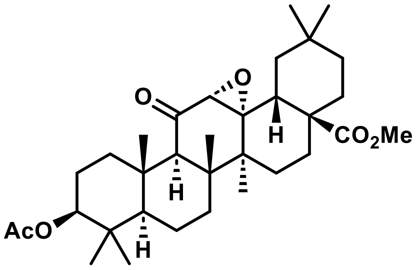

Chemical Formula: $C_{33}H_{50}O_6$
Exact Mass: 542,3607

In a 25-mL round-bottom flask was charged allyl alcohol **SI-4** (100 mg, 0.19 mmol, 1.0 eq.) in dichloromethane (2 mL) and *m*-CPBA (77%, 229 mg, 1.0 mmol, 5.2 eq.) was added. After stirring for 2 hours the reaction was quenched by addition of saturated $NaHCO_3$ and $Na_2S_2O_3$ solutions and extracted with dichloromethane. The pooled organic extracts were dried over $MgSO_4$ and evaporated to give crude product (110 mg) which was directly used in the next step.

The crude epoxide was dissolved in dichloromethane (2 mL) and DMP (154 mg, 0.36 mmol, 1.5 eq.) was added. After 1 hour an additional portion of DMP (154 mg, 0.36 mmol, 1.5 eq.) was added and the mixture left to stir for 16 hours at room temperature. The reaction was then quenched by addition of saturated $NaHCO_3$ and $Na_2S_2O_3$ solutions and extracted with dichloromethane. The pooled organic extracts were dried over $MgSO_4$ and evaporated to give crude product which was purified by column chromatography to give 80 mg (78% over 2 steps) of epoxyketone **28**.

^{1}H NMR (600 MHz, $CDCl_3$) δ = 4.48 (dd, *J* = 11.8, 4.6 Hz, 1H), 3.67 (s, 3H), 3.10 (s, 1H), 2.44 (s, 1H), 2.17 (dt, *J* = 13.5, 3.6 Hz, 1H), 2.06 – 2.04 (m, 1H), 2.04 (s, 3H), 2.03 – 1.99 (m, 1H), 1.87 (td, *J* = 14.1, 4.5 Hz, 1H), 1.71 – 1.57 (m, 6H), 1.50 (d, *J* = 13.7 Hz, 1H), 1.47 – 1.34 (m, 2H), 1.32 – 1.27 (m, 2H), 1.31 (s, 3H), 1.29 (s, 3H), 1.25 (s, 1H), 1.23 – 1.18 (m, 1H), 1.00 (td, *J* = 13.5, 3.7 Hz, 1H), 0.95 (s, 3H), 0.93 (s, 3H), 0.90 – 0.86 (m, 1H), 0.84 (s, 3H), 0.84 (s, 3H), 0.80 (s, 3H), 0.65 (dd, *J* = 11.6, 2.1 Hz, 1H).

^{13}C NMR (151 MHz, $CDCl_3$) δ = 207.7, 177.8, 171.1, 80.6, 70.3, 69.1, 56.2, 54.7, 52.1, 47.8, 47.2, 40.8, 40.6, 38.4, 38.3, 37.8, 36.2, 34.0, 33.9, 33.2, 32.3, 30.6, 29.0, 28.3, 23.4, 23.4, 23.0, 22.6, 21.5, 20.9, 17.7, 17.1, 16.4.

HR-MS (ESI): Calculated for $C_{33}H_{51}O_6$ $[M+H]^+$ 543.3680, found: 543.3697.

Methyl (4aS,6aS,6bR,8aR,10S,12aS,12bR,14bS)-10-acetoxy-14-hydroxy-2,2,6a,6b,9,9,12a-heptamethyl-13-oxo-1,3,4,5,6,6a,6b,7,8,8a,9,10,11,12,12a,12b,13,14b-octadecahydropicene-4a(2H)-carboxylate (29)

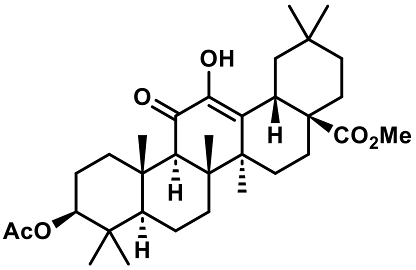

Chemical Formula: $C_{33}H_{50}O_6$
Exact Mass: 542,3607

In a schlenk flask epoxyketone **28** (5 mg, 9.5 µmol, 1.0 eq.) was charged in dry dichloromethane (1 mL). The solution was chilled to -20 °C, at which point boron trifluoride etherate was introduced (1 µL, 10 µmol, 1.0 eq.) and the mixture allowed to slowly warm up to 0 °C. After TLC showed full consumption of starting material, the reaction was quenched by addition of aq. $NaHCO_3$ solution and the dichloromethane layer separated. Extraction of the aqueous phase with dichloromethane (5 mL) and drying ($MgSO_4$) and evaporation of the pooled organic phases gave the crude product. This was purified via column chromatography (1 g SiO_2) to give 4 mg (80%) of enol **29** as a colourless oil.

^{1}H NMR (600 MHz, $CDCl_3$) δ = 6.21 (s, 1H), 4.51 (dd, *J* = 11.7, 4.8 Hz, 1H), 3.74 – 3.64 (m, 1H), 3.63 (s, 3H), 2.80 (dt, *J* = 13.6, 3.7 Hz, 1H), 2.46 (s, 1H), 2.05 (s, 3H), 2.05 – 2.00 (m, 2H), 1.77 (td, *J* = 14.0, 4.4 Hz, 1H), 1.73 – 1.68 (m, 1H), 1.68 – 1.62 (m, 2H), 1.62 – 1.58 (m, 2H), 1.56 (m, 1H), 1.43 – 1.37 (m, 4H), 1.36 (s, 3H), 1.30 – 1.23 (m, 2H), 1.23 – 1.18 (m, 2H), 1.13 (s, 3H), 1.09 (td, *J* = 13.7, 4.3 Hz, 1H), 0.99 (s, 3H), 0.93 (s, 3H), 0.92 (s, 3H), 0.87 (s, 6H), 0.81 (dd, *J* = 11.9, 1.7 Hz, 1H).
^{13}C NMR (151 MHz, $CDCl_3$) δ = 195.5, 178.0, 171.1, 142.3, 136.9, 80.6, 60.5, 55.2, 52.0, 46.2, 45.6, 41.7, 40.4, 38.8, 38.2, 37.5, 34.2, 33.4, 33.3, 33.0, 32.0, 30.7, 28.2, 28.0, 23.7, 23.43, 23.41, 23.3, 21.6, 18.9, 17.4, 16.8, 16.5.
HR-MS (ESI): Calculated for $C_{33}H_{51}O_6$ $[M+H]^+$ 543.3680, found: 543.3691.

Methyl (4aS,6aS,6bR,8aR,10S,12aR,12bR,14bS)-10-hydroxy-2,2,6a,6b,9,9,12a-heptamethyl-1,3,4,5,6,6a,6b,7,8,8a,9,10,11,12,12a,12b,13,14b-octadecahydropicene-4a(2H)-carboxylate (SI-6)

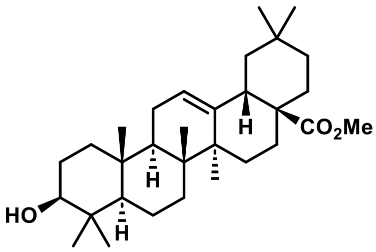

Chemical Formula: $C_{31}H_{50}O_3$
Exact Mass: 470,3760

In a 100-mL round-bottom flask was charged oleanolic acid **3** (95%, 2.00 g, 4.38 mmol, 1.0 eq.) and dissolved in acetone (30 mL). Potassium carbonate (666 mg, 4.82 mmol, 1.1 eq.) was added followed by methyl iodide (0.41 mL, 6.57 mmol, 1.5 eq.). The mixture was stirred at room temperature for 48 hours, concentrated and extracted with aq. $NaHCO_3$ solution and CH_2Cl_2 (200 mL). The crude product was subsequently dried at 1 mbar to provide 2.05 g (99%) pure methyl ester **SI-6**. Alternatively, the reaction could be carried out in DMF (5 mL per gram of **SI-6**) which resulted in complete conversion in 1 hour and a slightly lower yield of 95%.

Analytical data in accordance with literature.[11]

m.p.: 199 – 202 °C, Lit.: 198 – 200 °C [11]

Optical rotation: $[\alpha]_D^{20}$= +69.1 (c 0.86, MeOH), Lit.:[11] +66.5 (c 0.34, $CHCl_3$)

^{1}H NMR (600 MHz, $CDCl_3$) δ = 5.27 (t, *J* = 3.7, 1H), 3.62 (s, 3H), 3.20 (dd, *J* = 11.3, 4.3, 1H), 2.85 (dd, *J* = 14.0, 4.6, 1H), 1.96 (td, *J* = 14.1, 13.4, 4.1, 1H), 1.88 (hd, *J* = 11.2, 3.7, 2H), 1.71 – 1.64 (m, 1H), 1.64 – 1.48 (m, 9H), 1.47 – 1.24 (m, 6H), 1.20 – 1.14 (m, 1H), 1.12 (s, 3H), 1.08 – 1.02 (m, 1H), 0.98 (s, 3H), 0.92 (s, 3H), 0.90 (s, 3H), 0.89 (s, 3H), 0.77 (s, 3H), 0.73 (d, *J*=2.1, 1H), 0.72 (s, 3H).

^{13}C NMR (151 MHz, $CDCl_3$) δ = 178.4, 143.9, 122.5, 79.1, 55.4, 51.7, 47.8, 46.9, 46.0, 41.8, 41.4, 39.4, 38.9, 38.6, 37.2, 34.0, 33.3, 32.8, 32.5, 30.8, 28.2, 27.8, 27.3, 26.1, 23.8, 23.5, 23.2, 18.5, 17.0, 15.7, 15.4.

HR-MS (ESI): Calculated for $C_{31}H_{51}O_3$ $[M+H]^+$ 471.3832, found: 471.3828.

Methyl (4aS,6aS,6bR,8aR,10S,12aS,12bR,13R,14bS)-13-hydroperoxy-10-hydroxy-2,2,6a,6b,9,9,12a-heptamethyl-1,3,4,5,6,6a,6b,7,8,8a,9,10,11,12,12a,12b,13,14b-octadecahydropicene-4a(2H)-carboxylate (26)

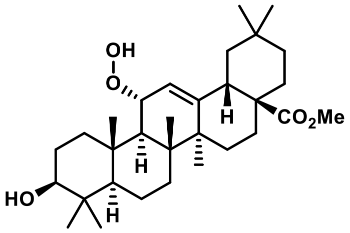

In a microwave vial was charged starting material **SI-6** (100 mg, 0.21 mmol, 1.0 eq.), followed by tetrabutylammonium bromide (14 mg, 0.04 mmol, 20 mol%) and Rose Bengal (4 mg, 4 μmol, 2 mol%). The solids were dissolved in acetonitrile/chloroform (5 mL, 10/1 ratio and oxygen was bubbled through the solution for 2 minutes. The vial was closed, ensuring oxygen atmosphere via a balloon. A blue LED lamp (Kessil PR160L, 456 nm, 40 W) was then used to irradiate the mixture under strong stirring over 16 hours. Afterwards, the reaction was concentrated and directly applied to column chromatography (6 g SiO_2) to give 25 mg (24%) of C-11 ketone (not depicted) as a side product, as well as 65 mg (61%) of hydroperoxide **26** as a white solid.

m.p.: 145 – 148 °C.

Optical rotation: $[\alpha]_D^{20}$= -1.8 (c 0.5, $CHCl_3$).

^{1}H NMR (600 MHz, $CDCl_3$) δ = 5.56 (d, *J* = 3.9 Hz, 1H), 4.49 (dd, *J* = 8.7, 3.9 Hz, 1H), 3.63 (s, 3H), 3.23 (dd, *J* = 11.6, 4.6 Hz, 1H), 3.07 – 2.83 (m, 1H), 1.99 (td, *J* = 13.7, 4.1 Hz, 1H), 1.94 (dt, *J* = 13.8, 3.5 Hz, 1H), 1.77 (d, *J* = 8.6 Hz, 1H), 1.73 – 1.67 (m, 1H), 1.66 – 1.63 (m, 3H), 1.62 – 1.50 (m, 5H), 1.49 – 1.30 (m, 5H), 1.25 (m, 2H), 1.22 (s, 3H), 1.21 – 1.19 (m, 1H), 1.08 (dt, *J* = 14.3, 3.5 Hz, 1H), 1.00 (s, 3H), 0.99 (s, 3H), 0.95 (s, 3H), 0.91 (s, 3H), 0.78 (s, 3H), 0.78 – 0.75 (m, 1H), 0.75 (s, 3H).

^{13}C NMR (151 MHz, $CDCl_3$) δ = 178.2, 151.9, 121.6, 81.4, 78.8, 55.2, 51.8, 50.3, 46.4, 46.0, 43.0, 42.0, 41.0, 39.4, 39.1, 38.1, 33.9, 33.2, 33.0, 32.3, 30.9, 28.2, 28.1, 27.4, 24.9, 23.8, 23.0, 18.7, 18.5, 16.9, 15.6.

HR-MS (ESI): Calculated for $C_{31}H_{50}O_5Na[M+Na]^+$ 525.3550, found: 525.3553.

Methyl (4aS,6bR,8aR,10S,12aS,12bR,12cS,13aR,13bS,13cS)-10-hydroxy-2,2,6b,9,9,12a,13b-heptamethyl-1,3,4,5,6b,7,8,8a,9,10,11,12,12a,12b,12c,13a,13b,13c-octadecahydropiceno[13,14-b]oxirene-4a(2H)-carboxylate (27)

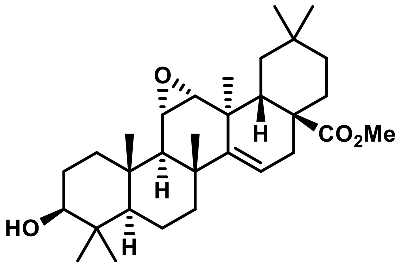

Chemical Formula: $C_{31}H_{48}O_4$
Exact Mass: 484,3553

In a 10-mL round-bottom flask was charged hydroperoxide **26** (12 mg, 24 µmol, 1.0 eq.) and dissolved in dry dichloromethane (1.5 mL). The solution was chilled to 0 °C and tin(II)-triflate (2 mg, 5 µmol, 20 mol%) was introduced and stirred for 20 minutes. After that the reaction mixture was quenched by addition of saturated aq. $NaHCO_3$ solution and extracted with ethyl acetate (10 mL). Purification via column chromatography (1 g SiO_2) provided 7.5 mg (64%) of epoxide **27** as a white solid.

m.p.: 138 °C

Optical rotation: $[\alpha]_D^{20}$= -2.5 (c 0.4, $CHCl_3$)

^{1}H NMR (600 MHz, $CDCl_3$) δ = 5.48 (dd, *J* = 8.1, 3.6 Hz, 1H), 3.58 (s, 3H), 3.23 (dd, *J* = 11.3, 4.9 Hz, 1H), 3.13 (t, *J* = 5.1 Hz, 1H), 2.98 (d, *J* = 4.6 Hz, 1H), 2.74 – 2.63 (m, 1H), 2.48 (ddd, *J* = 14.9, 8.1, 1.2 Hz, 1H), 2.03 – 1.99 (m, 1H), 1.97 (dt, *J* = 13.1, 3.3 Hz, 1H), 1.89 (dt, *J* = 13.4, 3.5 Hz, 1H), 1.75 – 1.66 (m, 2H), 1.66 – 1.59 (m, 2H), 1.58 – 1.44 (m, 3H), 1.43 – 1.40 (m, 1H), 1.30 (dd, *J* = 13.7, 3.7 Hz, 1H), 1.27 – 1.22 (m, 3H), 1.05 (s, 3H), 1.03 (dd, *J* = 14.0, 3.1 Hz, 1H), 0.98 (s, 3H), 0.98 (s, 2x 3H), 0.96 (d, *J* = 5.5 Hz, 1H), 0.90 (s, 3H), 0.84 (s, 3H), 0.81 (s, 3H), 0.73 (dd, *J* = 12.1, 2.2 Hz, 1H).

^{13}C NMR (151 MHz, $CDCl_3$) δ = 178.3, 159.8, 118.1, 79.1, 58.8, 54.7, 53.7, 52.1, 52.0, 51.1, 41.7, 40.2, 39.0, 38.8, 38.4, 37.5, 36.7, 35.4, 34.0, 32.6, 32.4, 31.2, 29.3, 28.0, 27.5, 27.0, 24.0, 20.6, 19.0, 17.1, 15.6

HR-MS (ESI): Calculated for $C_{31}H_{48}O_4Na[M+Na]^+$ 507.3445, found: 507.3455.

Synthesis of Alstoscholarinoid B

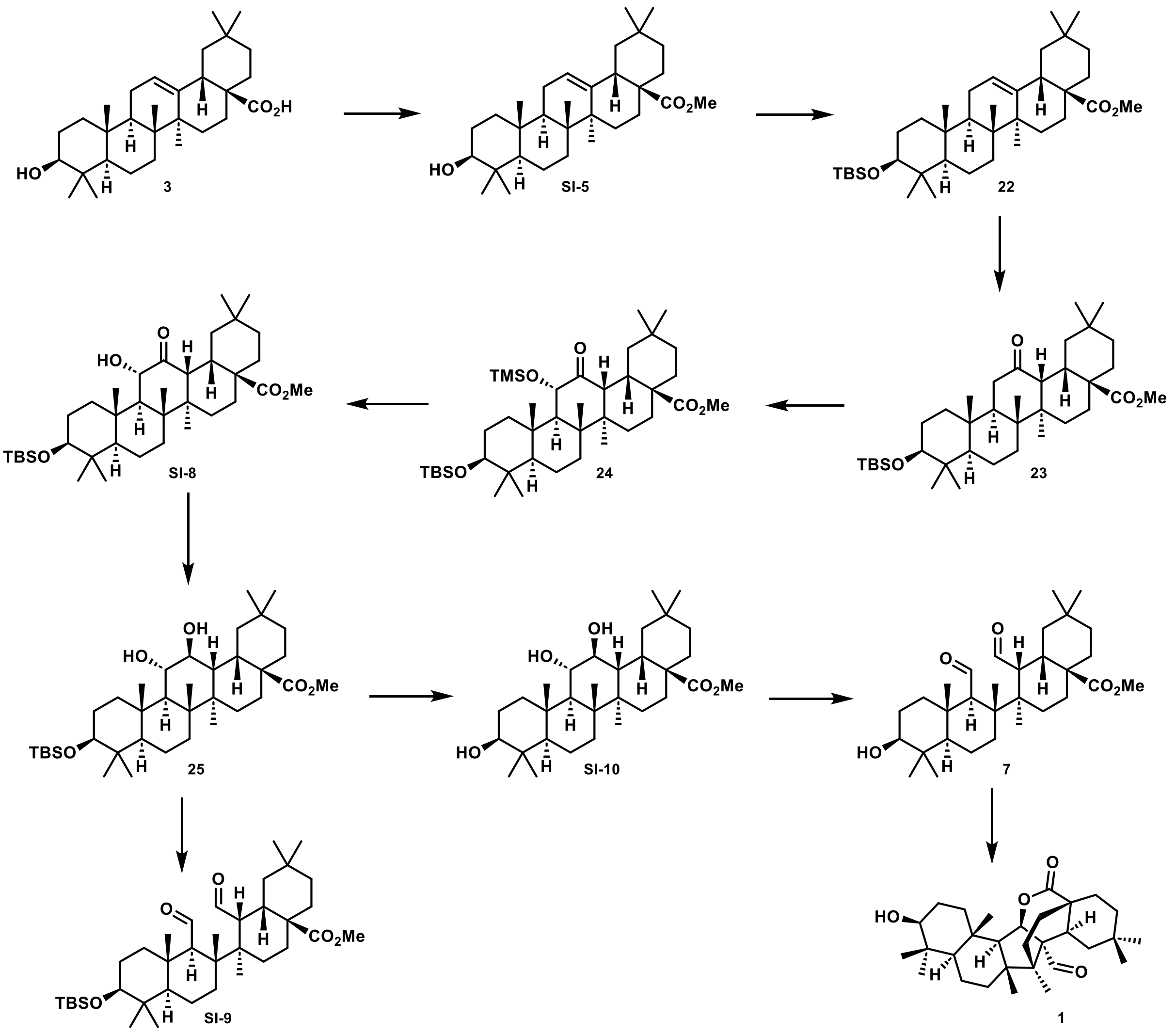

Scheme S6: Synthetic route towards Alstoscholarinoid B (**2**)

The cleavage of the *trans*-diol compounds **25** and **SI-10** was feasible in our hands, in contrast to the previous report by Shi [12], who re-routed through a *cis*-diol. Reagents capable of effecting the desired cleavage were $PhI(OAc)_2$ and $Pb(OAc)_4$. With C-3 deprotected diol a solvent switch from dichloromethane to chloroform was necessary to increase concentration in the reaction media. For the aldol addition reaction, we screened strong bases (LDA, LiHMDS) as well as enamine catalysis systems (pyrrolidine/AcOH, L-proline, etc.), eventually using optimized conditions from the Shi group [12].

Methyl (4aS,6aS,6bR,8aR,10S,12aR,12bR,14bS)-10-((*tert*-butyldimethylsilyl)oxy)-*2,2,6a,6b,9,9,12a-heptamethyl-1,3,4,5,6,6a,6b,7,8,8a,9,10,11,12,12a,12b,13,14b-* octadecahydropicene-4a(2H)-carboxylate (22)

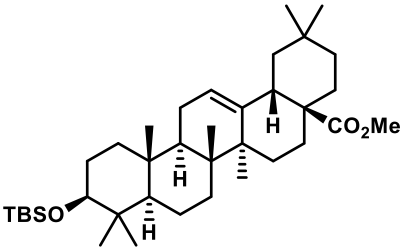

Chemical Formula: $C_{37}H_{64}O_3Si$
Exact Mass: 584,4625

In a 100-mL round-bottom flask methyl oleanolate **SI-6** (3.85 g, 8.18 mmol, 1.0 eq.) was charged in dry DMF (40 mL). The solution was treated with TBSCl (2.80 g, 18 mmol, 2.2 eq.) and imidazole (1.67 g, 24.5 mmol, 3.0 eq.) and heated to 50 °C in a water bath. After 16 hours the reaction was complete and the mixture was flooded with saturated aq. $NaHCO_3$ solution (100 mL). Extraction with EtOAc (3x), washing with brine and drying over $MgSO_4$ provided the crude product, which was purified via flash chromatography to give 3.95 g (83%) of the product **22** as a white solid.

m.p.: 187 – 190 °C

Optical rotation: $[\alpha]_D^{20}$= +78.9 (c 0.78, MeOH)

^{1}H NMR (600 MHz, $CDCl_3$) δ = 5.28 (t, *J* = 3.7 Hz, 1H), 3.62 (s, 3H), 3.18 (dd, *J* = 11.4, 4.5 Hz, 1H), 2.85 (dd, *J* = 13.9, 4.6 Hz, 1H), 2.02 – 1.93 (m, 1H), 1.86 (qdt, *J* = 10.8, 6.9, 3.7 Hz, 2H), 1.68 (td, *J* = 13.9, 4.5 Hz, 1H), 1.65 – 1.55 (m, 4H), 1.54 – 1.48 (m, 5H), 1.48 – 1.37 (m, 2H), 1.37 – 1.30 (m, 2H), 1.26 (dt, *J* = 12.2, 2.9 Hz, 1H), 1.21 – 1.17 (m, 1H), 1.15 (dd, *J* = 7.7, 5.4 Hz, 1H), 1.12 (s, 3H), 1.08 – 1.02 (m, 1H), 0.92 (s, 3H), 0.89 (s 3x 3H), 0.88 (s, 9H), 0.74 (s, 3H), 0.71 (s, 3H), 0.69 (d, *J* = 1.8 Hz, 1H), 0.03 (s, 2x 3H).

^{13}C NMR (151 MHz, $CDCl_3$) δ = 178.5, 143.9, 122.6, 79.6, 55.5, 51.7, 47.8, 46.9, 46.0, 41.8, 41.4, 39.5, 39.4, 38.6, 37.1, 34.0, 33.3, 32.9, 32.5, 30.9, 28.7, 27.83, 27.76, 26.10, 26.07 (3C), 23.8, 23.6, 23.2, 18.7, 18.3, 17.0, 16.2, 15.5, -3.6, -4.8.

HR-MS (ESI): Calculated for $C_{37}H_{65}O_3Si$ $[M+H]^+$ 585.4698, found: 585.4690.

Methyl (4aS,6aR,6bR,8aR,10S,12aR,12bR,14aR,14bS)-10-((*tert*-butyldimethylsilyl)oxy)-2,2,6a,6b,9,9,12a-heptamethyl-14-oxoicosahydropicene-4a(2H)-carboxylate (23)

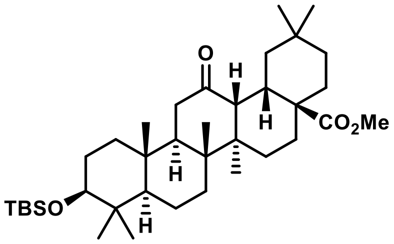

Chemical Formula: $C_{37}H_{64}O_4Si$
Exact Mass: 600,4574

In a 100-mL round-bottom flask olefin **22** (3.95 g, 6.75 mmol, 1.0 eq.) was dissolved in chloroform (10 mL). To the solution m-CPBA (3.18 g, 14.2 mmol, 2.1 eq.) was added in two portions (1.3 eq. followed by 0.8 eq. after 1 hour). After stirring at room temperature for 2 hours the reaction was concentrated and the solvent switched for dry toluene (20 mL). After chilling the solution to -30 °C, boron trifluoride etherate (1.08 g, 7.43 mmol, 1.1 eq.) was introduced and stirred for 30 minutes. After allowing to warm to 0 °C the mixture was quenched by addition of saturated aq. $NaHCO_3$ and $Na_2S_2O_3$ solutions and extracted with dichloromethane (3x). Pooled organic extracts were washed with brine and dried over $MgSO_4$. Evaporation provided the crude product which was purified by flash chromatography using pentane/EtOAc = 8/1 to give 2.87 g (71%) of ketone **23** as a white solid.

m.p.: 170 °C

Optical rotation: $[\alpha]_D^{20}$= -8.5 (c 0.91, MeOH)

^{1}H NMR (600 MHz, $CDCl_3$) δ = 3.68 (s, 3H), 3.15 (dd, *J* = 11.4, 4.4 Hz, 1H), 2.89 – 2.68 (m, 1H), 2.61 (d, *J* = 4.3 Hz, 1H), 2.23 (dd, *J* = 16.7, 5.0 Hz, 1H), 2.12 (dd, *J* = 16.7, 13.1 Hz, 1H), 1.94 (ddd, *J* = 13.5, 3.7, 2.3 Hz, 1H), 1.89 (td, *J* = 14.7, 4.4 Hz, 1H), 1.79 (td, *J* = 13.8, 4.6 Hz, 1H), 1.70 – 1.62 (m, 2H), 1.62 – 1.57 (m, 1H), 1.55 – 1.51 (m, 1H), 1.51 – 1.44 (m, 3H), 1.44 – 1.38 (m, 1H), 1.32 (ddtd, *J* = 15.1, 10.3, 5.6, 2.0 Hz, 2H), 1.25 – 1.17 (m, 2H), 1.17 – 1.10 (m, 1H), 1.07 (ddd, *J* = 13.2, 4.3, 1.9 Hz, 1H), 0.97 (s, 3H), 0.95 (s, 3H), 0.93 (s, 3H), 0.90 (s, 3H), 0.90 (s, 3H), 0.90 – 0.84 (m, 2H), 0.88 (s, 9H), 0.84 (s, 3H), 0.74 (s, 3H), 0.69 (dd, *J* = 11.6, 2.2 Hz, 1H), 0.03 (s, 3H), 0.02 (s, 3H).

^{13}C NMR (151 MHz, $CDCl_3$) δ = 212.2, 178.6, 79.3, 55.3, 52.0, 49.9, 47.5, 42.0, 41.4, 39.5, 38.7, 38.1, 37.0, 36.3, 34.6, 33.6, 33.1, 32.1, 30.8, 28.5, 27.7, 27.6, 26.0 (3C), 23.3, 22.9, 20.6, 18.6, 18.3, 16.3, 16.0, 15.4, 14.5, 11.6, -3.6, -4.8.

HR-MS (ESI): Calculated for $C_{37}H_{64}O_4SiNa$ $[M+Na]^+$ 623.4466, found: 623.4468.

Methyl (4aS,6aR,6bR,8aR,10S,12aS,12bR,13S,14aR,14bS)-10-((*tert*-butyldimethylsilyl)oxy)-2,2,6a,6b,9,9,12a-heptamethyl-14-oxo-13-((trimethylsilyl)oxy)icosahydropicene-4a(2H)-carboxylate (**24**)

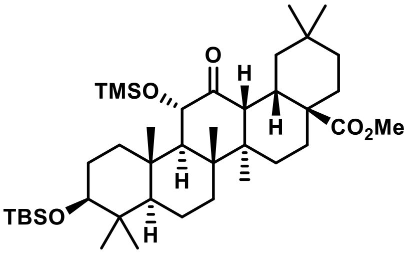

Chemical Formula: $C_{40}H_{72}O_5Si_2$
Exact Mass: 688,4918

In a schlenk-flask was placed ketone **23** (1.00 g, 1.61 mmol, 1.0 eq.) and dissolved in dry THF (60 mL). The solution was chilled to -75 °C and treated with LiHMDS solution (1M, 9.61 g, 10.8 mmol, 6.5 eq.) followed by TMSCl (0.72 g, 6.64 mmol, 4.0 eq.) after 30 minutes. After stirring for 1 hour at -70 °C the solution slowly allowed to warm up to 0 °C. The reaction was diluted with toluene and shaken with saturated aq. $NaHCO_3$ solution once. After evaporation of the toluene phase the crude enol ether intermediate **SI-7** was dissolved in dichloromethane (10 mL) and stirred at 0 °C. A solution of *m*-CPBA (0.584 g, 2.61 mmol, 1.5 eq.) was added dropwise and stirred for further 15 minutes after the addition was finished. The mixture was then concentrated and applied directly to column chromatography using pentane/EtOAc = 15/1 as eluent to give 1.03 g (90%) of ketone **24** as a colourless solid.

m.p.: 218 – 220 °C.

Optical rotation: $[\alpha]_D^{20}$= -33.1 (c 0.84, MeOH).

^{1}H NMR (600 MHz, $CDCl_3$) δ = 4.04 (dd, *J* = 10.3, 0.8 Hz, 1H), 3.68 (s, 3H), 3.15 (dd, *J* = 11.5, 4.6 Hz, 1H), 2.95 – 2.73 (m, 1H), 2.66 (d, *J* = 4.3 Hz, 1H), 2.09 (dt, *J* = 14.0, 3.6 Hz, 1H), 1.95 – 1.84 (m, 2H), 1.79 (td, *J* = 13.7, 4.6 Hz, 1H), 1.68 – 1.58 (m, 3H), 1.54 – 1.49 (m, 1H), 1.47 (ddd, *J* = 13.8, 4.2, 2.9 Hz, 1H), 1.44 – 1.37 (m, 2H), 1.32 (td, *J* = 13.7, 4.2 Hz, 1H), 1.28 – 1.23 (m, 1H), 1.23 – 1.17 (m, 2H), 1.12 – 1.03 (m, 2H), 0.97 (s, 3H), 0.97 (s, 3H), 0.96 (s, 3H), 0.94 (s, 3H), 0.90 (s, 3H), 0.88 (s, 9H), 0.75 (s, 3H), 0.73 – 0.70 (m, 1H), 0.13 (s, 9H), 0.03 (s, 3H), 0.02 (s, 3H).

^{13}C NMR (151 MHz, $CDCl_3$) δ = 209.4, 177.4, 77.8, 75.3, 55.2, 54.3, 50.8, 49.4, 46.4, 40.9, 40.2, 39.0, 38.8, 37.6, 35.1, 33.4, 32.3, 31.84, 31.78, 31.4, 27.5, 26.8, 26.6, 24.9 (3C), 22.2, 21.8, 19.37, 17.29, 17.0, 16.9, 15.2, 14.9, 4.8, -0.1 (3C), -4.2, -6.0.

HR-MS (ESI): Calculated for $C_{40}H_{72}O_5Si_2Na$ $[M+Na]^+$ 711.4810, found: 711.4816.

Methyl (4aS,6aR,6bR,8aR,10S,12aS,12bR,13S,14aR,14bS)-10-((*tert*-butyldimethylsilyl)oxy)-13-hydroxy-2,2,6a,6b,9,9,12a-heptamethyl-14-oxoicosahydropicene-4a(2H)-carboxylate (SI-8)

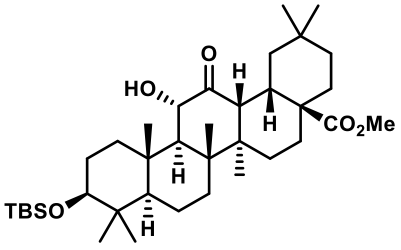

Chemical Formula: $C_{37}H_{64}O_5Si$
Exact Mass: 616,4523

In a falcon tube was charged silylated ketone **24** (600 mg, 0.87 mmol, 1.0 eq.) and dissolved in dry THF (20 mL). HF-pyridine complex (70% HF, 1.35 mL, 15.0 mmol, 17.2 eq.) was added and the mixture stirred until starting material consumption was complete by TLC. Reaction was quenched by addition of saturated aq. $NaHCO_3$ solution and extraction with dichloromethane. The product was obtained after flash chromatography using pentane/EtOAc = 10/1 as eluent to give 510 mg (95%) of **SI-8** as a white solid.

m.p.: 188 – 190 °C

Optical rotation: $[\alpha]_D^{20}$= -20.4 (c 0.41, MeOH).

^{1}H NMR (600 MHz, $CDCl_3$) δ = 4.03 (dt, *J* = 11.1, 1.5 Hz, 1H), 4.00 (d, *J* = 1.6 Hz, 1H), 3.69 (s, 3H), 3.16 (dd, *J* = 11.5, 4.6 Hz, 1H), 2.88 (d, *J* = 4.2 Hz, 1H), 2.82 – 2.73 (m, 1H), 2.32 (dt, *J* = 13.9, 3.6 Hz, 1H), 2.12 (ddd, *J* = 13.4, 3.6, 2.2 Hz, 1H), 1.91 (td, *J* = 13.2, 4.4 Hz, 1H), 1.79 (td, *J* = 13.8, 4.6 Hz, 1H), 1.68 – 1.56 (m, 3H), 1.52 – 1.44 (m, 3H), 1.44 – 1.38 (m, 1H), 1.34 (td, *J* = 13.7, 4.2 Hz, 1H), 1.31 – 1.24 (m, 2H), 1.24 – 1.19 (m, 1H), 1.14 – 1.09 (m, 1H), 1.08 – 1.01 (m, 1H), 1.05 (s, 3H), 0.98 (s, 3H), 0.93 (s, 3H), 0.91 (s, 3H), 0.90 (s, 3H), 0.88 (s, 9H), 0.77 (s, 3H), 0.71 (dd, *J* = 11.7, 2.2 Hz, 1H), 0.02 (s, 6H).

^{13}C NMR (151 MHz, $CDCl_3$) δ = 213.3, 178.3, 79.0, 75.3, 56.5, 55.4, 52.0, 49.5, 47.4, 42.9, 42.5, 40.0, 39.9, 39.0, 36.3, 34.3, 33.4, 32.92, 32.87, 32.5, 30.7, 28.6, 28.1, 27.8, 25.9 (3C), 23.2, 22.6, 20.3, 18.4, 18.1, 17.6, 16.1, 15.9, -3.7, -4.9.

HR-MS (ESI): Calculated for $C_{37}H_{64}O_5SiNa$ $[M+Na]^+$ 639.4415, found: 639.4416.

Methyl (4aS,6aR,6bR,8aR,10S,12aS,12bR,13S,14S,14aR,14bS)-10-((*tert*-butyldimethylsilyl)oxy)-13,14-dihydroxy-2,2,6a,6b,9,9,12a-heptamethylicosahydropicene-4a(2H)-carboxylate (25)

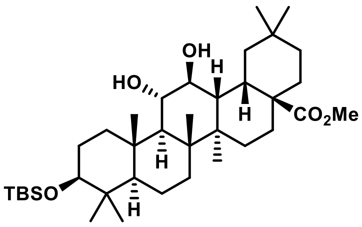

Chemical Formula: $C_{37}H_{66}O_5Si$
Exact Mass: 618,4680

In a schlenk flask was charged α-hydroxy ketone **SI-8** (510 mg, 0.83 mmol, 1.0 eq) in dry methanol (50 mL) and dry THF (30 mL) and chilled to 0 °C. To the solution $NaBH_4$ (159 mg, 4.12 mmol, 5.2 eq.) was added and the mixture stirred at 0 °C until complete consumption of starting material was detected by TLC (2.5 hours). The reaction mixture was quenched by addition of saturated aq. NH_4Cl and extraction with diethyl ether (3x). After washing with brine, drying over $MgSO_4$ and evaporating, the crude product was recrystallized from ethanol/water = 1/1. The crystalline mass obtained was further purified by flash chromatography to give 435 mg (85%) of analytically pure alcohol **25** as a colourless solid.

m.p.: 175 – 176 °C

Optical rotation: $[\alpha]_D^{20}$= +12.9 (c 0.61, MeOH)

^{1}H NMR (600 MHz, $CDCl_3$) δ = 3.96 – 3.81 (m, 2H), 3.67 (s, 3H), 3.18 (dd, *J* = 11.6, 4.6 Hz, 1H), 2.56 (dt, *J* = 13.5, 3.7 Hz, 1H), 2.49 (dtd, *J* = 14.0, 4.5, 1.6 Hz, 1H), 2.10 (d, *J* = 3.4 Hz, 1H), 1.88 (tdd, *J* = 7.7, 6.0, 3.4 Hz, 3H), 1.77 – 1.67 (m, 3H), 1.64 – 1.56 (m, 4H), 1.55 – 1.36 (m, 5H), 1.33 (td, *J* = 13.4, 4.0 Hz, 1H), 1.30 (s, 3H), 1.24 (dt, *J* = 12.3, 2.7 Hz, 1H), 1.18 (dtd, *J* = 15.2, 5.1, 3.2 Hz, 1H), 1.05 (s, 3H), 1.05 – 0.99 (m, 1H), 0.94 (s, 3H), 0.91 (s, 3H), 0.89 (s, 6H), 0.88 (s, 9H), 0.75 (s, 3H), 0.73 – 0.70 (m, 1H), 0.02 (d, *J* = 1.2 Hz, 6H).

^{13}C NMR (151 MHz, $CDCl_3$) δ = 179.1, 79.0, 78.6, 73.0, 56.2, 52.0, 50.1, 48.7, 43.5, 41.2, 40.5, 40.2, 39.8, 39.7, 38.6 (2C), 34.6, 34.2, 33.7, 33.2, 30.9, 30.8, 28.8, 28.1, 26.1 (3C), 23.9, 23.7, 20.5, 18.3, 18.2, 17.6, 16.7, 16.3, -3.6, -4.8.

HR-MS (ESI): Calculated for $C_{37}H_{66}O_5SiNa$ $[M+Na]^+$ 641.4572, found: 641.4580.

Methyl (1R,1'R,2R,2'R,4aS,4'aR,6'S,8aS,8'aS)-6'-((tert-butyldimethylsilyl)oxy)-1,1'-diformyl-2,2',5',5',7,7,8'a-heptamethyloctadecahydro-[2,2'-binaphthalene]-4a(2H)-carboxylate (SI-9)

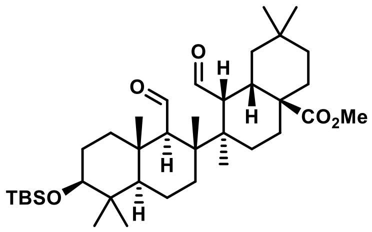

Chemical Formula: $C_{37}H_{64}O_5Si$
Exact Mass: 616,4523

In a round-bottom flask was charged TBS-protected diol **25** (99 mg, 0.16 mmol, 1.0 eq.) and dissolved in dichloromethane (1 mL). Chilled to -12 °C and $PhI(OAc)_2$ (57 mg, 0.18 mmol, 1.1 eq.) was introduced and stirred for 25 minutes. The reaction mixture was filtered over a short silica plug with DCM and evaporated. Purification by column chromatography (8 g SiO_2) using pentane/EtOAc = 15/1 provided 70 mg (71%) of dialdehyde **SI-9** as a white solid.

m.p.: 161 – 163 °C

Optical rotation: $[\alpha]_D^{20}$= -25.6 (c 0.45, MeOH)

^{1}H NMR (600 MHz, CD_2Cl_2) δ = 10.02 (d, *J* = 5.3 Hz, 1H), 9.93 (d, *J* = 6.1 Hz, 1H), 3.76 (s, 3H), 3.16 (dd, *J* = 11.4, 4.7 Hz, 1H), 2.67 (dt, *J* = 14.2, 5.1 Hz, 1H), 2.60 (t, *J* = 5.3 Hz, 1H), 1.92 (td, *J* = 13.7, 3.8 Hz, 1H), 1.87 (d, *J* = 6.2 Hz, 1H), 1.77 – 1.65 (m, 3H), 1.63 – 1.42 (m, 7H), 1.40 – 1.30 (m, 3H), 1.26 (dt, *J* = 13.5, 3.6 Hz, 1H), 1.22 (s, 3H), 1.19 (s, 3H), 1.14 (s, 3H), 1.12 – 1.06 (m, 1H), 0.89 (s, 3H), 0.87 (s, 10H), 0.86 (s, 4H), 0.82 (s, 3H), 0.72 (s, 3H), 0.53 (dd, *J* = 12.7, 2.5 Hz, 1H), 0.03 (s, 3H), 0.03 (s, 3H).

^{13}C NMR (151 MHz, $CDCl_3$) δ = 208.49, 208.45, 177.6, 79.1, 65.1, 54.0, 52.9, 52.5, 46.7, 45.7, 43.2, 39.8, 39.2, 38.51, 38.48, 38.0, 36.4, 34.1, 33.2, 33.0, 31.6, 30.9, 28.4, 27.5, 26.0 (3C), 23.6, 23.23, 19.27, 19.1, 18.3, 18.2 (2C), 16.0, -3.8, -4.9.

HR-MS (ESI): Calculated for $C_{37}H_{65}O_5Si$ $[M+H]^+$ 617.4596, found: 617.4601.

Methyl (4aS,6aR,6bR,8aR,10S,12aS,12bR,13S,14S,14aR,14bS)-10,13,14-trihydroxy-2,2,6a,6b,9,9,12a-heptamethylicosahydropicene-4a(2H)-carboxylate (SI-10)

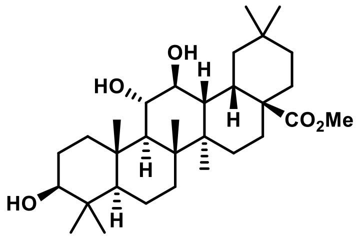

Chemical Formula: $C_{31}H_{52}O_5$
Exact Mass: 504,3815

In a 50-mL round bottom flask was charged diol **25** (98 mg, 0.16 mmol, 1.0 eq.) and dissolved in diethyl ether (3 mL). In a separate flask dry methanol (16 mL) was chilled to 0 °C and treated with acetyl chloride (0.7 mL, 9.5 mmol, 60 eq.) dropwise. After stirring the mixture for two minutes, it was added to the solution of **25**. After 2 hours the reaction was complete and quenched by addition of saturated aq. $NaHCO_3$ solution. After extraction with dichloromethane the crude product could be crystallized from hot dichloromethane to give 77 mg (96%) of triol **SI-10** as a white solid.

All analytical data in agreement with the literature.[9]

m.p.: 157 – 158 °C, Lit.:[9] 156 – 157 °C

Optical rotation: $[\alpha]_D^{20}$= -5.6 (c 0.13, CH_2Cl_2), Lit.: $[\alpha]_D^{25}$= -10.5 (c 0.14, CH_2Cl_2),

^{1}H NMR (600 MHz, $CDCl_3$) δ = 3.89 – 3.82 (m, 2H), 3.67 (s, 3H), 3.25 – 3.18 (m, 1H), 2.64 (dt, *J* = 13.4, 3.6 Hz, 1H), 2.49 (dtd, *J* = 13.9, 4.6, 1.6 Hz, 1H), 1.97 – 1.83 (m, 3H), 1.78 – 1.68 (m, 2H), 1.64 (d, *J* = 10.8 Hz, 1H), 1.58 (ddd, *J* = 11.8, 7.0, 2.9 Hz, 2H), 1.55 – 1.43 (m, 2H), 1.40 (td, *J* = 13.3, 3.4 Hz, 1H), 1.33 (dt, *J* = 12.5, 3.8 Hz, 1H), 1.30 (s, 3H), 1.28 – 1.24 (m, 2H), 1.21 – 1.15 (m, 1H), 1.06 (s, 3H), 1.02 (dt, *J* = 13.9, 3.5 Hz, 1H), 0.98 (s, 3H), 0.94 (s, 3H), 0.91 (s, 3H), 0.78 (s, 3H), 0.74 (dd, *J* = 12.1, 1.8 Hz, 1H).

^{13}C NMR (151 MHz, $CDCl_3$) δ = 179.1, 78.60, 78.55, 72.9, 56.1, 51.9, 50.0, 48.7, 43.5, 41.1, 40.5, 39.8, 39.7, 39.6, 38.7, 38.6, 34.5, 34.2, 33.6, 33.2, 30.9, 30.8, 28.4, 27.7, 23.9, 23.7, 20.5, 18.0, 17.6, 16.6, 15.8.

Methyl (1R,1'R,2R,2'R,4aS,4'aR,6'S,8aS,8'aS)-1,1'-diformyl-6'-hydroxy-2,2',5',5',7,7,8'a-heptamethyloctadecahydro-[2,2'-binaphthalene]-4a(2H)-carboxylate (7)

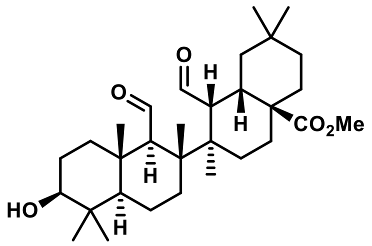

Chemical Formula: $C_{31}H_{50}O_5$
Exact Mass: 502,3658

In a 10-mL Schlenk flask was charged triol **SI-9** (77 mg, 0.15 mmol, 1.0 eq.) in dry chloroform (4 mL, substrate is not significantly soluble in dichloromethane) and chilled to -12 °C. A solution of $PhI(OAc)_2$ (54 mg, 0.17 mmol, 1.1 eq.) was added in dry chloroform (1 mL) was added and full conversion observed by TLC after 10 minutes. The reaction mixture was filtered over a short plug of silica and evaporated after washing with dichloromethane. The crude material (94 mg) was purified by column chromatography (10 g SiO_2) using pentane/EtOAc = 2:1 to pure EtOAc to yield 57 mg (74%) of dialdehyde **7** as a white solid.

Analytical data in accordance with literature.[12]

m.p.: 179 °C, Lit.: 183 – 184 °C[12]

Optical rotation: $[\alpha]_D^{20}$= -45.2 (c 0.2, CH_2Cl_2), Lit.: -41.9 (c 0.12, CH_2Cl_2)[12]

^{1}H NMR (600 MHz, $CDCl_3$) δ = 10.03 (d, *J* = 5.2 Hz, 1H), 9.96 (d, *J* = 6.1 Hz, 1H), 3.80 (s, 3H), 3.20 (dd, *J*= 11.6, 4.7 Hz, 1H), 2.78 – 2.71 (m, 1H), 2.67 (t, *J* = 5.2 Hz, 1H), 1.96 – 1.86 (m, 2H), 1.82 – 1.44 (m, 12H), 1.40 – 1.25 (m, 5H), 1.24 (s, 3H), 1.21 (s, 3H), 1.15 (s, 3H), 1.10 (m, 1H), 0.95 (s, 3H), 0.90 (s, 3H), 0.84 (s, 3H), 0.76 (s, 3H), 0.56 (dd, *J* = 12.3, 2.3 Hz, 1H).

^{13}C NMR (151 MHz, $CDCl_3$) δ = 208.19, 208.15, 177.5, 78.3, 64.6, 53.6, 52.5, 52.4, 46.5, 45.5, 43.1, 39.0, 38.9, 38.4, 38.0, 37.9, 35.9, 34.0, 33.3, 32.7, 31.3, 30.8, 28.0, 27.9, 26.8, 23.35, 23.27, 18.8, 18.1, 15.6.

Alstoscholarinoid B, (4aS,6aS,6bR,8aR,10S,12aS,12bR,13R,13aR,13bS)-10-hydroxy-2,2,6a,6b,9,9,12a-heptamethyl-15-oxooctadecahydro-13,4a-(epoxymethano)dibenzo[a,i]fluorene-13a(2H)-carbaldehyde (2)

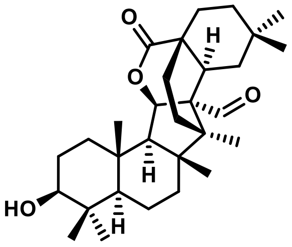

Chemical Formula: $C_{30}H_{46}O_4$
Exact Mass: 470,3396

In a pressure vial was charged dialdehyde **7** (40 mg, 0.08 mmol, 1.0 eq.) and dissolved in dry toluene (1 mL) and DBU (1.66 mL, 11.1 mmol, 14.0 eq.). The mixture was degassed by sparging with argon for 10 minutes and then sealed and submerged in an oil-bath pre-heated to 175 °C. Reaction was heated at that temperature for 1 hour before allowing to cool to room temperature and partitioning between water (10 mL) and dichloromethane (3x 20 mL). Combined extracts were dried over $MgSO_4$ and evaporated to give crude product, which was purified by column chromatography (4 g SiO_2) using pentane/EtOAc/CH_2Cl_2 = 3/2/1 to give 21 mg (56%) of Alstoscholarinoid B (**2**) as a white solid.

m.p.: >300 °C; Lit.:[12] 333 – 334 °C

Optical rotation: $[\alpha]_D^{20}$= +17.0 (c 0.2, CH_2Cl_2); Lit.:[12] +18.2 (c 0.11, CH_2Cl_2)

^{1}H NMR (600 MHz, $CDCl_3$) δ = 10.02 (s, 1H), 5.12 (d, *J* = 2.6 Hz, 1H), 3.18 (dd, *J* = 10.7, 5.6 Hz, 1H), 2.19 (t, *J* = 8.7 Hz, 1H), 2.02 – 1.95 (m, 2H), 1.91 – 1.80 (m, 4H), 1.77 – 1.57 (m, 5H), 1.54 (m, 2H), 1.49 – 1.41 (m, 1H), 1.41 – 1.27 (m, 4H), 1.24 – 1.19 (m, 1H), 1.24 (s, 3H), 1.22 (s, 3H), 1.20 (s, 3H), 0.95 (s, 3H), 0.94 (s, 3H), 0.88 (d, *J* = 2.9 Hz, 1H), 0.86 (s, 3H), 0.80 (s, 3H), 0.63 (dd, *J* = 11.7, 3.2 Hz, 1H).

^{13}C NMR (151 MHz, $CDCl_3$) δ = 206.4, 177.5, 84.6, 79.0, 60.8, 57.5, 56.5, 48.8, 46.9, 38.9, 38.5, 38.2, 37.7, 37.6, 37.3, 37.2, 33.7, 33.3, 31.0, 30.4, 29.6, 28.2, 27.7, 27.4, 27.2, 24.1, 20.9, 19.9, 18.4, 15.2.

HR-MS (ESI): Calculated for $C_{30}H_{47}O_5$ $[M+H]^+$ 471.3469, found 471.3478.

NMR comparison with original isolation report[8] and synthesis[12,13,14]

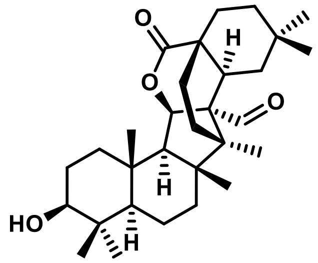

Atom	Natural **2**[8] δH [ppm]	Synthetic **2**[12]	Synthetic **2** - this work δH [ppm (Hz)]	ΔδH [ppm]
**1**	1.86, 1.19	1.86, 1.25	1.86, 1.21	+0.02
**2**	1.71	1.71 (6.8, 4.2 Hz)	1.71	0.00
**3**	3.18 (9.7, 6.5 Hz)	3.17 (9.5, 6.7 Hz)	3.18 (10.7, 5.6 Hz)	0.00
**5**	0.64 (11.1, 3.7 Hz)	0.63 (11.1, 3.8 Hz)	0.63 (11.7, 3.2 Hz)	-0.01
**6**	1.62, 1.57	1.62, 1.60	1.62, 1.58	+0.01
**7**	1.37, 1.31	1.37, 1.29	1.37, 1.30	-0.01
**9**	0.88 (2.4 Hz)	0.88 (2.7 Hz)	0.88 (2.9 Hz)	0.00
**11**	5.12 (2.4 Hz)	5.11 (2.6 Hz)	5.12 (2.6 Hz)	0.00
**12**	10.02	10.01	10.02	0.00
**15**	1.95, 1.83	1.94, 1.84	1.95, 1.83	0.00
**16**	1.99, 1.85	1.98 (5.6 Hz), 1.86	2.00, 1.85	+0.01
**18**	2.19 (9.1 Hz)	2.18 (8.7 Hz)	2.19 (8.7 Hz)	0.00
**19**	1.54 (9.1 Hz)	1.54	1.54	0.00
**21**	1.89, 1.65	1.89 (6.8, 4.0), 1.65 (4.0 Hz)	1.90, 1.66	+0.01
**22**	1.43, 1.35	1.41, 1.33	1.42, 1.34	-0.01
**23**	0.94	0.93	0.94	0.00
**24**	0.80	0.79	0.80	0.00
**25**	1.22	1.22	1.22	0.00
**26**	1.20	1.20	1.20	0.00
**27**	1.25	1.24	1.24	-0.01
**29**	0.86	0.86	0.86	0.00
**30**	0.95	0.95	0.95	0.00

NMR comparison with original isolation report[8] and synthesis[12,13,14]

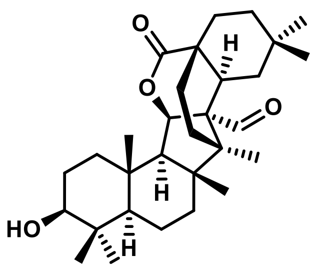

	Natural **2**[8]	Synth. **2**[12,13,14]	Synth. **2** - this work	$\Delta\delta_C$
Atom	δ_C [ppm]	δ_C [ppm]	δ_C [ppm]	[ppm]
**1**	38.0	38.1	38.2	+0.1
**2**	27.0	27.1	27.2	+0.1
**3**	78.9	78.9	79.0	+0.1
**4**	38.7	38.8	38.9	+0.1
**5**	56.4	56.4	56.5	+0.1
**6**	19.8	19.8	19.9	+0.1
**7**	38.4	38.4	38.5	+0.1
**8**	48.7	48.7	48.8	+0.1
**9**	60.7	60.7	60.8	+0.1
**10**	29.5	29.5	29.6	+0.1
**11**	84.4	84.5	84.6	+0.1
**12**	206.3	206.2	206.4	+0.1
**13**	57.3	57.4	57.5	+0.1
**14**	46.8	46.8	46.9	+0.1
**15**	30.8	30.8	31.0	+0.2
**16**	27.2	27.2	27.4	+0.2
**17**	37.4	37.5	37.7	+0.2
**18**	37.6	37.6	37.6	0.0
**19**	37.0	37.1	37.2	+0.1
**20**	37.2	37.2	37.3	+0.1
**21**	30.2	30.3	30.4	+0.1
**22**	33.6	33.6	33.7	+0.1
**23**	28.1	28.1	28.2	+0.1
**24**	15.1	15.1	15.2	+0.1
**25**	18.2	18.2	18.4	+0.2
**26**	20.8	20.8	20.9	+0.1
**27**	27.5	27.5	27.7	+0.2
**28**	177.4	177.4	177.5	+0.1
**29**	24.0	24.0	24.1	+0.1
**30**	33.2	33.2	33.3	+0.1

^{1}H-NMR from natural source reproduced from [8] Copyright © 2021 American Chemical Society.

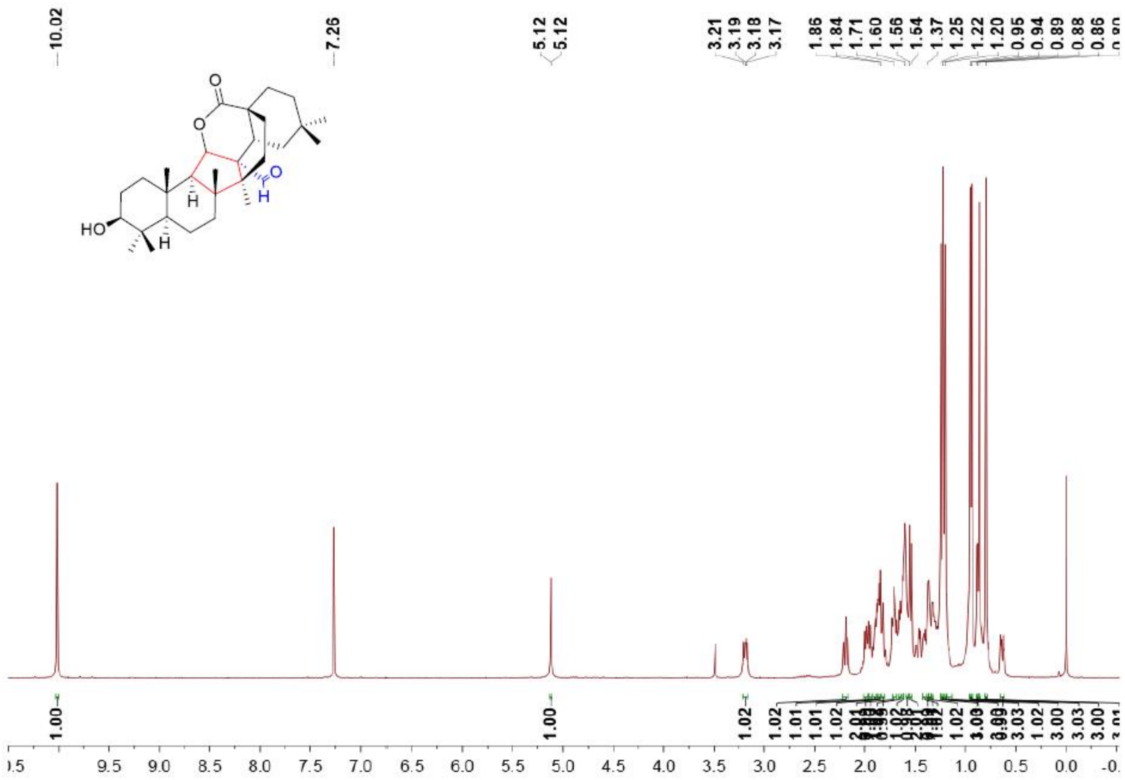

^{1}H-NMR of synthetic material:

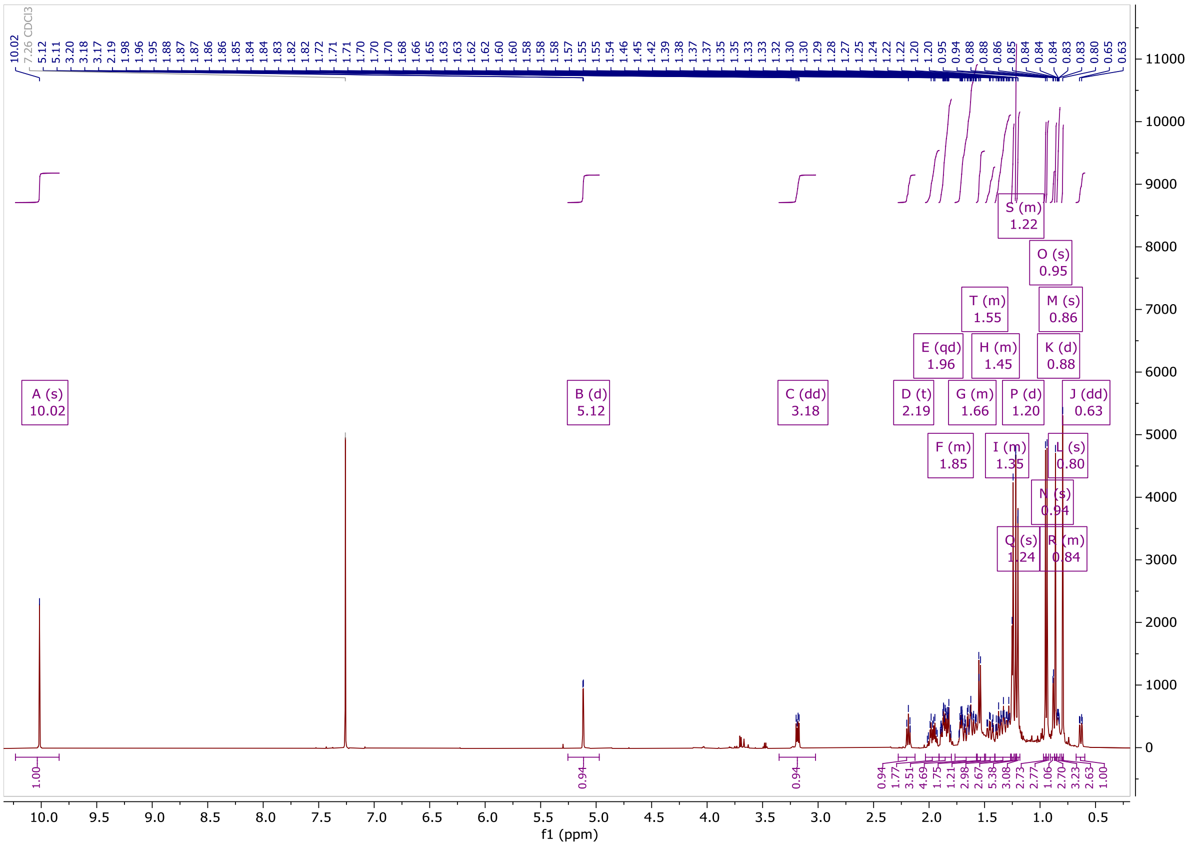

Spectra

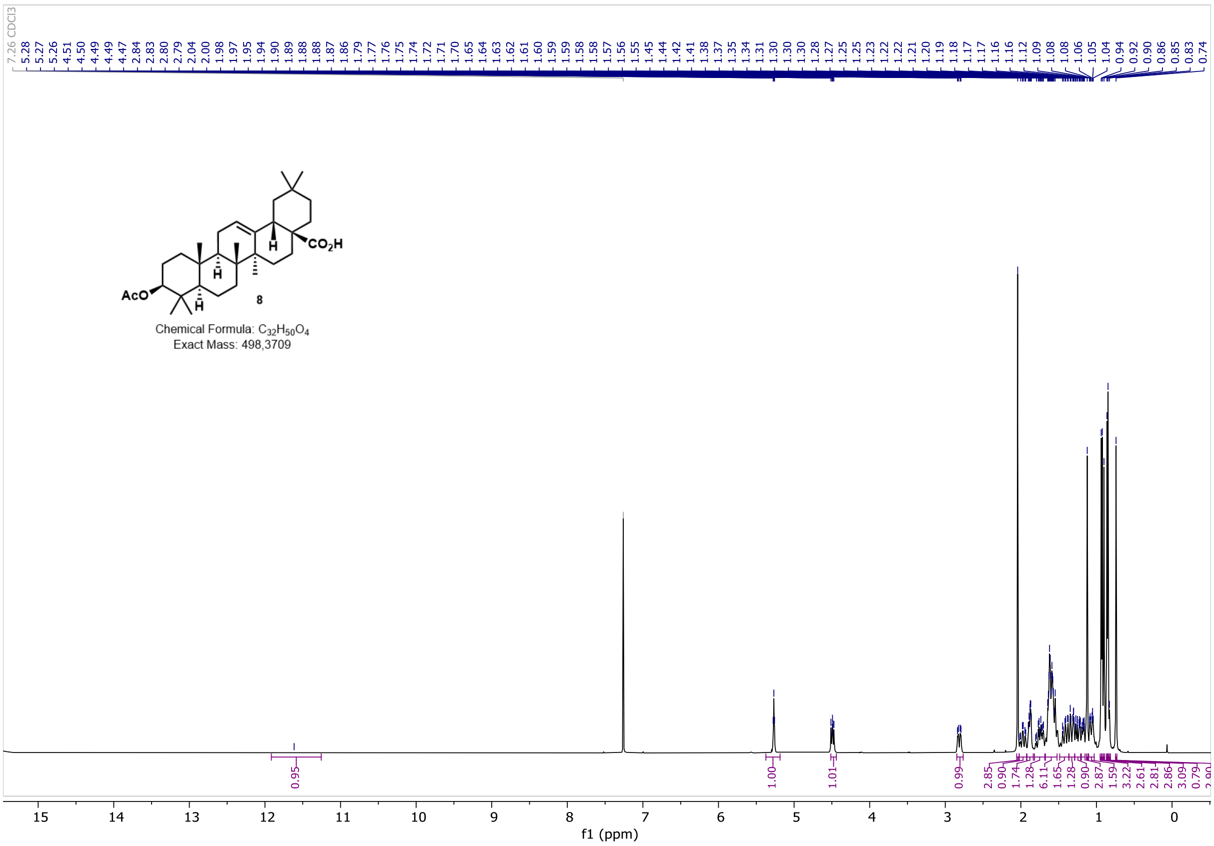

AcO
CO$_2$H
8
Chemical Formula: $C_{32}H_{50}O_4$
Exact Mass: 498,3709
f1 (ppm)

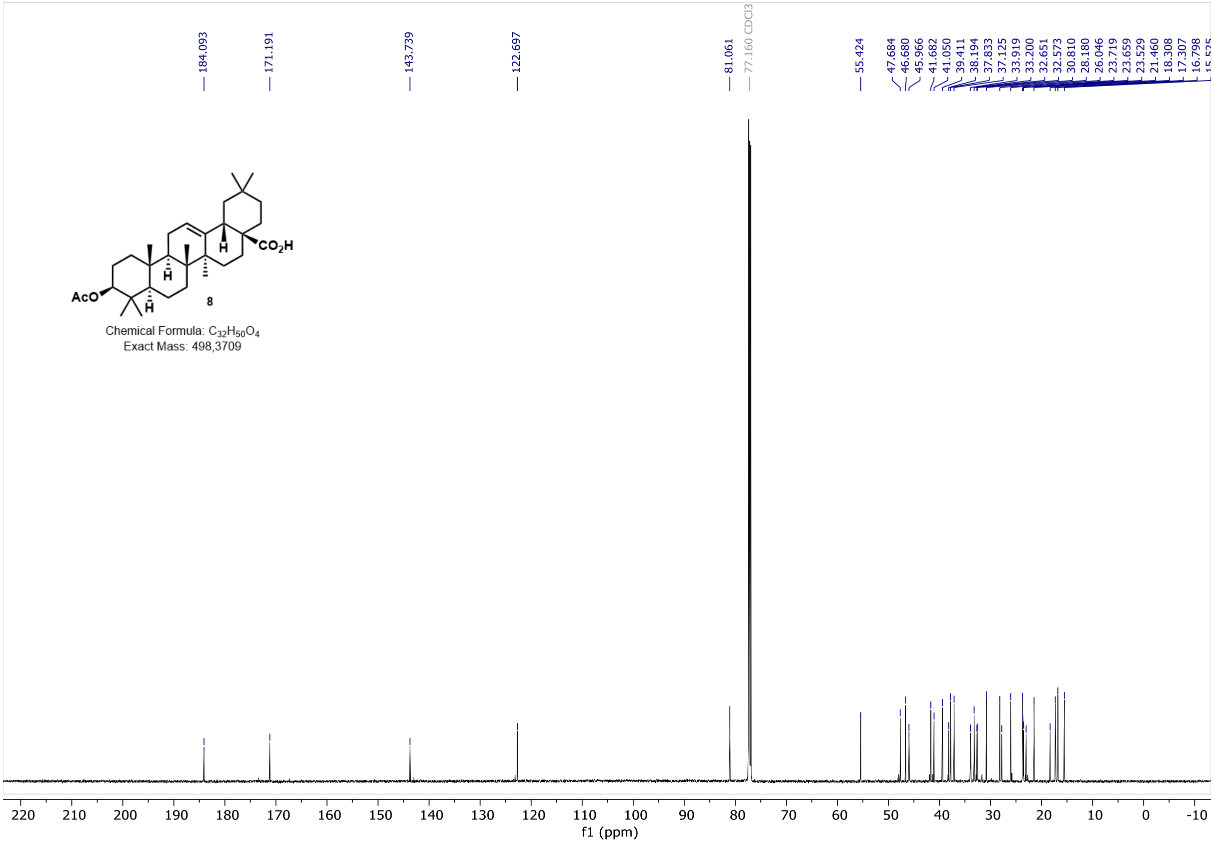

AcO
CO$_2$H
8
Chemical Formula: $C_{32}H_{50}O_4$
Exact Mass: 498,3709
184.093
171.191
143.739
122.697
81.061
77.160 CDCl3
55.424
f1 (ppm)

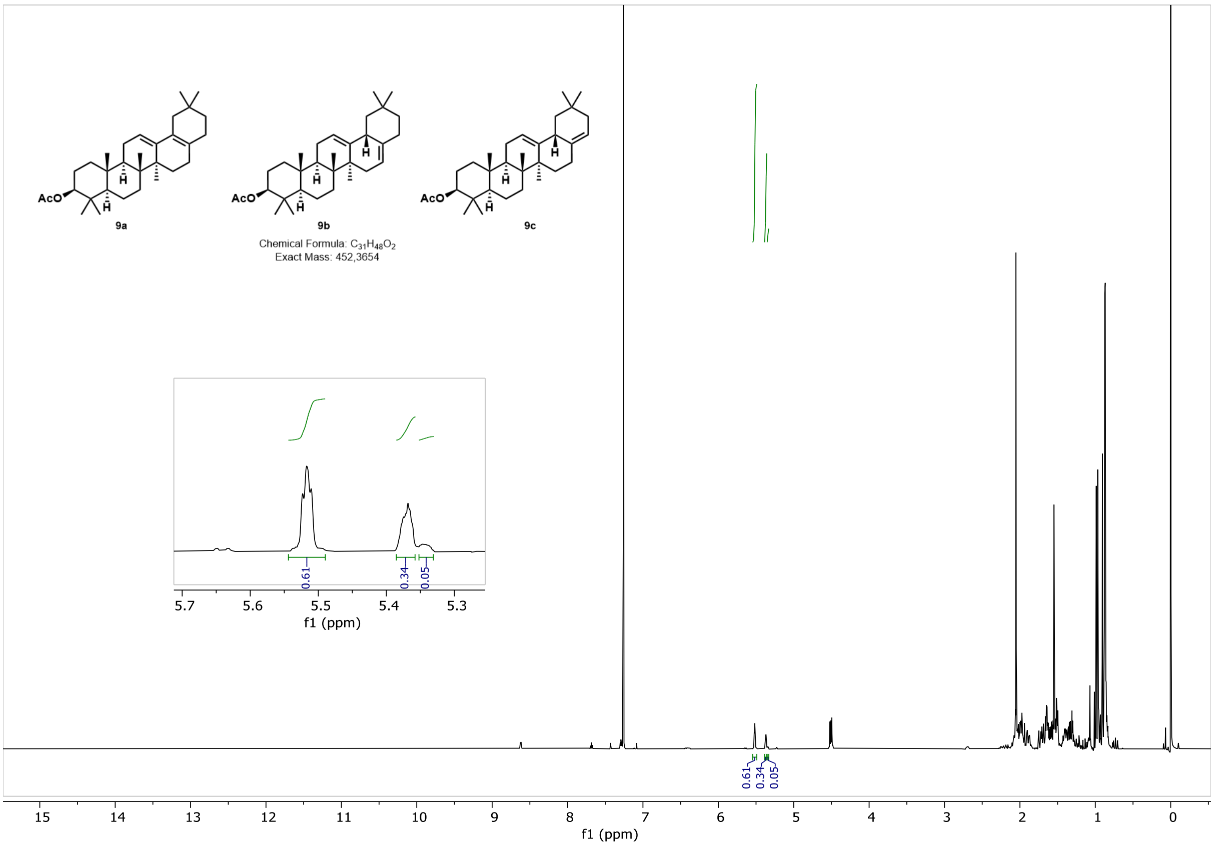
AcO
9a
9b
9c
Chemical Formula: $C_{31}H_{48}O_2$
Exact Mass: 452,3654
0.61
0.34
0.05
f1 (ppm)

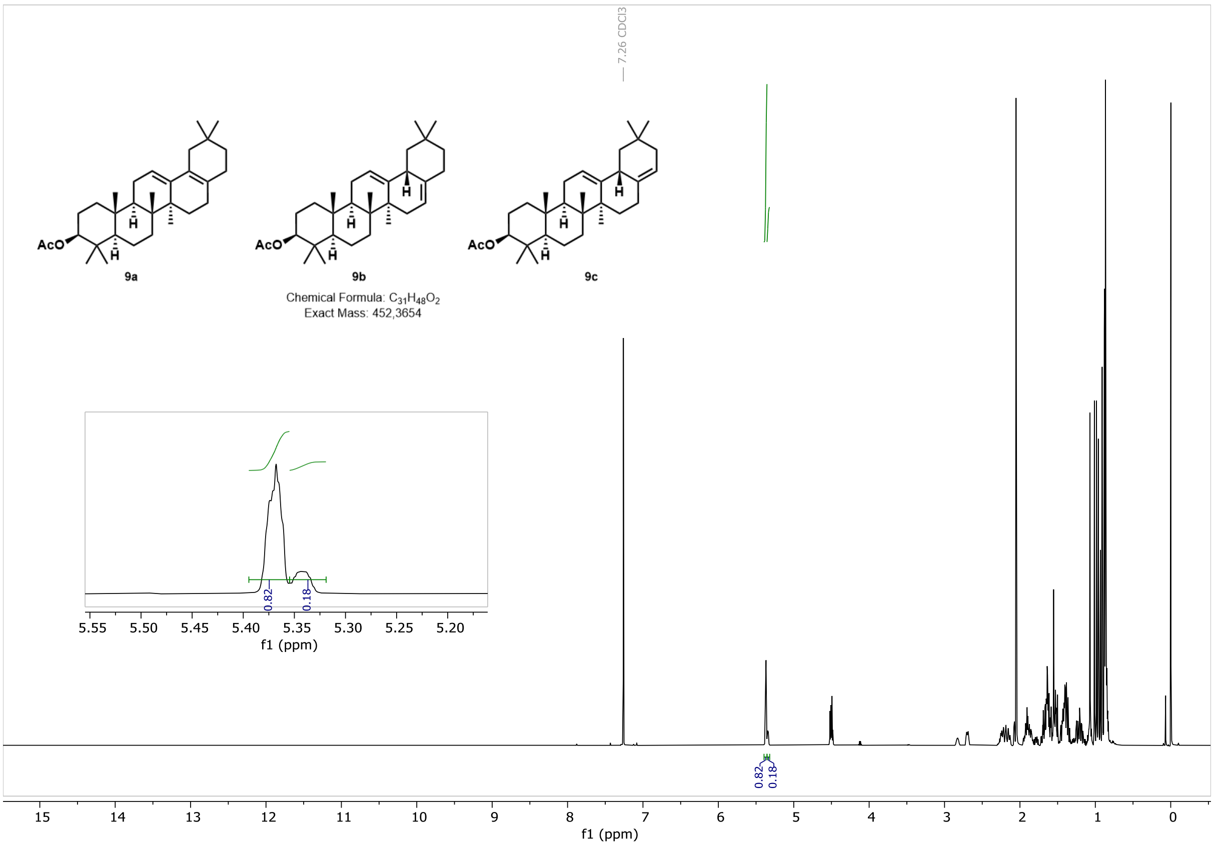
7.26 CDCl3
AcO
9a
9b
9c
Chemical Formula: $C_{31}H_{48}O_2$
Exact Mass: 452,3654
0.82
0.18
f1 (ppm)

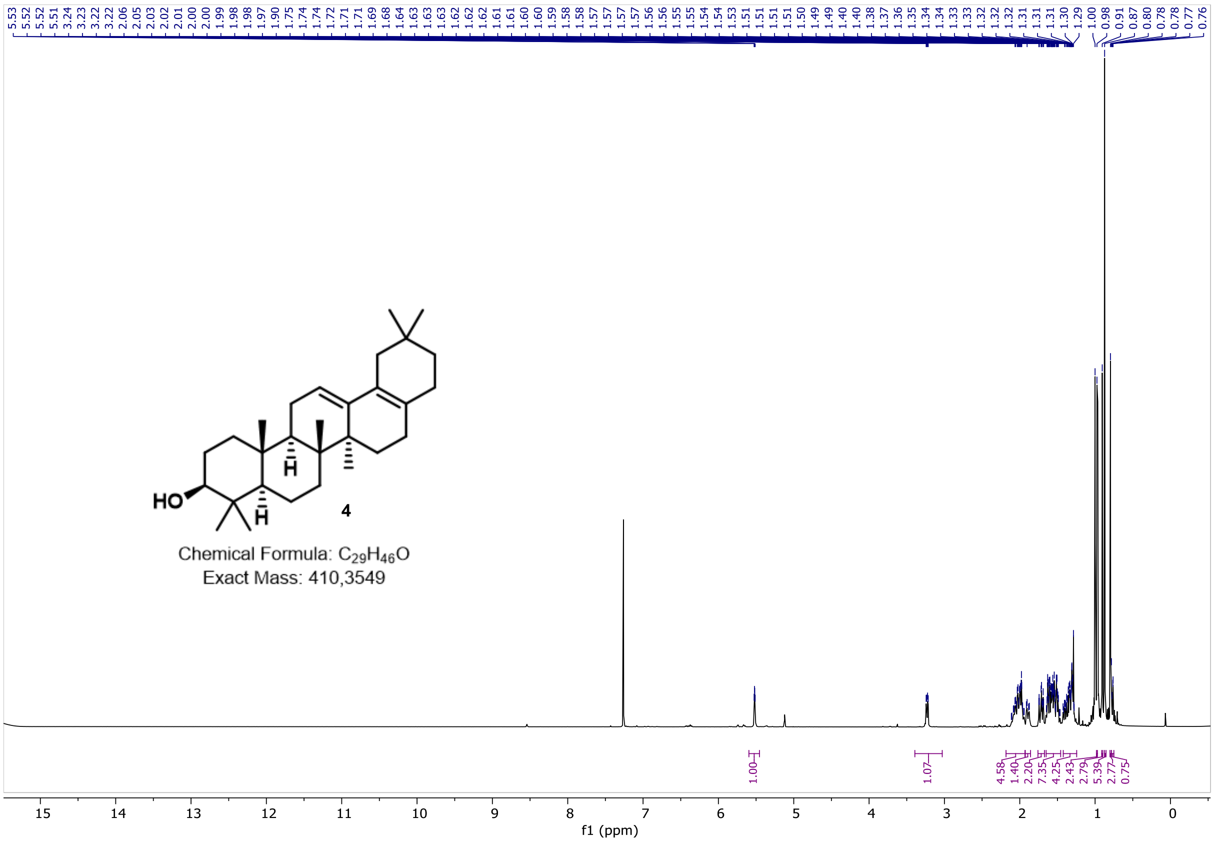
5.53
5.52
5.52
5.51
3.24
3.23
3.22
3.22
2.06
2.05
2.03
2.02
2.01
2.00
1.99
1.98
1.98
1.97
1.90
1.75
1.74
1.74
1.72
1.71
1.71
1.69
1.68
1.64
1.63
1.63
1.63
1.62
1.62
1.62
1.61
1.61
1.60
1.59
1.58
1.58
1.57
1.57
1.57
1.56
1.56
1.55
1.55
1.54
1.54
1.53
1.51
1.51
1.51
1.51
1.50
1.49
1.49
1.40
1.40
1.38
1.37
1.36
1.35
1.34
1.34
1.33
1.33
1.32
1.32
1.32
1.31
1.31
1.31
1.30
1.29
1.00
0.98
0.91
0.87
0.80
0.78
0.78
0.77
0.76
HO
H
H
4
Chemical Formula: C29H46O
Exact Mass: 410,3549
1.00
1.07
4.58
1.40
2.20
7.35
4.25
2.43
2.79
5.39
2.77
0.75
15 14 13 12 11 10 9 8 7 6 5 4 3 2 1 0
f1 (ppm)

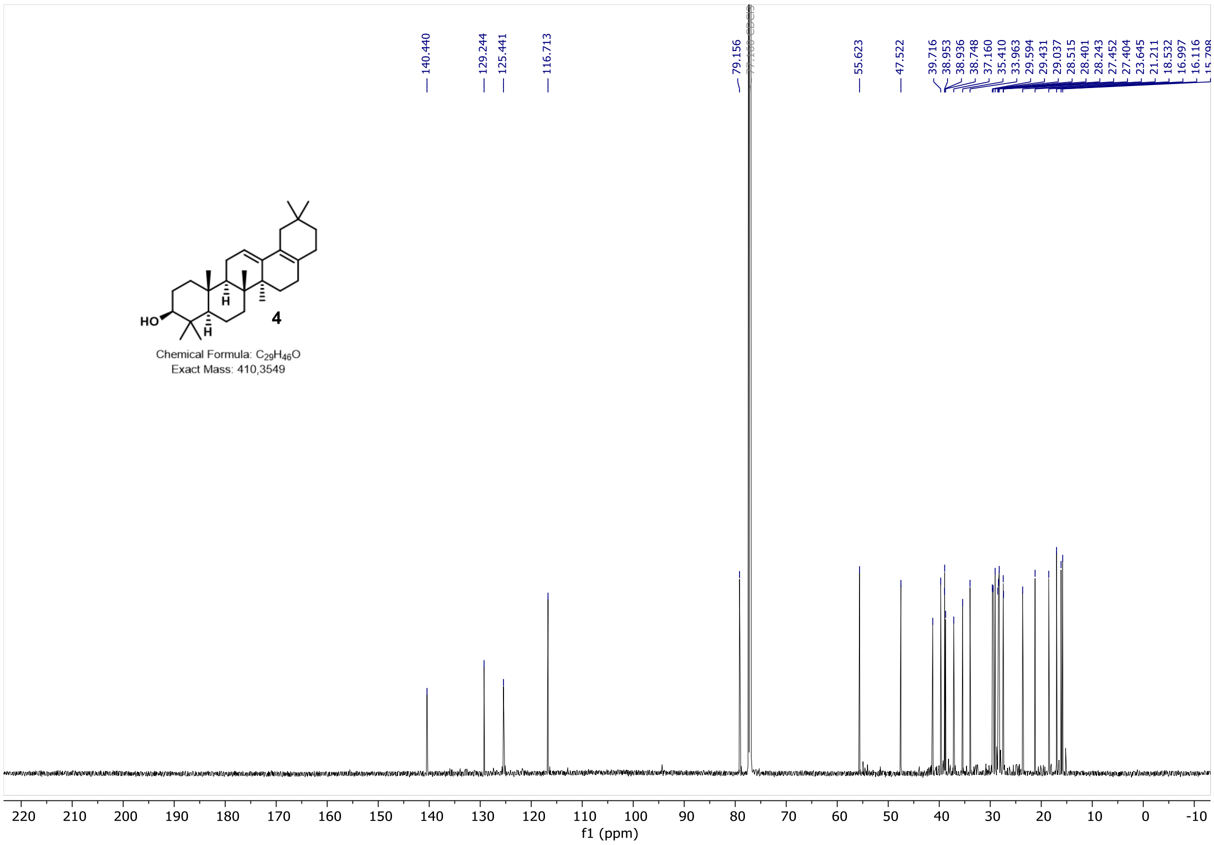
140.440
129.244
125.441
116.713
79.156
55.623
47.522
39.716
38.953
38.936
38.748
37.160
35.410
33.963
29.594
29.431
29.037
28.515
28.401
28.243
27.452
27.404
23.645
21.211
18.532
16.997
16.116
HO
H
H
4
Chemical Formula: C29H46O
Exact Mass: 410,3549
220 210 200 190 180 170 160 150 140 130 120 110 100 90 80 70 60 50 40 30 20 10 0 -10
f1 (ppm)

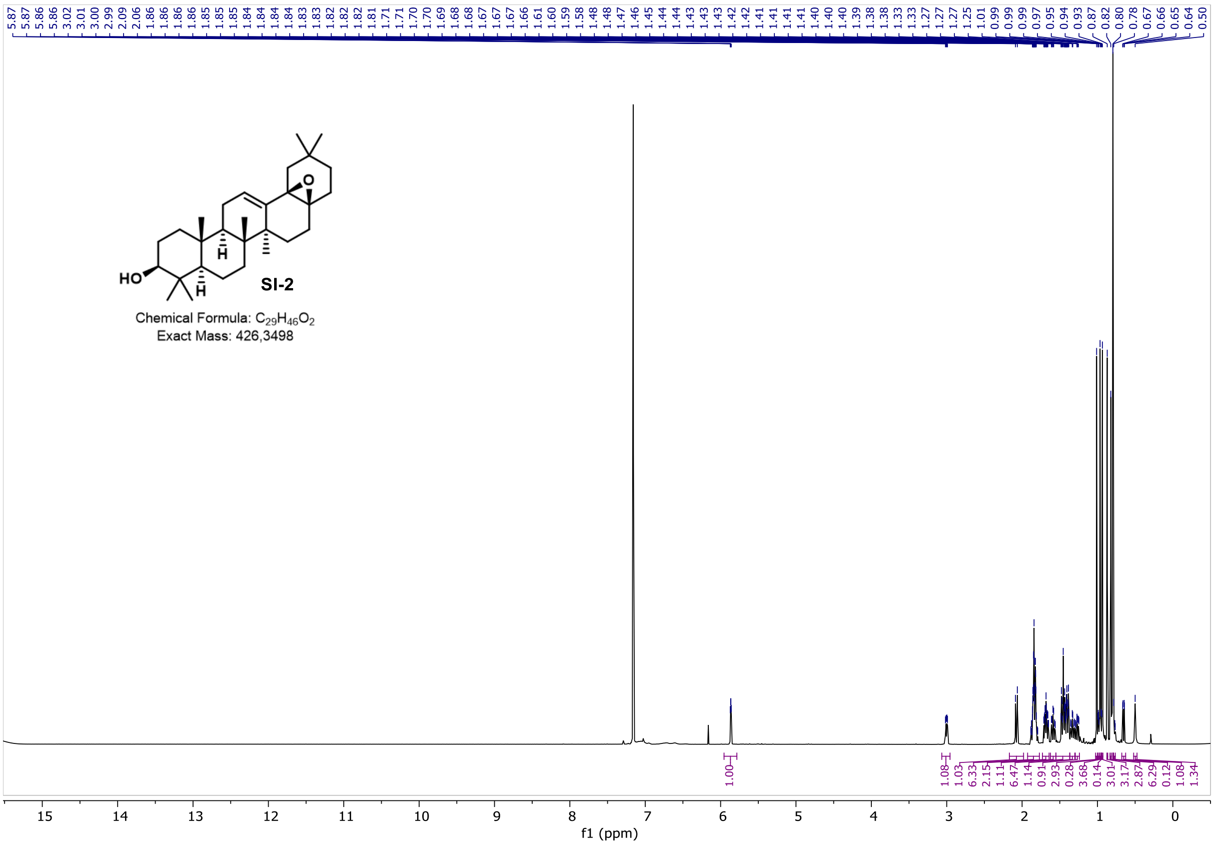
HO
H
H
SI-2
Chemical Formula: C29H46O2
Exact Mass: 426,3498
f1 (ppm)

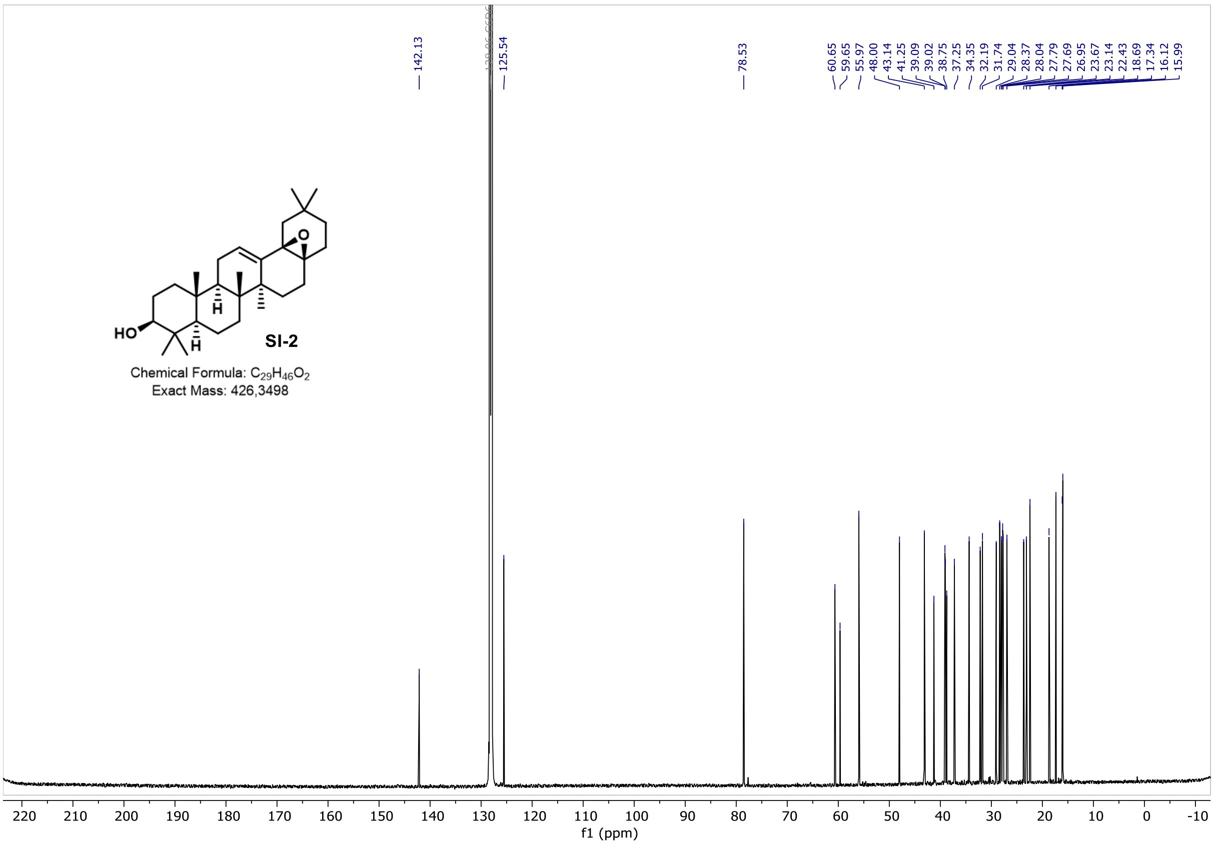
142.13
125.54
78.53
60.65
59.65
55.97
48.00
43.14
41.25
39.09
39.02
38.75
37.25
34.35
32.19
31.74
29.04
28.37
28.04
27.79
27.69
26.95
23.67
23.14
22.43
18.69
17.34
16.12
15.99
HO
H
H
SI-2
Chemical Formula: C29H46O2
Exact Mass: 426,3498
f1 (ppm)

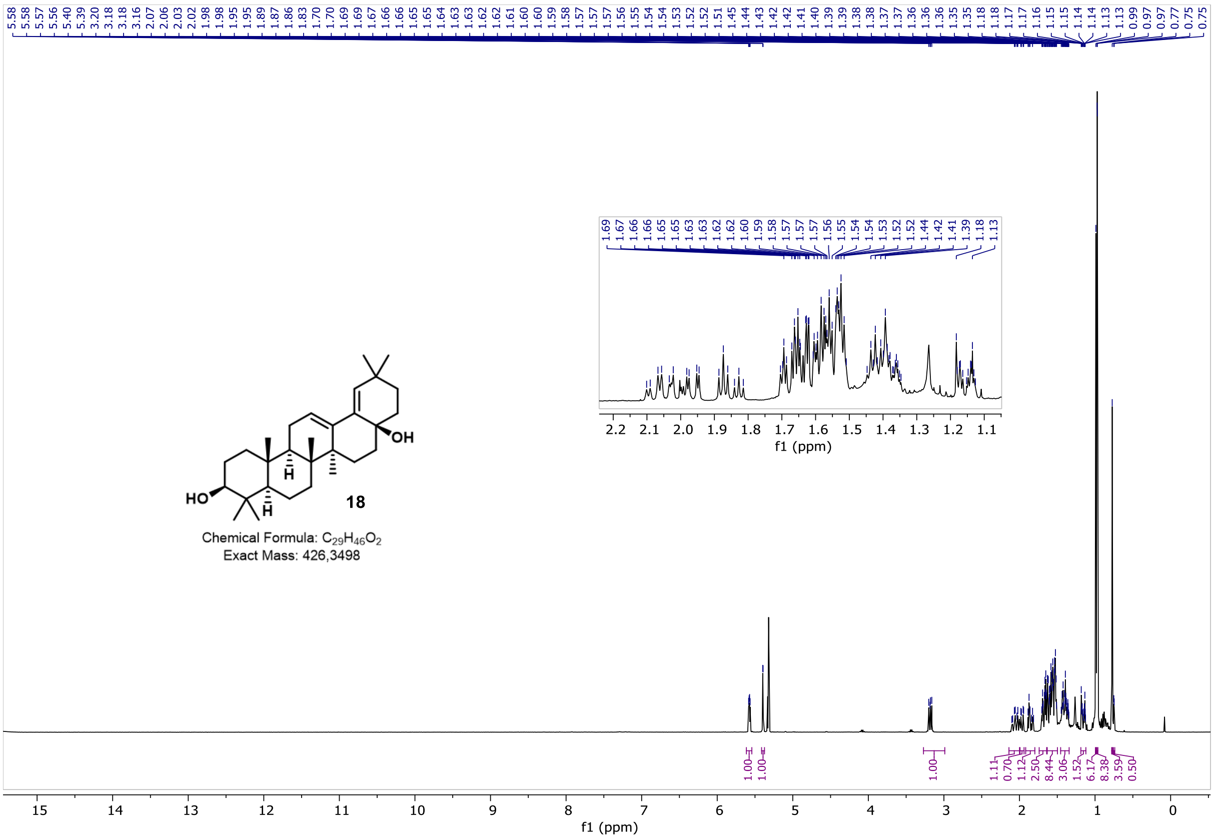
HO
OH
18
Chemical Formula: $C_{29}H_{46}O_2$
Exact Mass: 426,3498
f1 (ppm)
f1 (ppm)

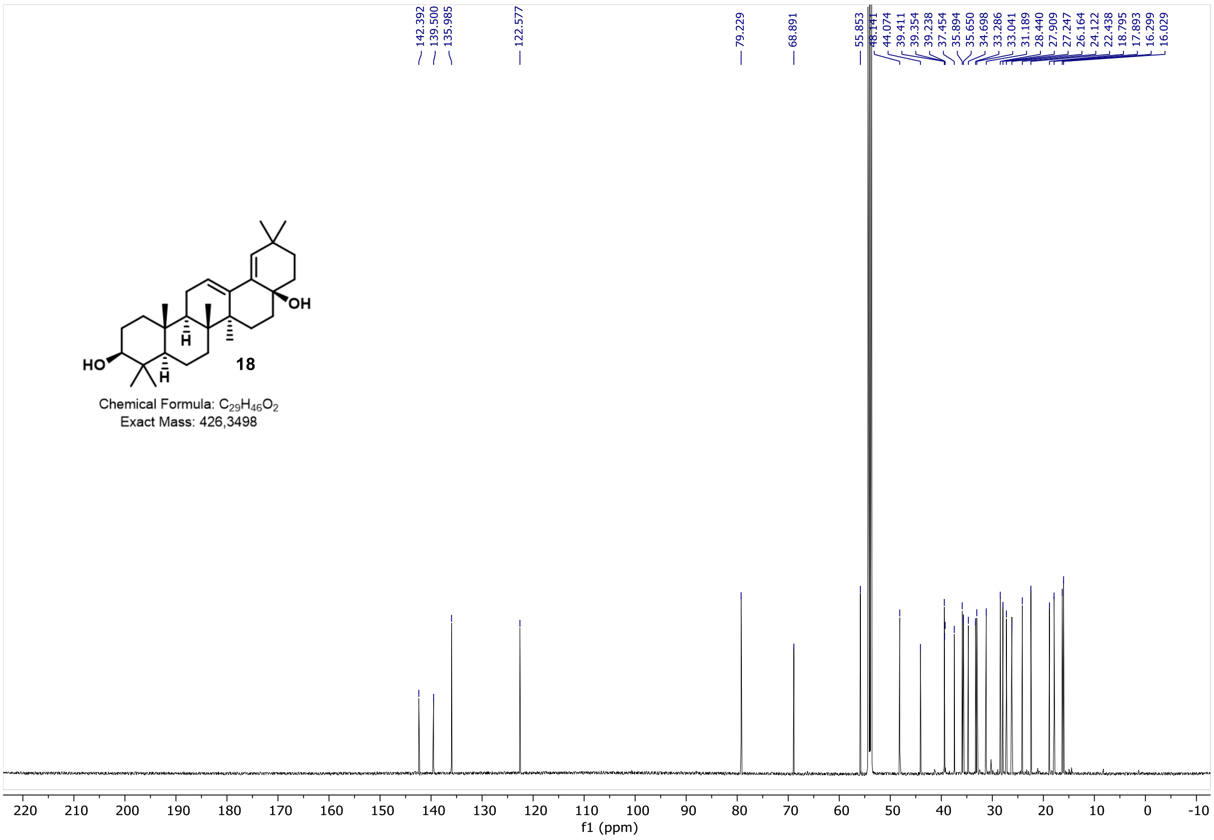
142.392
139.500
135.985
122.577
79.229
68.891
55.853
48.141
44.074
39.411
39.354
39.238
37.454
35.894
35.650
34.698
33.286
33.041
31.189
28.440
27.909
27.247
26.164
24.122
22.438
18.795
17.893
16.299
16.029
HO
OH
18
Chemical Formula: $C_{29}H_{46}O_2$
Exact Mass: 426,3498
f1 (ppm)

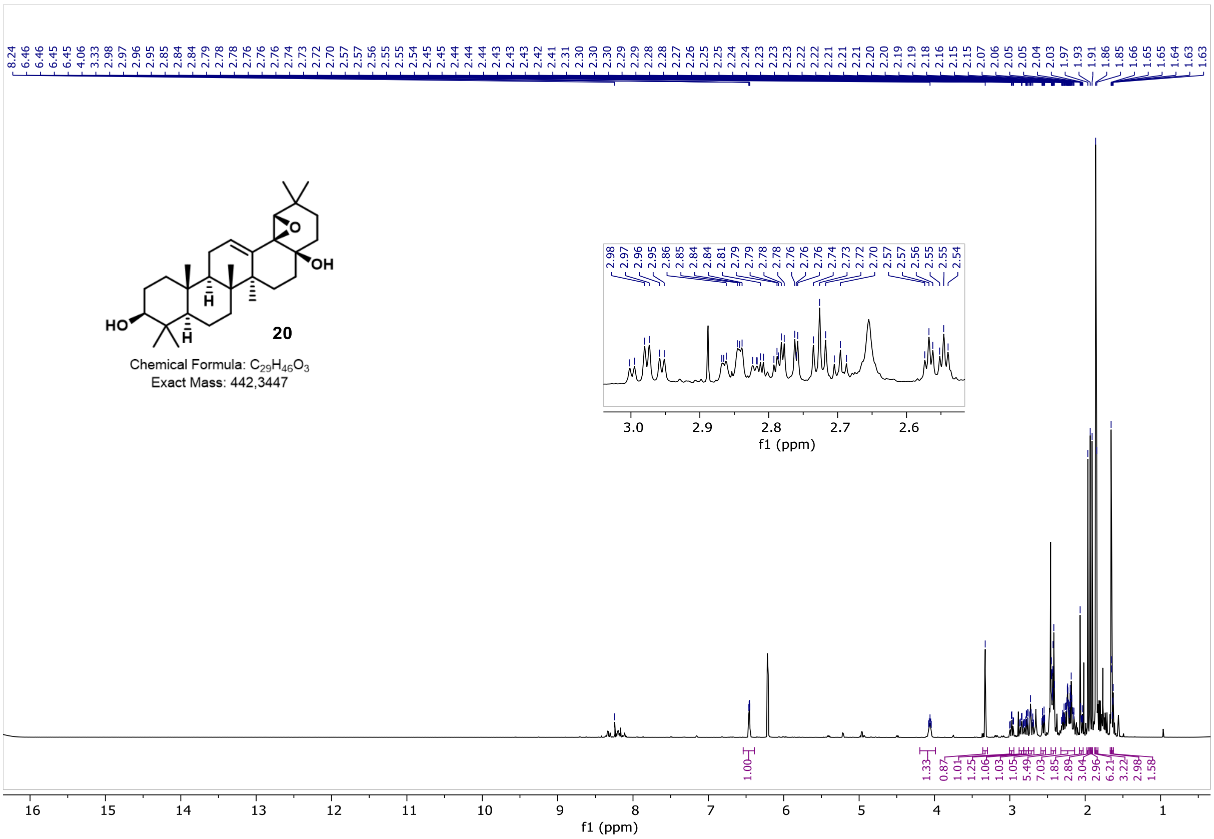
HO
OH
O
H
H
20
Chemical Formula: $C_{29}H_{46}O_3$
Exact Mass: 442,3447
f1 (ppm)
f1 (ppm)

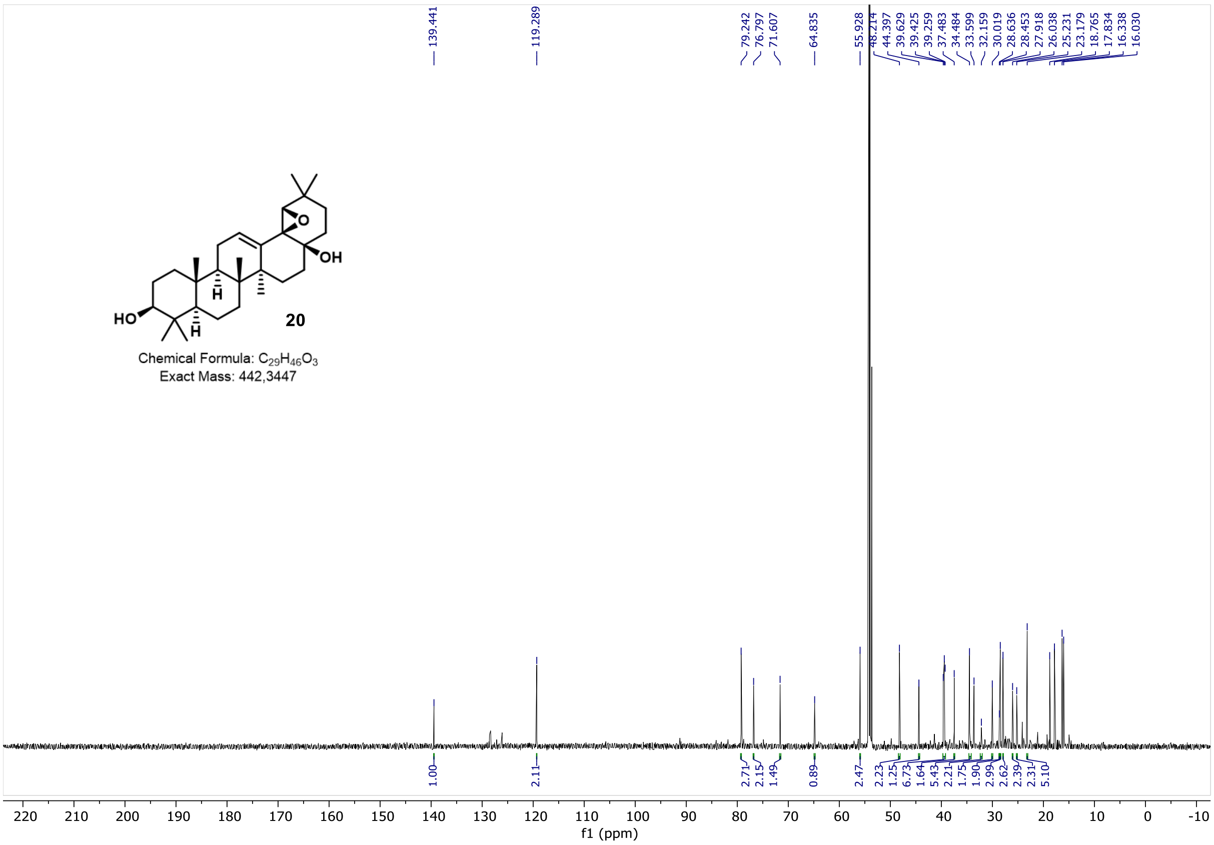
139.441
119.289
79.242
76.797
71.607
64.835
55.928
48.214
44.397
39.629
39.425
39.259
37.483
34.484
33.599
32.159
30.019
28.636
28.453
27.918
26.038
25.231
23.179
18.765
17.834
16.338
16.030
HO
OH
O
H
H
20
Chemical Formula: $C_{29}H_{46}O_3$
Exact Mass: 442,3447
f1 (ppm)

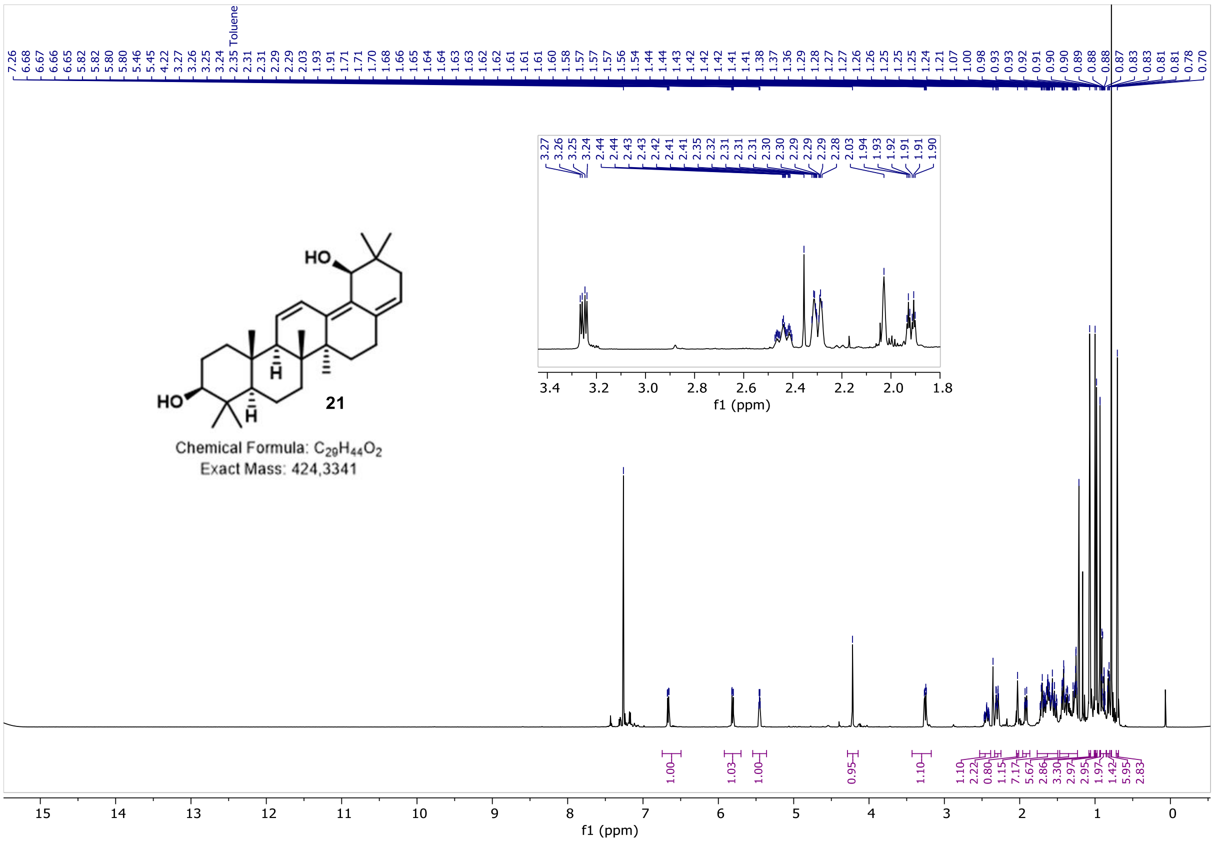

Toluene
HO
HO
H
H
21
Chemical Formula: $C_{29}H_{44}O_2$
Exact Mass: 424,3341
f1 (ppm)

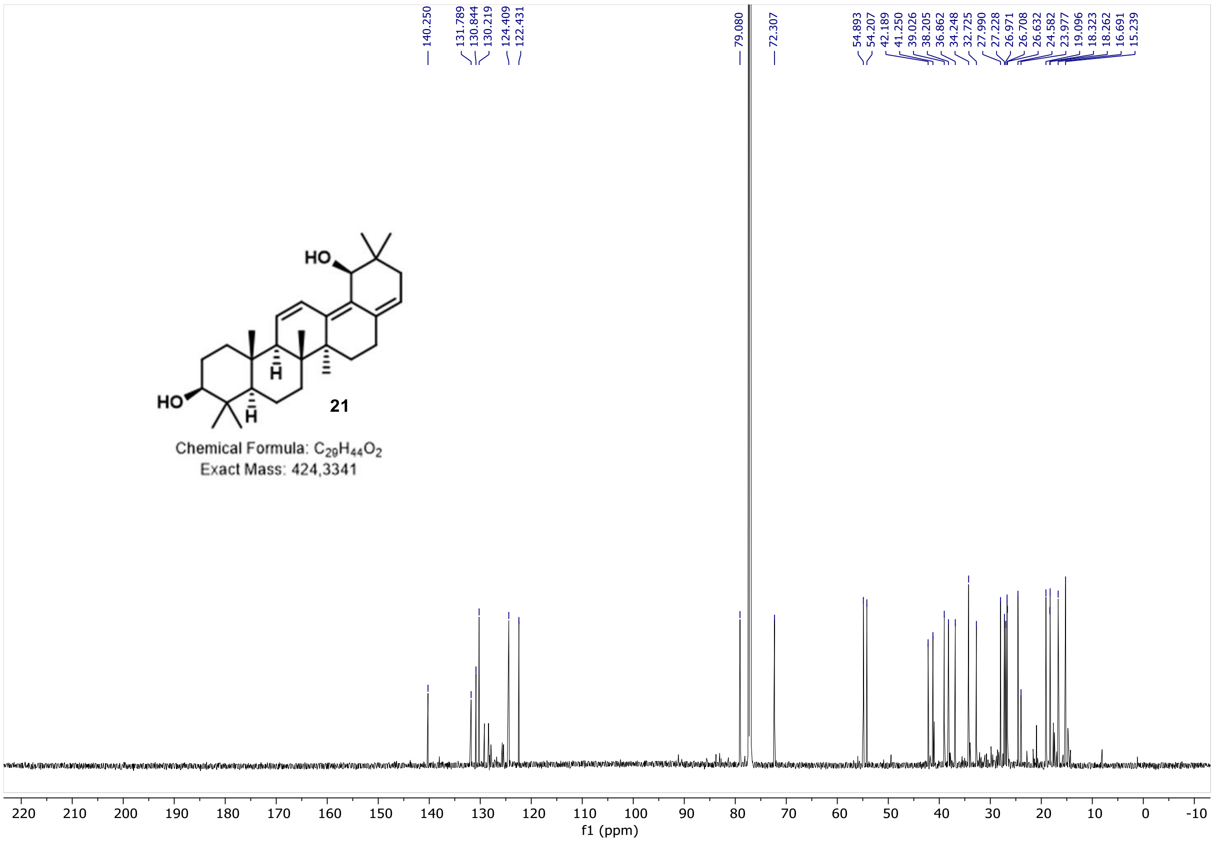

140.250
131.789
130.844
130.219
124.409
122.431
79.080
72.307
54.893
54.207
42.189
41.250
39.026
38.205
36.862
34.248
32.725
27.990
27.228
26.971
26.708
26.632
24.582
23.977
19.096
18.323
18.262
16.691
15.239
HO
HO
H
H
21
Chemical Formula: $C_{29}H_{44}O_2$
Exact Mass: 424,3341
f1 (ppm)

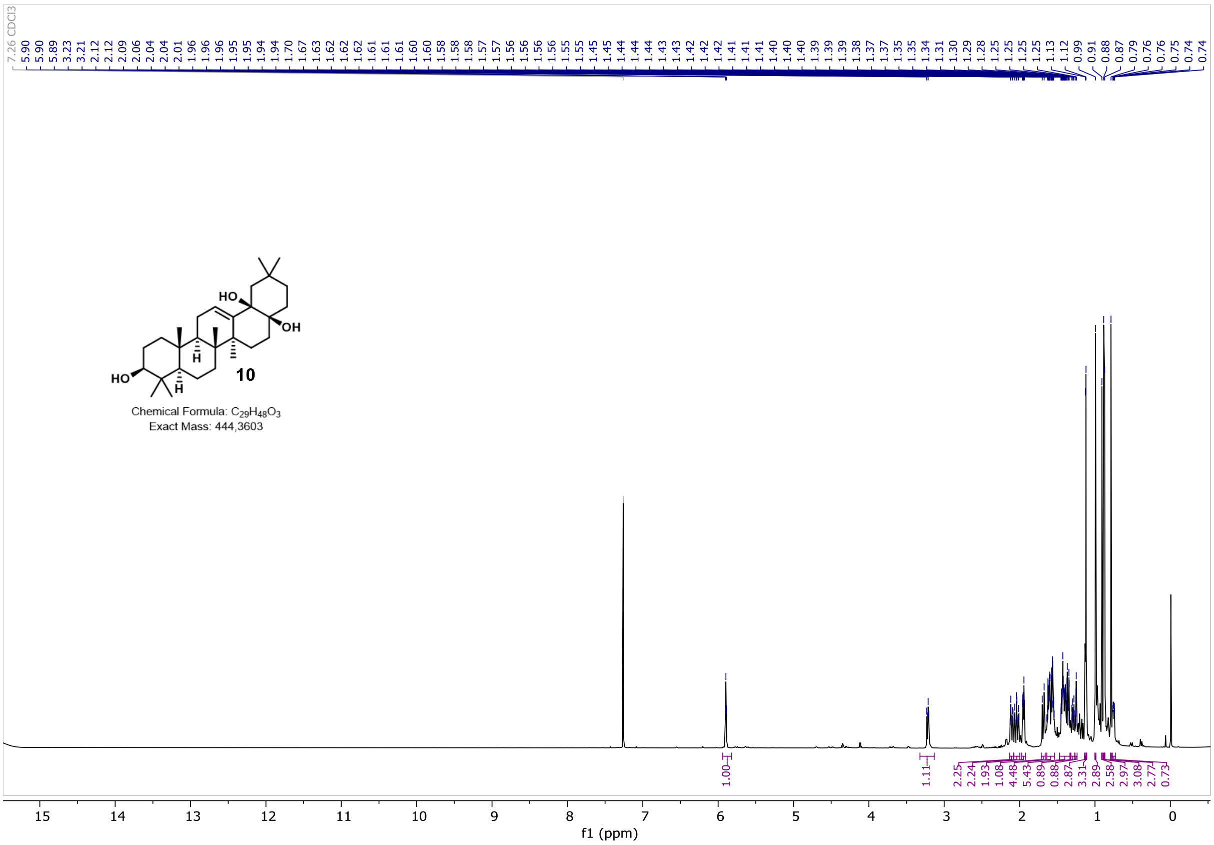

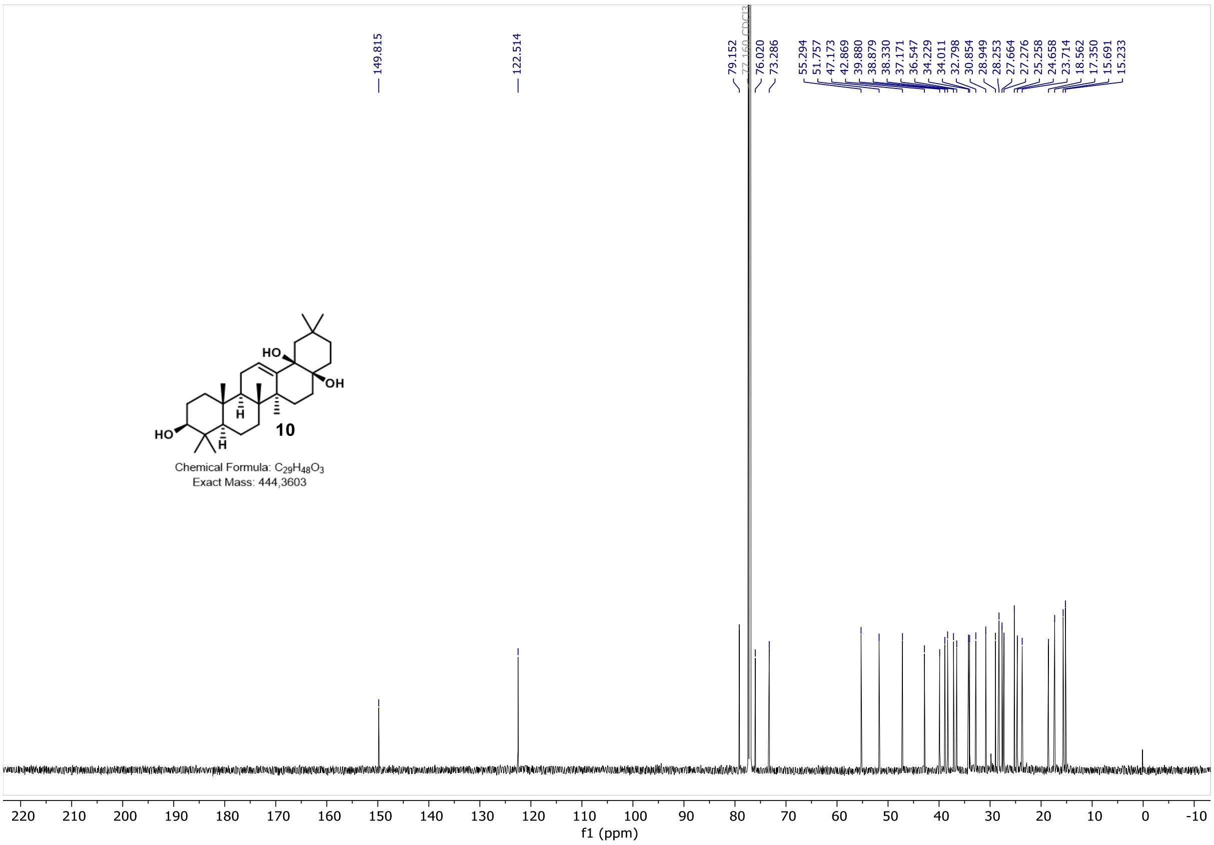

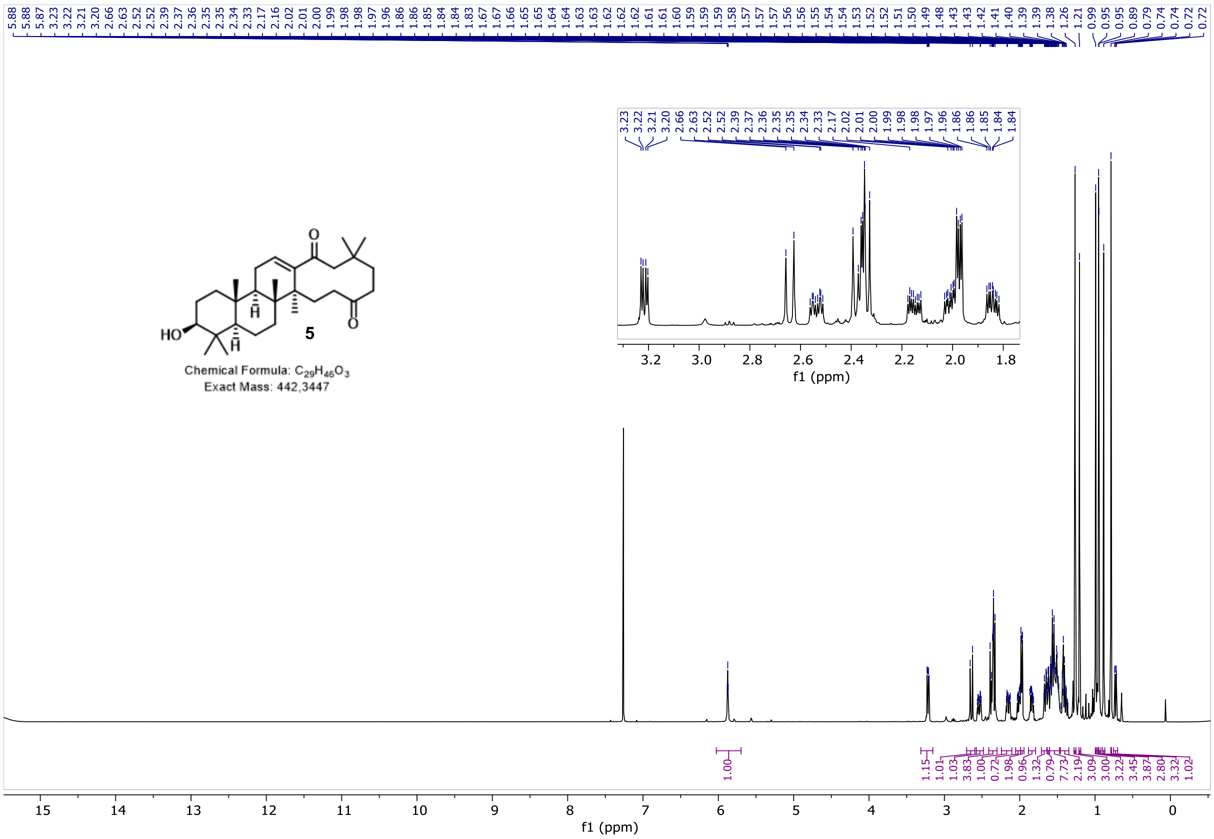
5
Chemical Formula: $C_{29}H_{46}O_3$
Exact Mass: 442,3447
f1 (ppm)

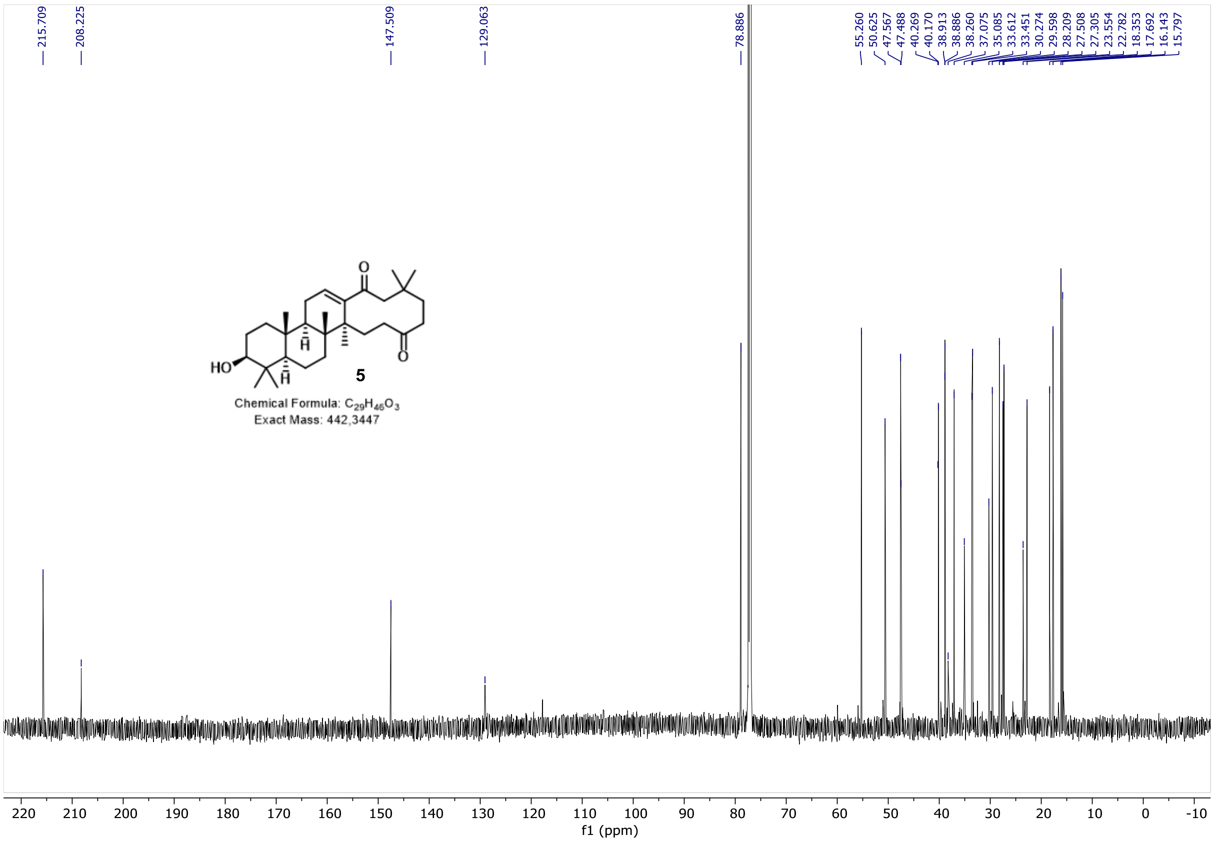
5
Chemical Formula: $C_{29}H_{46}O_3$
Exact Mass: 442,3447
f1 (ppm)

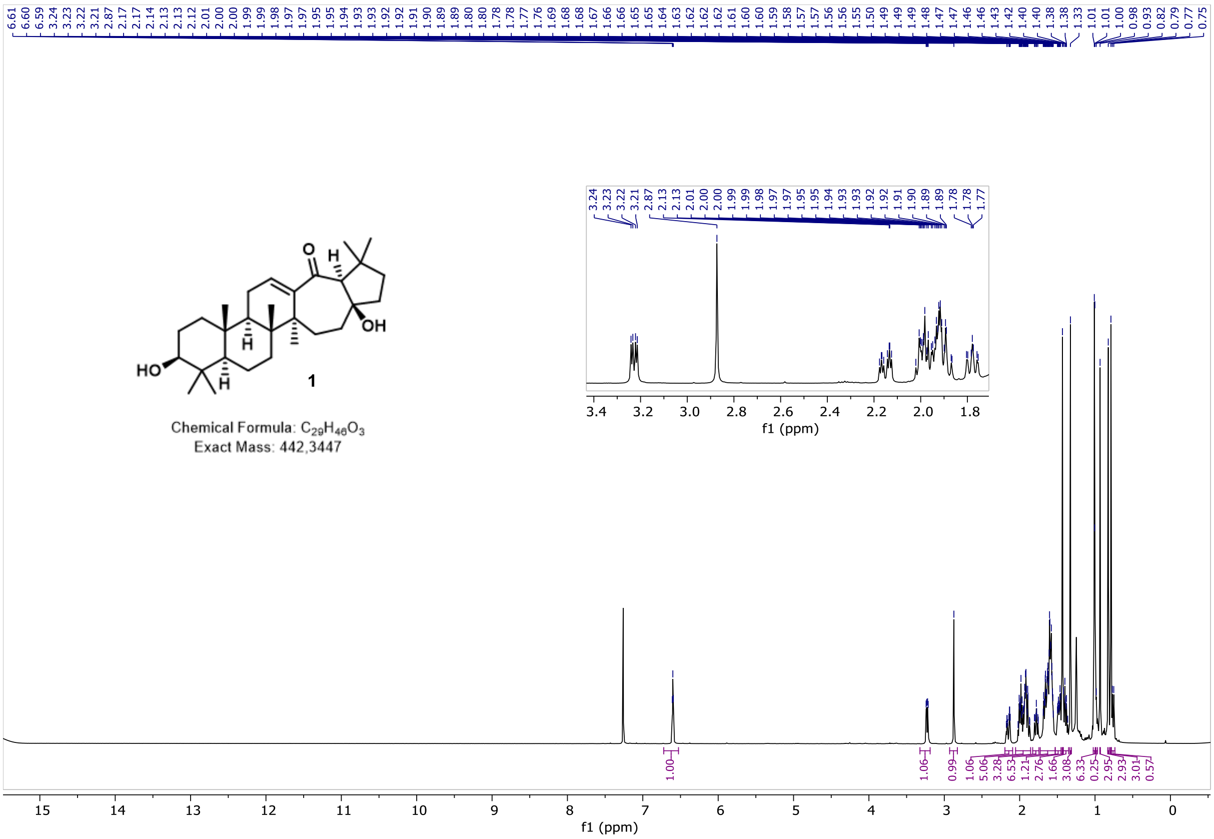
6.61 6.60 6.59 3.24 3.23 3.22 3.21 2.87 2.17 2.17 2.14 2.13 2.13 2.12 2.01 2.00 2.00 1.99 1.99 1.98 1.97 1.97 1.95 1.95 1.94 1.93 1.93 1.92 1.92 1.91 1.90 1.89 1.89 1.80 1.78 1.78 1.77 1.76 1.69 1.68 1.68 1.67 1.66 1.66 1.65 1.65 1.64 1.63 1.62 1.62 1.61 1.60 1.60 1.59 1.58 1.57 1.57 1.56 1.56 1.55 1.50 1.49 1.49 1.49 1.48 1.47 1.46 1.46 1.43 1.42 1.40 1.40 1.38 1.38 1.33 1.01 1.01 1.00 0.98 0.93 0.82 0.79 0.77 0.75
1
Chemical Formula: $C_{29}H_{46}O_3$
Exact Mass: 442,3447
f1 (ppm)

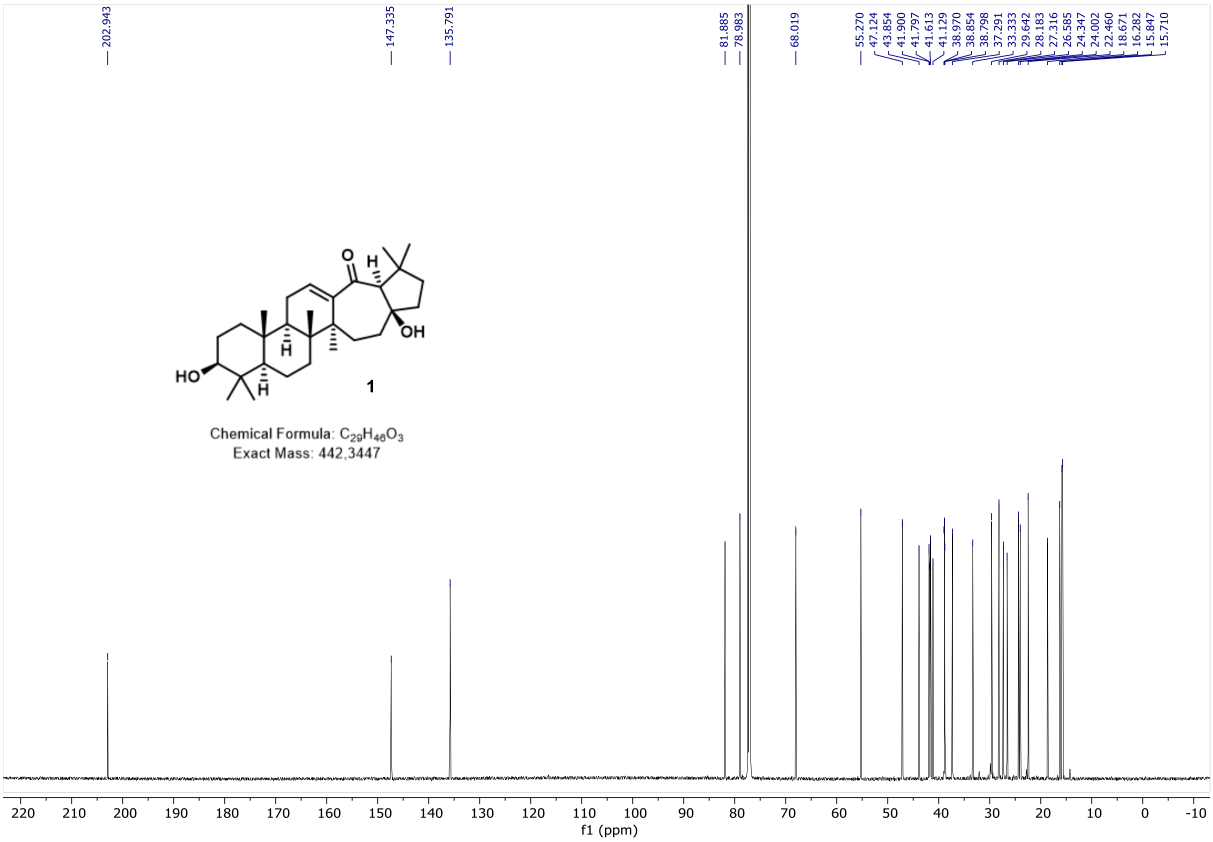
202.943 147.335 135.791 81.885 78.983 68.019 55.270 47.124 43.854 41.900 41.797 41.613 41.129 38.970 38.854 38.798 37.291 33.333 29.642 28.183 27.316 26.585 24.347 24.002 22.460 18.671 16.282 15.847 15.710
1
Chemical Formula: $C_{29}H_{46}O_3$
Exact Mass: 442,3447
f1 (ppm)

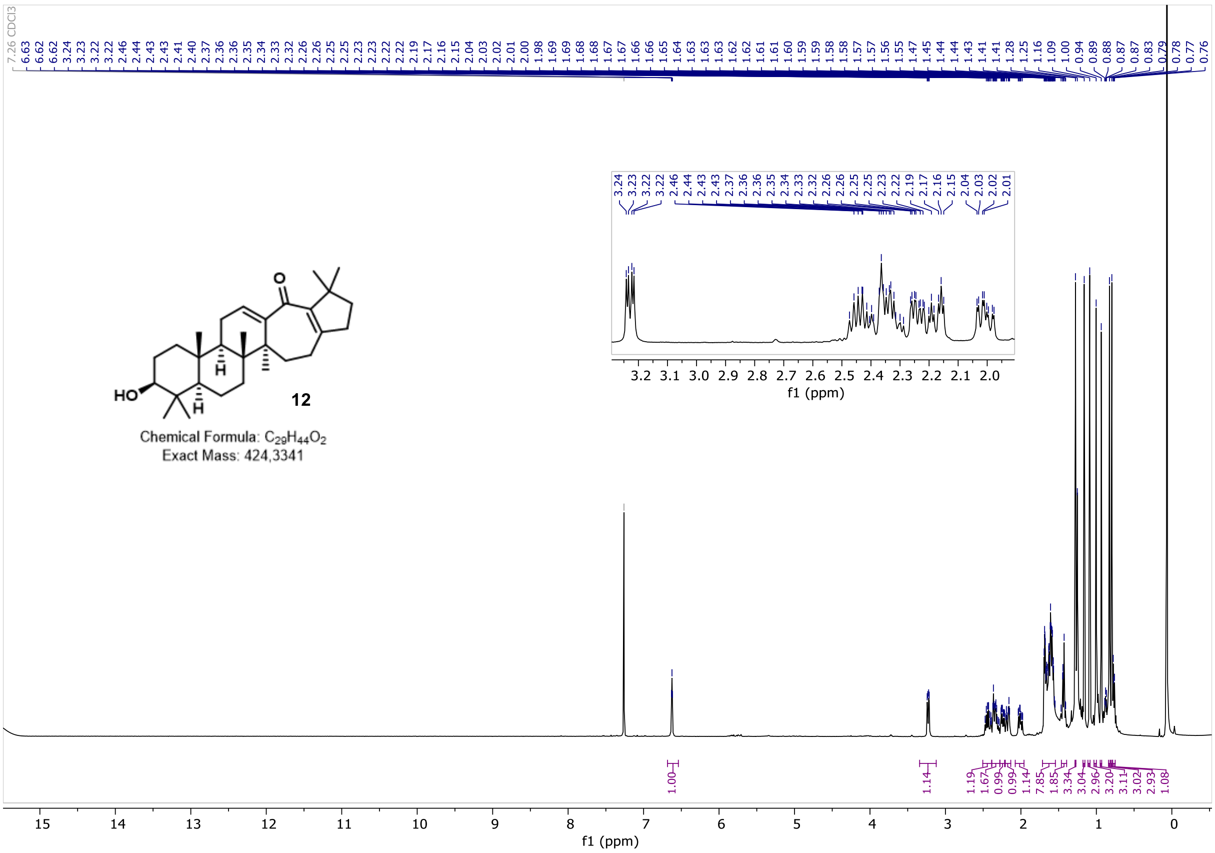
7.26 CDCl3
6.63 6.62 6.62 3.24 3.23 3.22 3.22 2.46 2.44 2.43 2.43 2.41 2.40 2.37 2.36 2.36 2.35 2.34 2.33 2.32 2.32 2.26 2.26 2.25 2.25 2.23 2.23 2.22 2.22 2.19 2.17 2.16 2.15 2.04 2.03 2.02 2.01 2.00 1.98 1.69 1.69 1.69 1.68 1.68 1.67 1.67 1.66 1.66 1.65 1.64 1.63 1.63 1.63 1.62 1.62 1.61 1.61 1.60 1.59 1.59 1.58 1.58 1.57 1.57 1.56 1.55 1.47 1.45 1.44 1.44 1.43 1.41 1.41 1.28 1.25 1.16 1.09 1.00 0.94 0.89 0.88 0.87 0.87 0.83 0.79 0.78 0.77 0.76
HO
12
Chemical Formula: $C_{29}H_{44}O_2$
Exact Mass: 424,3341
1.00 1.14 1.19 1.67 0.99 0.99 1.14 7.85 1.85 3.34 3.04 2.96 3.20 3.11 3.02 2.93 1.08
f1 (ppm)

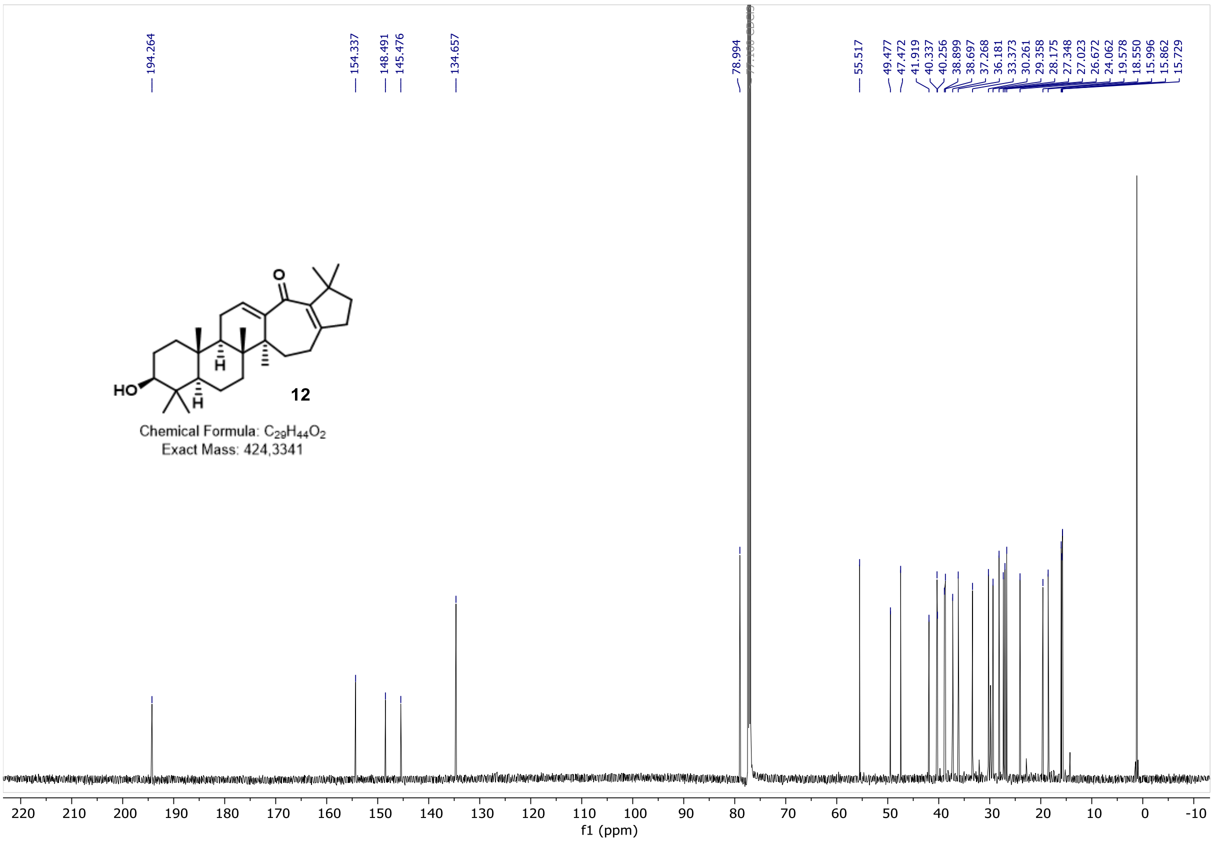
194.264
154.337 148.491 145.476 134.657
78.994
55.517 49.477 47.472 41.919 40.337 40.256 38.899 38.697 37.268 36.181 33.373 30.261 29.358 28.175 27.348 27.023 26.672 24.062 19.578 18.550 15.996 15.862 15.729
HO
12
Chemical Formula: $C_{29}H_{44}O_2$
Exact Mass: 424,3341
f1 (ppm)

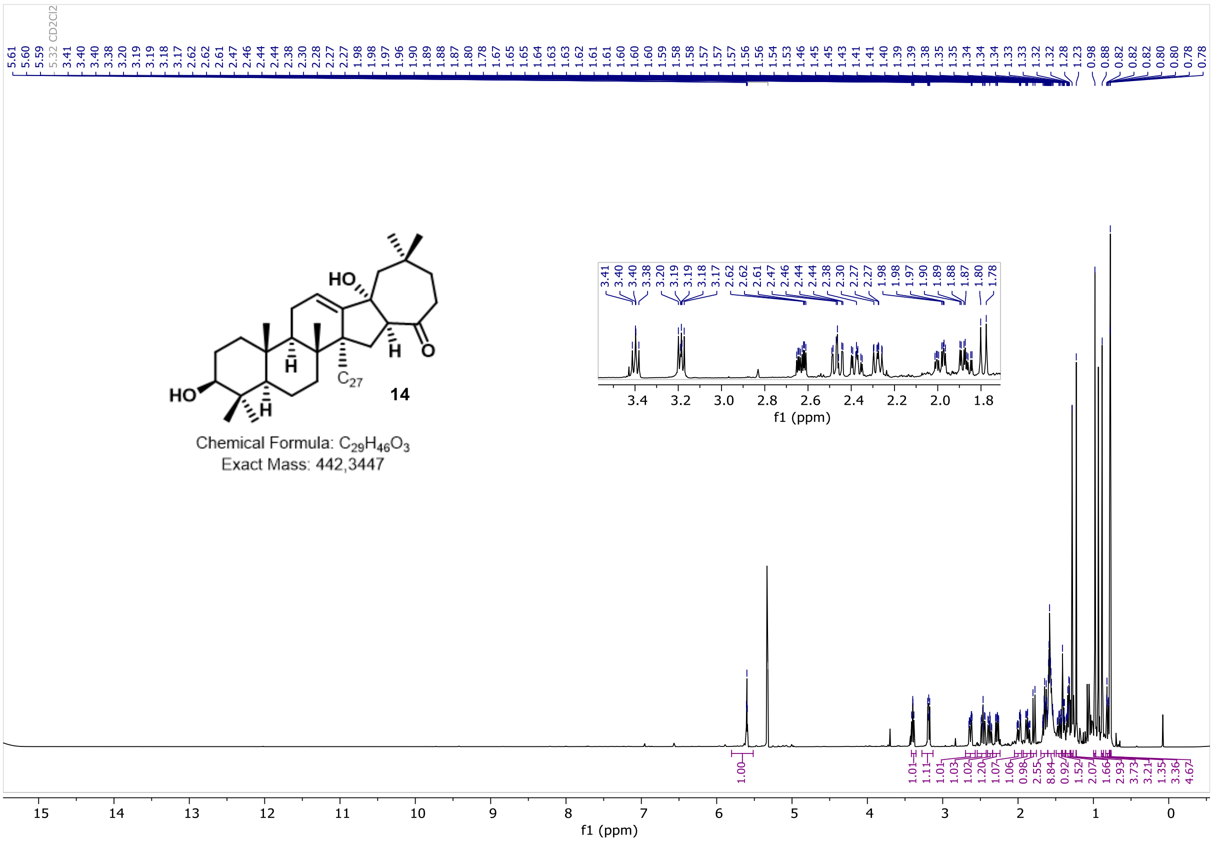
HO
HO
C27
14
Chemical Formula: $C_{29}H_{46}O_3$
Exact Mass: 442,3447
f1 (ppm)

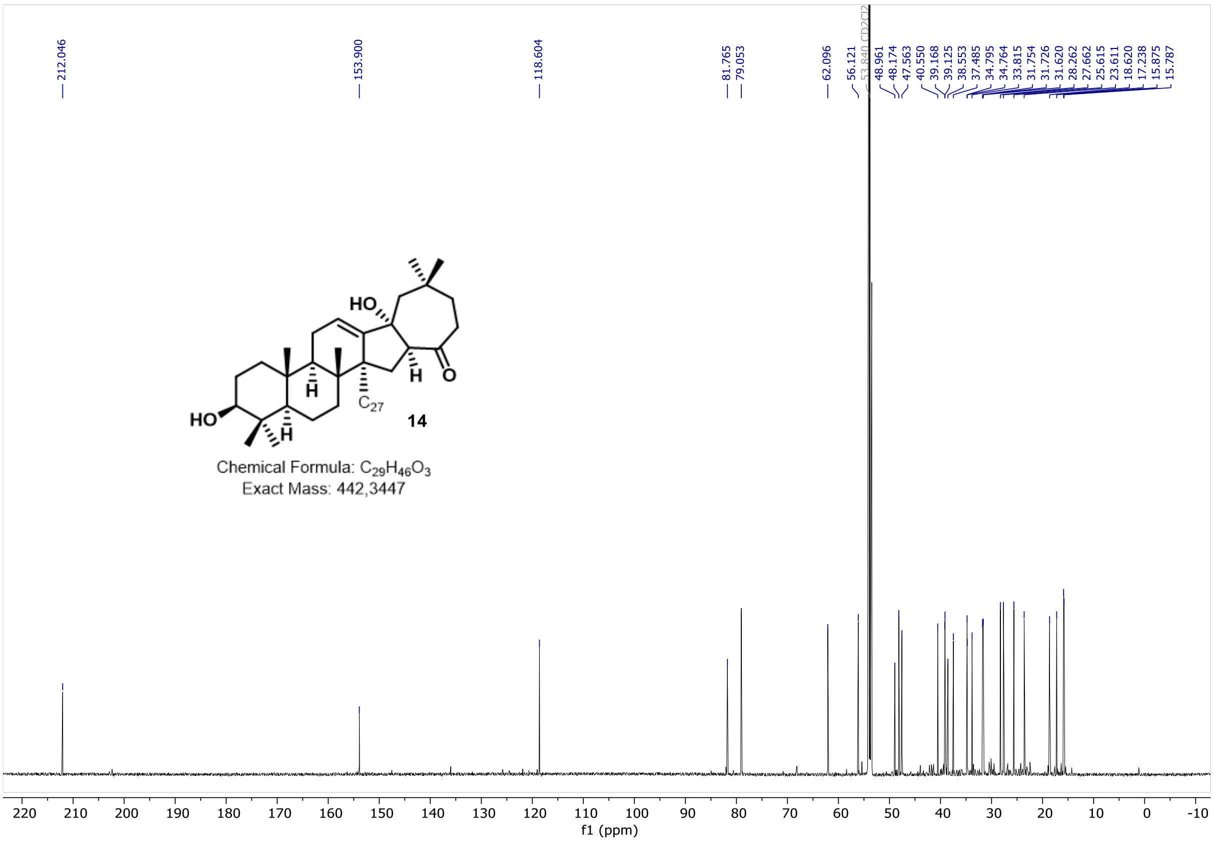
212.046
153.900
118.604
81.765
79.053
62.096
56.121
53.840 CD2Cl2
48.961
48.174
47.563
40.550
39.168
39.125
38.553
37.485
34.795
34.764
33.815
31.754
31.726
31.620
28.262
27.662
25.615
23.611
18.620
17.238
15.875
15.787
HO
HO
C27
14
Chemical Formula: $C_{29}H_{46}O_3$
Exact Mass: 442,3447
f1 (ppm)

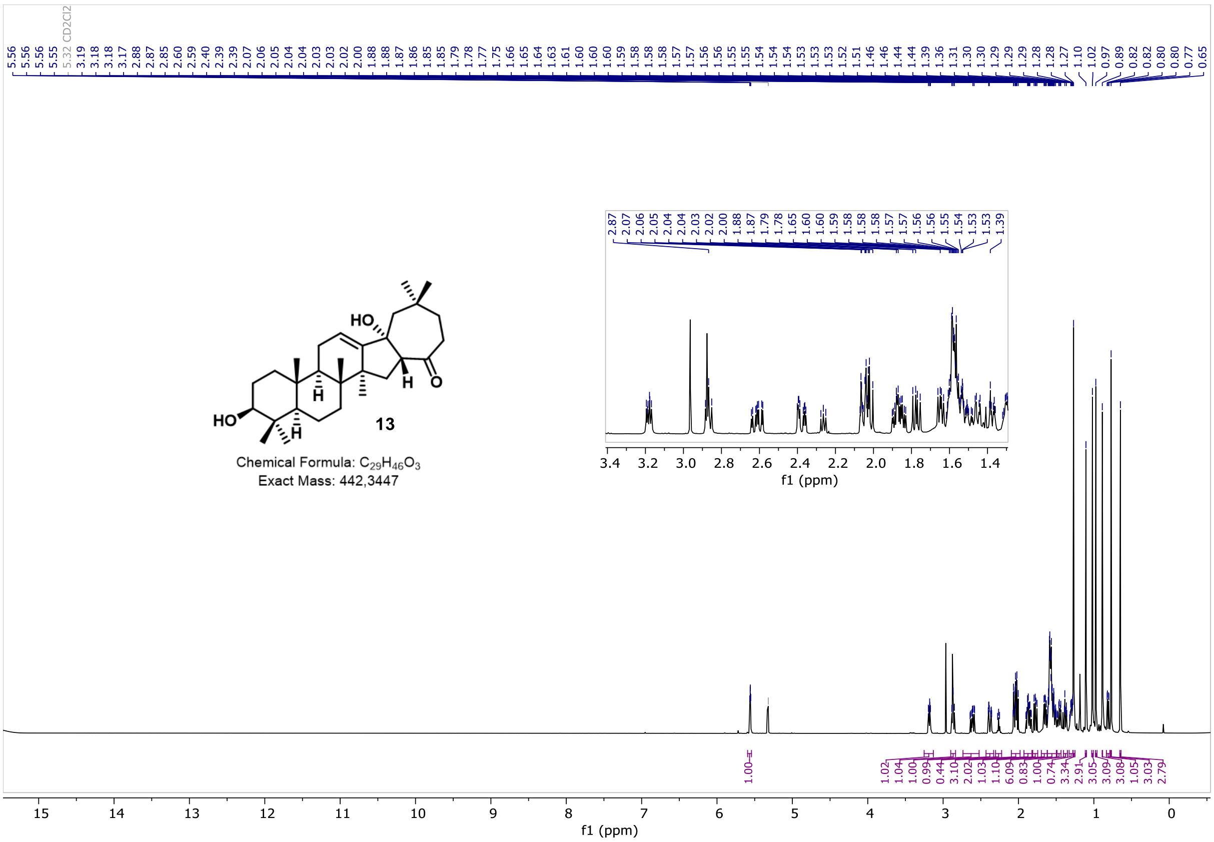
5.56 5.56 5.56 5.55 5.32 CD2Cl2 3.19 3.18 3.18 3.17 2.88 2.87 2.85 2.60 2.59 2.40 2.39 2.39 2.07 2.06 2.05 2.04 2.04 2.03 2.03 2.02 2.00 1.88 1.87 1.86 1.85 1.85 1.79 1.78 1.77 1.75 1.66 1.65 1.64 1.63 1.61 1.60 1.60 1.59 1.58 1.58 1.58 1.57 1.57 1.56 1.56 1.55 1.55 1.54 1.54 1.54 1.53 1.53 1.53 1.52 1.51 1.46 1.46 1.44 1.44 1.39 1.36 1.31 1.30 1.30 1.29 1.29 1.29 1.29 1.28 1.28 1.27 1.10 1.02 0.97 0.89 0.82 0.82 0.80 0.77 0.65
HO
HO
H
H
H
13
Chemical Formula: $C_{29}H_{46}O_3$
Exact Mass: 442,3447
f1 (ppm)

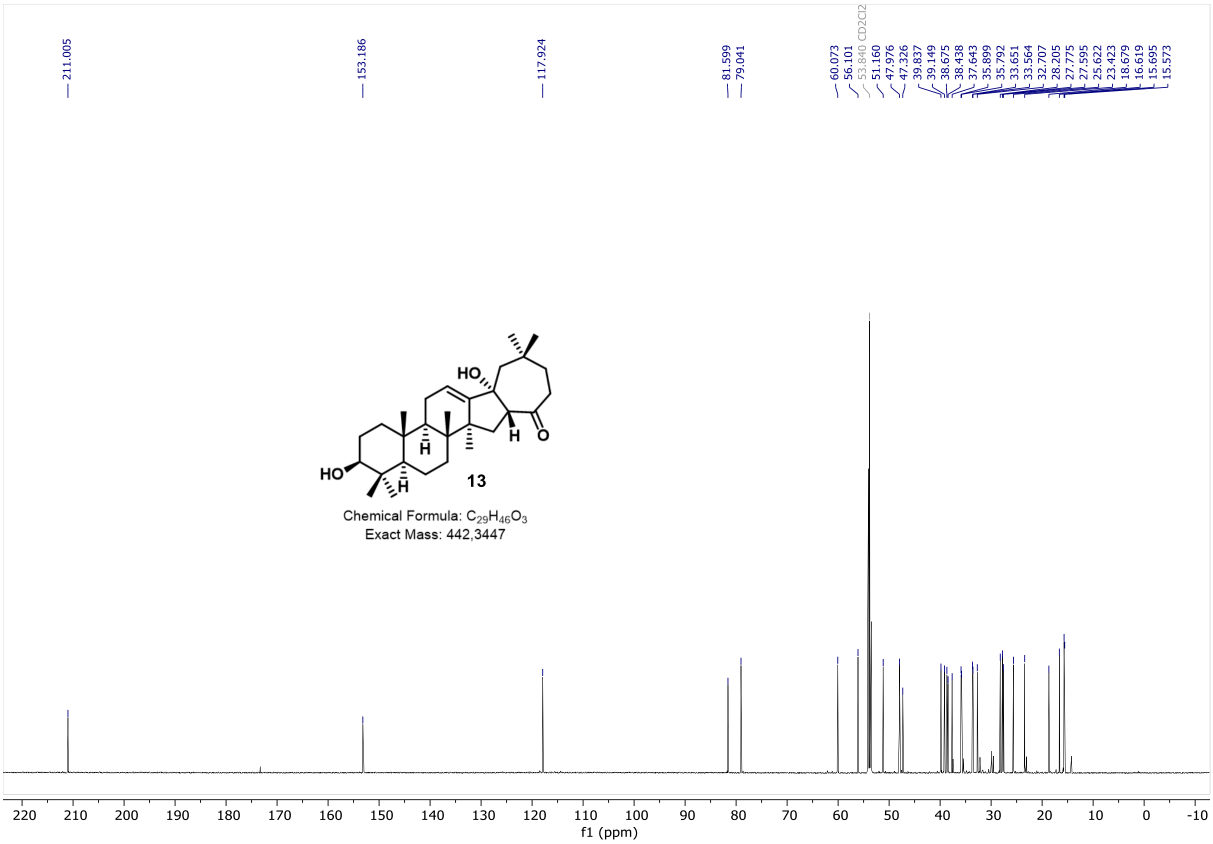
211.005 153.186 117.924 81.599 79.041 60.073 56.101 53.840 CD2Cl2 51.160 47.976 47.326 39.837 39.149 38.675 38.438 37.643 35.899 35.792 33.651 33.564 32.707 28.205 27.775 27.595 25.622 23.423 18.679 16.619 15.695 15.573
HO
HO
H
H
H
13
Chemical Formula: $C_{29}H_{46}O_3$
Exact Mass: 442,3447
f1 (ppm)

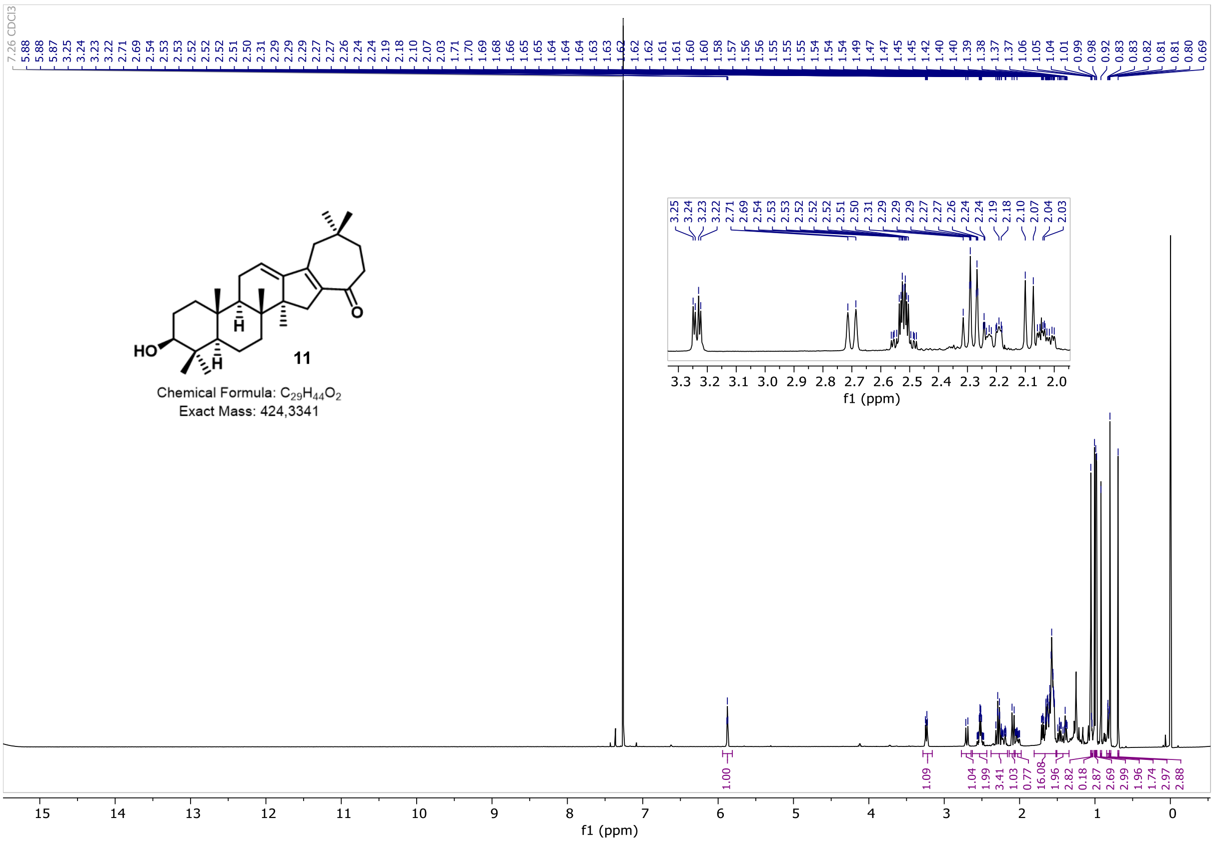
7.26 CDCl3
5.88 5.88 5.87 3.25 3.24 3.23 3.22 2.71 2.69 2.54 2.53 2.53 2.52 2.52 2.51 2.50 2.31 2.29 2.29 2.29 2.27 2.27 2.26 2.24 2.24 2.19 2.18 2.10 2.07 2.03 1.71 1.70 1.69 1.68 1.66 1.65 1.65 1.64 1.64 1.63 1.63 1.62 1.62 1.62 1.61 1.61 1.60 1.60 1.58 1.57 1.56 1.56 1.55 1.55 1.55 1.54 1.54 1.54 1.49 1.47 1.47 1.45 1.45 1.42 1.40 1.39 1.38 1.37 1.37 1.06 1.05 1.04 1.01 0.99 0.98 0.92 0.83 0.83 0.82 0.81 0.81 0.80 0.69
3.25 3.24 3.23 3.22 2.71 2.69 2.54 2.53 2.53 2.52 2.52 2.51 2.50 2.31 2.29 2.29 2.29 2.27 2.27 2.26 2.24 2.24 2.19 2.18 2.10 2.07 2.04 2.03
3.3 3.2 3.1 3.0 2.9 2.8 2.7 2.6 2.5 2.4 2.3 2.2 2.1 2.0
f1 (ppm)
HO
H
H
O
11
Chemical Formula: $C_{29}H_{44}O_2$
Exact Mass: 424,3341
1.00 1.09 1.04 1.99 3.41 1.03 0.77 16.08 1.96 2.82 0.18 2.87 2.69 2.99 1.96 1.74 2.97 2.88
15 14 13 12 11 10 9 8 7 6 5 4 3 2 1 0
f1 (ppm)

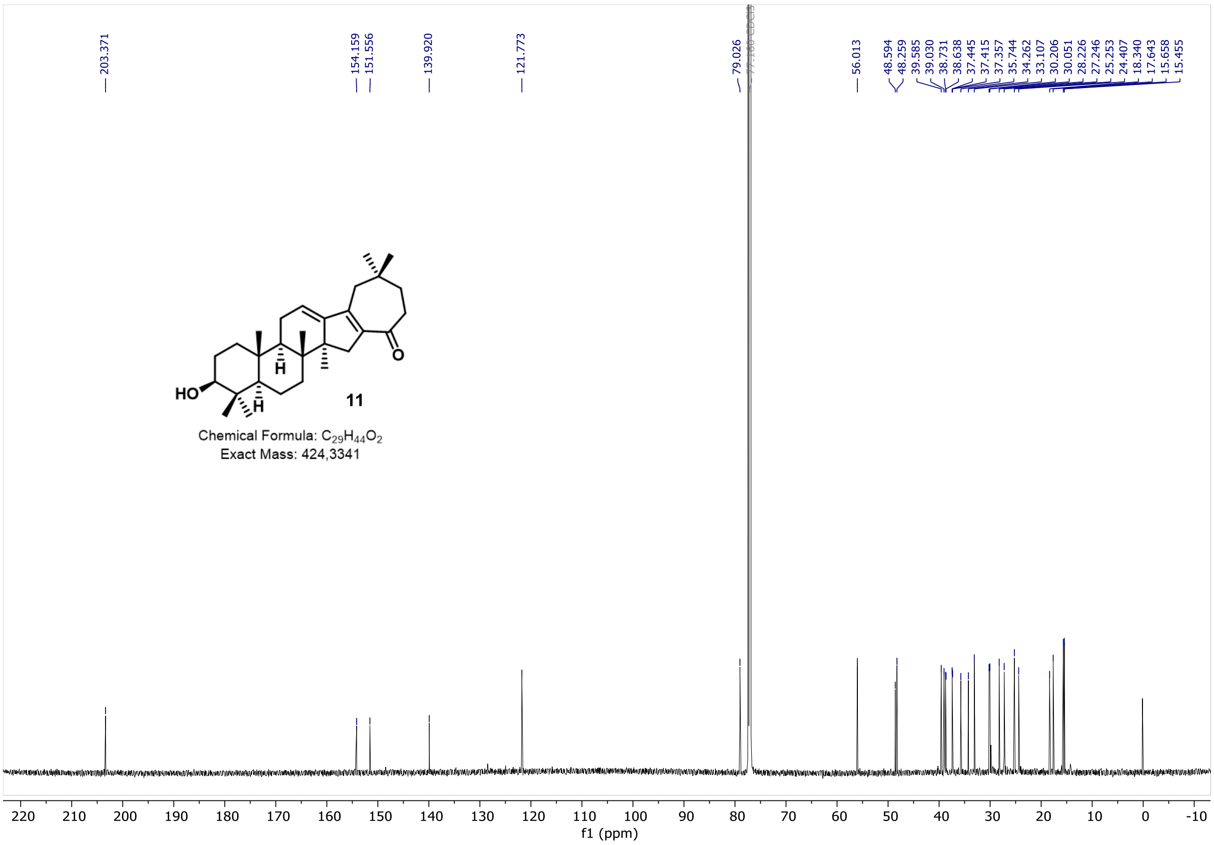
203.371
154.159 151.556
139.920
121.773
79.026
77.160 CDCl3
56.013
48.594 48.259 39.585 39.030 38.731 38.638 37.445 37.415 37.357 35.744 34.262 33.107 30.206 30.051 28.226 27.246 25.253 24.407 18.340 17.643 15.658 15.455
HO
H
H
O
11
Chemical Formula: $C_{29}H_{44}O_2$
Exact Mass: 424,3341
220 210 200 190 180 170 160 150 140 130 120 110 100 90 80 70 60 50 40 30 20 10 0 -10
f1 (ppm)

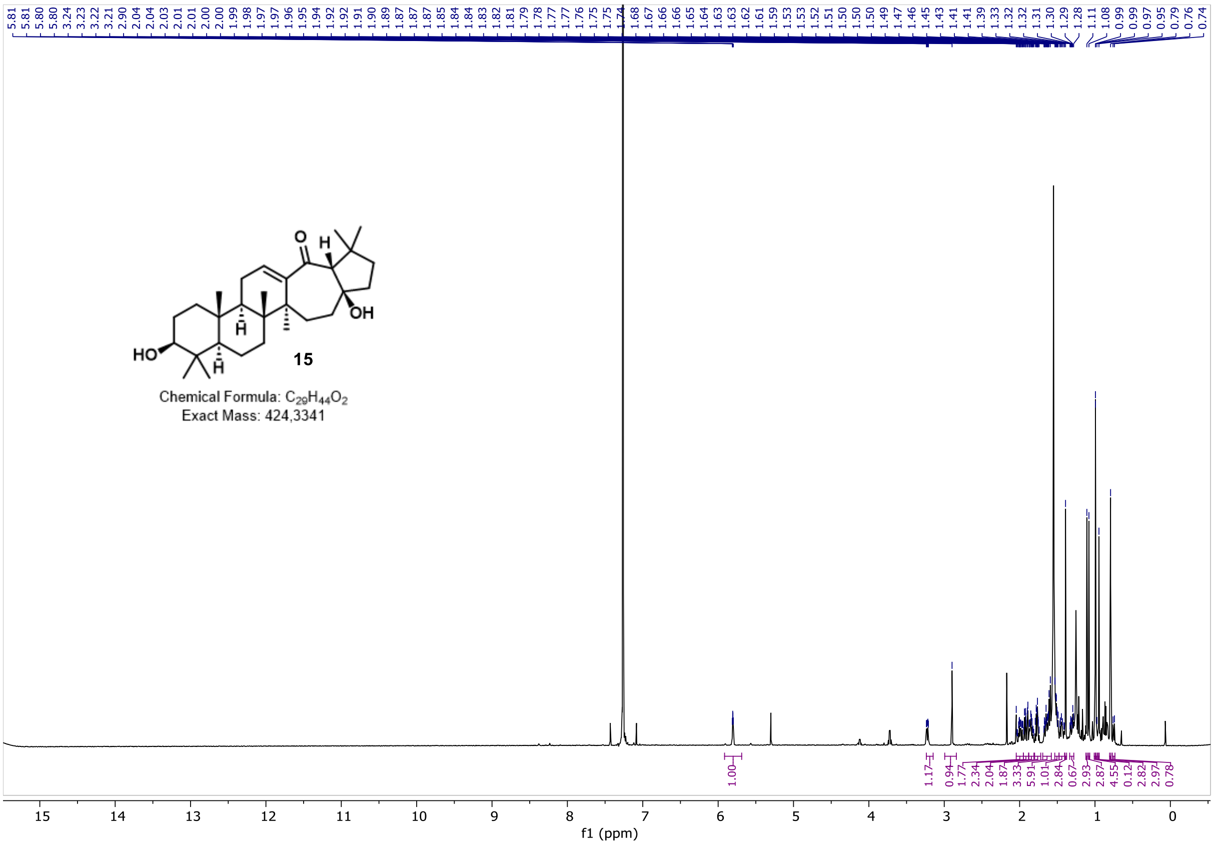
5.81 5.81 5.80 5.80 3.24 3.23 3.22 3.21 2.90 2.04 2.04 2.03 2.01 2.01 2.00 2.00 1.99 1.98 1.97 1.97 1.96 1.95 1.94 1.92 1.92 1.91 1.90 1.89 1.87 1.87 1.87 1.85 1.84 1.84 1.83 1.82 1.81 1.79 1.78 1.77 1.77 1.76 1.75 1.75 1.74 1.68 1.67 1.66 1.66 1.65 1.64 1.63 1.63 1.62 1.61 1.59 1.53 1.53 1.52 1.51 1.50 1.50 1.50 1.49 1.47 1.46 1.45 1.43 1.41 1.41 1.39 1.33 1.32 1.32 1.31 1.30 1.29 1.28 1.11 1.08 0.99 0.99 0.97 0.95 0.79 0.76 0.74
O
H
OH
HO
H
H
15
Chemical Formula: $C_{29}H_{44}O_2$
Exact Mass: 424,3341
1.00 1.17 0.94 1.77 2.34 2.04 1.87 3.33 5.91 1.01 2.84 0.67 2.93 2.87 4.55 0.12 2.82 2.97 0.78
15 14 13 12 11 10 9 8 7 6 5 4 3 2 1 0
f1 (ppm)

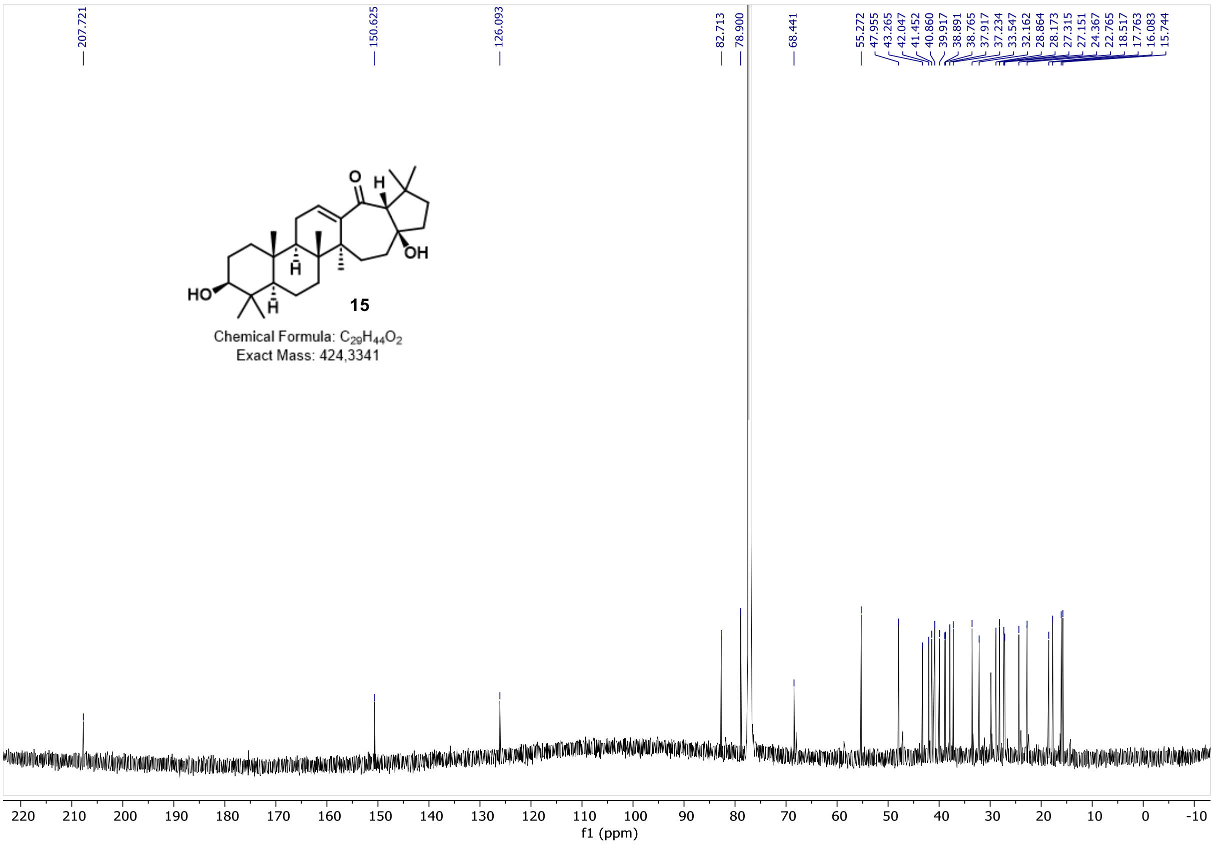
207.721
150.625
126.093
82.713
78.900
68.441
55.272 47.955 43.265 42.047 41.452 40.860 39.917 38.891 38.765 37.917 37.234 33.547 32.162 28.864 28.173 27.315 27.151 24.367 22.765 18.517 17.763 16.083 15.744
O
H
OH
HO
H
H
15
Chemical Formula: $C_{29}H_{44}O_2$
Exact Mass: 424,3341
220 210 200 190 180 170 160 150 140 130 120 110 100 90 80 70 60 50 40 30 20 10 0 -10
f1 (ppm)

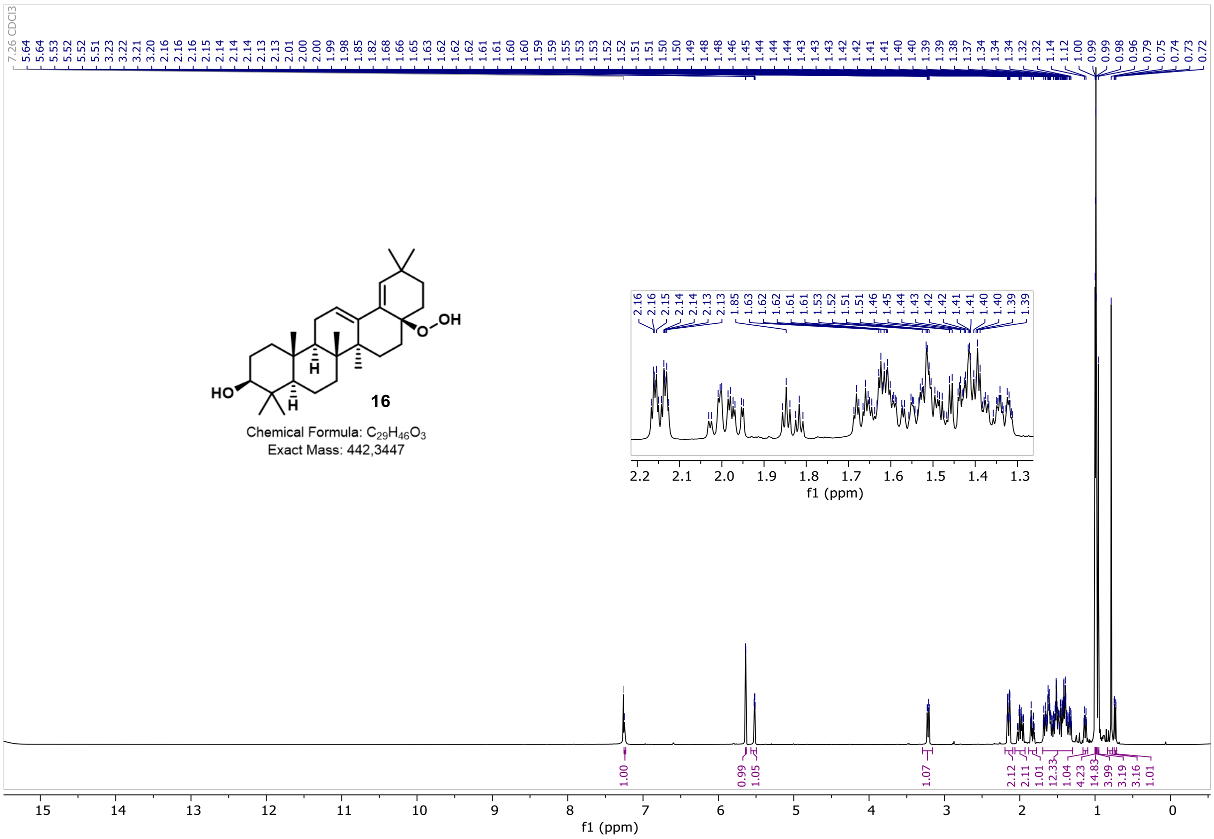
7.26 CDCl3
5.64 5.64 5.53 5.52 5.52 5.51 3.23 3.22 3.21 3.20 2.16 2.16 2.16 2.15 2.14 2.14 2.14 2.13 2.13 2.01 2.00 2.00 1.99 1.98 1.85 1.82 1.68 1.66 1.65 1.63 1.62 1.62 1.61 1.61 1.60 1.59 1.59 1.55 1.53 1.53 1.52 1.52 1.51 1.51 1.51 1.50 1.50 1.49 1.48 1.48 1.46 1.45 1.44 1.44 1.44 1.44 1.43 1.43 1.43 1.42 1.42 1.41 1.41 1.41 1.40 1.40 1.39 1.39 1.38 1.37 1.34 1.34 1.34 1.32 1.32 1.14 1.12 1.00 0.99 0.99 0.98 0.96 0.79 0.75 0.74 0.73 0.72
HO
H
H
O
OH
16
Chemical Formula: $C_{29}H_{46}O_3$
Exact Mass: 442,3447
2.16 2.16 2.15 2.14 2.14 2.14 2.13 2.13 1.85 1.63 1.62 1.62 1.61 1.61 1.53 1.52 1.51 1.51 1.46 1.45 1.44 1.43 1.42 1.42 1.41 1.41 1.40 1.40 1.39 1.39
2.2 2.1 2.0 1.9 1.8 1.7 1.6 1.5 1.4 1.3
f1 (ppm)
1.00 0.99 1.05 1.07 2.12 2.11 1.01 12.33 1.04 4.23 14.83 3.99 3.19 3.16 1.01
15 14 13 12 11 10 9 8 7 6 5 4 3 2 1 0
f1 (ppm)

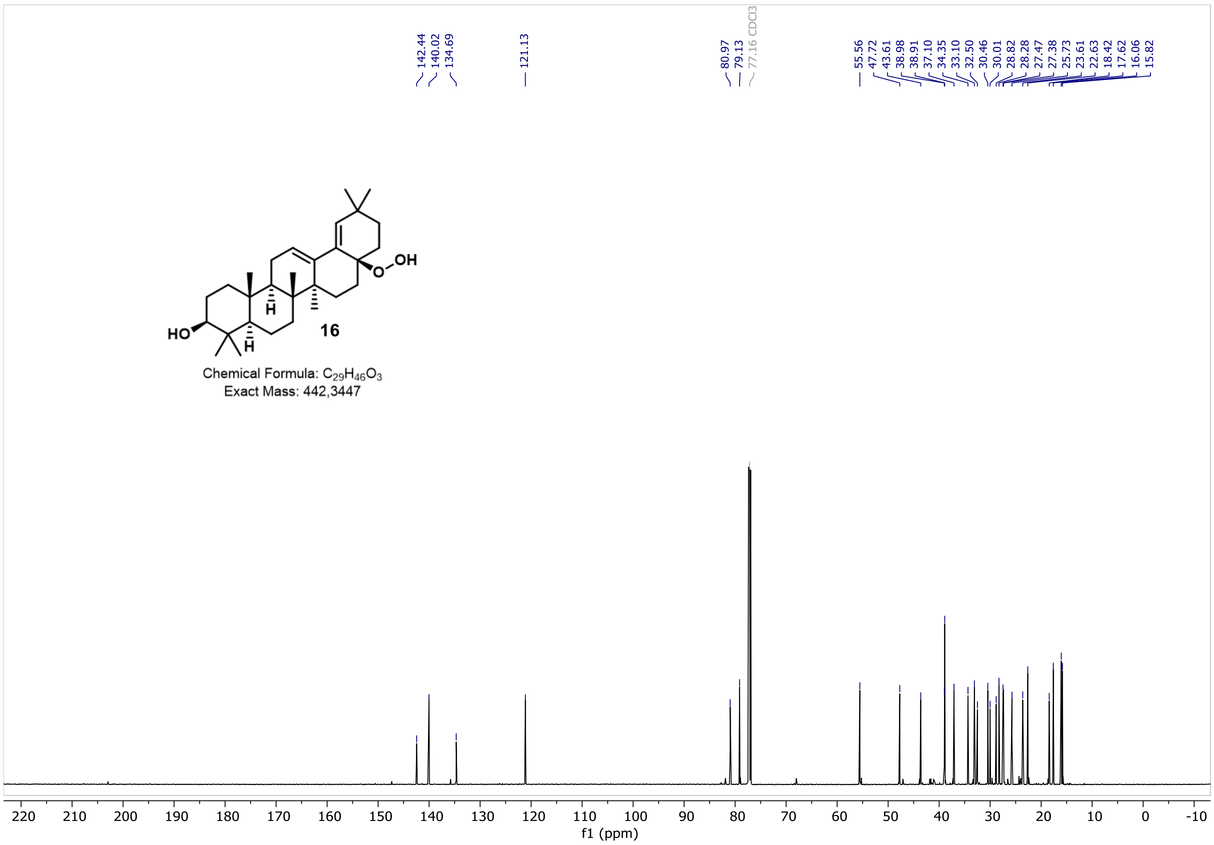
142.44 140.02 134.69 121.13 80.97 79.13 77.16 CDCl3 55.56 47.72 43.61 38.98 38.91 37.10 34.35 33.10 32.50 30.46 30.01 28.82 28.28 27.47 27.38 25.73 23.61 22.63 18.42 17.62 16.06 15.82
HO
H
H
O
OH
16
Chemical Formula: $C_{29}H_{46}O_3$
Exact Mass: 442,3447
220 210 200 190 180 170 160 150 140 130 120 110 100 90 80 70 60 50 40 30 20 10 0 -10
f1 (ppm)

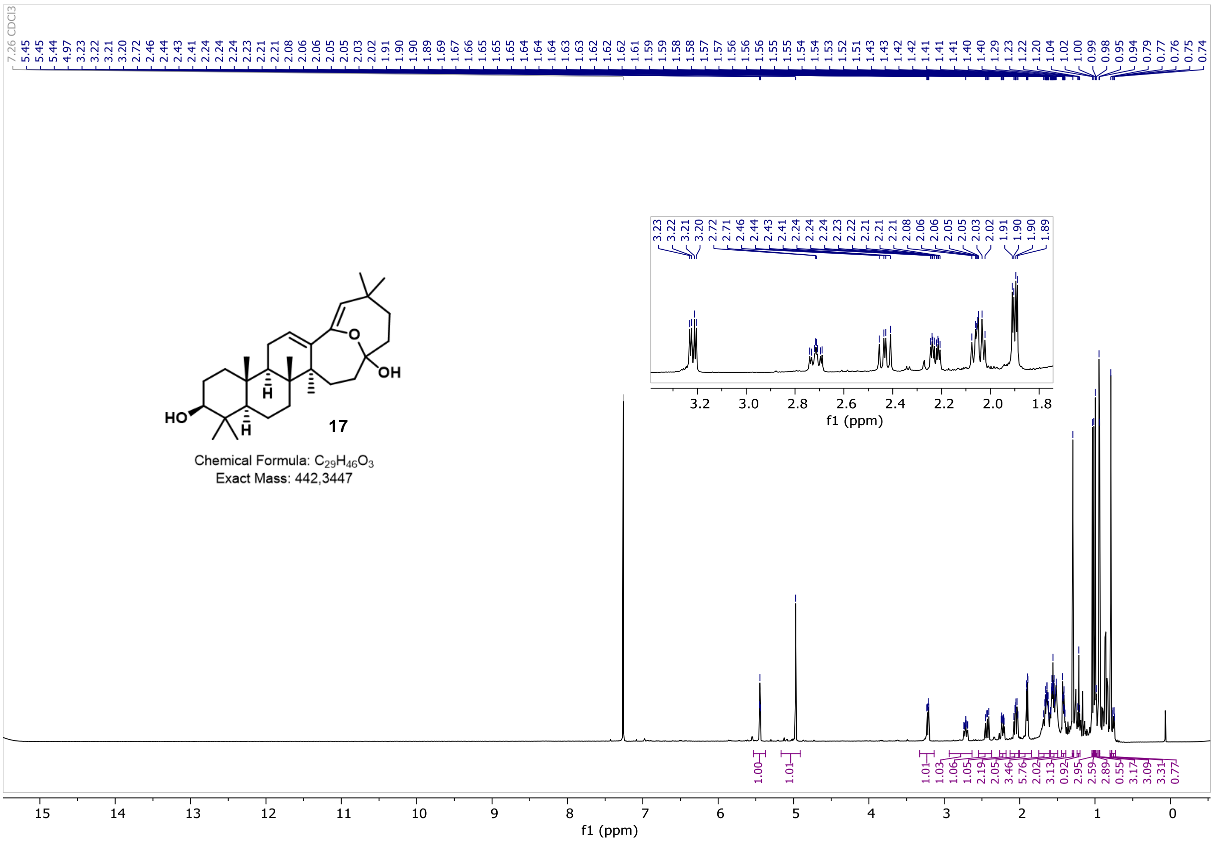
HO
H
H
OH
O
17
Chemical Formula: $C_{29}H_{46}O_3$
Exact Mass: 442,3447
f1 (ppm)

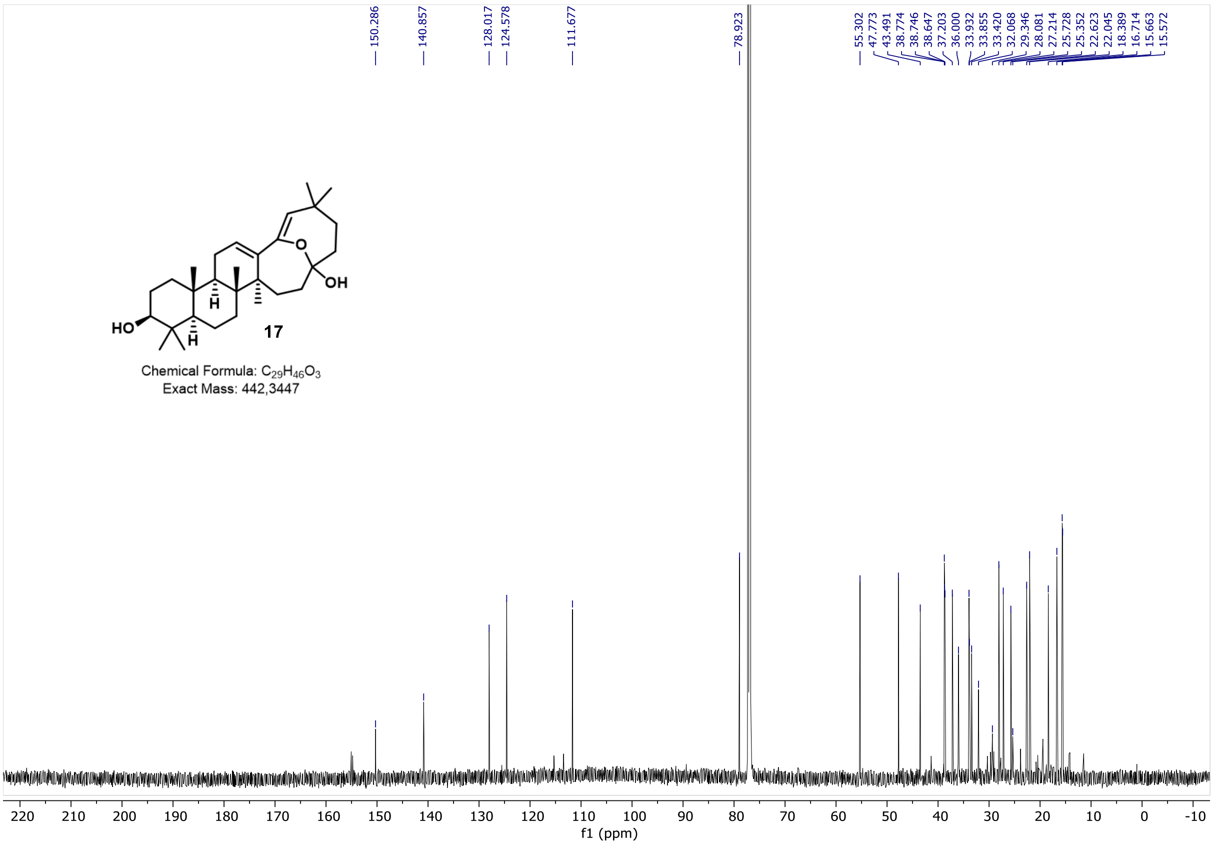
HO
H
H
OH
O
17
Chemical Formula: $C_{29}H_{46}O_3$
Exact Mass: 442,3447
150.286
140.857
128.017
124.578
111.677
78.923
55.302
47.773
43.491
38.774
38.746
38.647
37.203
36.000
33.932
33.855
33.420
32.068
29.346
28.081
27.214
25.728
25.352
22.623
22.045
18.389
16.714
15.663
15.572
f1 (ppm)

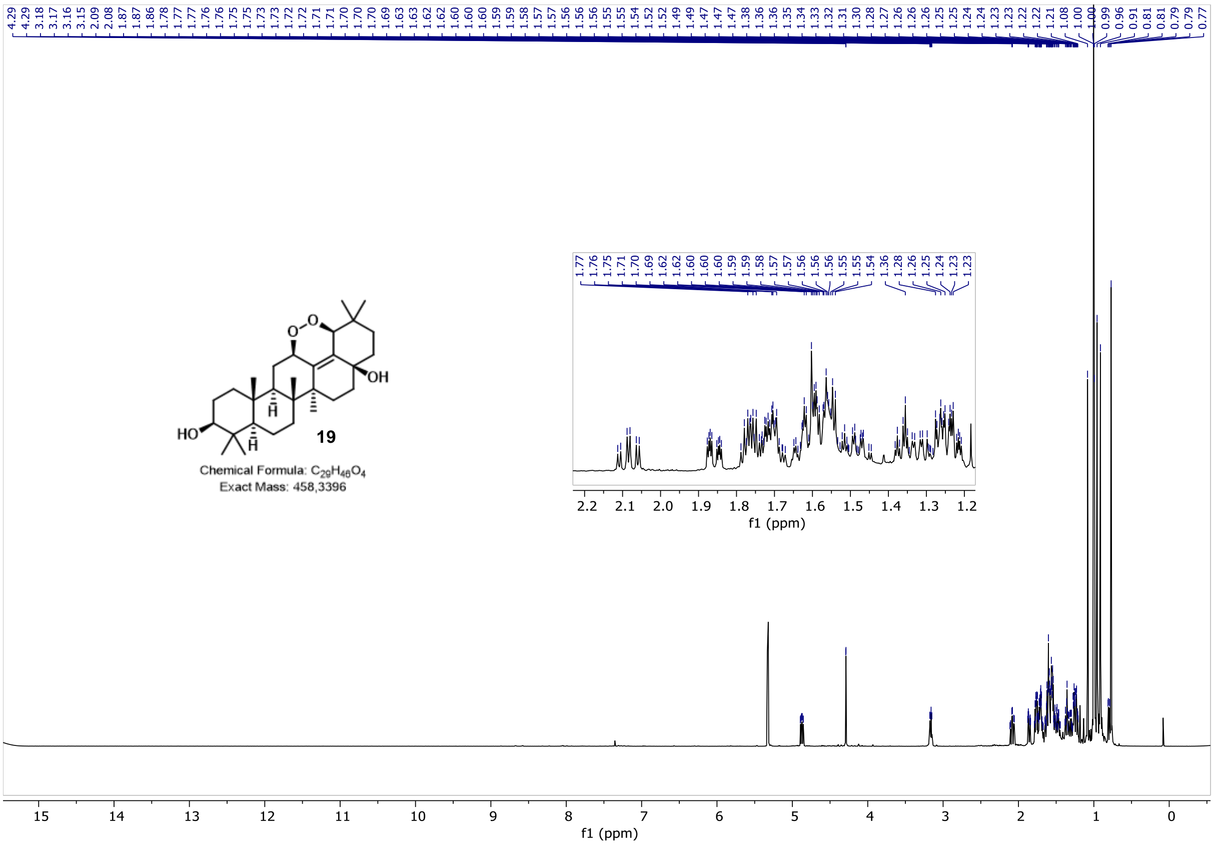
4.29 4.29 3.18 3.17 3.16 3.15 2.09 2.08 1.87 1.87 1.86 1.78 1.77 1.77 1.76 1.76 1.75 1.75 1.73 1.73 1.72 1.72 1.71 1.71 1.70 1.70 1.70 1.69 1.63 1.62 1.62 1.60 1.60 1.60 1.59 1.59 1.58 1.57 1.57 1.56 1.56 1.56 1.55 1.55 1.54 1.52 1.52 1.49 1.47 1.47 1.47 1.38 1.36 1.36 1.35 1.34 1.33 1.32 1.31 1.30 1.28 1.27 1.26 1.26 1.26 1.25 1.25 1.24 1.24 1.23 1.23 1.22 1.22 1.21 1.08 1.00 1.00 0.99 0.96 0.91 0.81 0.81 0.79 0.77
HO
OH
19
Chemical Formula: $C_{29}H_{46}O_4$
Exact Mass: 458,3396
1.77 1.76 1.75 1.71 1.70 1.69 1.62 1.62 1.60 1.60 1.60 1.59 1.59 1.58 1.57 1.57 1.56 1.56 1.56 1.55 1.55 1.54 1.36 1.28 1.26 1.25 1.25 1.24 1.23 1.23
2.2 2.1 2.0 1.9 1.8 1.7 1.6 1.5 1.4 1.3 1.2
f1 (ppm)
15 14 13 12 11 10 9 8 7 6 5 4 3 2 1 0
f1 (ppm)

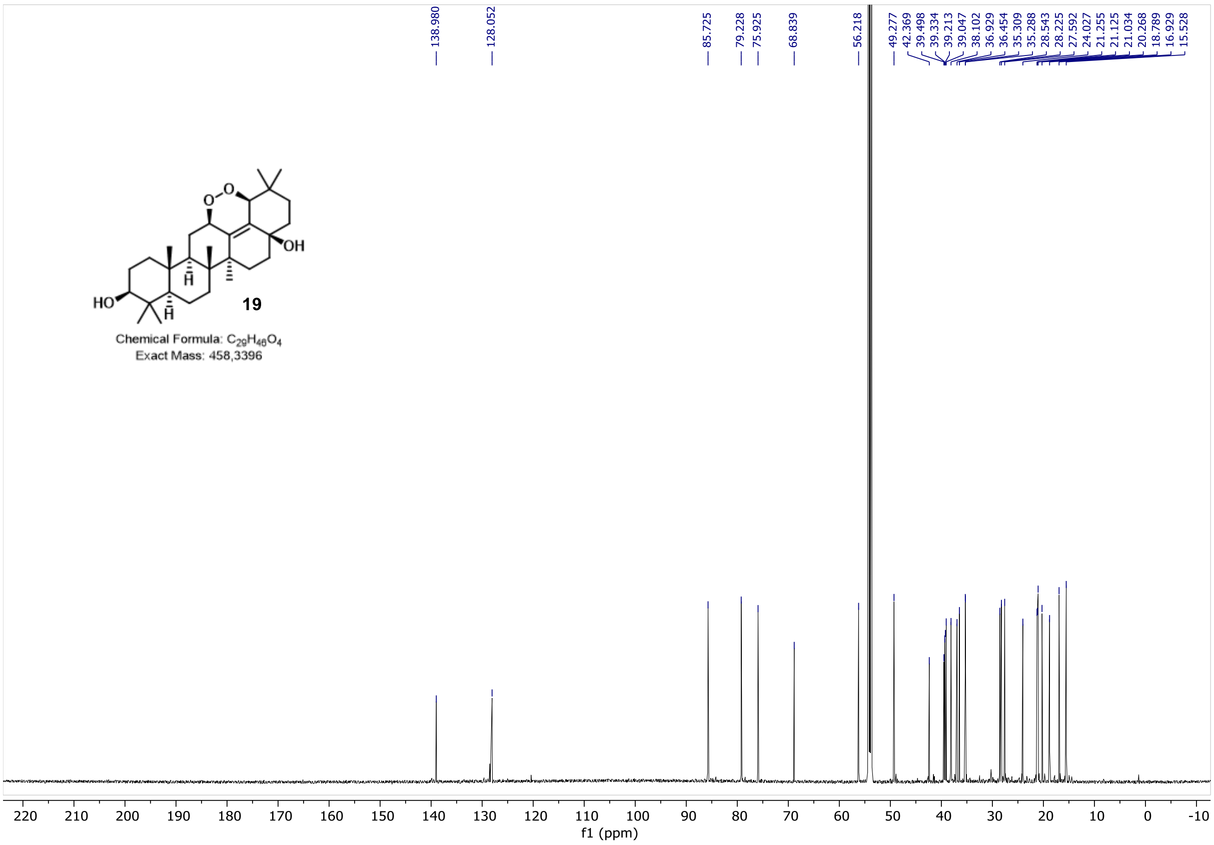
138.980
128.052
85.725
79.228
75.925
68.839
56.218
49.277 42.369 39.498 39.334 39.213 39.047 38.102 36.929 36.454 35.309 35.288 28.543 28.225 27.592 24.027 21.255 21.125 21.034 20.268 18.789 16.929 15.528
HO
OH
19
Chemical Formula: $C_{29}H_{46}O_4$
Exact Mass: 458,3396
220 210 200 190 180 170 160 150 140 130 120 110 100 90 80 70 60 50 40 30 20 10 0 -10
f1 (ppm)

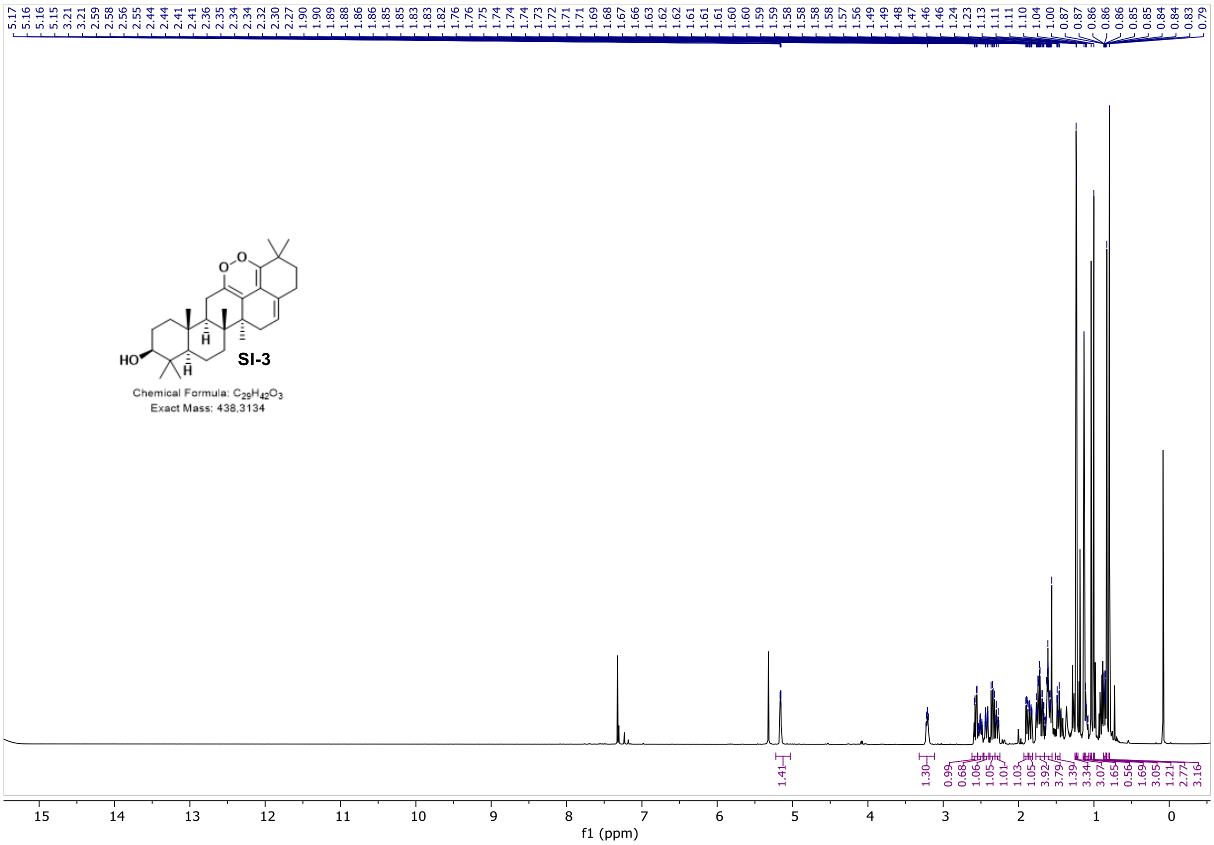
SI-3
Chemical Formula: $C_{29}H_{42}O_3$
Exact Mass: 438.3134
f1 (ppm)

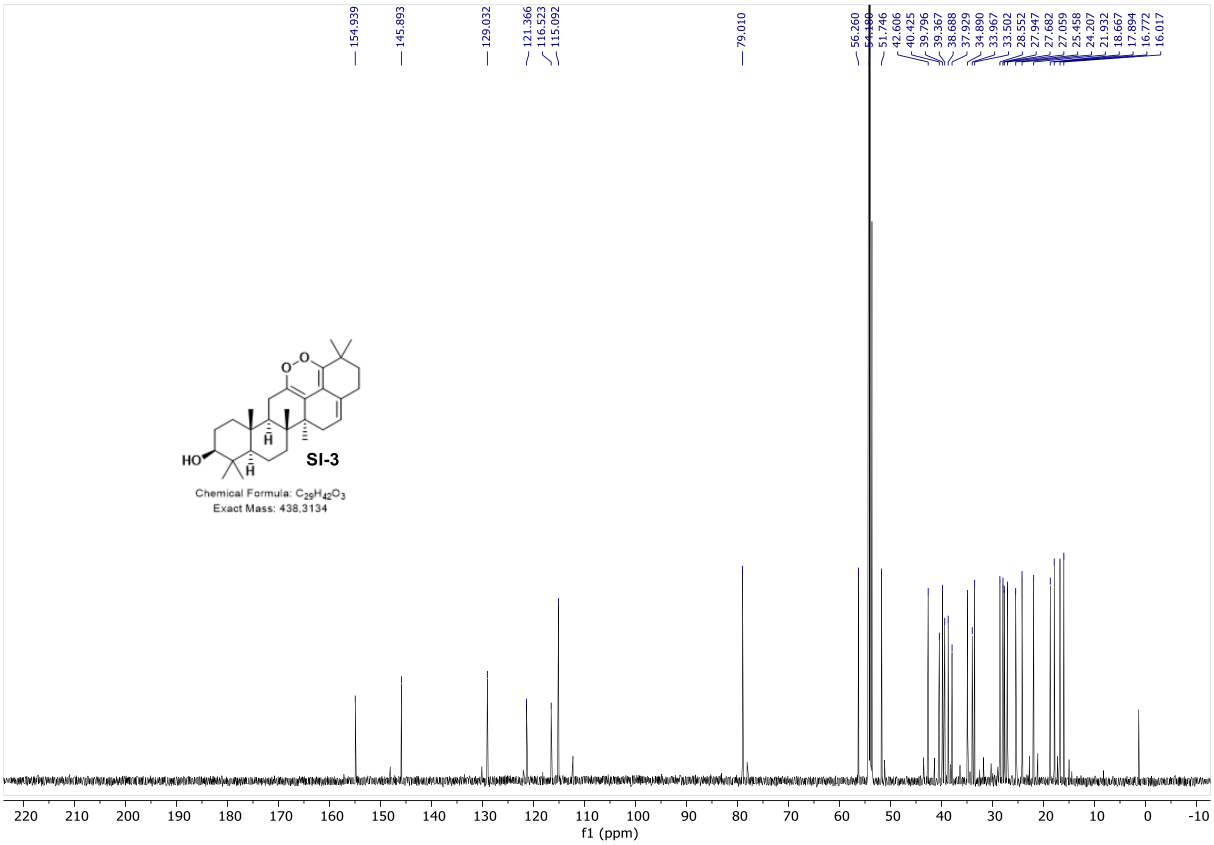
154.939
145.893
129.032
121.366
116.523
115.092
79.010
56.260
51.746
42.606
40.425
39.796
39.367
38.688
37.929
34.890
33.967
33.502
28.552
27.947
27.682
27.059
25.458
24.207
21.932
18.667
17.894
16.772
16.017
SI-3
Chemical Formula: $C_{29}H_{42}O_3$
Exact Mass: 438.3134
f1 (ppm)

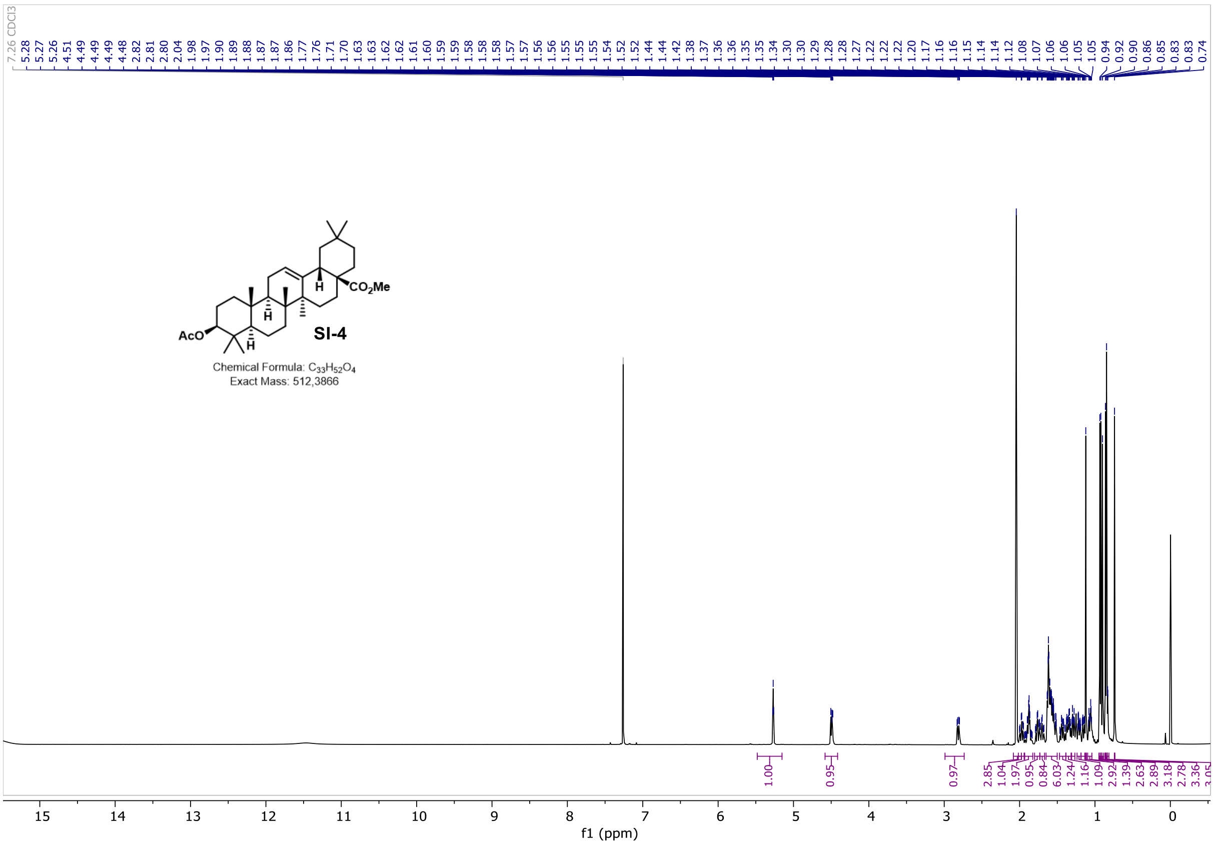

7.26 CDCl3
5.28
5.27
5.26
4.51
4.49
4.49
4.48
2.82
2.81
2.80
2.04
1.98
1.97
1.90
1.89
1.88
1.87
1.87
1.86
1.77
1.76
1.71
1.70
1.63
1.63
1.62
1.61
1.60
1.59
1.59
1.58
1.58
1.58
1.57
1.57
1.56
1.56
1.55
1.55
1.55
1.54
1.52
1.52
1.44
1.44
1.42
1.38
1.37
1.36
1.36
1.35
1.35
1.34
1.30
1.30
1.29
1.28
1.28
1.27
1.22
1.22
1.22
1.20
1.17
1.16
1.15
1.14
1.14
1.12
1.08
1.07
1.06
1.06
1.05
1.05
0.94
0.92
0.90
0.86
0.85
0.83
0.83
0.74
AcO
H
H
H
CO_2Me
SI-4
Chemical Formula: $C_{33}H_{52}O_4$
Exact Mass: 512,3866
1.00
0.95
0.97
f1 (ppm)

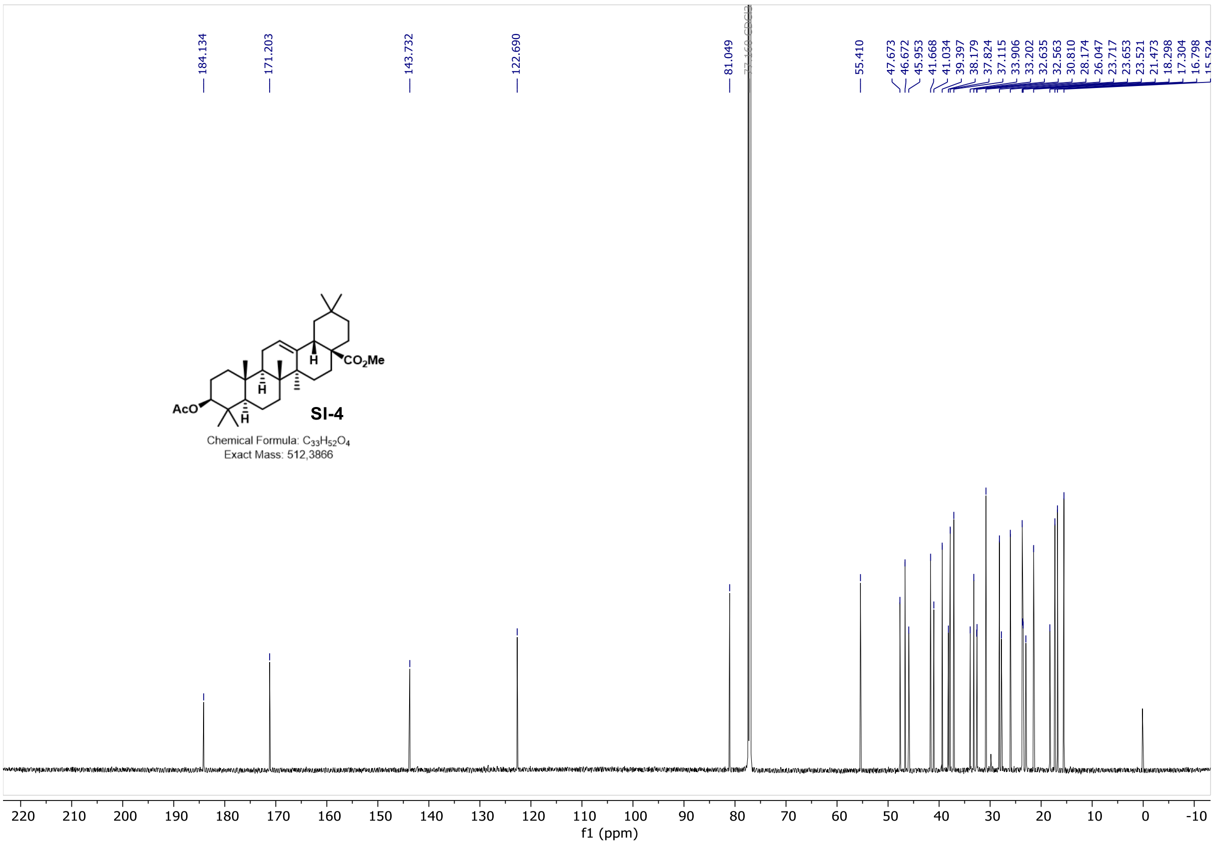

184.134
171.203
143.732
122.690
81.049
55.410
47.673
46.672
45.953
41.668
41.034
39.397
38.179
37.824
37.115
33.906
33.202
32.635
32.563
30.810
28.174
26.047
23.717
23.653
23.521
21.473
18.298
17.304
16.798
AcO
H
H
H
CO_2Me
SI-4
Chemical Formula: $C_{33}H_{52}O_4$
Exact Mass: 512,3866
f1 (ppm)

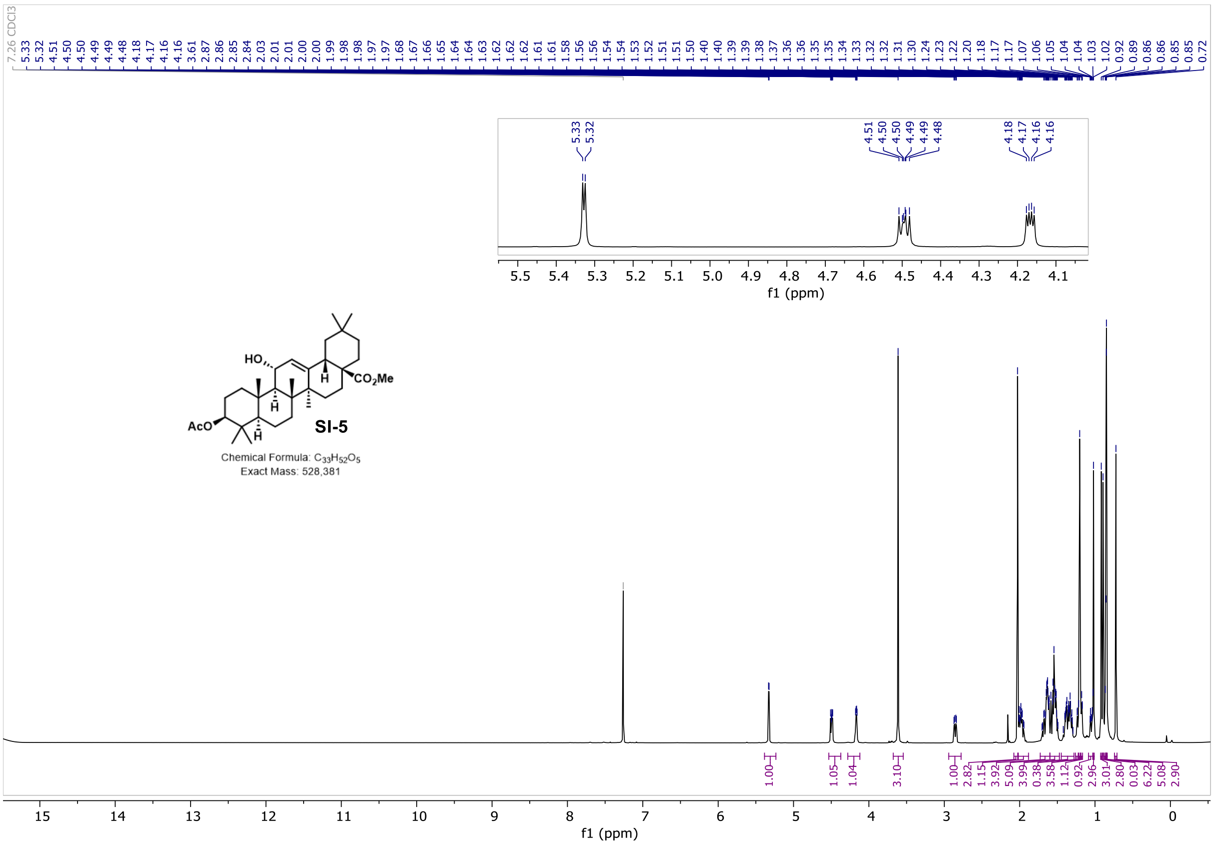
7.26 CDCl3
5.33
5.32
4.51
4.50
4.50
4.49
4.49
4.48
4.18
4.17
4.16
4.16
3.61
2.87
2.86
2.85
2.84
2.03
2.01
2.01
2.00
2.00
1.99
1.98
1.98
1.97
1.97
1.68
1.67
1.66
1.65
1.64
1.64
1.63
1.62
1.62
1.62
1.61
1.61
1.58
1.56
1.56
1.54
1.54
1.53
1.52
1.51
1.51
1.50
1.40
1.40
1.39
1.39
1.38
1.37
1.36
1.36
1.35
1.35
1.34
1.33
1.32
1.32
1.31
1.30
1.24
1.23
1.22
1.20
1.18
1.17
1.17
1.07
1.06
1.05
1.04
1.04
1.03
1.02
0.92
0.89
0.86
0.86
0.85
0.85
0.72
HO
H
H
H
CO2Me
AcO
SI-5
Chemical Formula: C33H52O5
Exact Mass: 528,381
1.00
1.05
1.04
3.10
1.00
2.82
1.15
3.92
5.09
3.99
0.38
3.58
1.12
0.92
2.96
3.01
2.80
0.03
6.22
5.08
2.90
f1 (ppm)

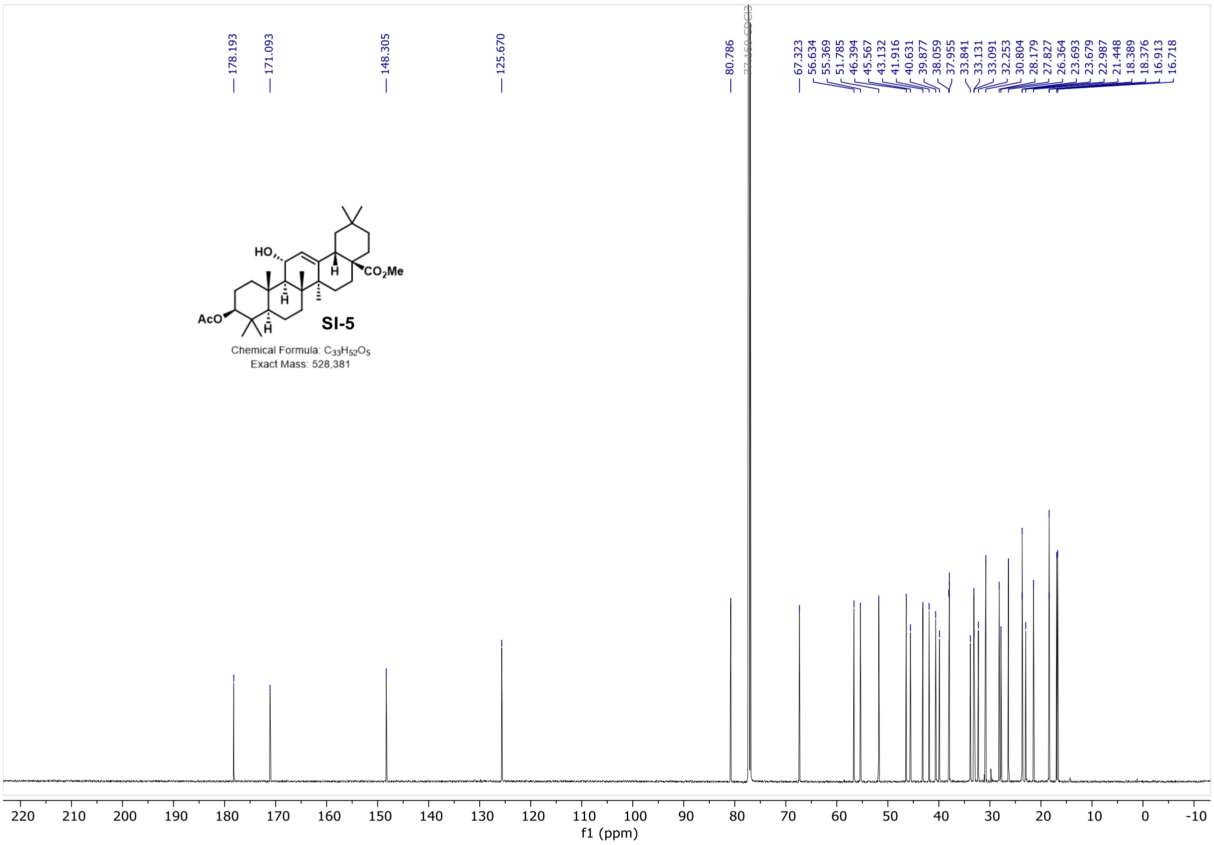
178.193
171.093
148.305
125.670
80.786
67.323
56.634
55.369
51.785
46.394
45.567
43.132
41.916
40.631
39.877
38.059
37.955
33.841
33.131
33.091
32.253
30.804
28.179
27.827
26.364
23.693
23.679
22.987
21.448
18.389
18.376
16.913
16.718
HO
H
H
H
CO2Me
AcO
SI-5
Chemical Formula: C33H52O5
Exact Mass: 528,381
f1 (ppm)

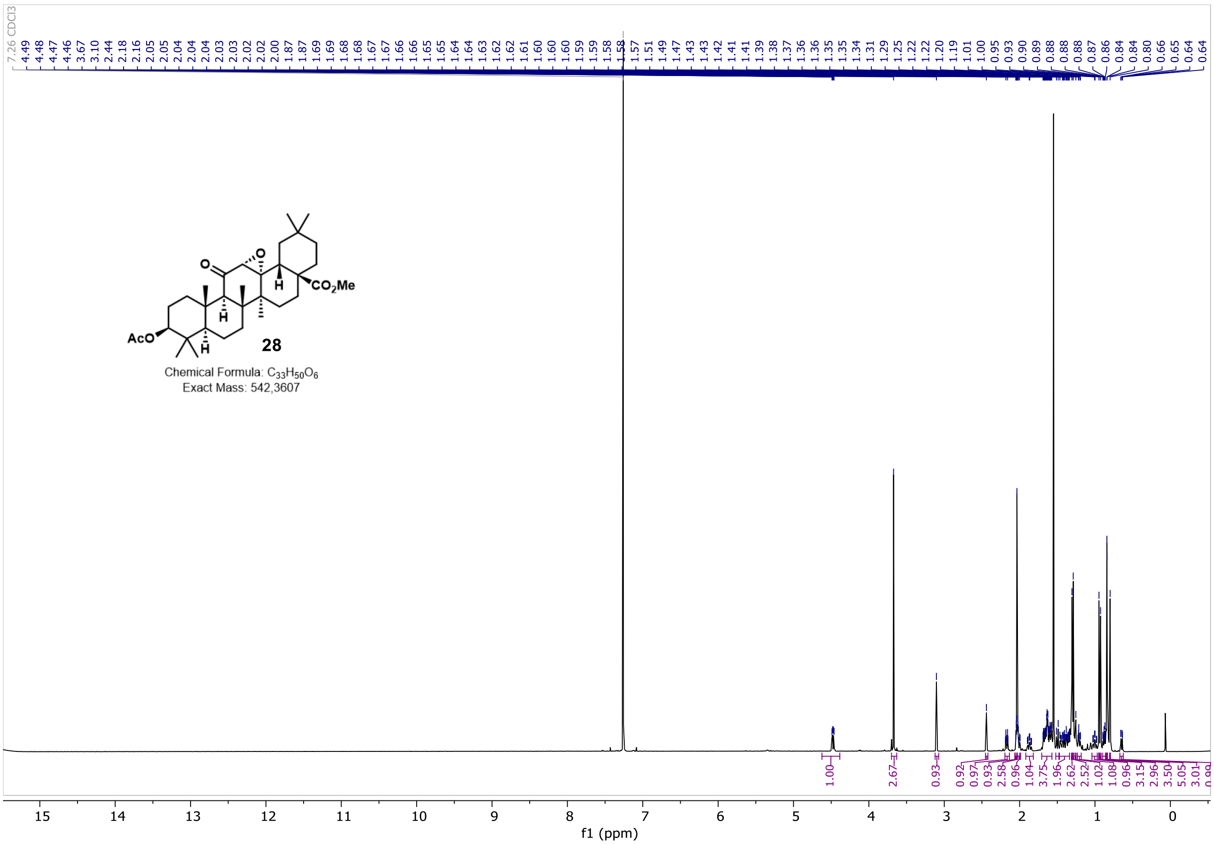
7.26 CDCl3
4.49 4.48 4.47 4.46 3.67 3.10 2.44 2.18 2.16 2.05 2.04 2.04 2.04 2.03 2.03 2.02 2.02 2.00 1.87 1.87 1.69 1.69 1.68 1.68 1.67 1.67 1.66 1.66 1.65 1.65 1.64 1.64 1.63 1.62 1.62 1.61 1.60 1.60 1.60 1.59 1.59 1.58 1.58 1.57 1.51 1.49 1.47 1.43 1.43 1.42 1.41 1.41 1.39 1.38 1.37 1.36 1.36 1.35 1.35 1.34 1.31 1.29 1.25 1.22 1.22 1.20 1.19 1.01 1.00 0.95 0.93 0.90 0.89 0.88 0.88 0.88 0.87 0.86 0.86 0.84 0.84 0.80 0.66 0.65 0.64 0.64
O
H
CO2Me
H
AcO
H
28
Chemical Formula: C33H50O6
Exact Mass: 542,3607
15 14 13 12 11 10 9 8 7 6 5 4 3 2 1 0
f1 (ppm)

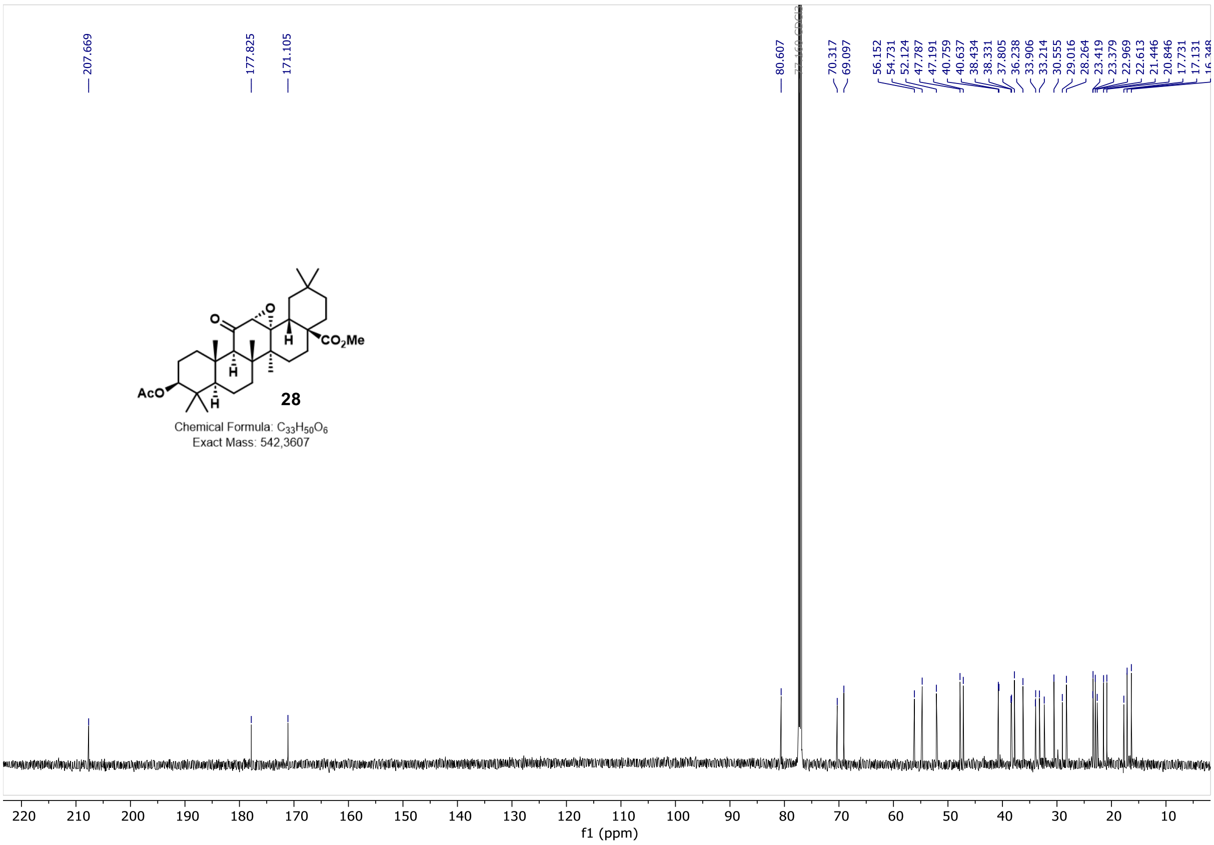
207.669
177.825
171.105
80.607
70.317 69.097
56.152 54.731 52.124 47.787 47.191 40.759 40.637 38.434 38.331 37.805 36.238 33.906 33.214 30.555 29.016 28.264 23.419 23.379 22.969 22.613 21.446 20.846 17.731 17.131
O
H
CO2Me
H
AcO
H
28
Chemical Formula: C33H50O6
Exact Mass: 542,3607
220 210 200 190 180 170 160 150 140 130 120 110 100 90 80 70 60 50 40 30 20 10
f1 (ppm)

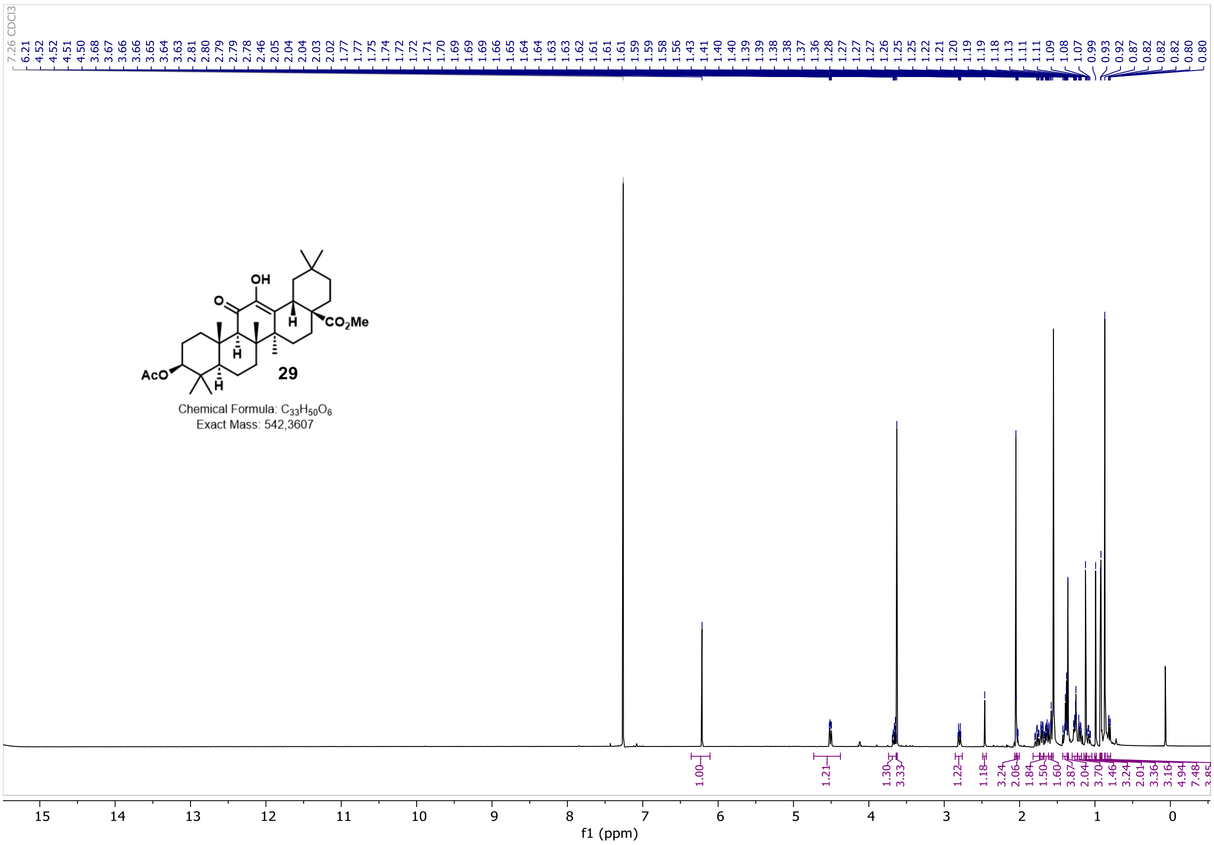
29
Chemical Formula: $C_{33}H_{50}O_6$
Exact Mass: 542,3607
f1 (ppm)

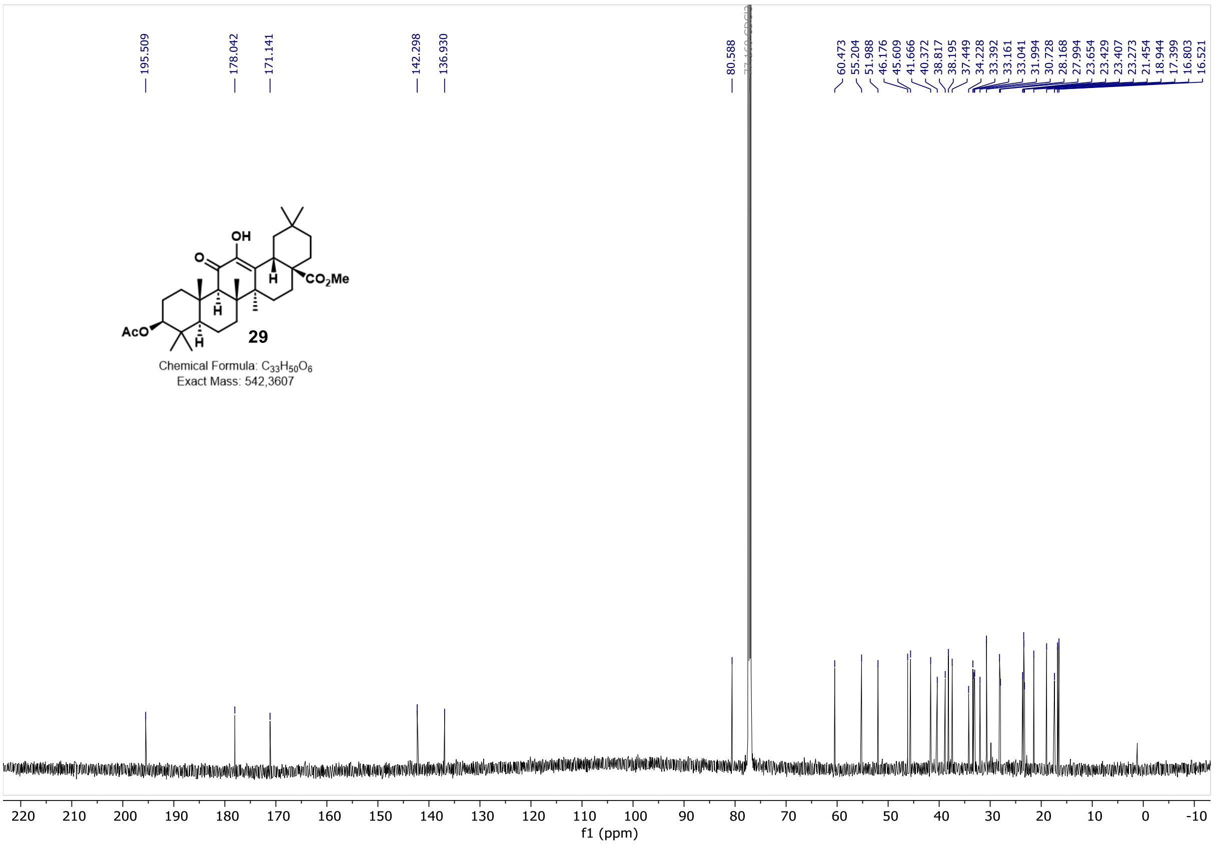
29
Chemical Formula: $C_{33}H_{50}O_6$
Exact Mass: 542,3607
f1 (ppm)

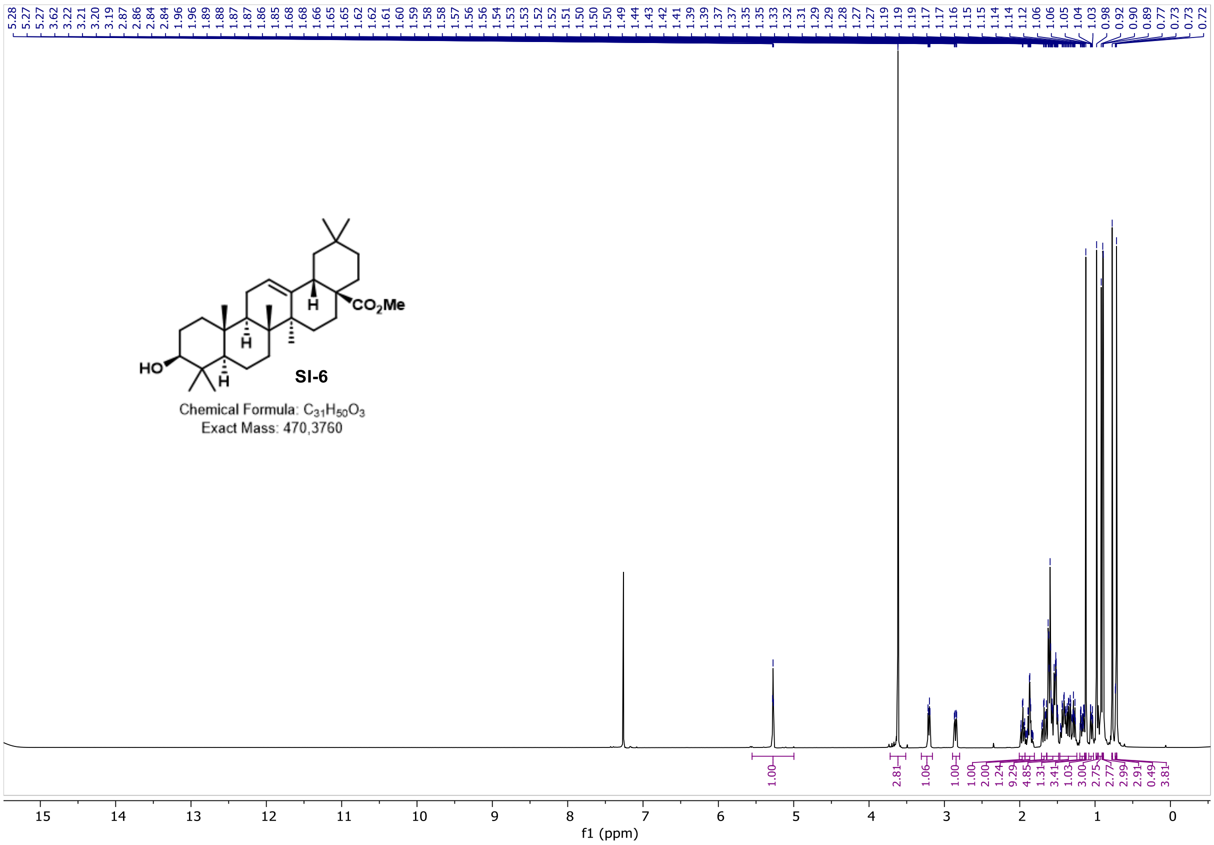

SI-6
Chemical Formula: $C_{31}H_{50}O_3$
Exact Mass: 470,3760
f1 (ppm)

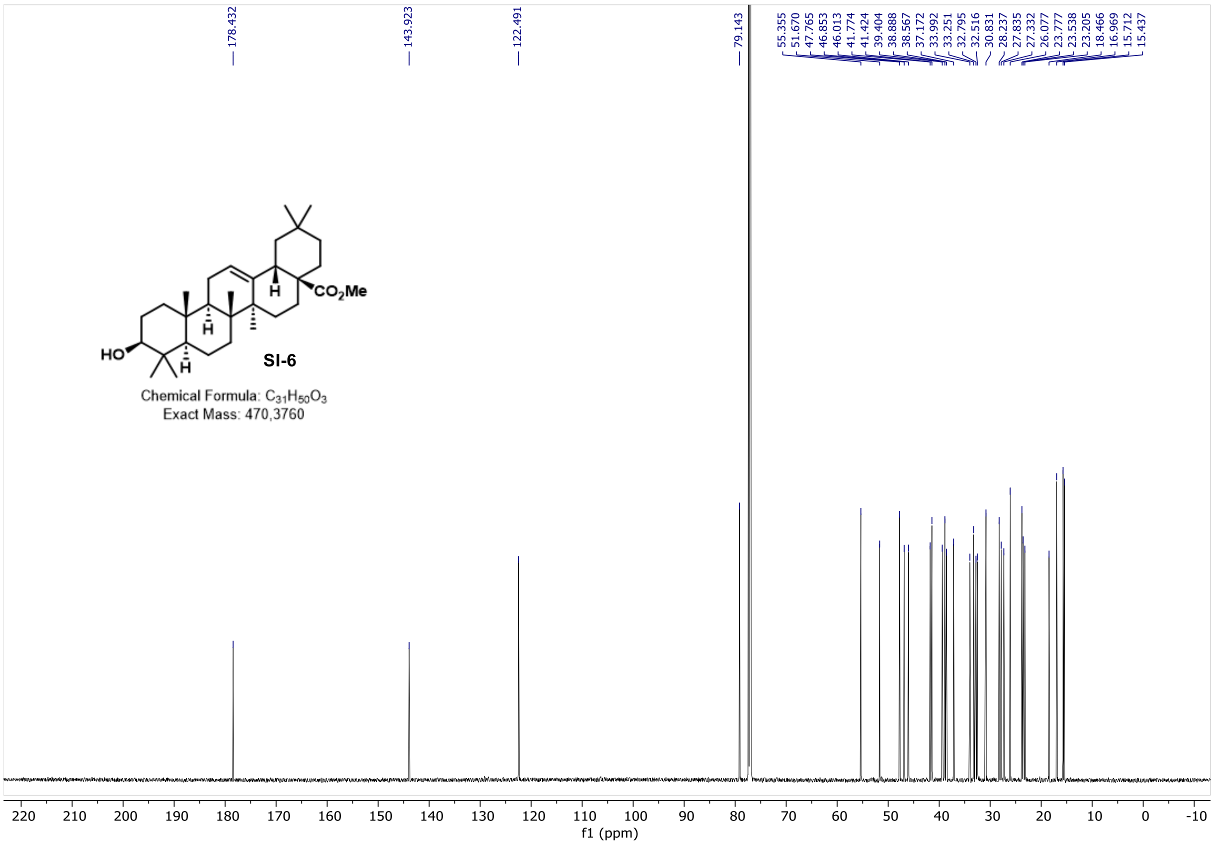

178.432
143.923
122.491
79.143
55.355
51.670
47.765
46.853
46.013
41.774
41.424
39.404
38.888
38.567
37.172
33.992
33.251
32.795
32.516
30.831
28.237
27.835
27.332
26.077
23.777
23.538
23.205
18.466
16.969
15.712
15.437
SI-6
Chemical Formula: $C_{31}H_{50}O_3$
Exact Mass: 470,3760
f1 (ppm)

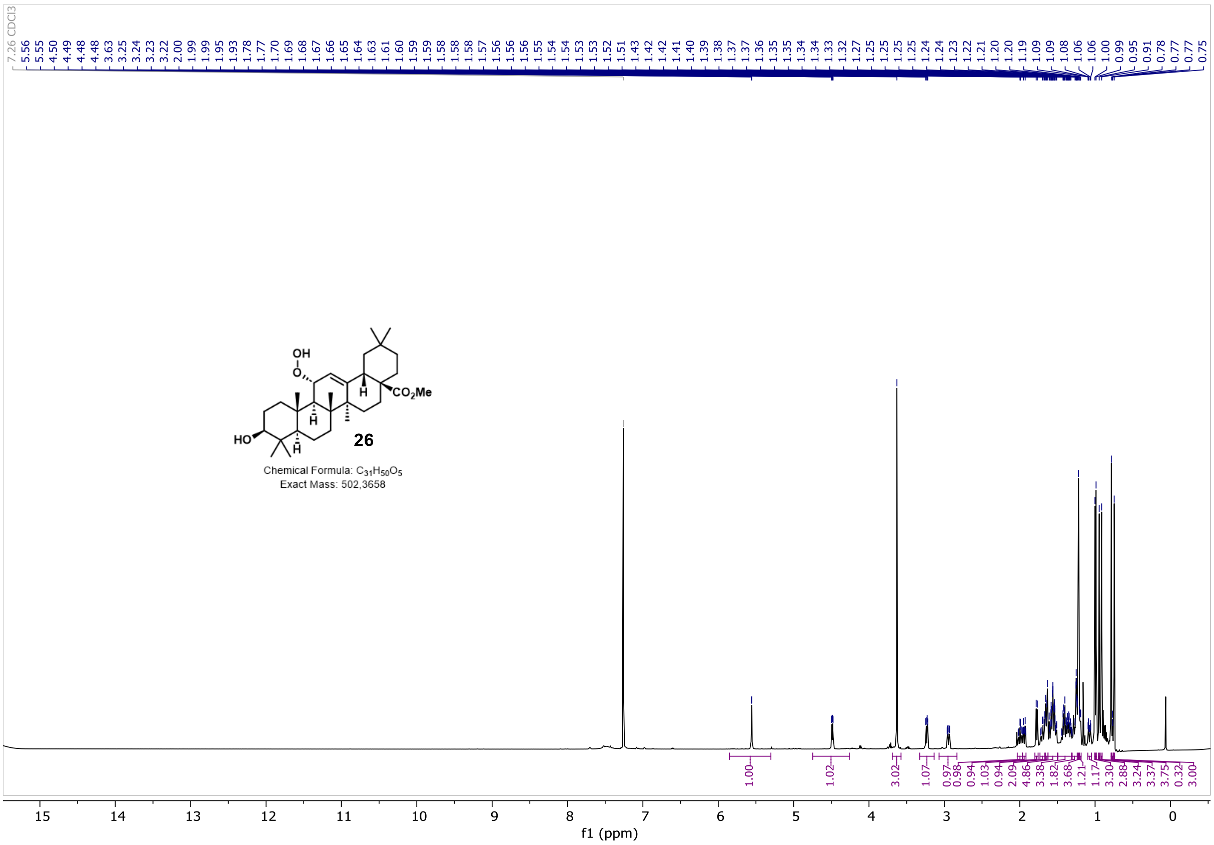
7.26 CDCl3
OH
O
H
CO2Me
H
HO
H
26
Chemical Formula: C31H50O5
Exact Mass: 502,3658
1.00
1.02
3.02
1.07
0.97
0.98
0.94
1.03
0.94
2.09
4.86
3.38
1.82
3.68
1.21
1.17
3.30
2.88
3.24
3.37
3.75
0.32
3.00
15 14 13 12 11 10 9 8 7 6 5 4 3 2 1 0
f1 (ppm)

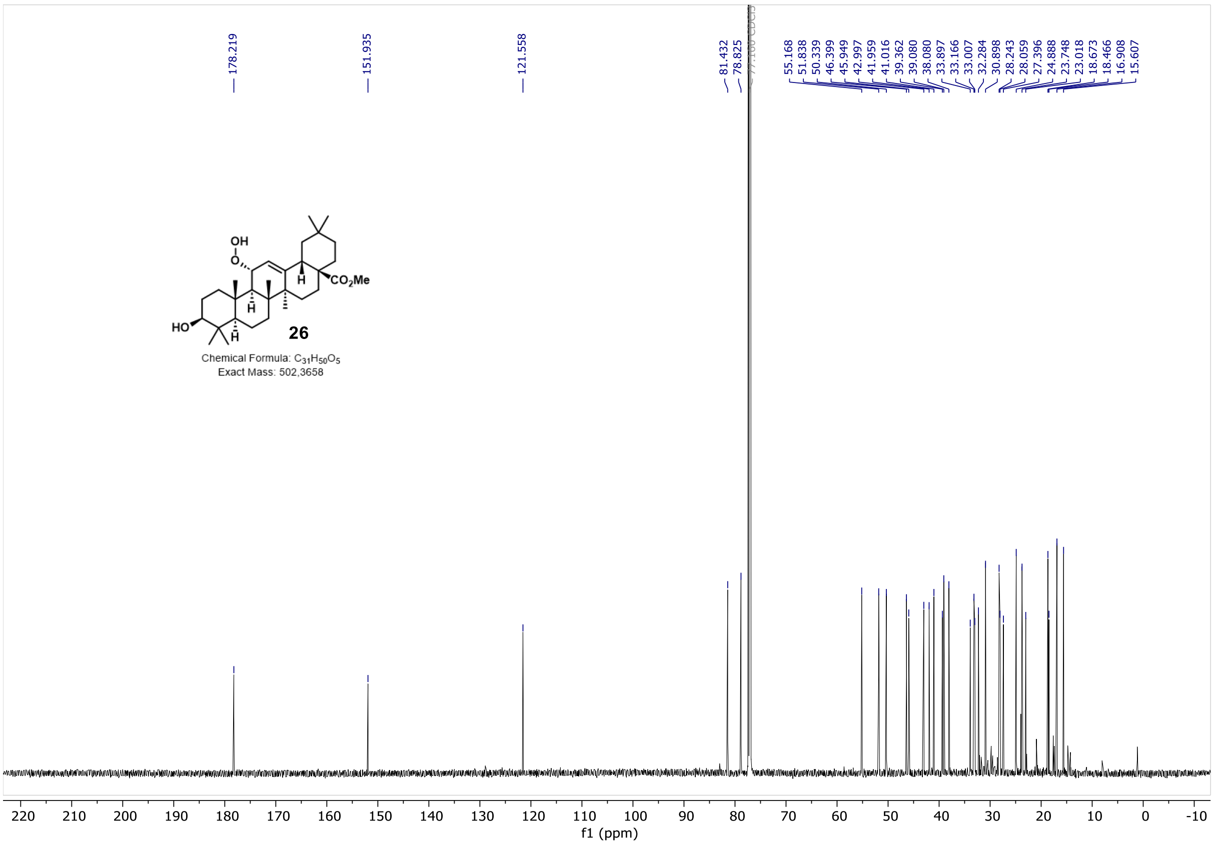
178.219
151.935
121.558
81.432
78.825
77.160 CDCl3
55.168
51.838
50.339
46.399
45.949
42.997
41.959
41.016
39.362
39.080
38.080
33.897
33.166
33.007
32.284
30.898
28.243
28.059
27.396
24.888
23.748
23.018
18.673
18.466
16.908
15.607
OH
O
H
CO2Me
H
HO
H
26
Chemical Formula: C31H50O5
Exact Mass: 502,3658
220 210 200 190 180 170 160 150 140 130 120 110 100 90 80 70 60 50 40 30 20 10 0 -10
f1 (ppm)

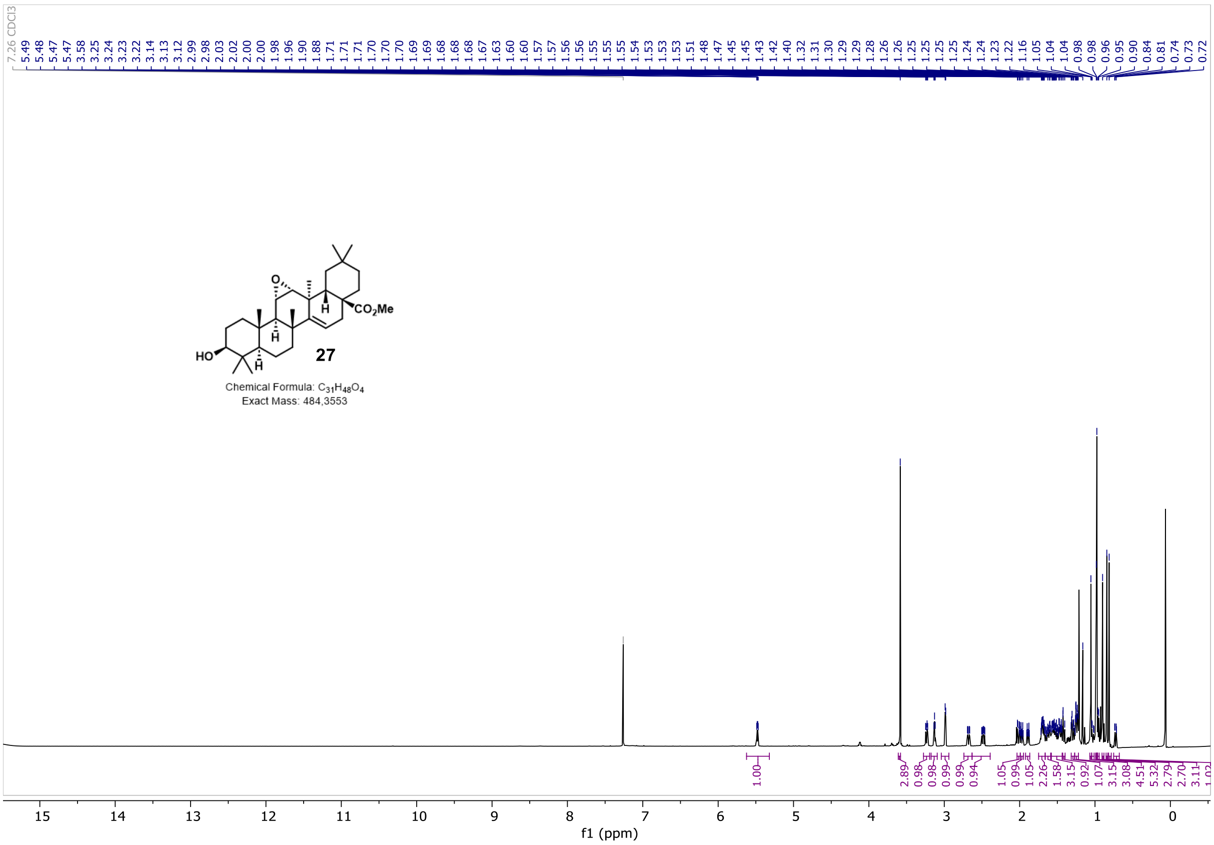
7.26 CDCl3
5.49 5.48 5.47 5.47 3.58 3.25 3.24 3.23 3.22 3.14 3.13 3.12 2.99 2.98 2.03 2.02 2.00 2.00 1.98 1.96 1.90 1.88 1.71 1.71 1.71 1.70 1.70 1.69 1.69 1.68 1.68 1.68 1.67 1.63 1.60 1.60 1.57 1.57 1.56 1.56 1.55 1.55 1.55 1.54 1.53 1.53 1.53 1.53 1.51 1.48 1.47 1.45 1.45 1.43 1.42 1.40 1.32 1.31 1.30 1.29 1.29 1.28 1.26 1.26 1.25 1.25 1.25 1.25 1.24 1.24 1.23 1.22 1.16 1.05 1.04 1.04 0.98 0.98 0.96 0.95 0.95 0.90 0.84 0.81 0.74 0.73 0.72
HO
H
H
H
O
CO2Me
27
Chemical Formula: C31H48O4
Exact Mass: 484,3553
1.00
2.89 0.98 0.98 0.99 0.94 1.05 0.99 1.05 2.26 1.58 3.15 0.92 1.07 3.15 3.08 4.51 5.32 2.79 2.70 3.11 1.02
15 14 13 12 11 10 9 8 7 6 5 4 3 2 1 0
f1 (ppm)

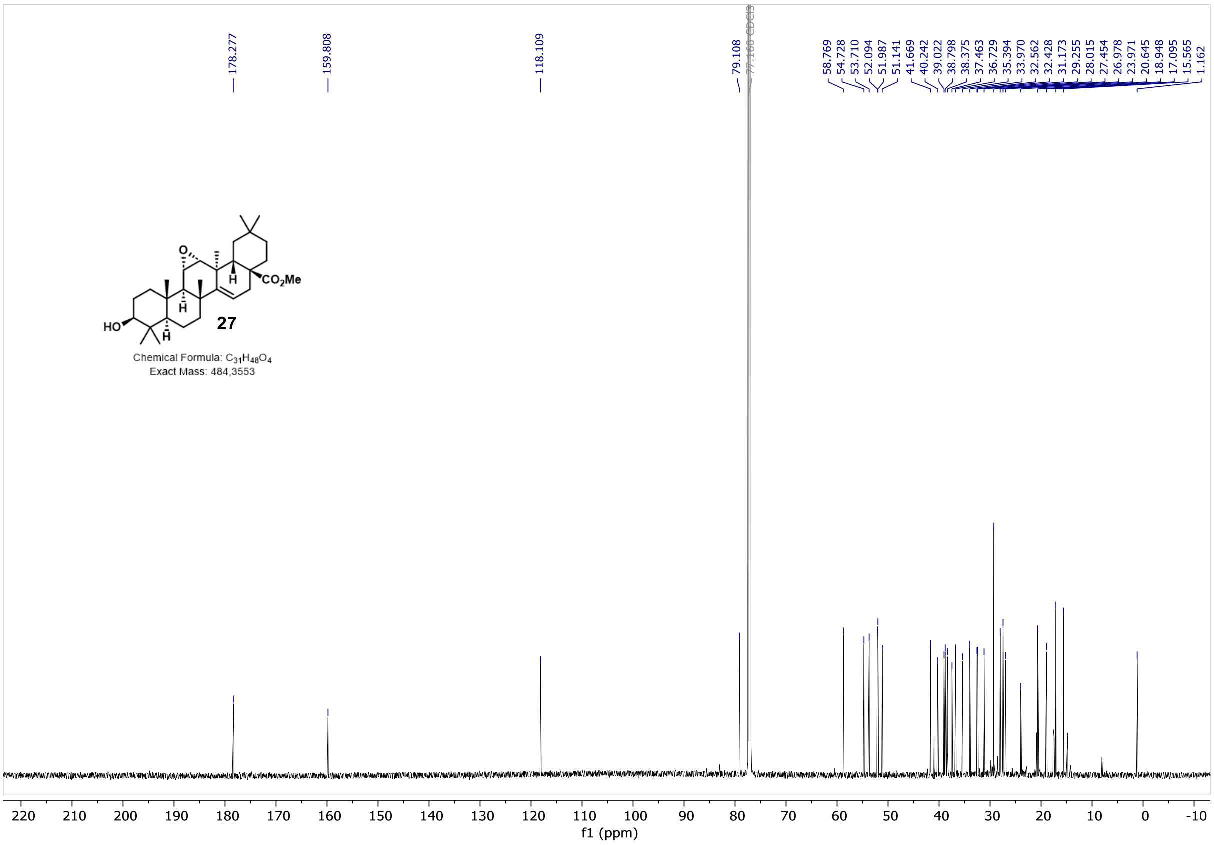
178.277
159.808
118.109
79.108
77.160 CDCl3
58.769 54.728 53.710 52.094 51.987 51.141 41.669 40.242 39.022 38.798 38.375 37.463 36.729 35.394 33.970 32.562 32.428 31.173 29.255 28.015 27.454 26.978 23.971 20.645 18.948 17.095 15.565 1.162
HO
H
H
H
O
CO2Me
27
Chemical Formula: C31H48O4
Exact Mass: 484,3553
220 210 200 190 180 170 160 150 140 130 120 110 100 90 80 70 60 50 40 30 20 10 0 -10
f1 (ppm)

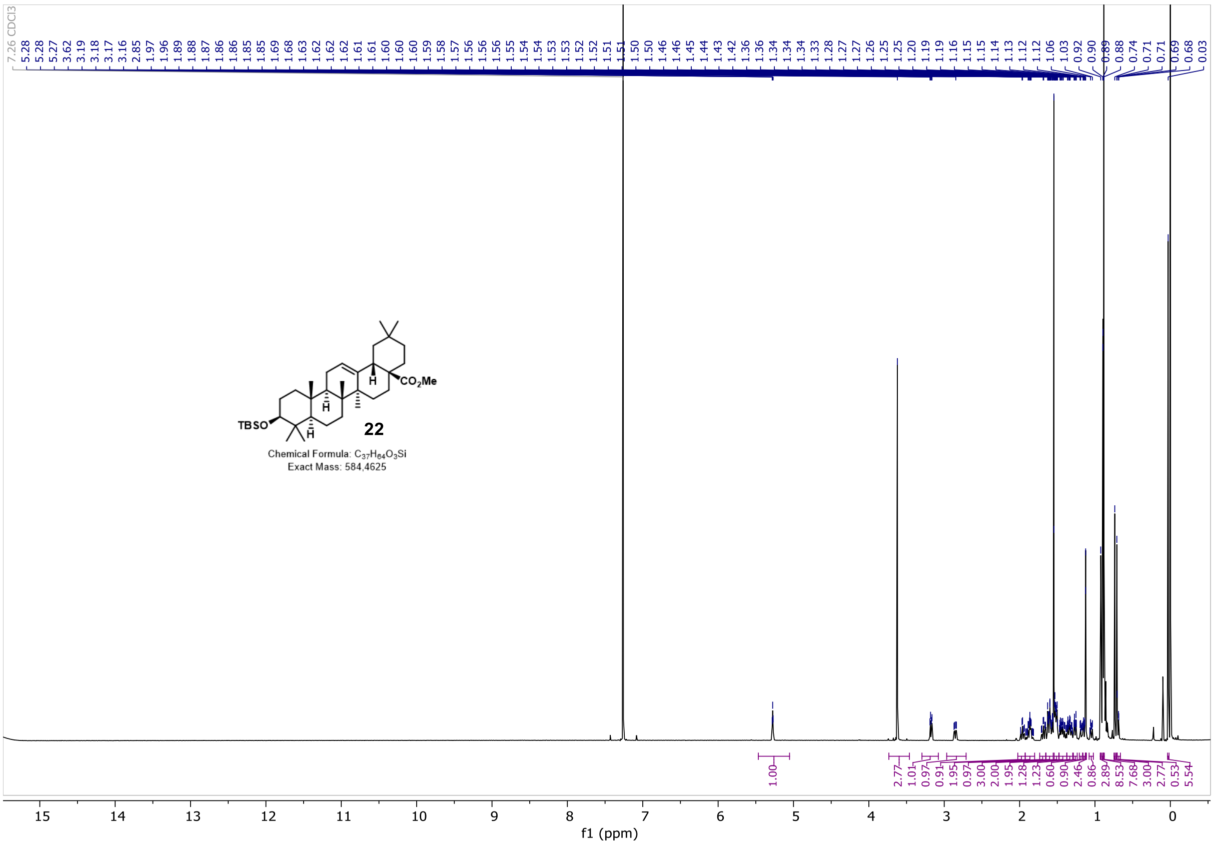

7.26 CDCl3
5.28
5.28
5.27
3.62
3.19
3.18
3.17
3.16
2.85
1.97
1.96
1.89
1.88
1.87
1.86
1.86
1.85
1.69
1.68
1.63
1.62
1.62
1.62
1.61
1.61
1.60
1.60
1.59
1.58
1.57
1.56
1.56
1.55
1.54
1.54
1.53
1.53
1.52
1.52
1.51
1.51
1.50
1.50
1.46
1.46
1.45
1.44
1.43
1.42
1.36
1.36
1.34
1.34
1.34
1.33
1.28
1.27
1.27
1.26
1.25
1.25
1.20
1.19
1.19
1.16
1.15
1.15
1.14
1.13
1.12
1.12
1.06
1.03
0.92
0.90
0.89
0.88
0.74
0.71
0.71
0.69
0.68
0.03
TBSO
H
H
H
CO_2Me
22
Chemical Formula: $C_{37}H_{64}O_3Si$
Exact Mass: 584.4625
1.00
2.77
1.01
0.97
0.91
1.95
0.97
3.00
2.00
1.95
1.28
1.23
0.60
0.90
2.46
0.86
2.89
8.53
7.68
3.00
2.77
0.53
5.54
15
14
13
12
11
10
9
8
7
6
5
4
3
2
1
0
f1 (ppm)

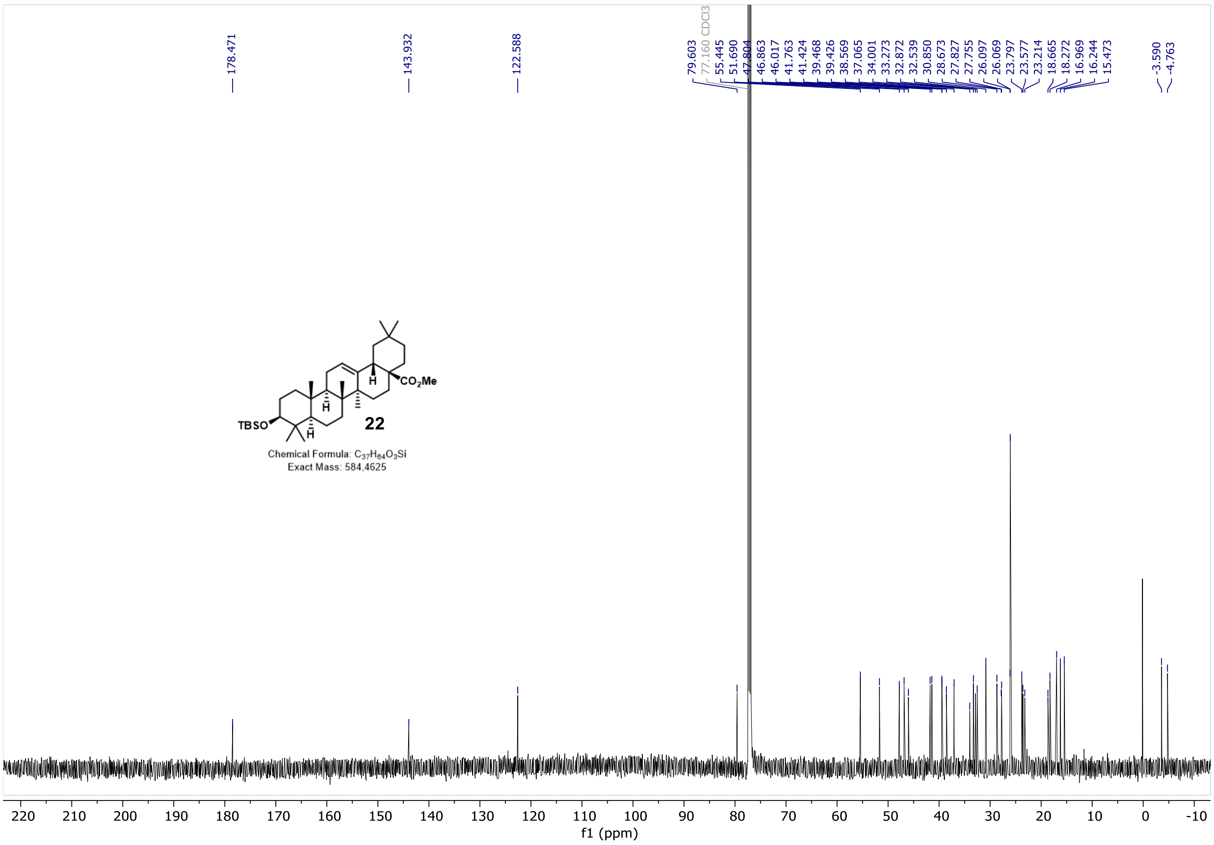

178.471
143.932
122.588
79.603
77.160 CDCl3
55.445
51.690
47.804
46.863
46.017
41.763
41.424
39.468
39.426
38.569
37.065
34.001
33.273
32.872
32.539
30.850
28.673
27.827
27.755
26.097
26.069
23.797
23.577
23.214
18.665
18.272
16.969
16.244
15.473
-3.590
-4.763
TBSO
H
H
H
CO_2Me
22
Chemical Formula: $C_{37}H_{64}O_3Si$
Exact Mass: 584.4625
220
210
200
190
180
170
160
150
140
130
120
110
100
90
80
70
60
50
40
30
20
10
0
-10
f1 (ppm)

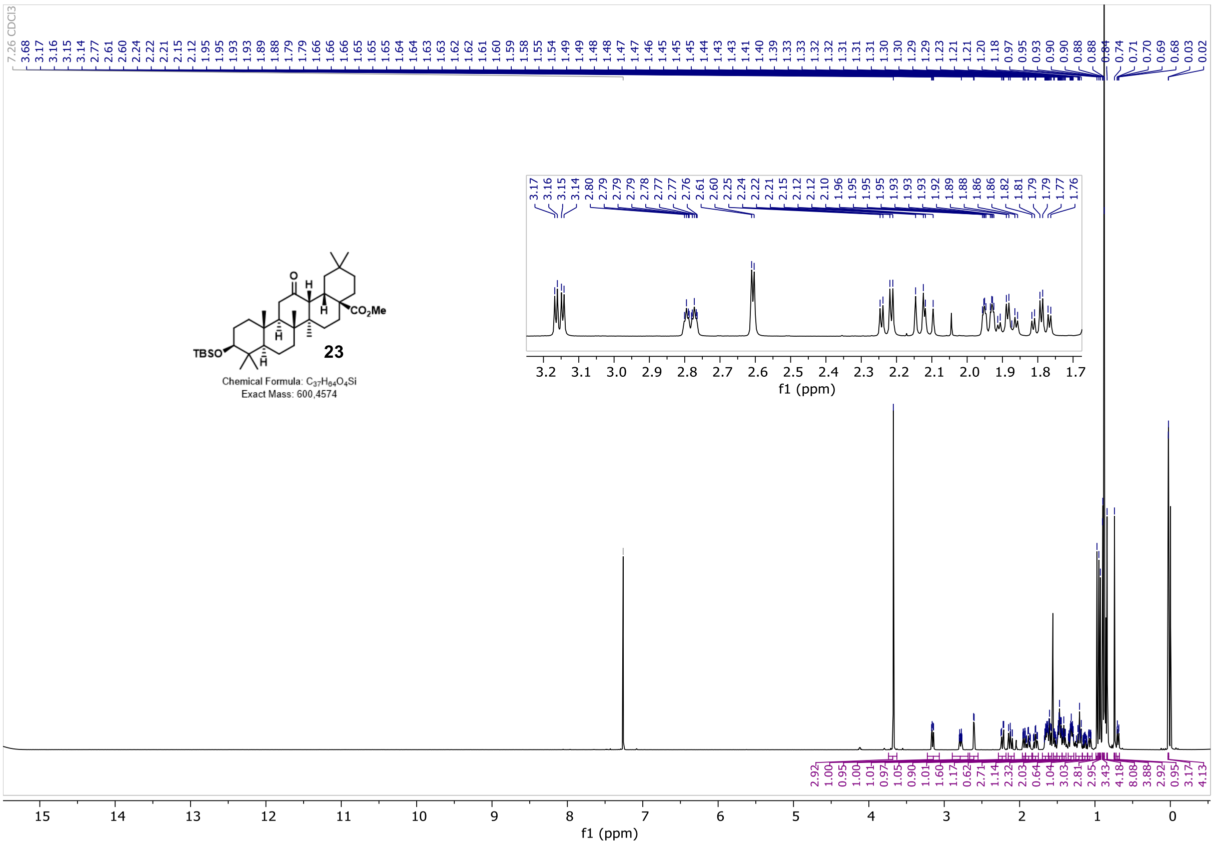
TBSO
CO2Me
23
Chemical Formula: C37H64O4Si
Exact Mass: 600,4574
7.26 CDCl3
f1 (ppm)

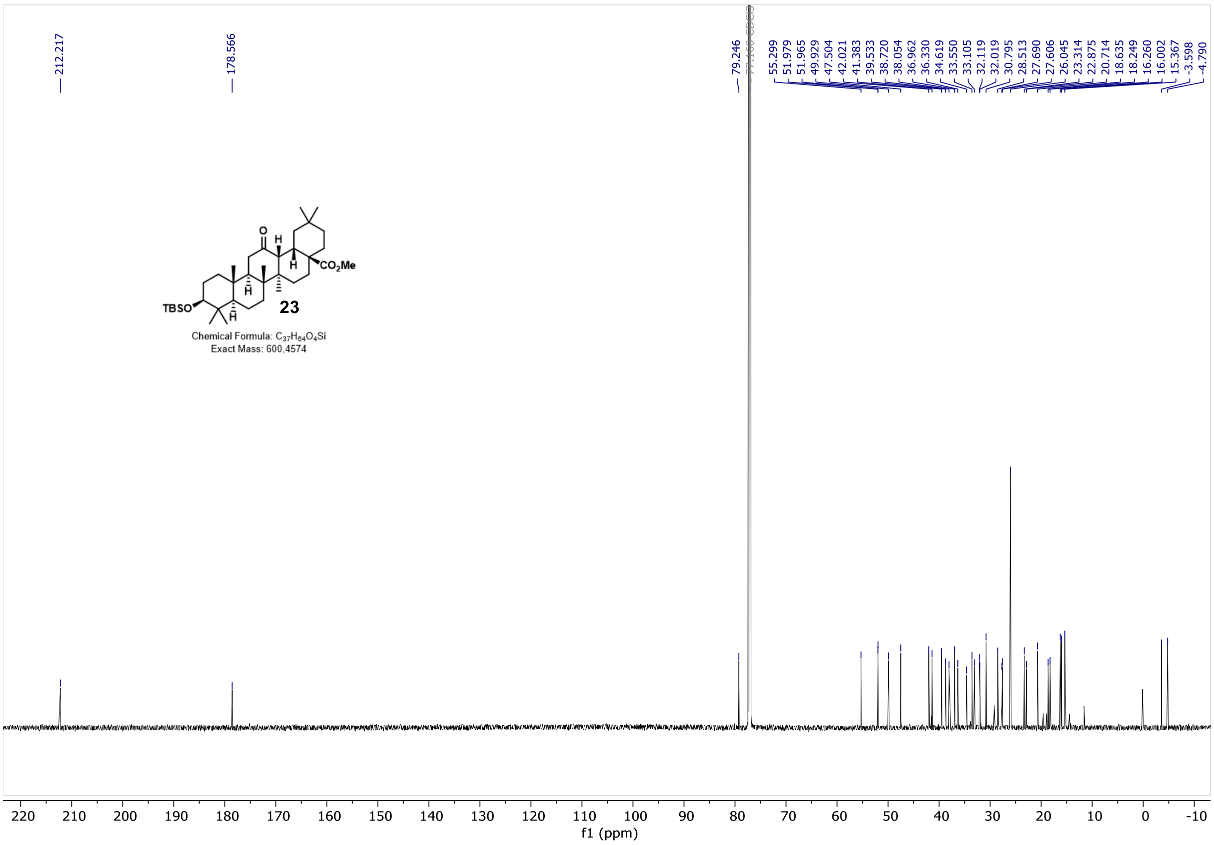
212.217
178.566
79.246
TBSO
CO2Me
23
Chemical Formula: C37H64O4Si
Exact Mass: 600,4574
f1 (ppm)

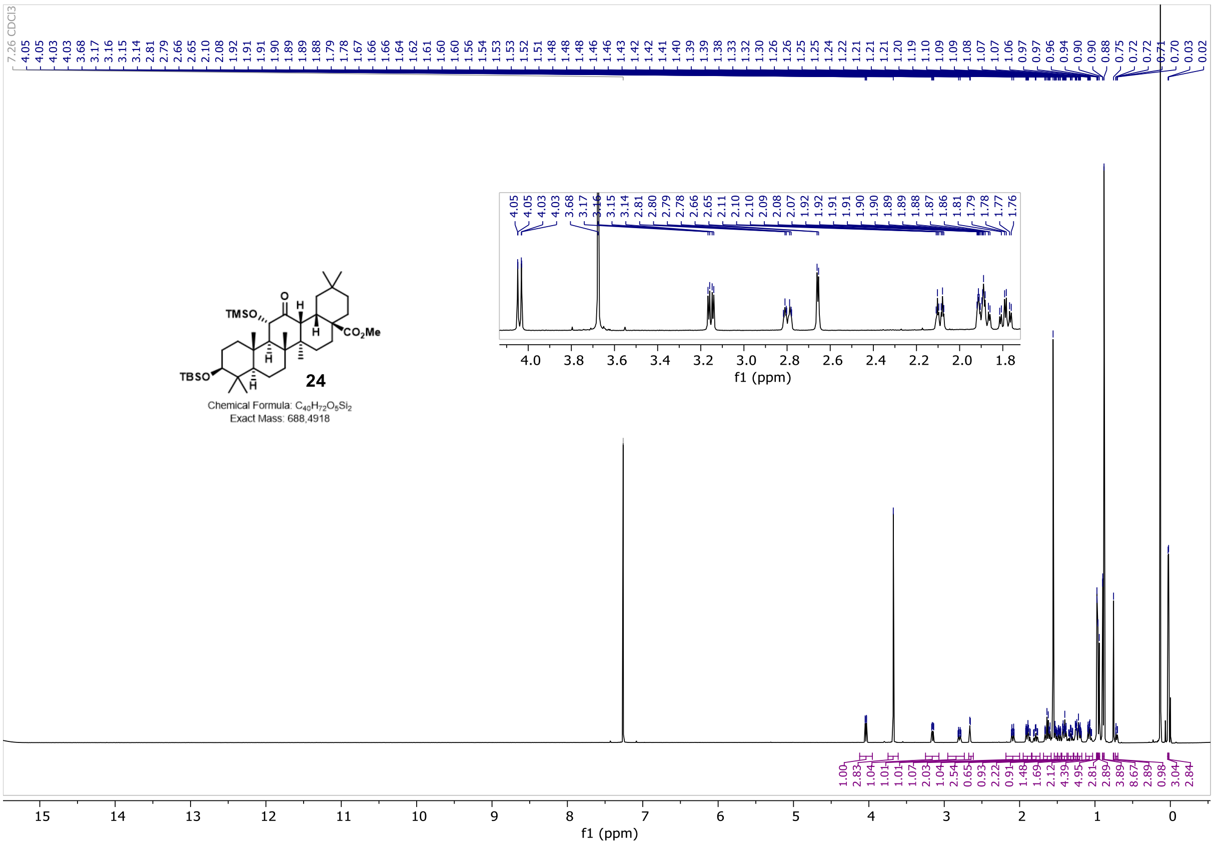
7.26 CDCl3
4.05 4.05 4.03 4.03 3.68 3.17 3.16 3.15 3.14 2.81 2.79 2.66 2.65 2.10 2.08 1.92 1.91 1.91 1.90 1.89 1.89 1.88 1.79 1.78 1.67 1.66 1.64 1.62 1.61 1.60 1.56 1.54 1.53 1.53 1.52 1.51 1.48 1.48 1.46 1.43 1.42 1.42 1.41 1.40 1.39 1.39 1.38 1.33 1.32 1.30 1.26 1.26 1.25 1.25 1.24 1.22 1.21 1.21 1.21 1.20 1.19 1.10 1.09 1.09 1.08 1.07 1.07 1.06 0.97 0.97 0.96 0.94 0.90 0.90 0.88 0.75 0.72 0.72 0.71 0.70 0.03 0.02
4.05 4.05 4.03 4.03 3.68 3.17 3.16 3.15 3.14 2.81 2.80 2.79 2.78 2.66 2.65 2.11 2.11 2.10 2.10 2.09 2.08 2.07 1.92 1.92 1.91 1.91 1.90 1.90 1.89 1.89 1.88 1.87 1.86 1.81 1.79 1.78 1.77 1.76
4.0 3.8 3.6 3.4 3.2 3.0 2.8 2.6 2.4 2.2 2.0 1.8
f1 (ppm)
TMSO
TBSO
H
CO2Me
24
Chemical Formula: C45H72O5Si2
Exact Mass: 688,4918
1.00 2.83 1.04 1.01 1.01 1.07 2.03 1.04 2.54 0.65 0.93 2.22 0.91 1.48 1.69 2.12 4.39 4.95 2.81 2.89 3.89 8.67 2.89 0.98 3.04 2.84
15 14 13 12 11 10 9 8 7 6 5 4 3 2 1 0
f1 (ppm)

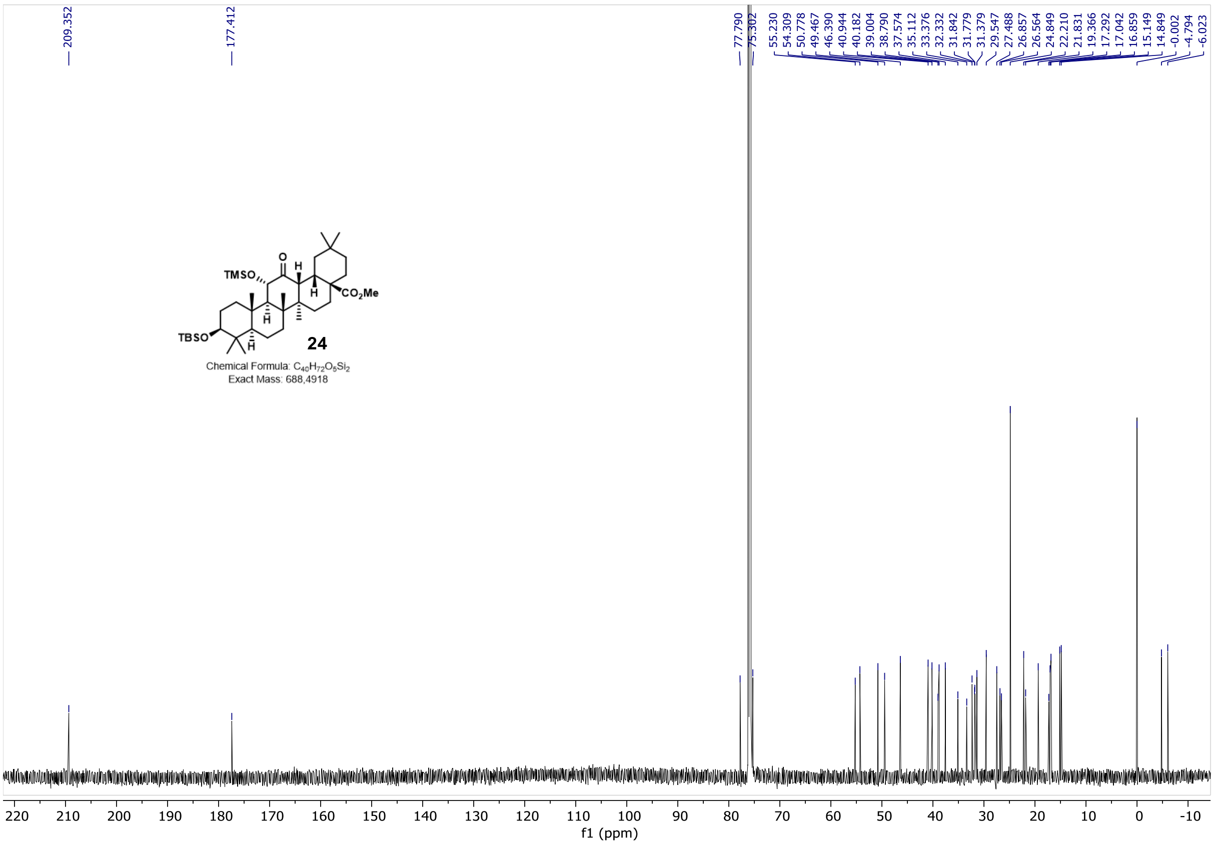
209.352
177.412
77.790 75.302
55.230 54.309 50.778 49.467 46.390 40.944 40.182 39.004 38.790 37.574 35.112 33.376 32.332 31.842 31.779 31.379 29.547 27.488 26.857 26.564 24.849 22.210 21.831 19.366 17.292 17.042 16.859 15.149 14.849 -0.002 -4.794 -6.023
TMSO
TBSO
H
CO2Me
24
Chemical Formula: C45H72O5Si2
Exact Mass: 688,4918
220 210 200 190 180 170 160 150 140 130 120 110 100 90 80 70 60 50 40 30 20 10 0 -10
f1 (ppm)

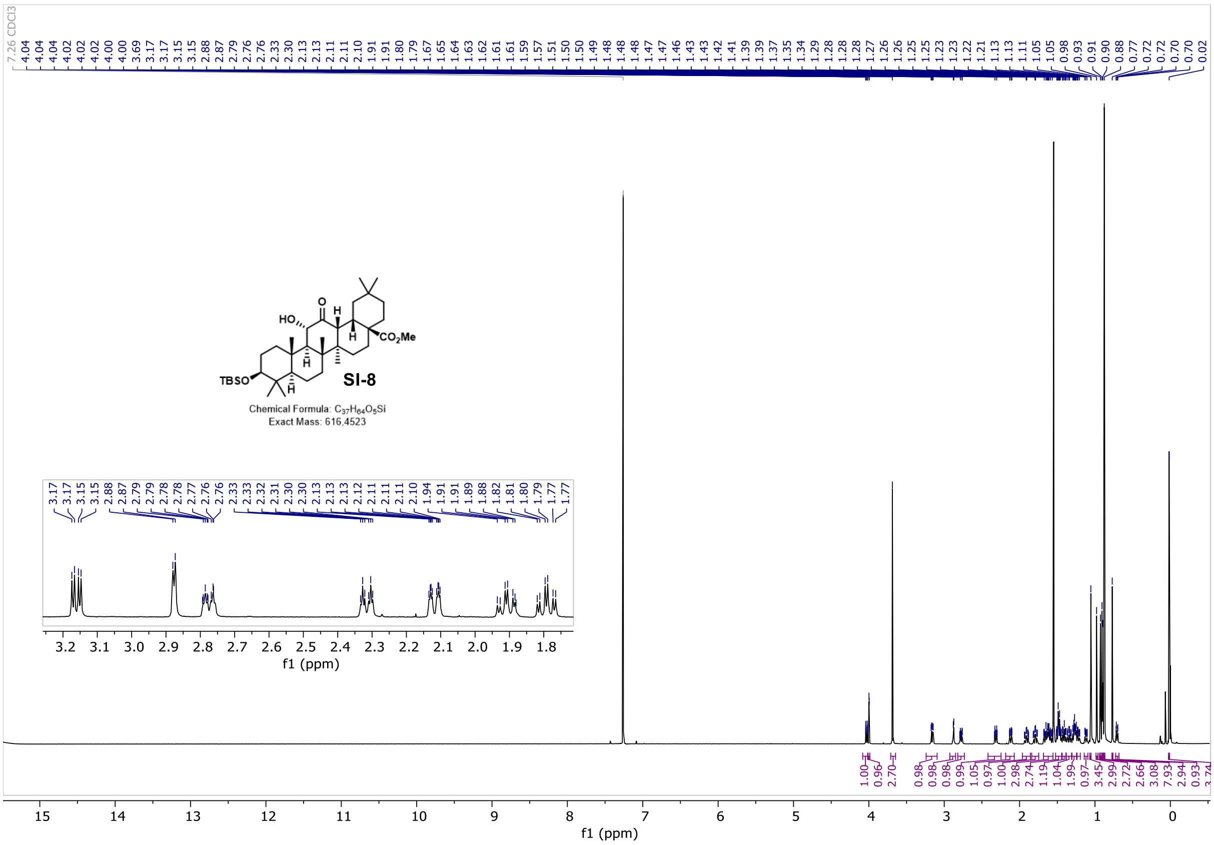
SI-8
Chemical Formula: $C_{37}H_{64}O_5Si$
Exact Mass: 616.4523
f1 (ppm)

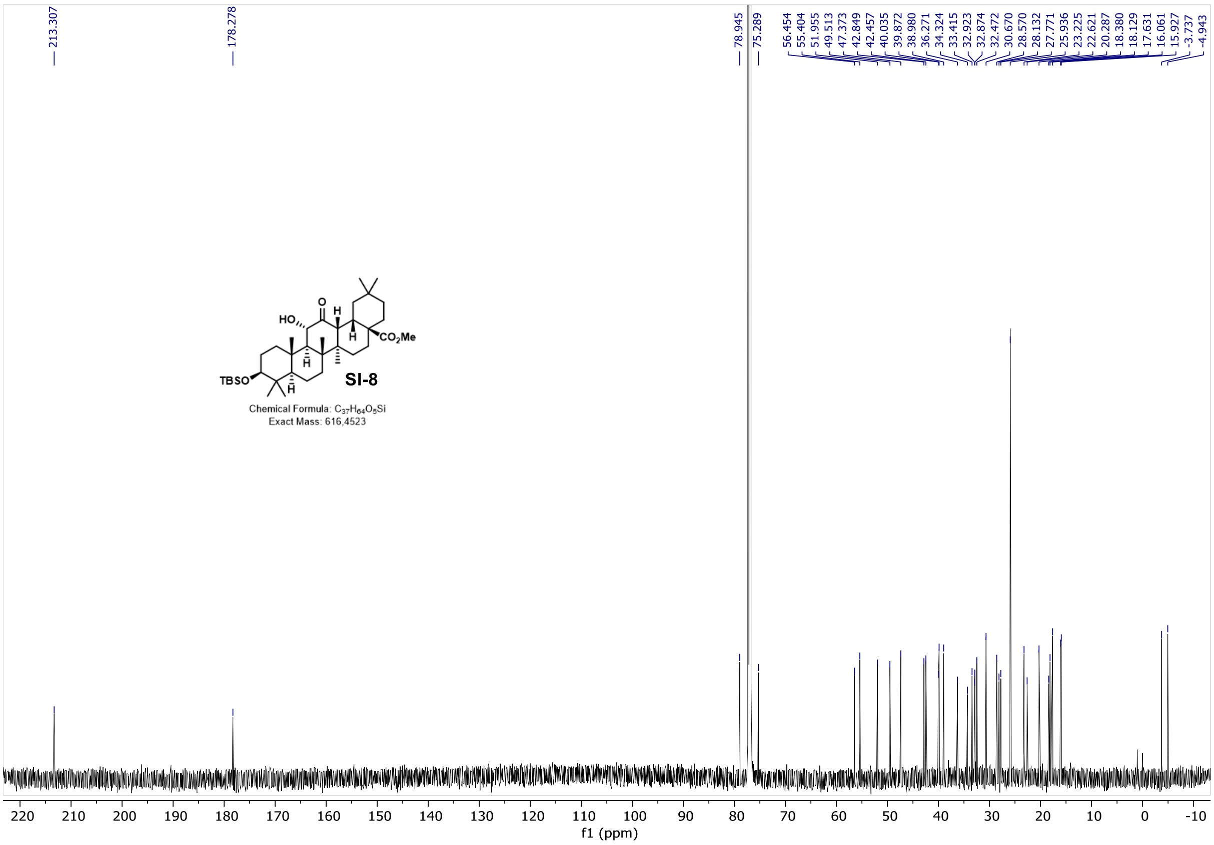
SI-8
Chemical Formula: $C_{37}H_{64}O_5Si$
Exact Mass: 616.4523
f1 (ppm)

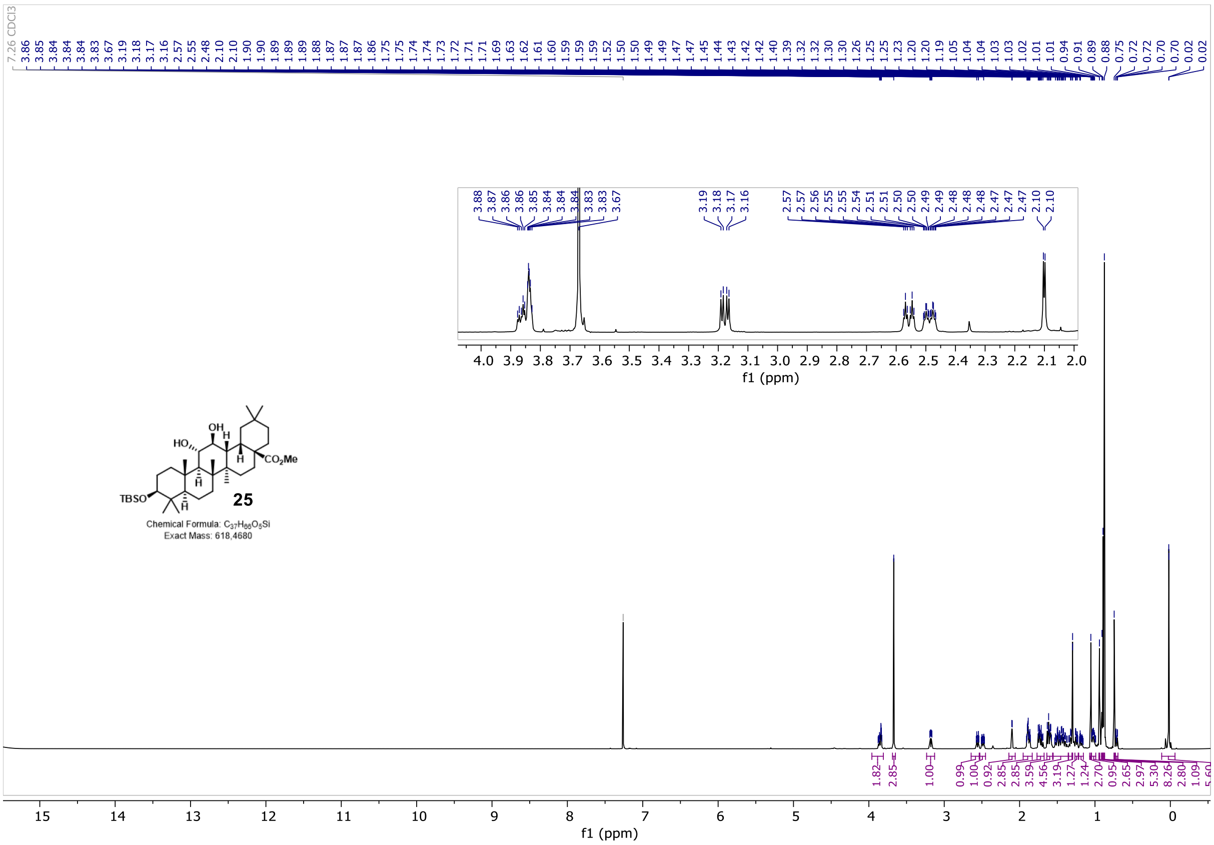

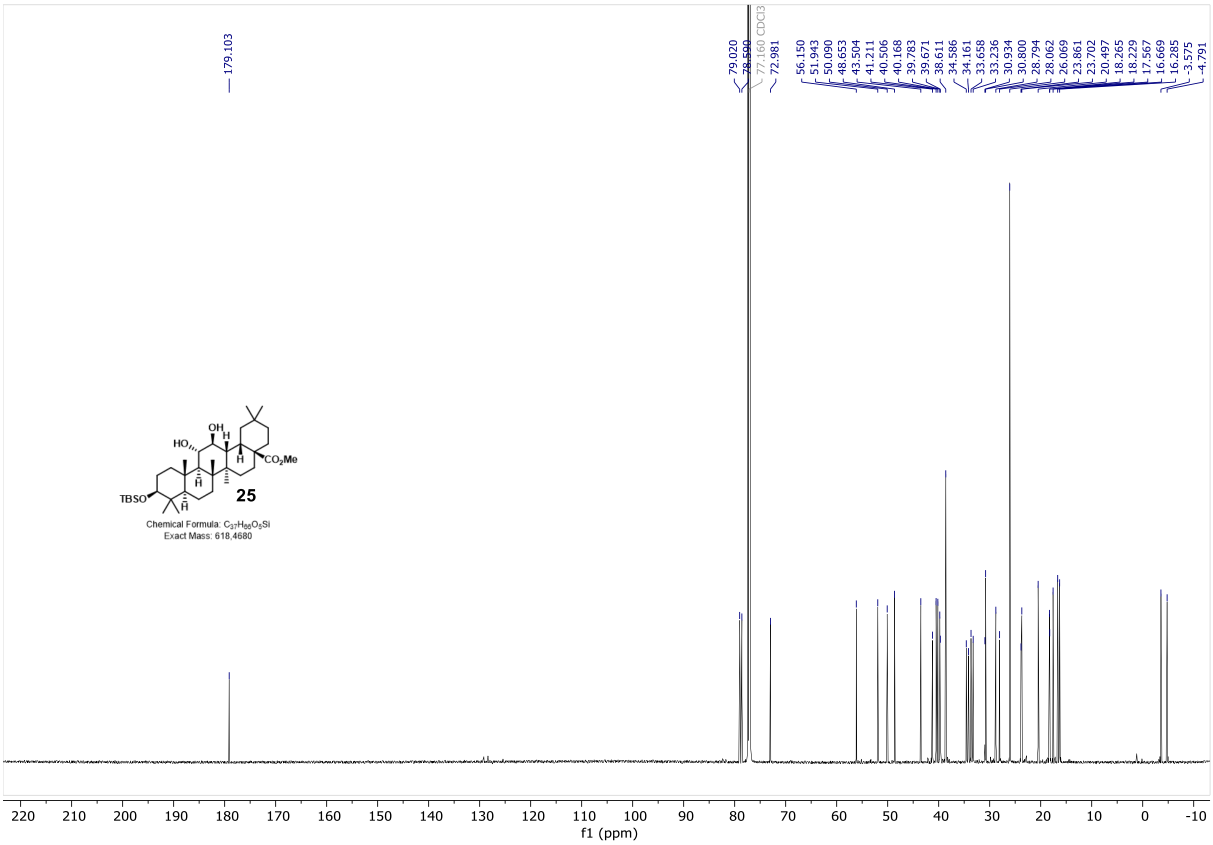

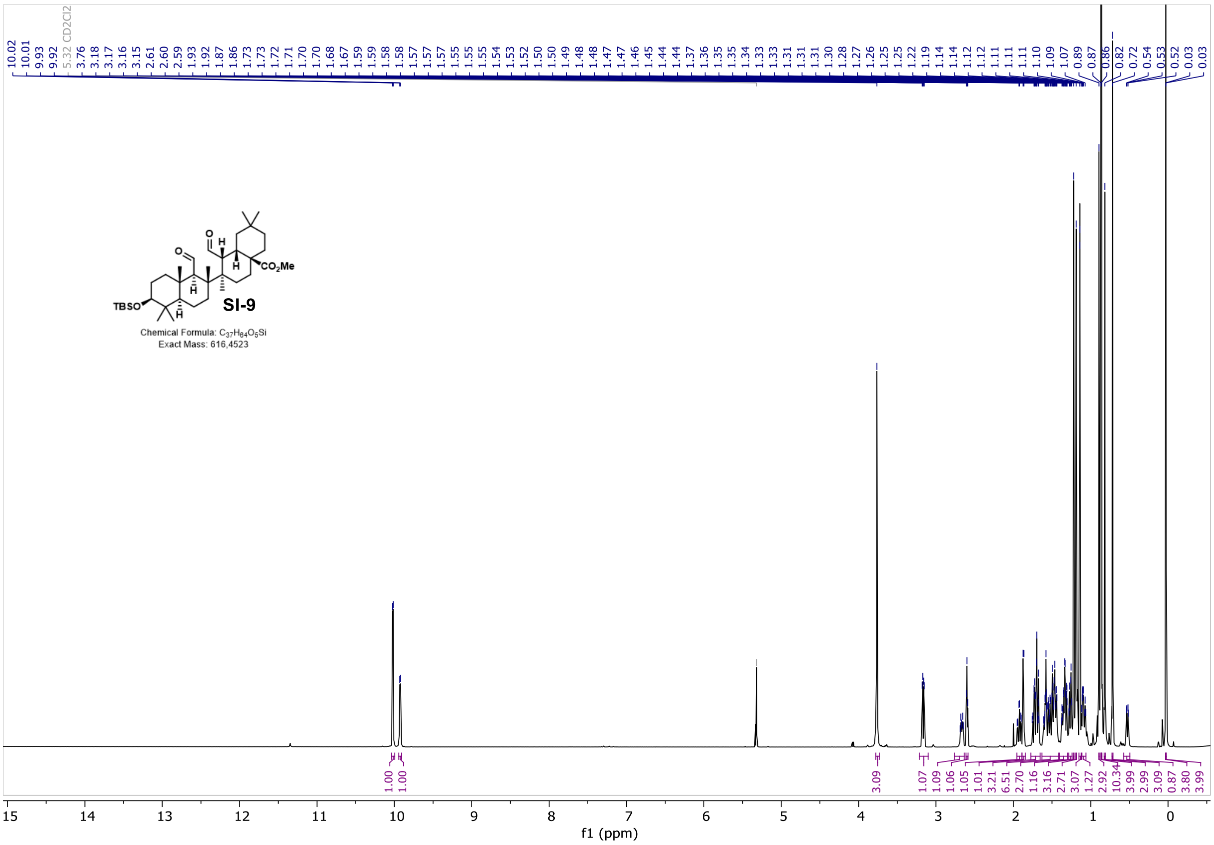
SI-9
Chemical Formula: $C_{37}H_{64}O_5Si$
Exact Mass: 616.4523
f1 (ppm)

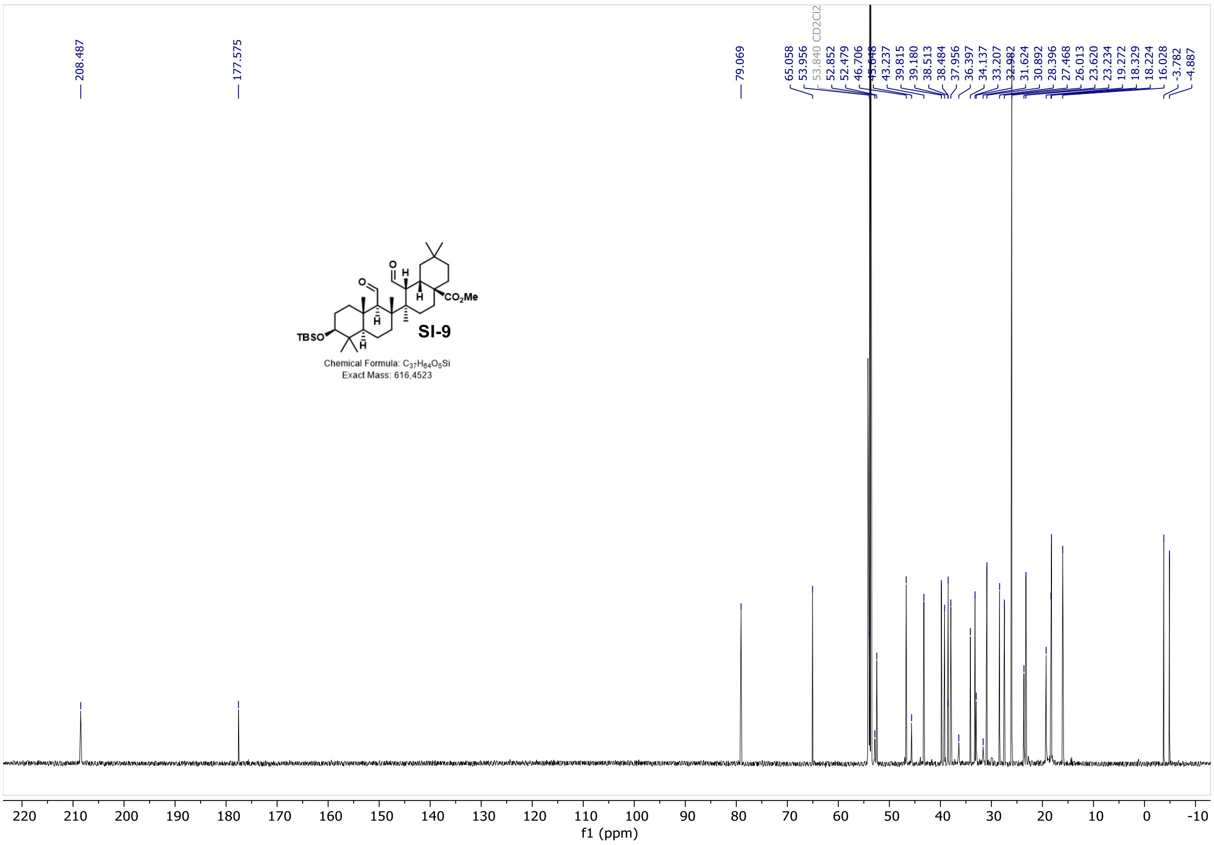
SI-9
Chemical Formula: $C_{37}H_{64}O_5Si$
Exact Mass: 616.4523
f1 (ppm)

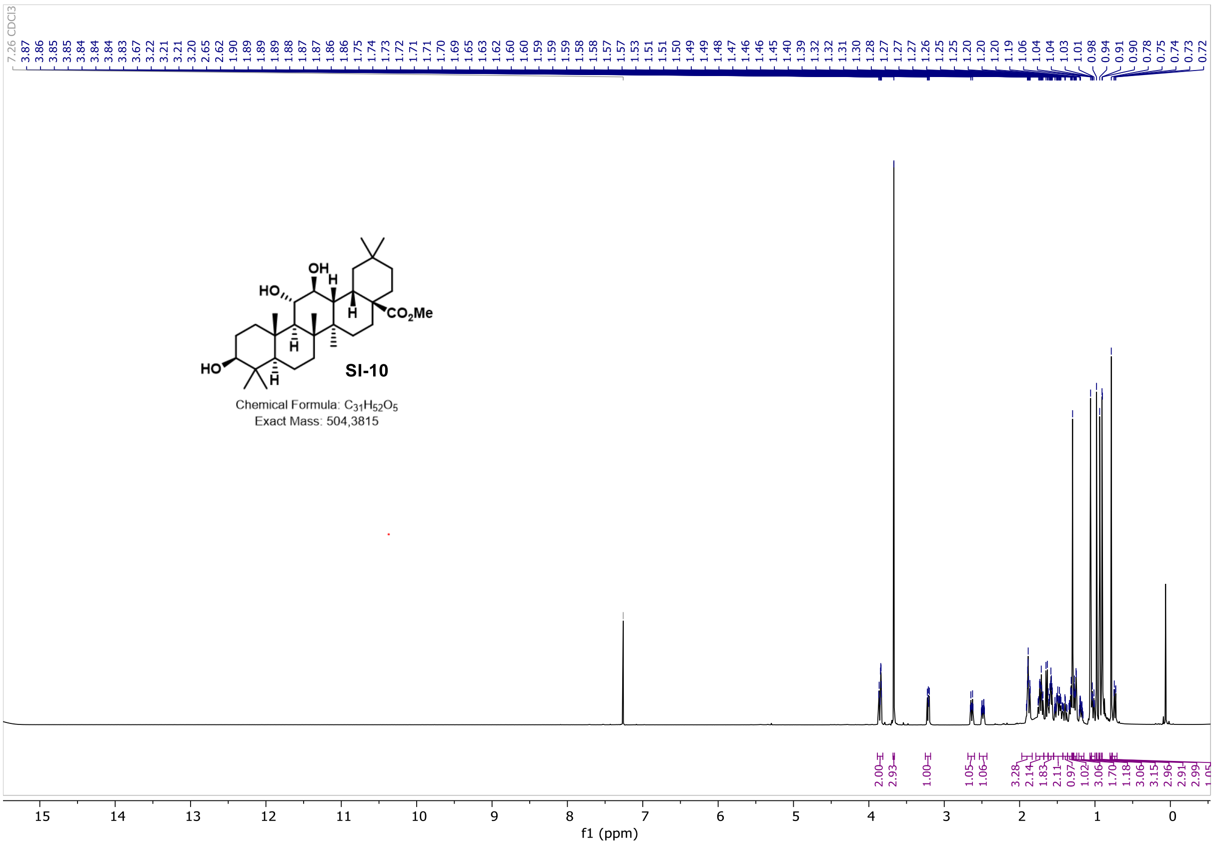
7.26 CDCl3
SI-10
Chemical Formula: $C_{31}H_{52}O_5$
Exact Mass: 504,3815
f1 (ppm)

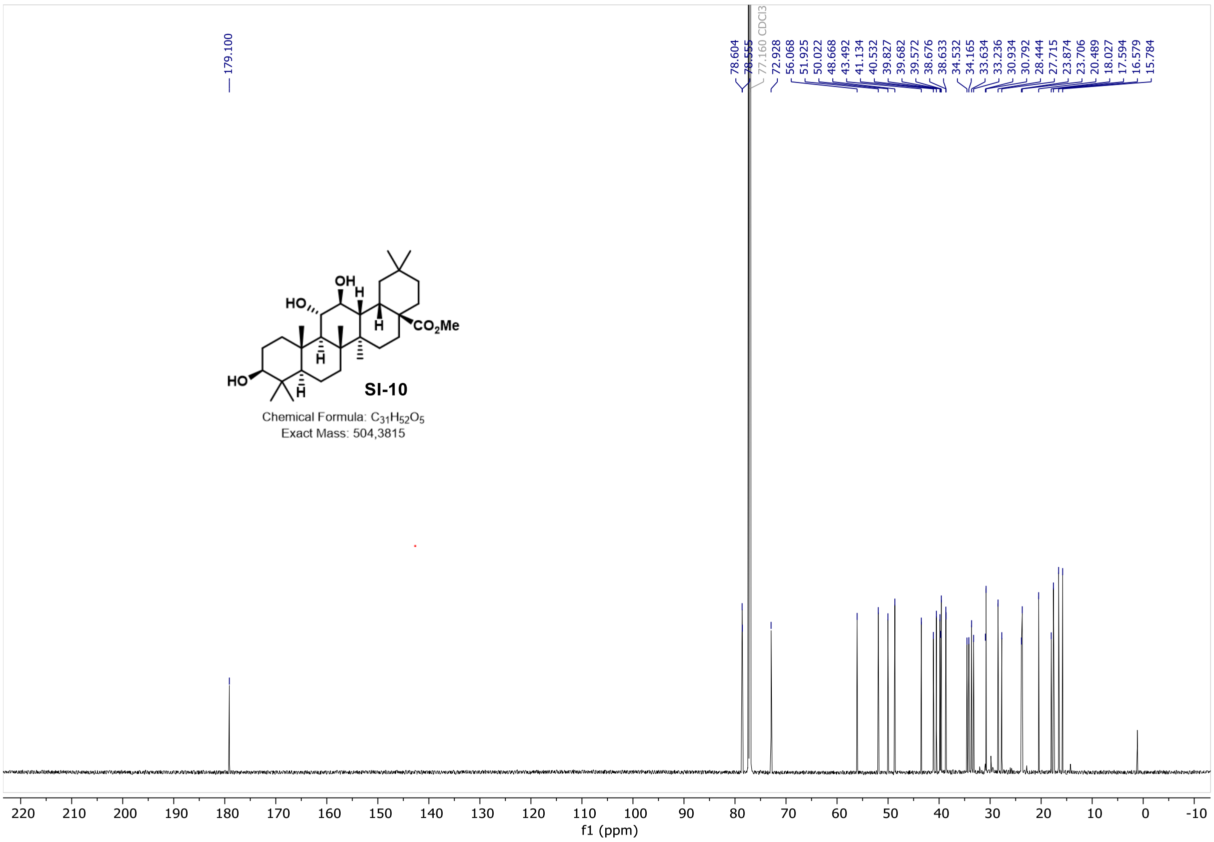
179.100
78.604
78.555
77.160 CDCl3
72.928
56.068
51.925
50.022
48.668
43.492
41.134
40.532
39.827
39.682
39.572
38.676
38.633
34.532
34.165
33.634
33.236
30.934
30.792
28.444
27.715
23.874
23.706
20.489
18.027
17.594
16.579
15.784
SI-10
Chemical Formula: $C_{31}H_{52}O_5$
Exact Mass: 504,3815
f1 (ppm)

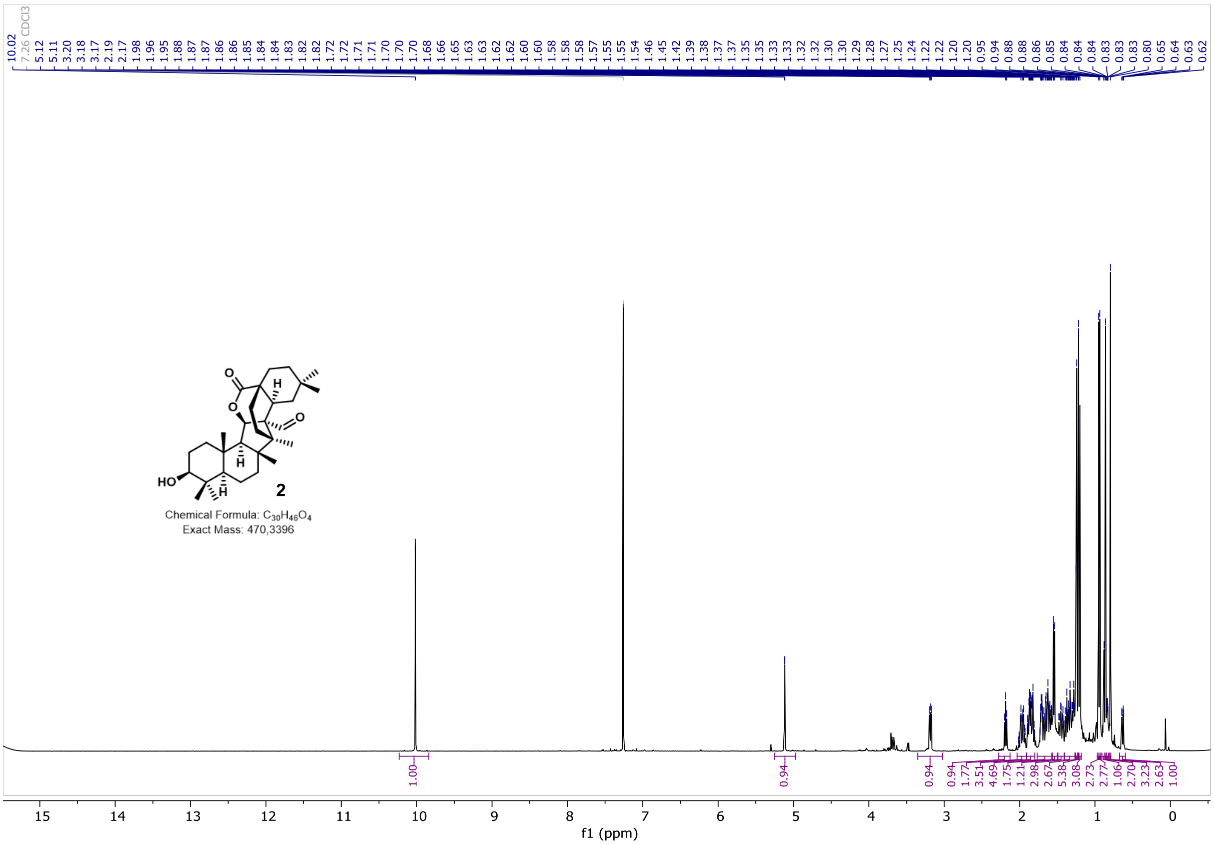
10.02
7.26 CDCl3
5.12
5.11
3.20
3.18
3.17
2.19
2.17
1.98
1.96
1.95
1.88
1.87
1.87
1.86
1.86
1.85
1.84
1.84
1.83
1.82
1.82
1.72
1.72
1.71
1.71
1.70
1.70
1.70
1.68
1.66
1.65
1.63
1.63
1.62
1.60
1.58
1.58
1.58
1.57
1.55
1.55
1.54
1.46
1.45
1.42
1.39
1.38
1.37
1.37
1.35
1.35
1.33
1.33
1.32
1.32
1.30
1.30
1.29
1.28
1.27
1.25
1.24
1.22
1.22
1.20
1.20
0.95
0.94
0.88
0.88
0.86
0.85
0.84
0.84
0.84
0.83
0.83
0.83
0.80
0.65
0.64
0.63
0.62
HO
H
H
H
2
Chemical Formula: $C_{30}H_{46}O_4$
Exact Mass: 470,3396
1.00
0.94
0.94
1.74
3.51
4.69
1.75
1.21
2.98
2.67
5.38
3.08
2.73
2.77
1.06
2.70
3.23
2.63
1.00
15 14 13 12 11 10 9 8 7 6 5 4 3 2 1 0
f1 (ppm)

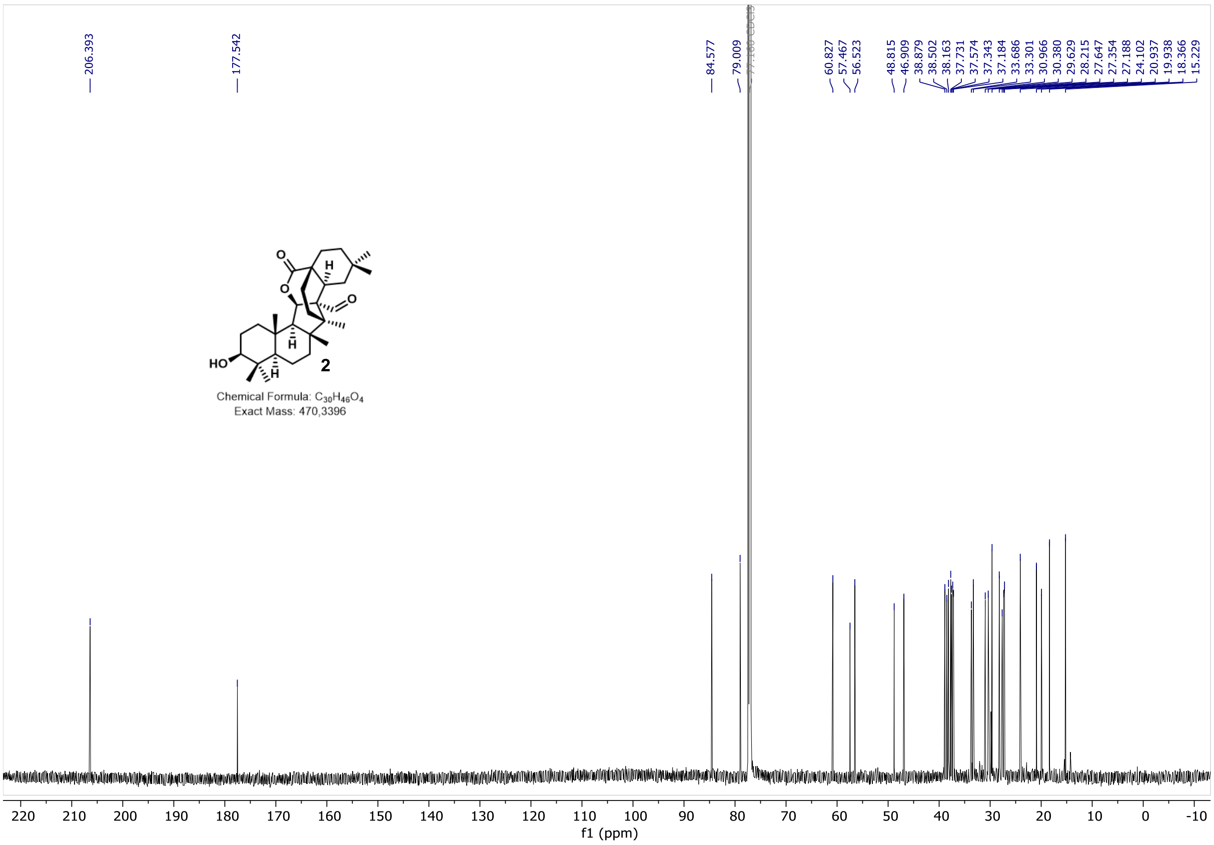
206.393
177.542
84.577
79.009
77.160 CDCl3
60.827
57.467
56.523
48.815
46.909
38.879
38.502
38.163
37.731
37.574
37.343
37.184
33.686
33.301
30.966
30.380
29.629
28.215
27.647
27.354
27.188
24.102
20.937
19.938
18.366
15.229
HO
H
H
H
2
Chemical Formula: $C_{30}H_{46}O_4$
Exact Mass: 470,3396
220 210 200 190 180 170 160 150 140 130 120 110 100 90 80 70 60 50 40 30 20 10 0 -10
f1 (ppm)

References

1. N. V. Heise, D. Major, S. Hoenke, M. Kozubek, I. Serbian, R. Csuk, *Molecules* **2022**, 27, 7, 2220.
2. T. C. Denner, N. V. Heise, I. Serbian, A. Angeli, C. T. Supuran, R. Csuk, *Steroids* **2024**, 205, 109381.
3. H. Itokawa, H. Nakajima, *Phytochemistry* **1981**, 20, 2539.
4. X. Sun, J. Chen, T. Ritter, *Nat. Chem.* **2018**, 10, 1229.
5. Q. Zhang, Z. Lu, X. Li, Y. Zheng, D. Yao, Y. Gu, C. Huo, B. Cong, *Chem. Nat. Compd.* **2015**, 51, 1, 178.
6. A. X. Huang, Z. Xiong, E. J. Corey, *J. Am. Chem. Soc.* **1999**, 121, 9999.
7. K. V. Rao, P. K. Bose, *J. Org. Chem.* **1962**, 27, 1470.
8. B. Hu, Y. Zhao, D. Xiong, Y. He, Z. Zhou, P. Zhu, Z. Wang, Y. Wang, L. Zhao, X. Luo, *Org. Lett.* **2021**, 23, 4158.
9. P. Chen, H. Wu, Y. Li, Y. Tang, *Tetrahedron Lett.* **2020**, 61, 33, 152172.
10. I. Okamoto, T. Takeya, Y. Kagawa, E. Kotani, *Chem. Pharm. Bull.* **2000**, 48, 120.
11. A. Loesche, A. Köwitsch, S. D. Lucas, Z. Al-Halabi, W. Sippl, A. Al-Harrasi, R. Csuk, *Bioorg. Chem.* **2019**, 85, 23.
12. C. Luo, L.-N. Zhan, Y. Shi, *Org. Lett.* **2023**, 25, 5735.
13. R. Li, J. Wu, *Org. Lett.* **2023**, 25, 6278.
14. L. He, W. Zhang, X. Zhang, X. Wu, Y. Han, J. Yan, W. Xie, *Org. Biomol. Chem.* **2023**, 21, 9346.
